# Novel Piperazine Derivatives of Vindoline as Anticancer Agents

**DOI:** 10.3390/ijms25147929

**Published:** 2024-07-19

**Authors:** Bernadett Zsoldos, Nóra Nagy, Viktória Donkó-Tóth, Péter Keglevich, Márton Weber, Miklós Dékány, Andrea Nehr-Majoros, Éva Szőke, Zsuzsanna Helyes, László Hazai

**Affiliations:** 1 Department of Organic Chemistry and Technology, Faculty of Chemical Technology and Biotechnology, Budapest University of Technology and Economics, Műegyetem rkp. 3, H-1111 Budapest, Hungary; 2 Spectroscopic Research Department, Gedeon Richter Plc., P.O. Box 27, H-1475 Budapest, Hungary; 3 Department of Pharmacology and Pharmacotherapy, Medical School & Centre for Neuroscience, University of Pécs, H-7624 Pécs, Hungary; 4 National Laboratory for Drug Research and Development, H-1117 Budapest, Hungary; 5 HUN-REN PTE Chronic Pain Research Group, H-7624 Pécs, Hungary

**Keywords:** vindoline, *N*-substituted piperazines, linkers, synthesis, antitumor effect, cell viability

## Abstract

A series of novel vindoline–piperazine conjugates were synthesized by coupling 6 *N*-substituted piperazine pharmacophores at positions 10 and 17 of *Vinca* alkaloid monomer vindoline through different types of linkers. The in vitro antiproliferative activity of the 17 new conjugates was investigated on 60 human tumor cell lines (NCI60). Nine compounds presented significant antiproliferative effects. The most potent derivatives showed low micromolar growth inhibition (*GI*_50_) values against most of the cell lines. Among them, conjugates containing [4-(trifluoromethyl)benzyl]piperazine (**23**) and 1-bis(4-fluorophenyl)methyl piperazine (**25**) in position 17 of vindoline were outstanding. The first one was the most effective on the breast cancer MDA-MB-468 cell line (*GI*_50_ = 1.00 μM), while the second one was the most effective on the non-small cell lung cancer cell line HOP-92 (*GI*_50_ = 1.35 μM). The CellTiter-Glo Luminescent Cell Viability Assay was performed with conjugates **20**, **23**, and **25** on non-tumor Chinese hamster ovary (CHO) cells to determine the selectivity of the conjugates for cancer cells. These compounds exhibited promising selectivity with estimated half-maximal inhibitory concentration (*IC*_50_) values of 2.54 μM, 10.8 μM, and 6.64 μM, respectively. The obtained results may have an impact on the design of novel vindoline-based anticancer compounds.

## 1. Introduction

**Figure 1:**
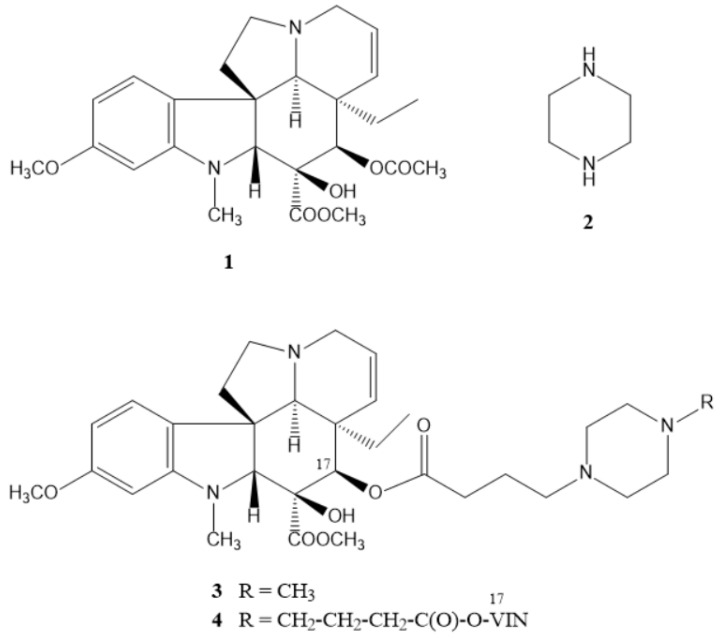
The structure of vindoline (**1**), piperazine (**2**), and previously synthesized piperazine–containing vindoline derivatives (**3**, **4**).

Vinblastine and vincristine, as dimeric indole *Vinca* alkaloids, are well known in pharmaceutical chemistry and have been used for decades in anticancer therapy as tubulin inhibitors. Many of their derivatives were synthesized during extensive research [1,2,3,4,5,6,7,8]. Structurally, they consist of two components, one of which is catharanthine, which is mostly used in dimer formation, and the other is vindoline (**1**) (Figure 1). The latter, in itself, is practically inactive. Many of its derivatives, however, have significant biological effects; namely, they show important antiproliferative activities [9,10,11,12,13].

Piperazine (**2**), as a privileged structure, is found in many drugs [14] and natural compounds [15]. It is especially used in pharmaceutical research due to its excellent physico-chemical characteristics; it provides beneficial pharmacodynamic and pharmacokinetic effects (e.g., solubility, bioavailability, etc.) to the molecule to which it has been coupled [16,17].

Piperazine-conjugated structures can exert many different biological effects, one of the most important is antitumor activity [17,18]. Numerous compounds have been linked to piperazine, such as anticancer molecules, steroids [19], berberine [20], pentacyclic terpenes [21], tylophorine alkaloids [22], harmine derivatives [23], and different heterocycles coupled with sulfonylpiperazines [24]. Recently, in our research group, the **3** *N*-methylpiperazine derivative and the **4** dimer were synthesized through the 17 position of vindoline (**1**) (Figure 1) [25]. The compounds were examined for their anticancer effect against a set of human gynecological tumor cell lines. The dimer (**4**) showed excellent antiproliferative activity (*IC*_50_ = 2.85 μM) on the SiHa cell line, while derivative **3** exerted the best outcomes (*IC*_50_ = 9.36 μM) on the HeLa cell line.

**Figure 2:**
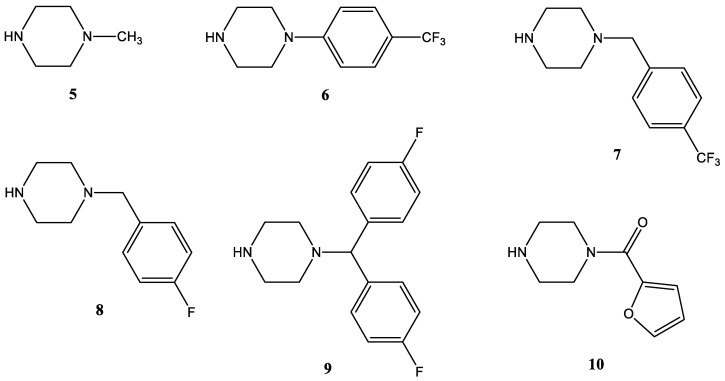
The structure of the selected piperazine pharmacophores (**5**–**10**).

Based on these results and the literature data, we continued our project, during which we coupled various *N*-substituted piperazines to position 17 of vindoline (**1**) via two types of linkers. A more flexible linker was formed using 4-bromobutyric acid, while a slightly more rigid linker was formed with succinic anhydride. To study the structure–activity relationships, the piperazine derivatives were also attached to position 10 of vindoline (**1**); in this case, the linker was built with chloroacetyl chloride. The piperazine pharmacophores planned for coupling were selected based on the work of İbiş et al. [16]. In this project, piperazine–oxazole hybrids were produced, which demonstrated remarkable cytotoxicity on all examined cell lines with *IC*_50_ values in the range of 0.09–11.7 μM. We chose the following six cheap and easily available piperazine derivatives: 1-methylpiperazine (**5**), 1-(4-trifluoromethylphenyl)piperazine (**6**), 1-[4-(trifluoromethyl)benzyl]piperazine (**7**), 1-(4-fluorobenzyl)piperazine (**8**), 1-bis(4-fluorophenyl)methyl piperazine (**9**), and 1-(2-furoyl)piperazine (**10**) (Figure 2). 

In summary, the aim of this work was to combine a *Vinca* alkaloid with one of the most frequent pharmacophore molecules, which is used in a wide range of fields of biological action. It was presumed that even the ineffective vindoline could possess important antitumor activity when connected with piperazines. Moreover, we had another goal, namely, the investigation of the cytotoxic activity of the new compounds not only on cancerous cells but also on non-tumor cells to determine the selectivity.

## 2. Results and Discussion

### 2.1. Chemistry

#### 2.1.1. Preparation of the Linker-Containing Vindoline Derivatives

**Scheme 1:**
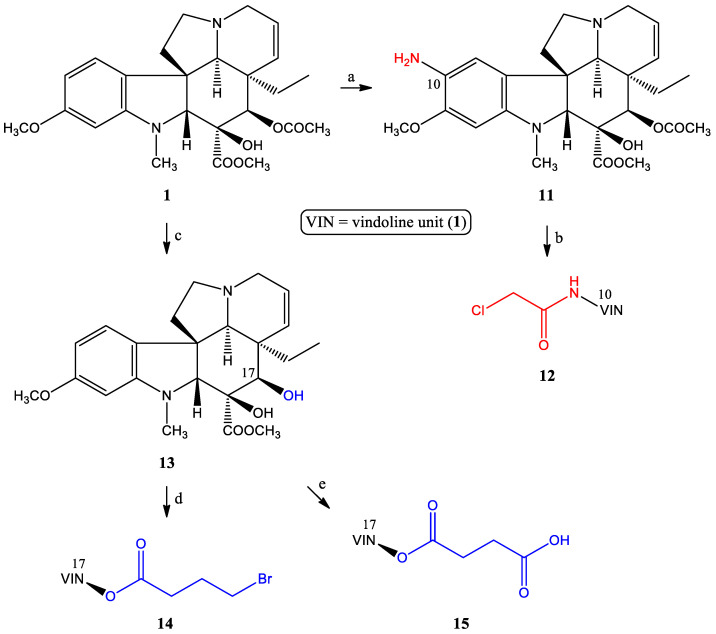
The formation of the linkers at positions 10 (marked in red) and 17 (marked in blue) of vindoline (**1**): (a) (1) THF, 1M HCl, NaNO_2_, 0 °C, 30 min; (2) NaBH_4_, MeOH, Ar, rt, 4 h, 92%; (b) chloroacetyl chloride, abs. DCM, K_2_CO_3_, Ar, rt, 7 h, 64%; (c) Na_2_CO_3_, MeOH, reflux, 24 h, 99%; (d) 4-bromobutyric acid, DCC, DMAP, abs. DCM, rt, 4 h, 75%; (e) succinic anhydride, DMAP, dry toluene, Ar, reflux, 6 h, 83%.

The substitution possibilities for forming linkers on the vindoline skeleton are primarily positions 10 and 17 (Scheme 1). The linkers were built according to known procedures.

The synthesis of 10-chloroacetamidovindoline (**12**) was previously presented by us [26] through the *N*-acylation reaction of 10-aminovindoline (**11**) with chloroacetyl chloride. Although a three-step synthesis of 10-aminovindoline (**11**) was also described by our research group, in this project, a simpler and shorter synthetic procedure was applied [26]. The nitrosation of vindoline (**1**) with sodium nitrite in an acidic medium and the following reduction by sodium borohydride resulted in the desired amino derivative (**11**) in a better overall yield (92%) than the previously described process [27]. 

The other targeted bonding position is the 17-hydroxy group, for which 17-desacetylvindoline (or 17-hydroxyvindoline) (**13**) was prepared with regioselective ester hydrolysis [28]. The 17-*O*-4-bromobutanoyl derivative (**14**) of 17-desacetylvindoline (**13**) was synthesized by a known method described by Keglevich and co-workers [29]. Finally, the 17-*O*-hemisuccinate derivative (**15**) was obtained in the *O*-acylation reaction of 17-desacetylvindoline (**13**) with succinic anhydride according to Passarella et al. [28]. 

#### 2.1.2. Coupling of the Linker-Containing Vindoline Derivatives With Piperazines

**Scheme 2:**
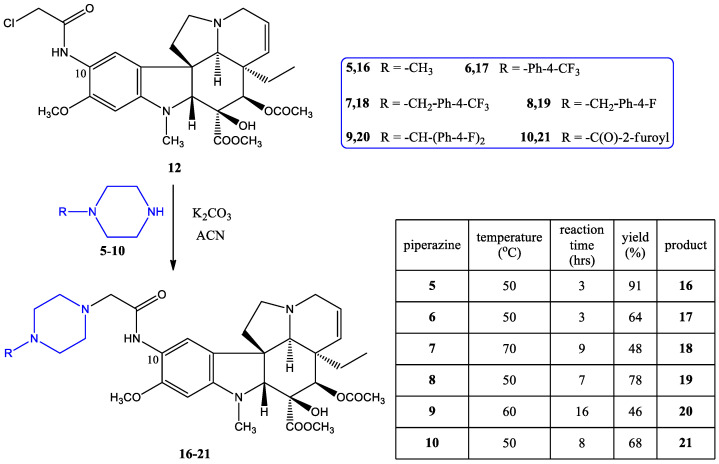
Synthesis of vindoline–piperazine conjugates (**16**–**21**) coupled via a linker formed from chloroacetyl chloride at position 10 of vindoline.

10-Chloroacetamidovindoline (**12**) was reacted with the corresponding piperazine derivative (**5**–**10**) (Figure 2) in acetonitrile solution in the presence of potassium carbonate (Scheme 2) in *N*-alkylation reactions, and this resulted in the **16**–**21** vindoline–piperazine conjugates in medium to excellent yields.

**Scheme 3:**
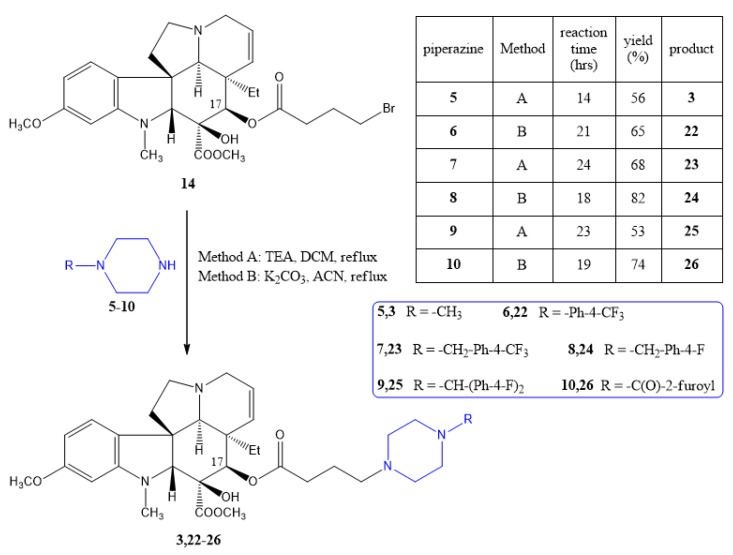
Synthesis of vindoline–piperazine conjugates (**3**, **22**–**26**) coupled via a linker formed from 4-bromobutyric acid at position 17 of vindoline.

Similarly, compounds **3** and **22**–**26** were prepared from the 17-*O*-4-bromobutanoyl derivative (**14**) with the given piperazines (**5**–**10**) (Scheme 3) using two methods, namely, with triethyl amine in dichloromethane solution (Method A) and in acetonitrile in the presence of potassium carbonate (Method B). Method B was used in cases where conversion was low with Method A. As mentioned, compound **3** had already been synthesized before by us [25]; however, now it was obtained with a slightly better yield. The latter was also necessary because we planned to subject it to a more extensive biological screening (NCI60) than before.

**Scheme 4:**
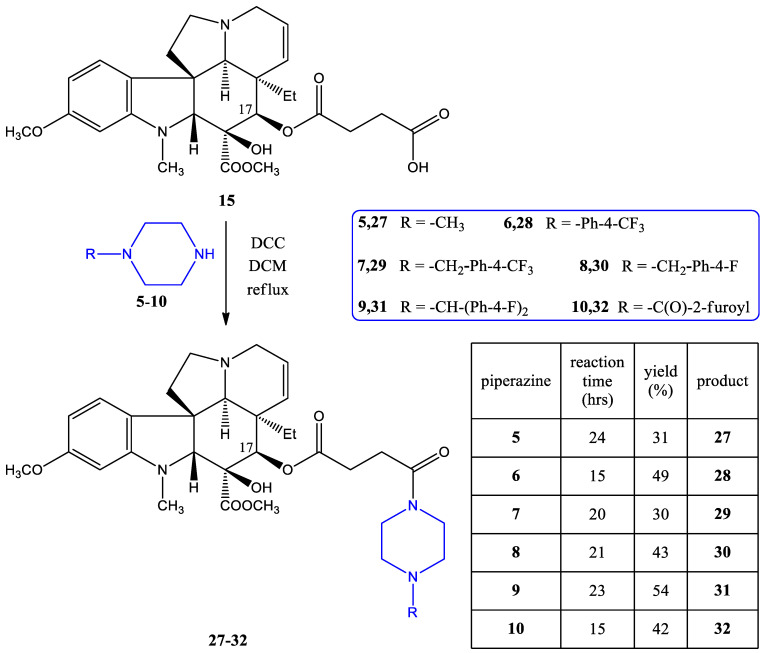
Synthesis of vindoline–piperazine conjugates (**27**–**32**) coupled via a linker formed from succinic anhydride at position 17 of vindoline.

Finally, piperazines **5**–**10** were *O*-acylated with the 17-*O*-hemisuccinate derivative (**15**). Dicyclohexyl carbodiimide (DCC) in dichloromethane solution at the reflux temperature was achieved for the preparation of the **27**–**32** vindoline–piperazine conjugates coupled across position 17 via an amide function (Scheme 4). 

Thus, since the same six piperazine derivatives (**5**–**10**) were coupled to different sites of vindoline, these piperazine conjugates through various types of linkers make a preliminary study of the structure–activity relationship in connection with the anticancer activities possible.

### 2.2. Evaluation of the Biological Activities

#### 2.2.1. NCI60 Screening

The in vitro antiproliferative activities of the 18 synthesized compounds (**3** and **16**–**32**) were examined against 60 human tumor cell lines (NCI60), representing leukemia, non-small cell lung cancer, colon cancer, CNS cancer, melanoma, ovarian cancer, renal cancer, prostate cancer, and breast cancer, at the National Cancer Institute (NCI, USA) according to the given protocols [30,31,32,33,34,35].

The screening results are given in the Supplementary Materials (Tables S1–S3), where the biological activities were determined for the 10^−5^ M concentration. The percentages of growths show the amount of living cancer cells compared to a reference. The negative numbers indicate a significant decrease in the cell number. As expected, a notable antiproliferative effect was not shown by vindoline (**1**), the linker-containing vindoline derivatives (**14** and **15**), and their precursors (**11** and **13**), except 10-chloroacetamidovindoline (**12**), which exerted moderate cytostatic activity.

The experimental data obtained for compounds **16**–**21** containing the piperazine side chain coupled at position 10 of vindoline (**1**) are presented in Table S1. One of them, derivative **17**, in which the piperazine nitrogen atom contains a 4-trifluoromethylphenyl substituent, proved to be highly effective in several types of cancer. In the case of colon cancer, the growth percent rate was found to be −84.40% on the KM12 cell line. For CNS cancer, more than −80% reduction was obtained on SF-539 and SNB-75 brain tumor cell lines. For melanoma, **17** was very effective on almost all cell lines, and the two most outstanding values were −98.17% (on SK-MEL-5) and −95.37% (on LOX-IMVI). In the case of breast cancer for the cell line MDA-MB-231/ATCC, a −86.10% growth rate was obtained. The 1-bis(4-fluorophenyl)methyl piperazine-containing compound (**20**) showed moderate antiproliferative activity on some colon, CNS, and melanoma cell lines. 

Growth percentages of compounds **3** and **22**–**26**, in which the piperazine building blocks are connected to position 17 of vindoline (**1**) via the *N*-alkyl side chain, are summarized in Table S2. Among them, quite outstanding activities were found. Compounds **23** ([4-(trifluoromethyl)benzyl]piperazine derivative) and **25** (1-bis(4-fluorophenyl)methyl piperazine derivative) were highly potent, resulting in more than a −60% growth rate on almost all tested cancer types and cell lines, especially on colon, CNS, melanoma, renal, and breast cancer cells. Derivative **22**, which contains the same 4-trifluoromethylphenyl substituent as **17** also showed important antiproliferative activity on colon cancer (COLO-215, −93.46%), CNS cancer (SF-539, −96.98%), melanoma (SK-MEL-5, −98.54%), and renal cancer (RXF-393, −91.93%).

Data of compounds **27**–**32** coupled with vindoline (**1**) in position 17 through an amide bond are shown in Table S3. The activity of compound **28** containing the 4-trifluoromethylphenyl substituent is also noteworthy in this context. Similar to 1-[4-(trifluoromethyl)benzyl]piperazine-containing derivative **29**, compound **28** is highly effective and rather selective in the cases of colon cancer (COLO-205, −90.33%) and melanoma (SK-MEL-5, −92.46%). The 1-bis(4-fluorophenyl)methyl piperazine-containing compound **31** should also be mentioned; it proved to be effective on several types of cancer, particularly on the leukemia MOLT-4 cell line (−98.81%). 

Since compounds **17**, **20**, **22**–**25, 28**, **29**, and **31** showed significant antiproliferative effects on several cancer cell lines during the one-dose test, they were subjected to a five-dose screening. The 50% growth inhibition (*GI*_50_) and their mean values are given in Table 1. Among them, the ([4-(trifluoromethyl)benzyl]piperazine-containing derivative **23** and the (1-bis(4-fluorophenyl)methyl piperazine-containing compound **25** were the most potent agents. The latter two derivatives resulted in less selectivity but outstanding cytotoxic activity with *GI*_50_ < 2 μM on almost all cell lines. The most significant activity was shown by compound **23** on the MDA-MB-468 cell line of breast cancer (*GI*_50_ = 1.00 μM), while compound **25** had the most significant activity on the HOP-92 cell line of non-small cell lung cancer (*GI*_50_ = 1.35 μM). In addition, compounds **22**, **28**, and **31** exhibited mean *GI*_50_ values below 4 μM. It should also be emphasized that compound **24** had a *GI*_50_ value of 1.00 μM on the renal cancer RXF 393 cell line and that derivative **28** had a *GI*_50_ value of 1.17 μM on the leukemia MOLT-4 cell line. 

In vitro anticancer screening revealed some interesting structure–activity relationships. The results suggest that (i) the substitution of vindoline with piperazine seems to be more beneficial in position 17 compared to position 10; (ii) concerning the linker, *N*-alkyl derivatives are more active than *N*-acyl analogs; and (iii) *N*-(4-trifluoromethylphenyl), *N*-[4-(trifluoromethyl)benzyl] and *N*-bis(4-fluorophenyl)methyl piperazine derivatives proved significantly more efficacious than conjugates containing *N*-methyl, *N*-(4-fluorobenzyl), and *N*-(2-furoyl) groups. 

#### 2.2.2. Effect of Selected Conjugates on Cell Viability of Non-Tumor Chinese Hamster Ovary (CHO) Cell Lines

**Figure 3:**
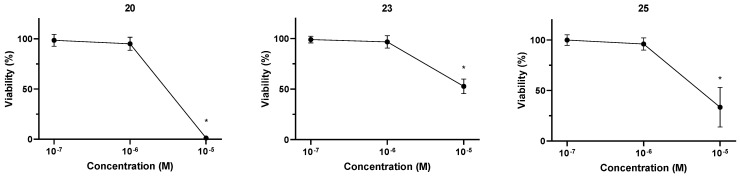
Effect of compounds **20**, **23**, and **25** on the viability of native CHO cells. The percentage values of viable cells after 48 h treatment are presented as mean ± SD of three independent experiments in technical triplicates (n = 9), One-way ANOVA, Dunnett’s post hoc test; * *p* < 0.0001 vs. control).

Three promising conjugates (**20**, **23**, **25**) were selected for testing on the non-tumor CHO cell line in the CellTiter-Glo Luminescent Cell Viability Assay (Promega Corporation, Madison, WI, USA) to reveal their selectivity for cancer cells. Treatment of CHO cells for 48 h with the compounds in the 10^−7^–10^−5^ M concentration range resulted in a concentration-dependent decrease in the luminescent signal proportional to the amount of ATP produced by living cells as an indicator of cell viability. Piperazine conjugate treatment did not affect CHO cell viability in 10^−7^ and 10^−6^ M concentrations, while treatment in 10^−5^ M concentrations resulted in significantly decreased cell viability with values of 1.25 ± 0.77%, 52.76 ± 7.25%, and 33.45 ± 19.62% for compounds **20**, **23**, and **25**, respectively (Figure 3).

Based on these data, *IC*_50_ values of compounds **20**, **23**, and **25** can be roughly estimated to be 2.54 μM, 10.8 μM, and 6.64 μM, respectively; these results show the promising selectivity of the compounds on tumor cells, as a significant inhibitory effect on non-tumor cell viability is exerted at remarkably higher concentrations compared to the investigated tumor cell lines.

## 3. Materials and Methods

### 3.1. General

All chemicals were purchased from Sigma-Aldrich (Budapest, Hungary) and were used as received. Melting points were measured on a VEB Analytik Dresden PHMK-77/1328 apparatus (Dresden, Germany) and were uncorrected. IR spectra were recorded on Zeiss IR 75 and 80 instruments (Thornwood, NY, USA). NMR measurements were performed on a Bruker Avance III HDX 400 MHz NMR spectrometer equipped with a ^31^P–^15^N{1H–^19^F} 5 mm CryoProbe Prodigy BBO probe, a Bruker Avance III HDX 500 MHz NMR spectrometer equipped with a ^1^H{^13^C/^15^N} 5 mm TCI CryoProbe, a Varian VNMRS 600 MHz NMR System NMR spectrometer, and a Bruker Avance III HDX 800 MHz NMR spectrometer equipped with a ^1^H–^19^F{^13^C/^15^N} 5 mm TCI CryoProbe (Bruker Corporation, Billerica, MA, USA). ^1^H And ^13^C chemical shifts are given on the delta scale as parts per million (ppm) relative to tetramethyl silane. One-dimensional ^1^H, and ^13^C spectra and two-dimensional ^1^H–^1^H COSY, ^1^H–^1^H NOESY, ^1^H–^13^C HSQC, and ^1^H–^13^C HMBC spectra were acquired using pulse sequences included in the standard spectrometer software package (Bruker TopSpin 3.5, Bruker Corporation). NMR spectra were processed with Bruker TopSpin 3.5 pl 6 (Bruker Corporation, Billerica, MA, USA) and ACD/Spectrus Processor version 2017.1.3 (Advanced Chemistry Development, Inc., Toronto, ON, Canada). ESI-HRMS and MS-MS analyses were performed on a Thermo Velos Pro Orbitrap Elite (Thermo Fisher Scientific, Bremen, Germany) system. The ionization method was ESI and operated in positive ion mode. The protonated molecular ion peaks were fragmented by CID (collision-induced dissociation) at a normalized collision energy of 35–65%. For the CID experiment, helium was used as the collision gas. The samples were dissolved in methanol. EI-HRMS analyses were performed on a Thermo Q Exactive GC Orbitrap (Thermo Fisher Scientific, Bremen, Germany) system. The ionization method was EI and operated in positive ion mode. The electron energy was 70 eV, and the source temperature was set at 250 °C. Data acquisition and analysis were accomplished with Xcalibur software version 4.0 (Thermo Fisher Scientific). The reactions were followed by analytical thin layer chromatography (TLC) on DC-Alufolien Kieselgel 60 F_254_ (Merck, Budapest, Hungary) plates. Preparative TLC analyses were performed on silica gel 60 PF_254+366_ (Merck) glass plates. Column chromatography was carried out using Silica gel 60 (0.040–0.063 mm) (Merck).

### 3.2. Chemistry

A detailed description of the syntheses can be found in the Supplementary Materials. The NMR and HRMS spectra of the new products (**16**–**32**), as well as the skeleton numberings of compounds used for NMR assignment, are also given in the Supplementary Materials (Figures S1–S131).

### 3.3. Biological Evaluation

#### 3.3.1. NCI60 Screening

A detailed description of the NCI screening procedures [30,31,32,33,34], including the one-dose and five-dose tests, can be found in the Supplementary Materials, on the website of NCI [35], and in our previous work [26].

#### 3.3.2. CellTiter-Glo Luminescent Cell Viability Assay on Non-Tumor CHO Cells

Compounds **20**, **23**, and **25** were dissolved in DMSO in 10 mM stock solutions and stored frozen until use. CHO cells were cultured in complete Dulbecco’s Modified Eagle’s Medium (DMEM, low glucose (1 g/L)) supplemented with 10% fetal bovine serum, 1% Gibco GlutaMAX-I (100×) solution, 1% Gibco MEM non-essential amino acid solution (100×), and 0.1% penicillin–streptomycin mixture. Cells were grown in T-25 size cell culture flasks under standard cell culture conditions (37 °C, 5% CO_2_) and passaged at 70–80% confluency. The assay was performed according to the manufacturer’s protocol, as described previously [36,37,38]. Briefly, CHO cells were seeded to opaque 96-well culture plates at a density of 5000 cells/100 µL complete DMEM/well. The side rows and columns of the plate were filled with sterile phosphate-buffer saline to avoid an edge effect. Following overnight incubation (37 °C, 5% CO_2_), the culture medium was aspirated from the cells and replaced with increasing concentrations of drug solutions (10^−5^, 10^−6^, and 10^−7^ M) diluted from the stock solution in sterile complete DMEM (100 µL/well). Untreated cells served as a control, and complete DMEM served as a luminescent background control. Drug-treated cells were incubated for 48 h (37 °C, 5% CO_2_), and then equilibrated to room temperature in 30 min. At the end of the incubation period, 100 µL of room temperature CellTiter-Glo reagent was added to each well, and the plates were placed on an orbital shaker for 2 min and incubated for additional 10 min at room temperature without shaking. The luminescent signal was measured using an EnSpire AlphaLisa microplate reader (Perkin Elmer, Inc., Waltham, MA, USA). The normalized luminescent values of the treated cells were compared to those of the untreated control. The statistical analysis and calculation of *IC*_50_ values were performed in GraphPad Prism 8.0.1 (GraphPad, La Jolla, CA, USA). Estimated *IC*_50_ values were calculated by using non-linear regression by fitting a sigmoidal dose–response curve to the data points.

## 4. Conclusions

Monomer *Vinca* alkaloid vindoline, which does not show any anticancer effect by itself, was coupled via different positions and linkers with *N*-substituted piperazine derivatives. The latter were chosen because the piperazine skeleton is a well-known pharmacophore, widely used in pharmaceutical research and the field of medicine for different indications. The substituents on the nitrogen atom of piperazines were alkyl, aryl, aralkyl, and heterocyclic groups. The products were prepared using simple, three-step synthetic routes. Among the compounds synthesized, several derivatives presented significant and excellent antiproliferative activity during the in vitro NCI-60 cell line screening, especially the derivatives with *N*-[4-(trifluoromethyl)benzyl] and *N*-bis(4-fluorophenyl)methyl substituents on the piperazine ring. Compound **23** was identified as the most potent antitumor candidate, exhibiting a growth inhibition (*GI*_50_) value of 1.00 μM on the breast cancer MDA-MB-46 cell line. In addition, several other conjugates showed low micromolar *GI*_50_ values against most of the examined cell lines. It is important to emphasize that a particularly valuable result is the selectivity that the most effective compounds showed on non-tumor cells with compound **23** having a half-maximal effective concentration (*EC*_50_) of 10.8 μM. The results obtained in this study are promising for further development; in particular, with the involvement of other piperazines, a more complete SAR could be elaborated. For example, an exciting continuation of the work could be the synthesis and biological evaluation of the *N*-bis[4-(trifluoromethyl)phenyl]methyl analog by hybridizing the piperazine units of the two most effective derivatives (**23** and **25**). Furthermore, the elucidation of the mechanism of action of these types of molecules would also be an interesting scope of study. Finally, we would like to highlight that this study may have a significant impact on the design of new Vinca alkaloid-based antitumor agents.

## Figures and Tables

**Table 1:** *GI*_50_ values (μM) of compounds **17**, **20**, **22–25**, **28**, **29**, and **31** against 60 human cancer cell lines in vitro. Values where *GI*_50_ < 2 μM are highlighted in bold.

Panel	Cell Line	17		20		22		23		24		25		28		29		31	
		GI_50_	Mean	GI_50_	Mean	GI_50_	Mean	GI_50_	Mean	GI_50_	Mean	GI_50_	Mean	GI_50_	Mean	GI_50_	Mean	GI_50_	Mean
Leukemia	CCRF-CEM	3.24	2.75	2.88	2.38	**1.86**	**1.77**	2.04	**1.71**	4.07	3.19	2.04	**1.76**	**1.66**	**1.81**	11.75	9.07	2.11	**1.89**
	HL-60(TB)	2.14		2.19		**1.66**		**1.74**		3.31		**1.55**		**1.45**		12.88		**1.93**	
	K-562	3.02		3.16		**1.41**		**1.70**		2.40		**1.91**		**1.70**		3.80		2.92	
	MOLT-4	2.09		2.14		**1.95**		**1.15**		**1.74**		**1.45**		**1.17**		5.25		**1.35**	
	RPMI-8226	3.09		**1.91**		2.04		2.00		4.17		**1.95**		2.57		6.92		**1.42**	
	SR	2.95		2.00		**1.70**		**1.66**		3.47		**1.70**		2.29		13.80		**1.63**	
Non-small CellLung Cancer	A549/ATCC	4.07	7.29	4.37	15.67	2.95	2.88	**1.78**	**1.80**	10.23	9.83	2.63	**1.85**	2.75	4.23	8.32	10.40	2.67	2.59
	EKVX	6.03		4.07		3.09		**1.70**		12.02		**1.70**		3.63		7.59		n.d.	
	HOP-62	3.72		89.13		3.02		**1.74**		10.23		**1.62**		5.50		14.13		2.55	
	HOP-92	2.57		5.01		**1.95**		**1.51**		**1.66**		**1.35**		2.09		4.27		**1.99**	
	NCI-H226	6.46		6.31		2.88		2.04		10.47		2.04		3.16		9.77		**1.84**	
	NCI-H23	10.72		6.76		4.57		**1.91**		13.18		**1.91**		4.37		12.02		2.70	
	NCI-H322M	15.14		12.02		3.72		2.24		8.32		**1.95**		10.00		14.45		4.99	
	NCI-H460	6.46		3.16		**1.86**		**1.55**		8.51		**1.78**		3.31		10.47		**1.93**	
	NCI-H522	10.47		10.23		**1.91**		**1.70**		13.80		**1.70**		3.24		12.59		2.04	
Colon Cancer	COLO 205	3.24	4.31	2.45	3.40	**1.78**	2.02	**1.58**	**1.64**	**1.95**	6.09	**1.66**	**1.73**	**1.91**	3.16	7.08	9.71	2.82	2.23
	HCC-2998	7.76		3.89		**1.82**		**1.51**		2.19		**1.66**		2.40		12.88		**1.24**	
	HCT-116	3.72		3.09		**1.91**		**1.70**		5.62		**1.78**		3.31		7.76		2.72	
	HCT-15	3.89		3.55		**1.82**		**1.74**		5.13		**1.70**		3.16		10.23		2.45	
	HT29	2.09		2.88		**1.74**		**1.66**		3.80		**1.91**		2.57		6.76		**1.76**	
	KM12	3.72		3.98		2.14		**1.74**		10.47		**1.78**		4.79		11.75		2.36	
	SW-620	5.75		3.98		2.95		**1.55**		13.49		**1.62**		3.98		11.48		2.27	
CNS Cancer	SF-268	3.02	4.01	5.13	4.40	3.63	2.47	**1.35**	**1.57**	5.25	8.08	**1.70**	2.21	4.07	3.69	5.75	11.07	2.98	2.60
	SF-295	3.55		3.47		**1.95**		**1.78**		12.59		**1.70**		3.16		9.33		2.11	
	SF-539	4.68		4.07		**1.78**		**1.58**		10.47		**1.66**		5.25		12.88		2.02	
	SNB-19	5.37		5.89		3.55		**1.58**		11.48		**1.66**		4.37		11.75		3.58	
	SNB-75	**1.45**		**1.82**		**1.66**		**1.45**		**1.12**		5.01		2.00		15.49		2.11	
	U251	6.03		6.03		2.24		**1.66**		7.59		**1.55**		3.31		11.22		2.78	
Melano-ma	LOX IMVI	4.27	5.89	3.24	5.60	**1.70**	**1.98**	**1.78**	**1.62**	5.01	5.92	**1.74**	**1.78**	3.24	3.63	13.80	9.42	**1.80**	2.40
	MALME-3M	14.79		8.13		**1.58**		**1.58**		2.29		**1.51**		5.37		11.48		2.20	
	M14	3.63		4.07		**1.86**		**1.55**		5.50		**1.91**		2.40		8.13		3.55	
	MDA-MB-435	**1.95**		3.89		**1.70**		**1.48**		5.50		**1.66**		3.89		10.47		2.70	
	SK-MEL-2	4.37		3.02		2.19		**1.78**		15.14		2.40		2.75		2.82		**1.62**	
	SK-MEL-28	5.89		12.30		**1.95**		**1.62**		4.68		**1.74**		5.01		11.75		3.26	
	SK-MEL-5	2.40		2.34		**1.70**		**1.35**		**1.66**		**1.55**		3.24		3.89		**1.84**	
	UACC-257	5.50		5.25		**1.82**		**1.74**		2.00		**1.74**		3.89		11.48		2.70	
	UACC-62	10.23		8.13		3.31		**1.66**		11.48		**1.78**		2.88		10.96		**1.92**	
Ovarian Cancer	IGROV1	10.00	6.48	7.94	5.30	2.00	3.05	**1.78**	**1.77**	2.51	10.09	**1.38**	**1.73**	3.31	3.92	10.72	10.59	2.50	2.15
	OVCAR-3	2.88		4.37		2.45		**1.55**		10.72		**1.74**		3.72		6.46		**1.60**	
	OVCAR-4	3.89		3.31		3.80		2.04		5.37		**1.95**		4.27		5.62		**1.62**	
	OVCAR-5	10.72		3.55		4.07		**1.62**		13.80		**1.66**		5.25		14.45		2.18	
	OVCAR-8	8.51		7.59		3.02		**1.82**		14.45		2.09		3.72		12.02		3.18	
	NCI/ADR-RES	6.03		4.47		2.29		**1.58**		11.75		**1.74**		3.31		12.30		**1.95**	
	SK-OV-3	3.31		5.89		3.72		**2.00**		12.02		**1.55**		3.89		12.59		2.02	
Renal Cancer	786–0	6.31	6.01	7.76	5.23	2.57	2.59	**1.48**	**1.63**	6.31	5.58	**1.58**	2.18	5.01	3.67	12.30	9.90	2.47	2.33
	A498	10.72		5.62		3.72		**1.62**		12.88		5.25		5.75		12.30		2.38	
	ACHN	5.62		4.90		3.24		**1.78**		5.89		2.09		3.80		10.72		2.06	
	CAKI-1	2.63		3.16		2.24		**1.48**		2.24		**1.48**		2.00		8.71		3.07	
	RXF 393	5.37		2.69		**1.55**		**1.35**		**1.00**		n.d.		2.29		4.47		n.d.	
	SN12C	7.76		4.90		2.63		2.09		8.32		**1.66**		4.17		11.48		**1.47**	
	TK-10	4.57		7.59		2.88		**1.66**		4.90		**1.51**		4.07		12.30		2.57	
	UO-31	5.13		5.25		**1.86**		**1.58**		3.09		**1.66**		2.29		6.92		2.32	
Prostate Cancer	PC-3	3.31	4.88	4.47	5.39	2.45	2.67	**1.58**	**1.58**	4.17	7.32	**1.51**	**1.65**	2.45	3.85	3.39	7.30	**1.86**	2.27
	DU-145	6.46		6.31		2.88		**1.58**		10.47		**1.78**		5.25		11.22		2.67	
Breast Cancer	MCF7	3.72	6.11	2.95	4.52	2.00	2.51	**1.55**	**1.53**	3.98	4.25	**1.70**	**1.67**	2.95	3.44	5.62	8.72	**1.74**	2.00
	MDA-MB231ATCC	11.22		9.12		2.82		**1.74**		2.82		**1.58**		4.17		14.13		3.02	
	HS 578T	14.79		5.89		2.82		**1.51**		4.07		**1.78**		4.79		10.23		**1.74**	
	BT-549	2.34		2.57		3.02		**1.66**		7.24		**1.78**		3.31		15.14		**1.81**	
	T-47D	2.19		3.55		3.02		**1.74**		5.62		**1.66**		3.55		5.25		2.47	
	MDA-MB-468	2.40		3.02		**1.38**		**1.00**		**1.74**		**1.51**		**1.86**		**1.95**		**1.20**	
Mean		5.53		6.27		2.44		**1.65**		6.84		**1.85**		3.55		9.76		2.29	

## Data Availability

The original contributions presented in the study are included in the article/Supplementary Materials, further inquiries can be directed to the corresponding author.

## Supplementary Materials

The supporting information can be downloaded at https://www.mdpi.com/article/10.3390/ijms25147929/s1.
