# Supplementary material for: Novel Piperazine Derivatives of Vindoline as Anticancer Agents
Source: Int J Mol Sci. 2024 Jul 19;25(14):7929. doi: 10.3390/ijms25147929 (PMC11277489; doi:10.3390/ijms25147929)
Supplement: Supplementary file 1 [file ijms-25-07929-s001.zip › ijms-3081013-supplementary.docx]

*Article*

**Novel Piperazine Derivatives of Vindoline as Anticancer Agents**

**Bernadett Zsoldos ^1^, Nóra Nagy ^1^ Viktória Donkó-Tóth ^1^, Péter Keglevich ^1^, Márton Weber ^2^, Miklós Dékány ^2^, Andrea Nehr-Majoros ^3,4,5^, Éva Szőke ^3,4,5^, Zsuzsanna Helyes ^3,4,5^ and László Hazai ^1,^***

^1^ Department of Organic Chemistry and Technology, Faculty of Chemical Technology and Biotechnology, Budapest University of Technology and Economics, Műegyetem rkp. 3, H-1111 Budapest, Hungary; [zs.detti@gmail.com](mailto:zs.detti@gmail.com) (B.Z.); [nagynora23@gmail.com](mailto:nagynora23@gmail.com) (N.N.); [donkone.toth.viktoria@vbk.bme.hu](mailto:donkone.toth.viktoria@vbk.bme.hu) (V.D.-T.); [keglevich.peter@vbk.bme.hu](mailto:keglevich.peter@vbk.bme.hu) (P.K.)

^2^ Spectroscopic Research Department, Gedeon Richter Plc., P. O. Box 27, H-1475 Budapest 10, Hungary; [weberm@richter.hu](mailto:weberm@richter.hu) (M.W.); [m.dekany@richter.hu](mailto:m.dekany@richter.hu) (M.D.)

^3^ Department of Pharmacology and Pharmacotherapy, Medical School & Centre for Neuroscience, University of Pécs, Pécs, Hungary; [majoros.andii@gmail.com](mailto:majoros.andii@gmail.com) (A.N.-M.); [eva.szoke@aok.pte.hu](mailto:eva.szoke@aok.pte.hu) (É.S.); [helyes.zsuzsanna@pte.hu](mailto:helyes.zsuzsanna@pte.hu) (Z.H.)

^4^ National Laboratory for Drug Research and Development, Budapest, Hungary

^5^ HUN-REN PTE Chronic Pain Research Group, Pécs, Hungary

***** Correspondence: [hazai.laszlo@vbk.bme.hu](mailto:hazai.laszlo@vbk.bme.hu) (L.H.)

**Supplementary materials**

**S.1. Chemistry**

**S.1.1. Synthesis of the linker-containing vindoline derivatives (12, 14, and 15) and their precursors (11 and 13)**

**S.1.1.1. Preparation of 10-aminovindoline (11); modified procedure**

1500 mg (3.29 mmol) of vindoline was dissolved in 75 mL of THF, then 15 mL (15.0 mmol, 4.6 eq.) of 1*M* hydrochloric acid and 600 mg (8.70 mmol, 2.6 eq.) of NaNO_2_ were added at 0 ^o^C. After stirring the mixture for 30 min the solvents were removed under reduced pressure. The residue was then taken up in 50 mL DCM and was washed with 50 mL distilled water after the pH was adjusted to 9 with 25% NH_4_OH solution. After phase separation, the water phase was extracted with 2 x 50 mL DCM, and the combined organic layer was dried over Na_2_SO_4_ and concentrated *in vacuo*. The crude product was dissolved in MeOH (100 mL) and under argon sodium borohydride (400 mg, 10.6 mmol, 3.2 eq.) was added in portions over 5 min. Based on TLC reaction monitoring, an additional 500 mg (13.2 mmol, 4.0 eq.) of NaBH_4_ was required in 3 parts. The reaction mixture was stirred for 4 h and then evaporated to dryness. The residue was dissolved in 50 mL DCM and was washed with 100 mL distilled water. After phase separation, the water phase was extracted with 2 x 50 mL DCM, and the combined organic layer was washed with 2 x 50 mL distilled water. The organic phase was dried over Na_2_SO_4_ and evaporated under diminished pressure, resulting in 1423 mg (92%) of brown crystalline product. ^1^H NMR, m.p., and *R_f_* data were in good agreement with the literature [26,27]. The obtained product was used in the next step (3.2.2.) without purification.

**S.1.1.2. Preparation of 10-chloroacetamidovindoline (12)**

To a solution of crude 10-aminovindoline (**11**) (880 mg, 1.87 mmol) in dry DCM (20 mL) anhydrous potassium carbonate (285 mg, 2.06 mmol, 1.1 eq.) was added under Ar atmosphere. The mixture was cooled to 0 °C, and a solution of chloroacetyl chloride (0.16 mL, 2.06 mmol, 1.1 eq.) in DCM (0.5 mL) was added dropwise. The mixture was allowed to reach room temperature. After 7 h of stirring the reaction mixture was filtered and the filtrate was diluted with DCM (60 mL), then washed with 5 % NaHCO_3_ solution (40 mL). The separated aqueous phase was extracted with DCM (2 x 20 mL). The combined organic layer was dried over Na_2_SO_4_ and concentrated *in vacuo*. The resulting crude product was subjected to preparative TLC (DCM : MeOH = 20 : 1) afforded the title compound as a white crystalline (654 mg, 64%). ^1^H NMR, m.p., and *R_f_* data were in good agreement with the literature [26].

**S.1.1.3. Preparation of 17-desacetylvindoline (13)**

To a solution of vindoline (**1**) (2000 mg, 4.38 mmol) in dry MeOH (40 mL) anhydrous sodium carbonate (1390 mg, 13.1 mmol, 3.0 eq.) was added. The reaction mixture was refluxed with vigorous stirring. After 24 h, TLC analysis showed that the starting material was consumed. Then, the mixture was evaporated to dryness *in vacuo* and the residue was dissolved in DCM (30 mL). Finally, the sodium carbonate was filtered out of the mixture and the filtrate was concentrated under reduced pressure, resulting in 1797 mg (99%) of white crystalline product. ^1^H NMR, m.p., and *R_f_* data were in good agreement with the literature [25,28].

**S.1.1.4. Preparation of 17-(*O*-4-bromobutanoyl)vindoline (14)**

17-Desacetylvindoline (**13**) (810 mg, 1.95 mmol) and 4-bromobutyric acid (342 mg, 2.05 mmol, 1.05 eq.) were dissolved in 15 mL of abs. DCM followed by a dropwise addition of 444 mg (2.15 mmol, 1,1 eq.) of DCC and 24 mg (0.195 mmol, 0.1 eq.) of 4-dimethylaminopyridine (DMAP) dissolved in another 15 mL of abs. DCM. The reaction mixture was stirred for 4 h and then filtered. The filtrate was evaporated under reduced pressure. After preparative TLC (dichloromethane: methanol = 20: 1) 831 mg (75%) of a pale yellow crystalline product was obtained. ^1^H NMR, m.p., and *R_f_* data were in good agreement with the literature [25,29].

**S.1.1.5. Preparation of** **17-(*O*-3-carboxypropanoyl)vindoline (15)**

To a solution of 17-desacetylvindoline (**13**) (1000 mg, 2.41 mmol) in dry toluene (70 mL) succinic anhydride (362 mg, 3.62 mmol, 1.5 eq.) and 4-dimethylaminopyridine (DMAP) (148 mg, 1.21 mmol, 0.5 eq.) was added. The reaction mixture was refluxed for 6 h and then. filtered The filtrate was evaporated under diminished pressure, and the residue was purified by column chromatography (SiO_2_, dichloromethane: methanol 20 : 1 🡪 10 : 1) to yield **15** (1028 mg, 83%) as a pale yellow crystalline product. ^1^H NMR, m.p., and *R_f_* data were in good agreement with the literature [25,28].

**S.1.2. Synthesis of vindoline - piperazine conjugates (16-21, 3, 22-26, and 27-32)**

**S.1.2.1. General procedure for the synthesis of products (16-21)**

10-Chloroacetamidovindoline (**12**) (100 mg, 0.18 mmol), the appropriate piperazine (**5**-**10**) (0.27 mmol, 1.5 eq.), and anhydrous potassium carbonate (25 mg, 0.18 mmol, 1.0 eq.) were dissolved in MeCN (5 mL). The reaction mixture was stirred at the temperature and time presented in Scheme 1. Then, the mixture was filtered, and the filtrate was evaporated under reduced pressure. The pale yellow crystalline products (**16**-**21**) were isolated after preparative TLC (DCM : MeOH = 15 : 1).

Product **16**

84 mg (91%). M.p.: 128-129 °C. TLC (DCM : MeOH = 15 : 1); *R_f_* = 0.40. IR (KBr) 3676, 3304, 2935, 2876, 2793, 1522, 1222 cm^-1^. ^1^H NMR (499.9 MHz; DMSO-*d*_6_) *δ* (ppm): 0.43 (3H; t; *J* = 7.3 Hz; H_3_-18); 0.95 (1H; dq; *J* = 14.2, 7.3 Hz; H_x_-19); 1.47 (1H; dq; *J* = 14.2, 7.4 Hz; H_y_-19); 1.94 (3H; s; C(17)-OCOCH_3_); 2.15-2.24 (5H; m; H_2_-6, N(4’)-CH_3_); 2.29-2.46 (4H; br s; H_2_-3’, H_2_-5’); 2.48-2.64 (9H; m; N(1)-CH_3_. H_x_-5. H-21. H_2_-2’. H_2_-6’); 2.80-2.86 (1H; m; H_x_-3); 3.02 (1H; d; *J* = 16.3 Hz; H_x_-2”); 3.08 (1H; d; *J* = 16.3 Hz; H_y_-2”); 3.26-3.34 (1H; m; H_y_-5); 3.42 (1H; br dd; *J* = 16.9, 4.6 Hz; H_y_-3); 3.52 (1H; s; H-2); 3.66 (3H; s; C(16)-COOCH_3_); 3.86 (3H; s; C(11)-OCH_3_); 5.09 (1H; br d; *J* = 10.1 Hz; H-15); 5.19 (1H; s; H-17); 5.83 (1H; ddd; *J* = 10.2, 4.8, 1.3 Hz; H-14); 6.45 (1H; s; H-12); 7.91 (1H; s; Hz; H-9); 8.76 (1H; s; C(16)-OH); 9.43 (1H; s; C(10)-NH-C(1”)). ^13^C NMR (125.7 MHz; DMSO-*d*_6_) *δ* (ppm): 7.4 (C-18); 20.7 (C(17)-OCOCH_3_); 30.4 (C-19); 38.8 (N(1)-CH_3_); 42.4 (C-20); 43.6 (C-6); 45.7 (N(4’)-CH_3_); 50.3 (C-3); 51.1 (C-5); 51.6 (C(16)-COOCH_3_); 52.5 (C-7); 52.6 (C-2’, C-6’); 54.9 (C-3’, C-5’); 56.0 (C(11)-OCH_3_); 61.1 (C-2”); 66.1 (C-21); 75.8 (C-17); 78.7 (C-16); 82.7 (C-2); 94.0 (C-12); 114.0 (C-9); 119.4 (C-10); 123.5 (C-8); 124.4 (C-14); 129.8 (C-15); 148.6 (C-13); 149.5 (C-11); 166.9 (C-1”); 170.0 (C(17)-OCOCH_3_); 171.5 (C(16)-COOCH_3_). HRMS: M+H=612.33944 (delta = 0.4 ppm; C_32_H_46_O_7_N_5_).

**Figure S1.** The skeleton numbering of compound **16** used for NMR assignment.


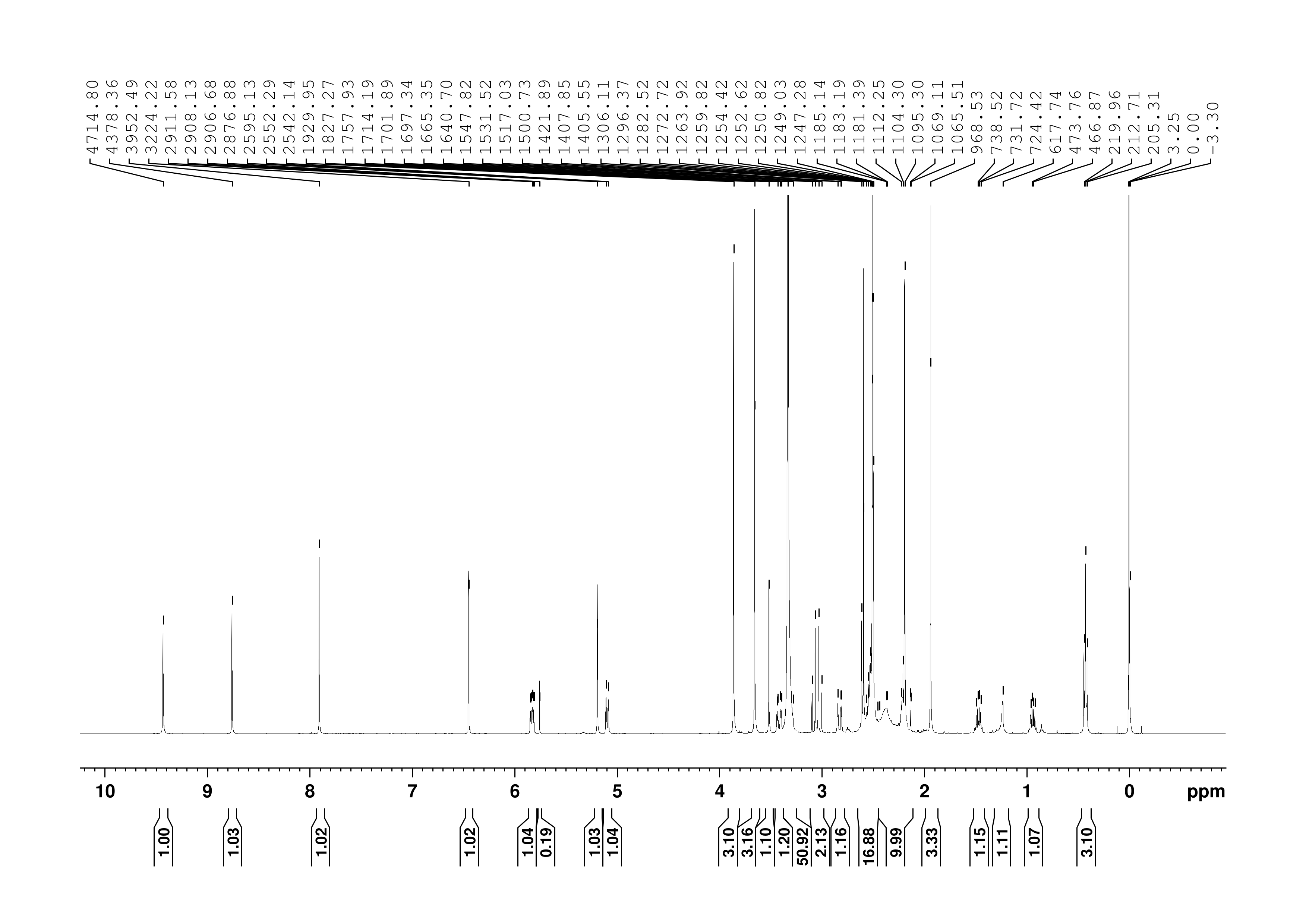


**Figure S2.** ^1^H NMR spectrum of compound **16**.


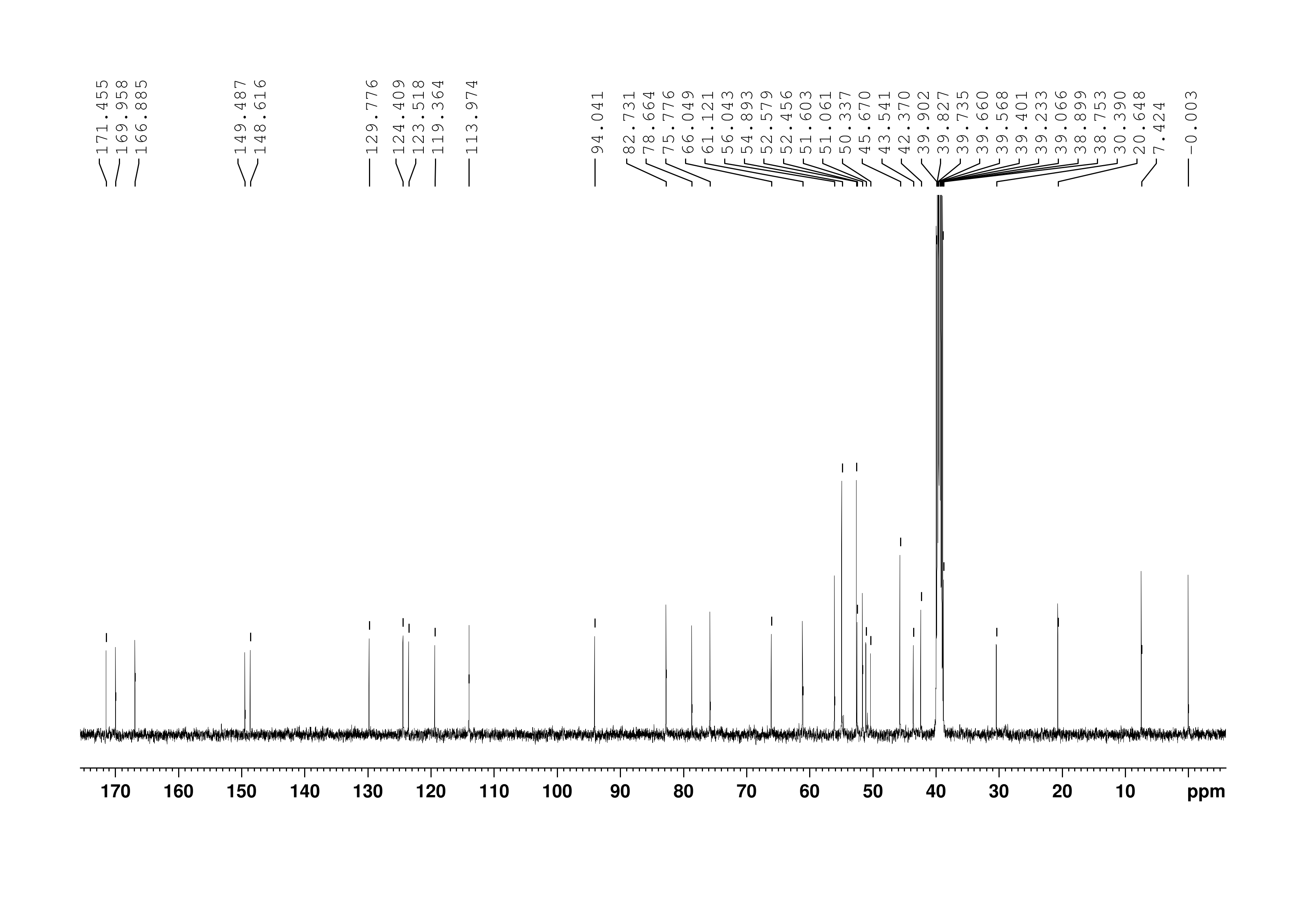


**Figure S3.** ^13^C NMR spectrum of compound **16**.


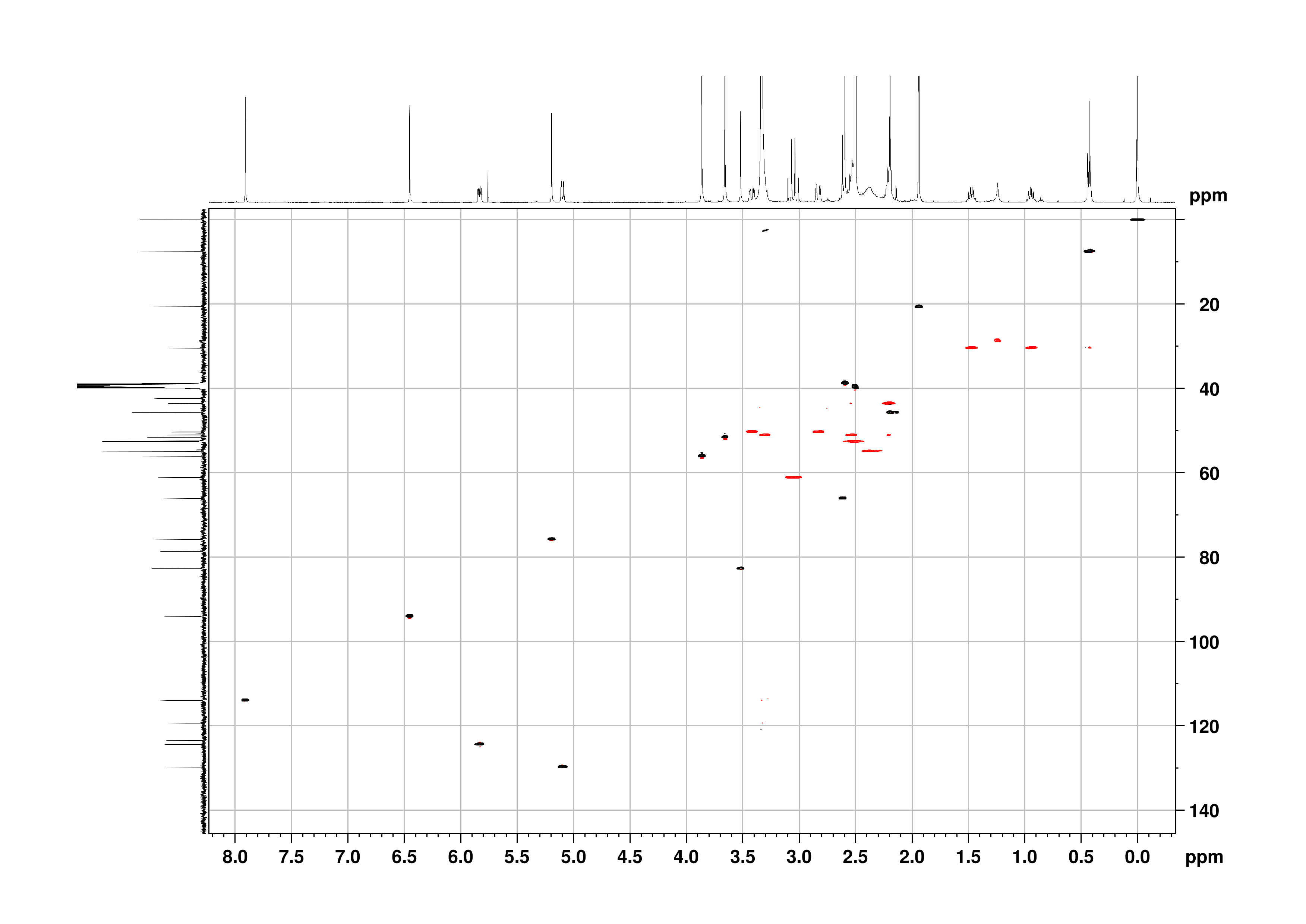


**Figure S4.** HSQC spectrum of compound **16**.


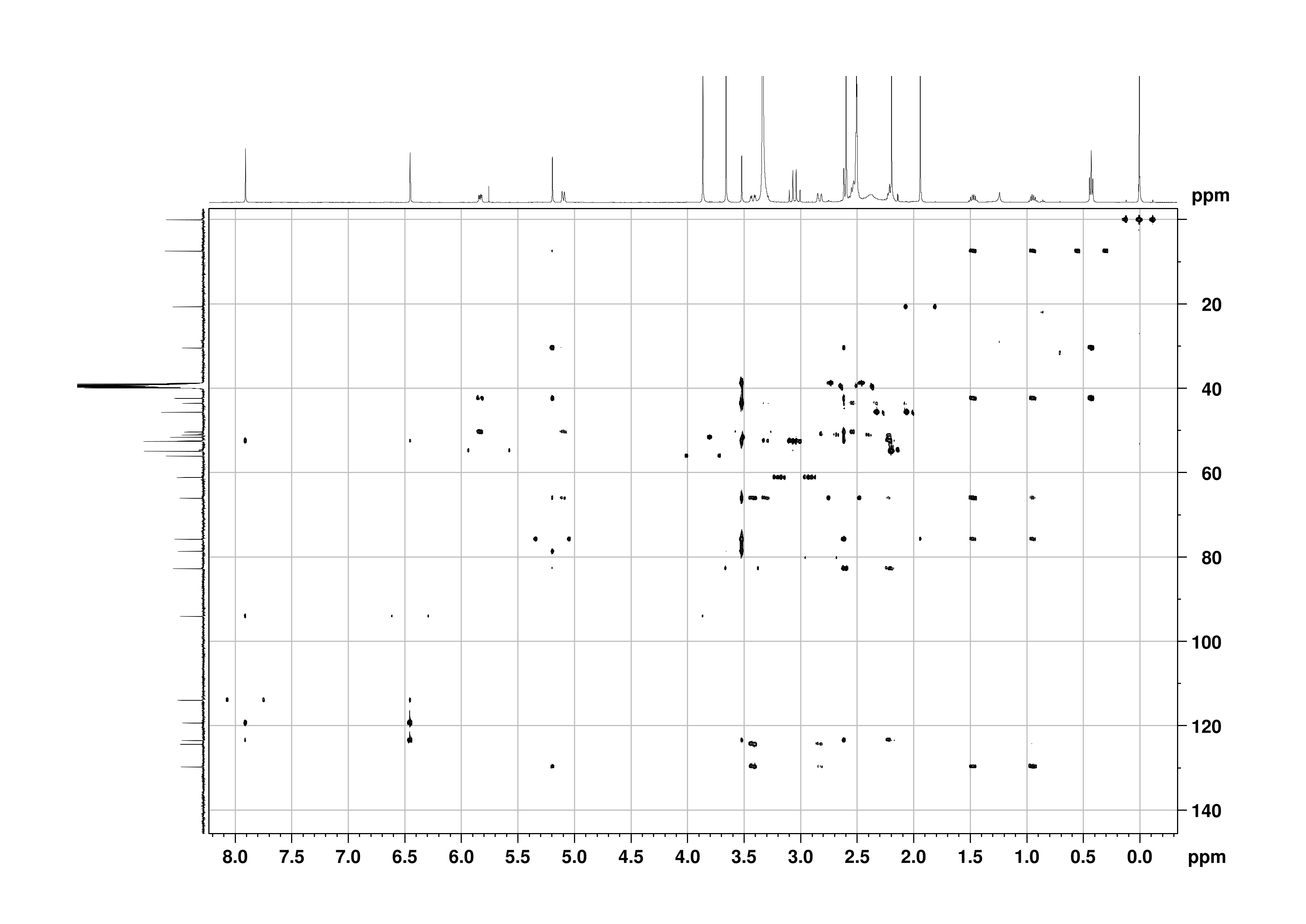


**Figure S5.** ^1^H-^13^C HMBC spectrum of compound **16**.


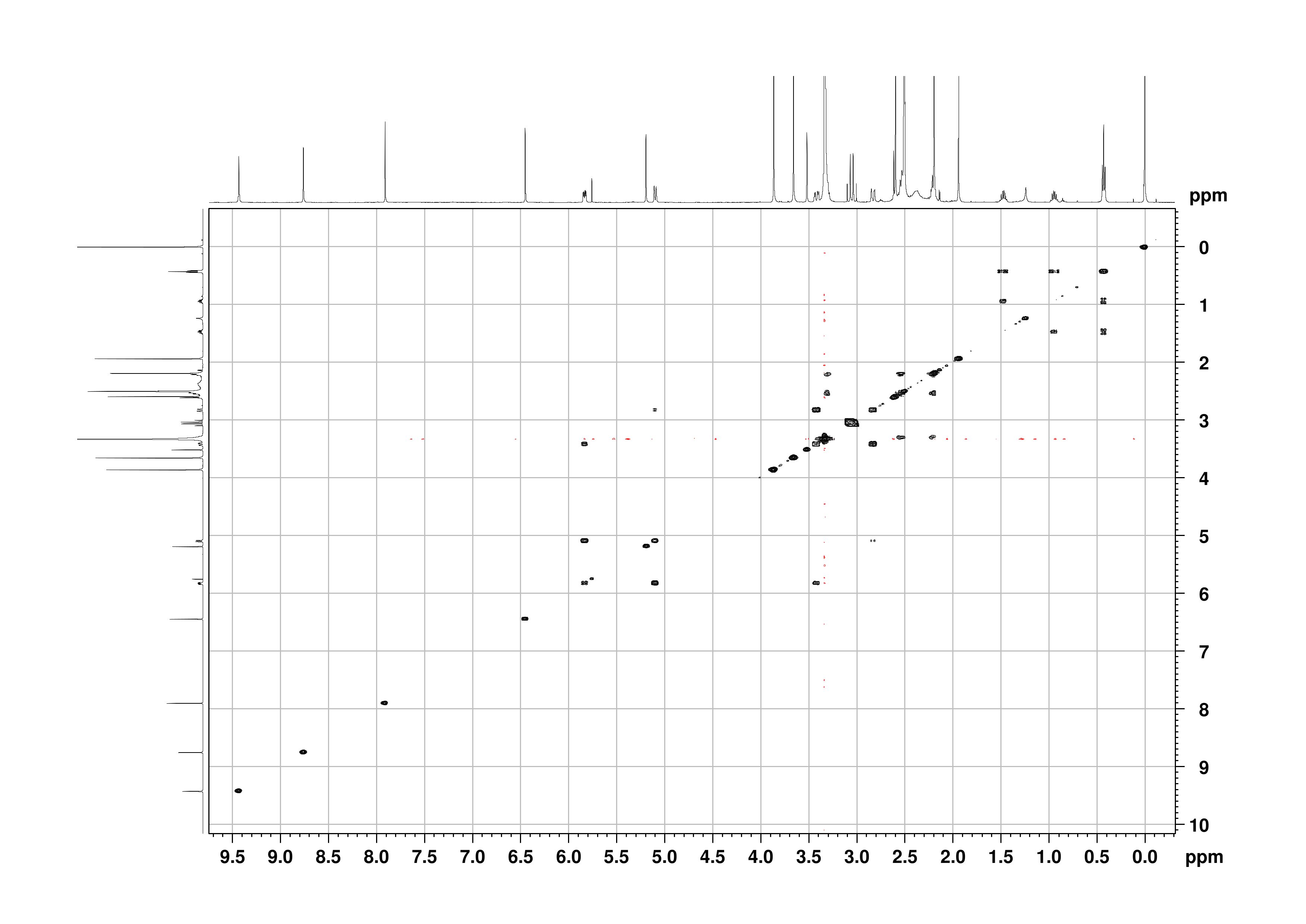


**Figure S6.** COSY spectrum of compound **16**.


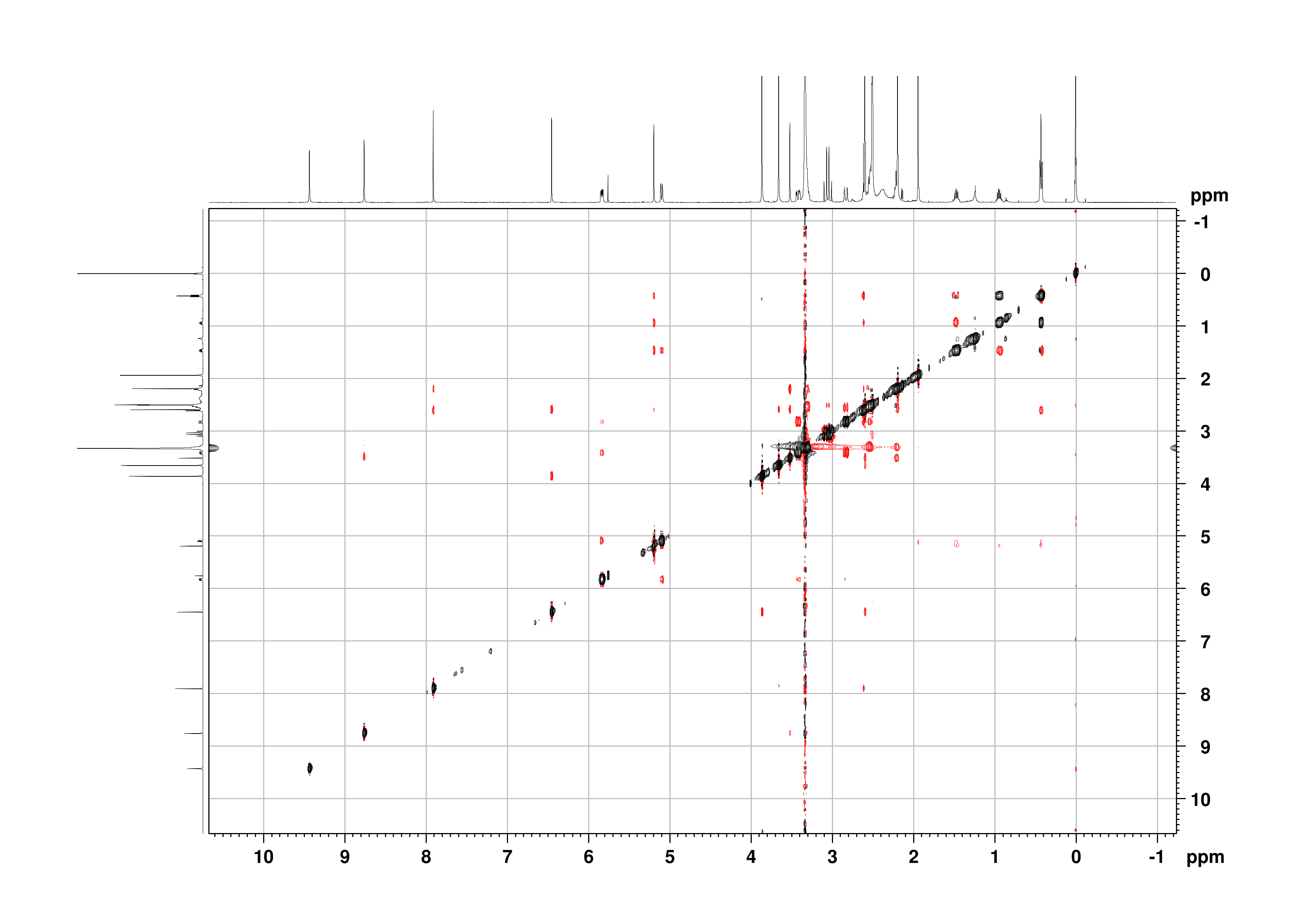


**Figure S7.** ROESY spectrum of compound **16**.

**Figure S8.** HRMS spectrum of compound **16**.

Product **17**

86 mg (64%). M.p.: 144-145 °C. TLC (DCM : MeOH = 15 : 1); *R_f_* = 0.54. IR (KBr) 3317, 2823, 1740, 1522, 1329, 1235 cm^-1^. ^1^H NMR (499.9 MHz; DMSO-*d*_6_) *δ* (ppm): 0.44 (3H; t; *J* = 7.3 Hz; H_3_-18); 0.95 (1H; dq; *J* = 14.2, 7.3 Hz; H_x_-19); 1.48 (1H; dq; *J* = 14.2, 7.4 Hz; H_y_-19); 1.94 (3H; s; C(17)-OCOCH_3_); 2.17-2.27 (2H; m; H_2_-6); 2.51-2.58 (1H; m; H_x_-5); 2.59 (3H; s; N(1)-CH_3_); 2.63 (1H; s; H-21); 2.65-2.70 (4H; m; H_2_-2’, H_2_-6’); 2.83 (1H; br d. *J* = 16.4 Hz; H_x_-3); 3.12 (1H; d; *J* = 16.2 Hz; H_x_-2”); 3.18 (1H; d; *J* = 16.2 Hz; H_y_-2”); 3.27-3.38 (5H; m; H_y_-5, H_2_-3’, H_2_-5’); 3.42 (1H; br dd; *J* = 16.9, 4.5 Hz; H_y_-3); 3.52 (1H; s; H-2); 3.66 (3H; s; C(16)-COOCH_3_); 3.83 (3H; s; C(11)-OCH_3_); 5.10 (1H; br d; *J* = 10.1 Hz; H-15); 5.20 (1H; s; H-17); 5.84 (1H; ddd; *J* = 10.2, 4.8, 1.4 Hz; H-14); 6.45 (1H; s; H-12); 7.10 (2H; d; *J* = 8.8 Hz; H-8’, H-12’); 7.52 (2H; d; *J* = 8.8 Hz; H-9’, H-11’); 7.89 (1H; s; Hz; H-9); 8.76 (1H; s; C(16)-OH); 9.40 (1H; s; C(10)-NH-C(1”)). ^13^C NMR (125.7 MHz; DMSO-*d*_6_) *δ* (ppm): 7.4 (C-18); 20.7 (C(17)-OCOCH_3_); 30.4 (C-19); 38.7 (N(1)-CH_3_); 42.4 (C-20); 43.6 (C-6); 47.2 (C-3’, C-5’); 50.3 (C-3); 51.1 (C-5); 51.6 (C(16)-COOCH_3_); 52.3 (C-2’, C-6’); 52.5 (C-7); 56.0 (C(11)-OCH_3_); 61.1 (C-2”); 66.1 (C-21); 75.8 (C-17); 78.7 (C-16); 82.7 (C-2); 94.0 (C-12); 114.2 (C-8’. C-12’); 114.4 (C-9); 117.9 (d; *J* = 32.0 Hz; C-10’); 119.2 (C-10); 123.5 (C-8); 124.4 (C-14); 124.9 (qu; *J* = 270.4 Hz; C-13’); 126.1 (qu; *J* = 3.6 Hz; C-9’. C-11’); 129.8 (C-15); 148.8 (C-13); 149.7 (C-11); 153.1 (C-7’); 166.7 (C-1”); 170.0 (C(17)-OCOCH_3_); 171.5 (C(16)-COOCH_3_). HRMS: M+H=742.34198 (delta = -0.3 ppm; C_38_H_47_O_7_N_5_F_3_).

**Figure S9.** The skeleton numbering of compound **17** used for NMR assignment.


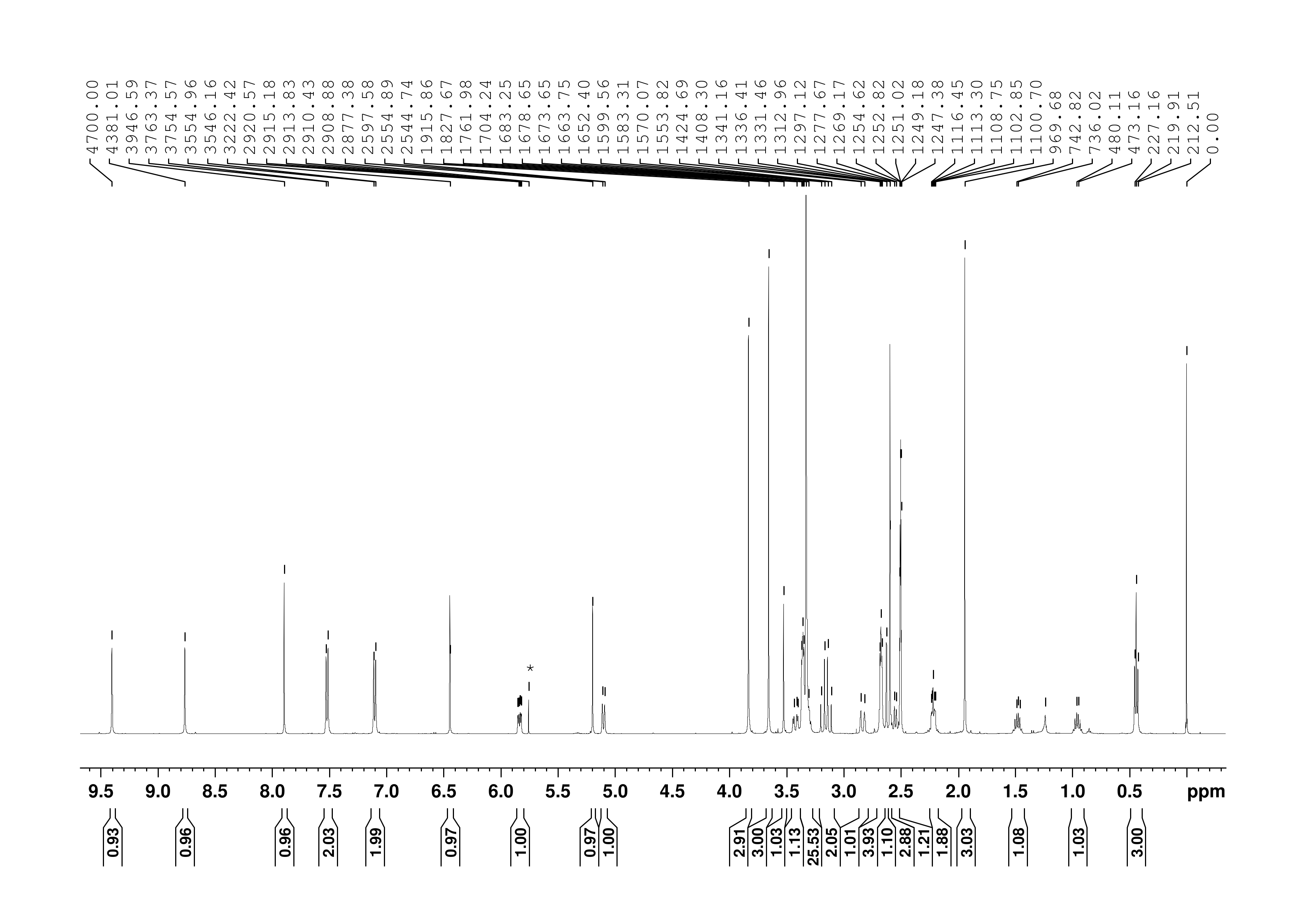


**Figure S10.** ^1^H NMR spectrum of compound **17.**

**
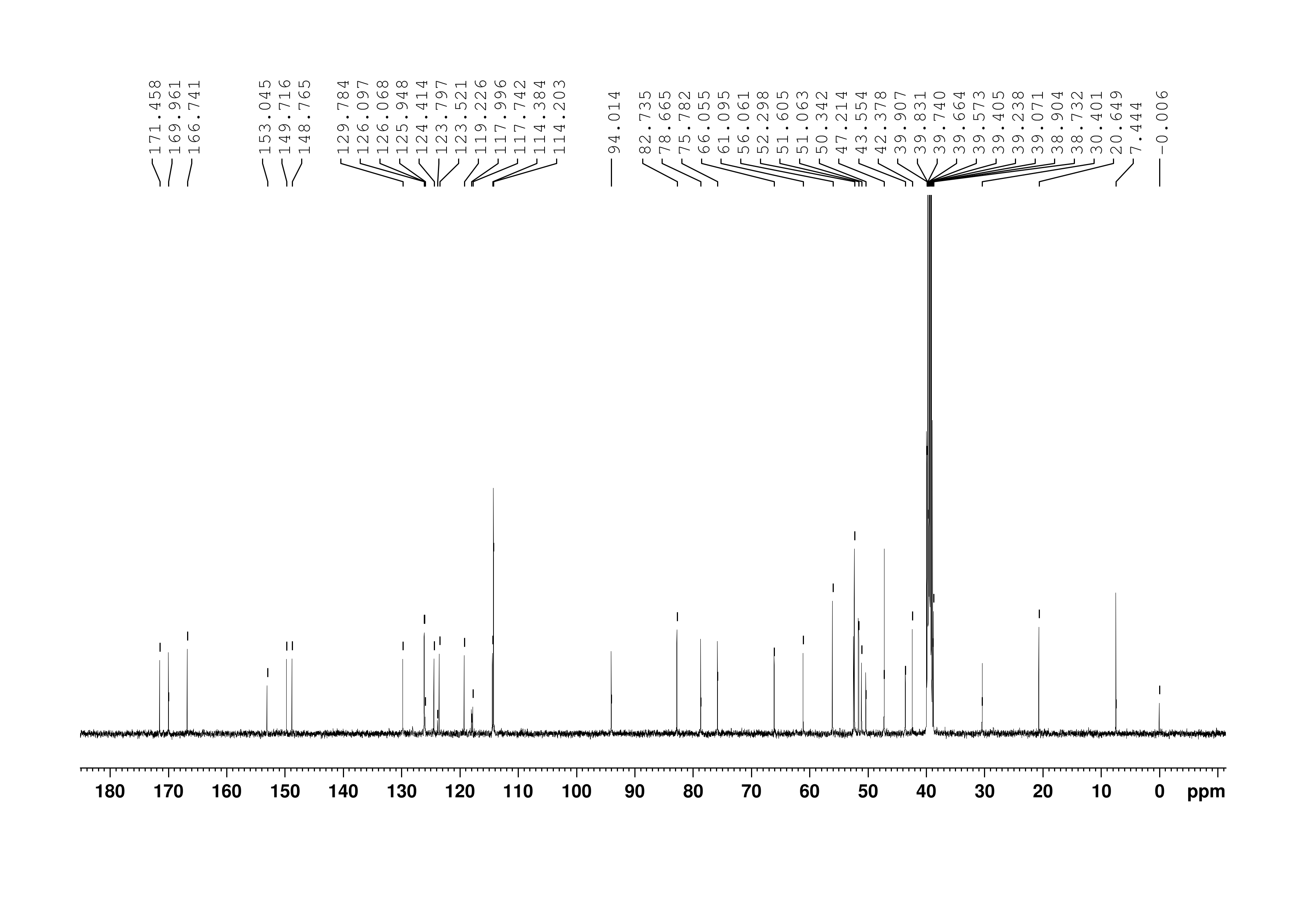
**

**Figure S11.** ^13^C NMR spectrum of compound **17.**

**
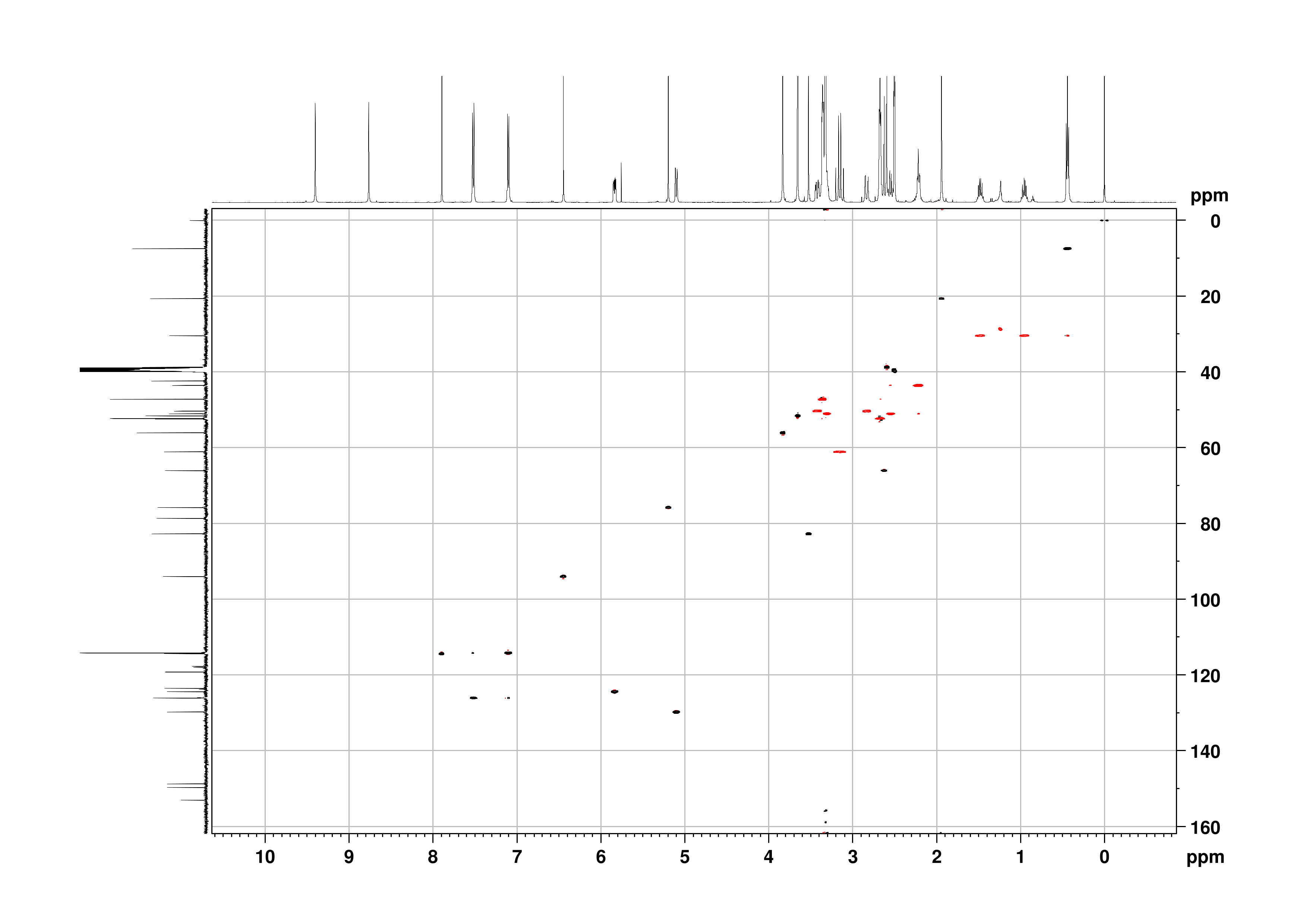
**

**Figure S12.** HSQC spectrum of compound **17.**


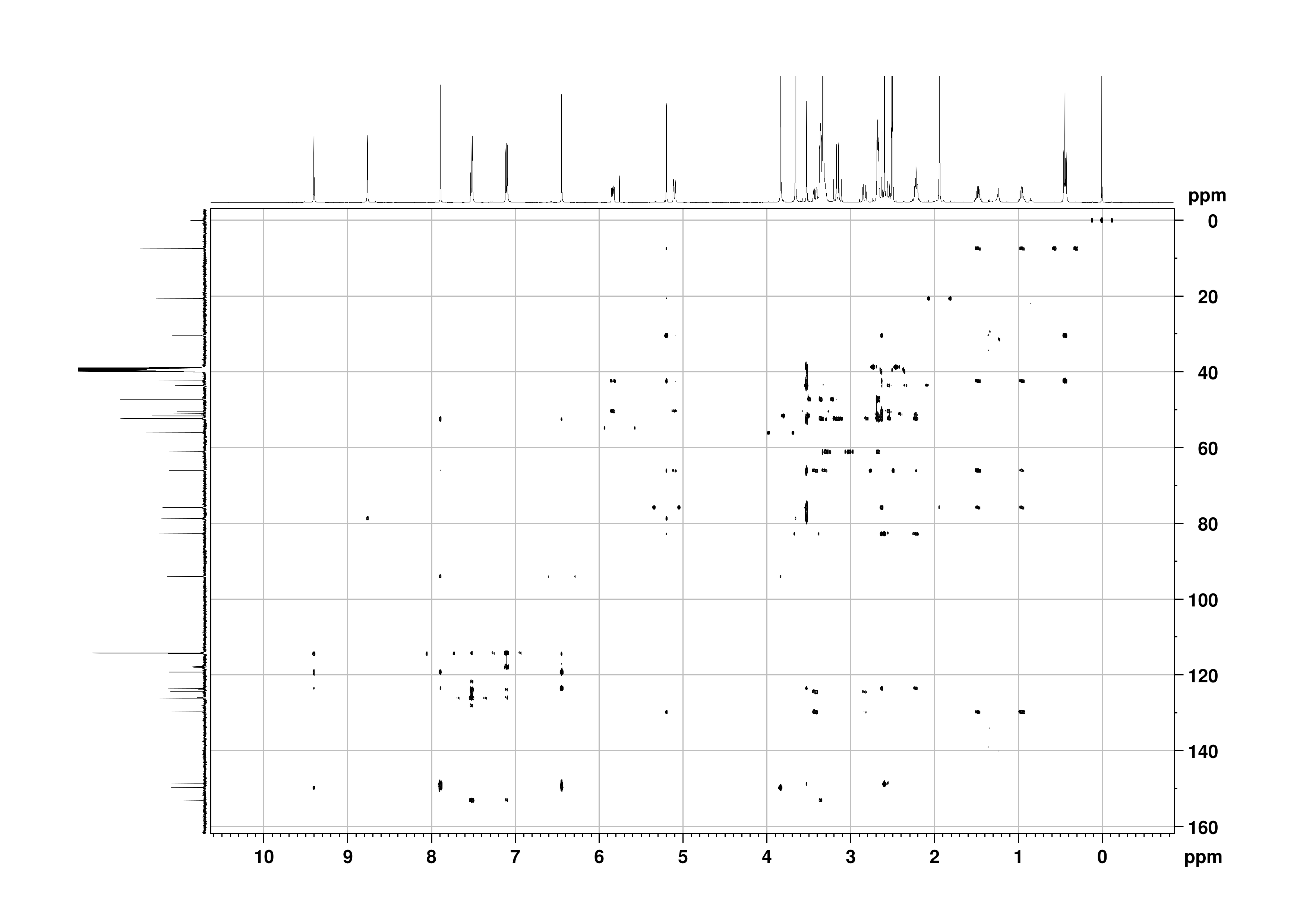


**Figure S13.** ^1^H-^13^C HMBC spectrum of compound **17.**

**
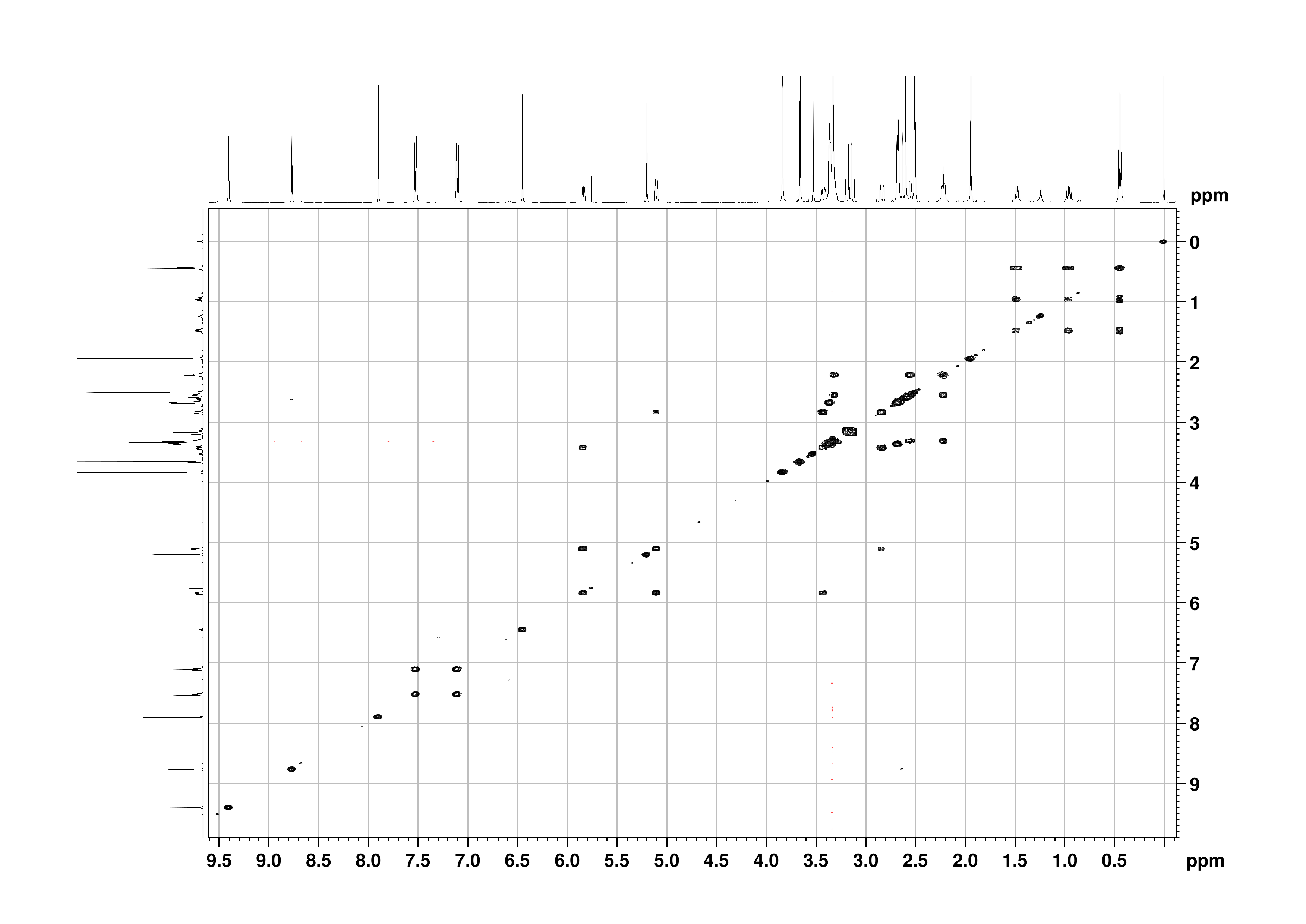
**

**Figure S14.** COSY spectrum of compound **17.**

**
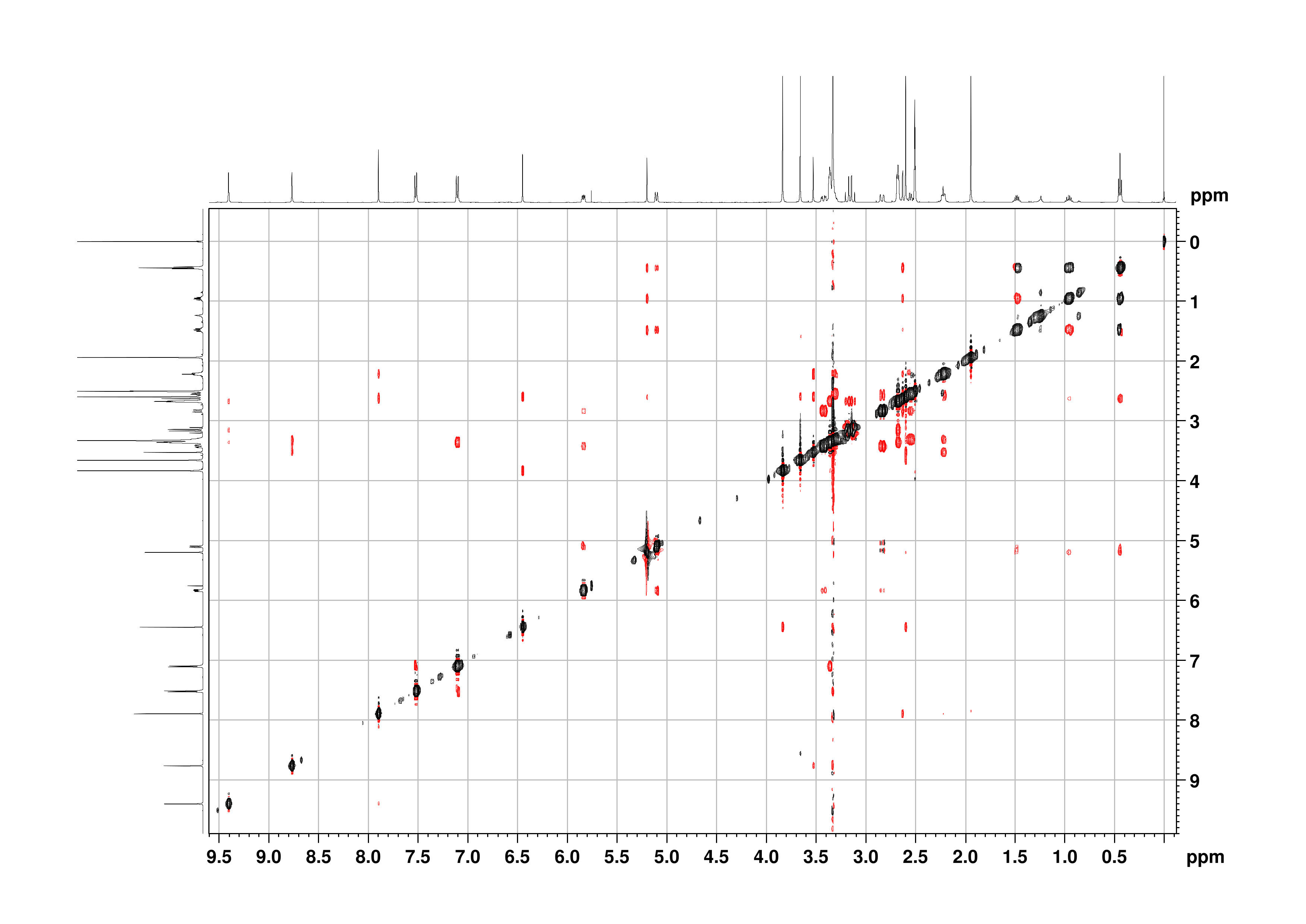
**

**Figure S15.** ROESY spectrum of compound **17.**

**Figure S16.** HRMS spectrum of compound **17.**

Product **18**

66 mg (48%). M.p.: 118-119 °C. TLC (DCM : MeOH = 15 : 1); *R_f_* = 0.39. IR (KBr) 3313, 2816, 1740, 1523, 1324, 1222 cm^-1^. ^1^H NMR (499.9 MHz; DMSO-*d*_6_) *δ* (ppm): 0.42 (3H; t; *J* = 7.3 Hz; H_3_-18); 0.94 (1H; dq; *J* = 14.1, 7.3 Hz; H_x_-19); 1.47 (1H; dq; *J* = 14.2, 7.4 Hz; H_y_-19); 1.94 (3H; s; C(17)-OCOCH_3_); 2.15-2.25 (2H; m; H_2_-6); 2.35-2.63 (13H; m; N(1)-CH_3,_ H_x_-5, H-21, H_2_-2’, H_2_-3’, H_2_-5’, H_2_-6’); 2.82 (1H; br d, *J* = 16.4 Hz; H_x_-3); 3.03 (1H; d; *J* = 16.3 Hz; H_x_-2”); 3.09 (1H; d; *J* = 16.3 Hz; H_y_-2”); 3.25-3.33 (1H; m; H_y_-5); 3.41 (1H; br dd; *J* = 16.8, 4.5 Hz; H_y_-3); 3.51 (1H; s; H-2); 3.62 (2H; s; H_2_-7’); 3.66 (3H; s; C(16)-COOCH_3_); 3.82 (3H; s; C(11)-OCH_3_); 5.09 (1H; br d; *J* = 10.2 Hz; H-15); 5.19 (1H; s; H-17); 5.83 (1H; ddd; *J* = 10.2, 4.9, 1.5 Hz; H-14); 6.45 (1H; s; H-12); 7.55 (2H; d; *J* = 8.0 Hz; H-9’, H-13’); 7.70 (2H; d; *J* = 8.1 Hz; H-10’, H-12’); 7.90 (1H; s; Hz; H-9); 8.75 (1H; s; C(16)-OH); 9.43 (1H; s; C(10)-NH-C(1”)). ^13^C NMR (125.7 MHz; DMSO-*d*_6_) *δ* (ppm): 7.4 (C-18); 20.7 (C(17)-OCOCH_3_); 30.4 (C-19); 38.7 (N(1)-CH_3_); 42.4 (C-20); 43.5 (C-6); 50.3 (C-3); 51.1 (C-5); 51.6 (C(16)-COOCH_3_); 52.5 (C-7); 52.7 (C-2’, C-3’, C-5’, C-6’); 56.0 (C(11)-OCH_3_); 61.1 (C-7’, C-2”); 66.1 (C-21); 75.8 (C-17); 78.7 (C-16); 82.7 (C-2); 94.0 (C-12); 113.9 (C-9); 119.4 (C-10); 123.5 (C-8); 124.4 (C-14); 124.9 (qu; *J* = 3.6 Hz; C-10’, C-12’); 127.5 (qu; *J* = 31.5 Hz; C-11’); 129.4 (C-9’, C-13’); 129.8 (C-15); 142.9 (C-8’); 148.6 (C-13); 149.5 (C-11); 166.8 (C-1”); 170.0 (C(17)-OCOCH_3_); 171.5 (C(16)-COOCH_3_). HRMS: M+H=756.35784 (delta = -0.03 ppm; C_39_H_49_O_7_N_5_F_3_).

**Figure S17.** The skeleton numbering of compound **18** used for NMR assignment.


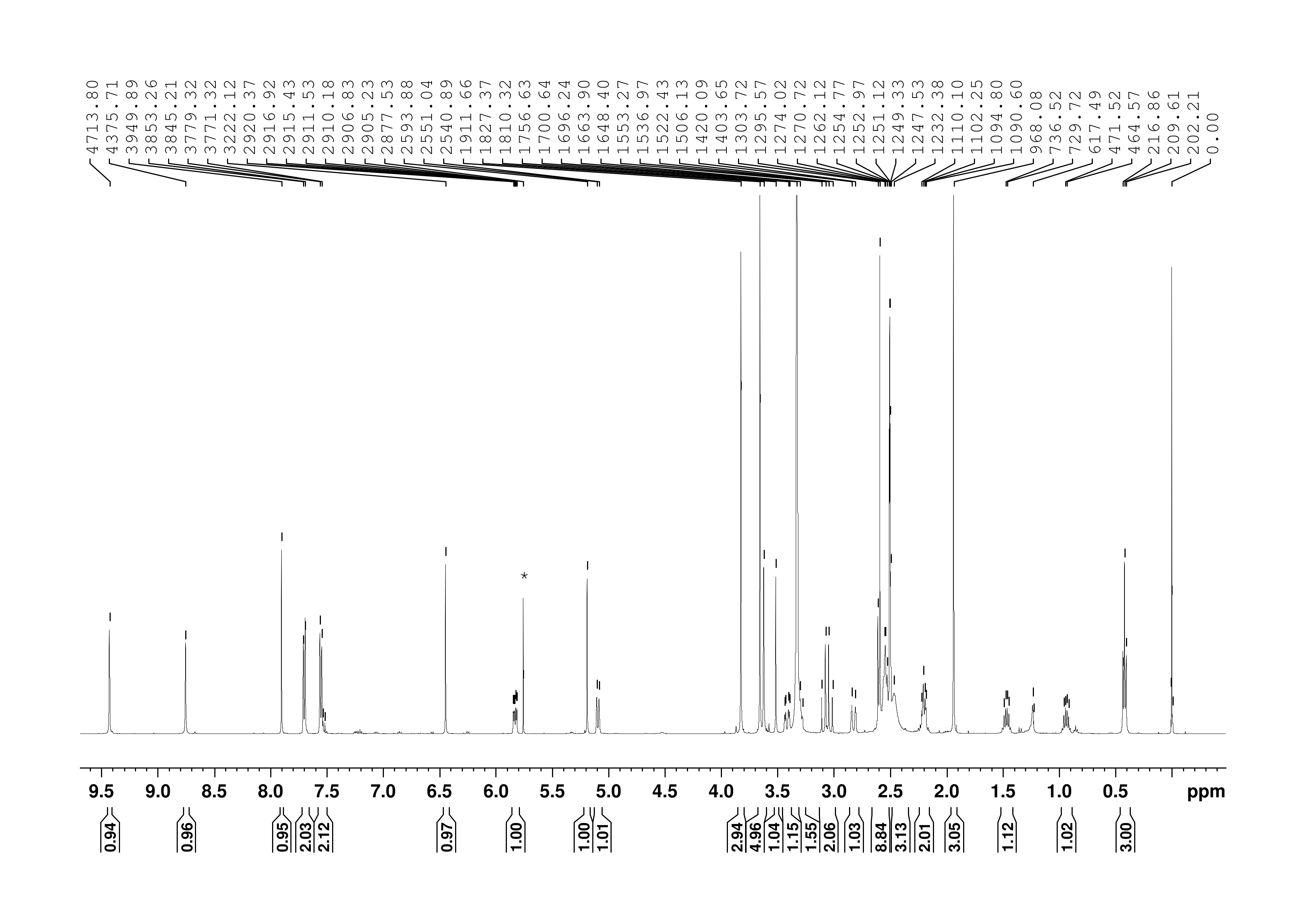


**Figure S18.** ^1^H NMR spectrum of compound **18.**

**
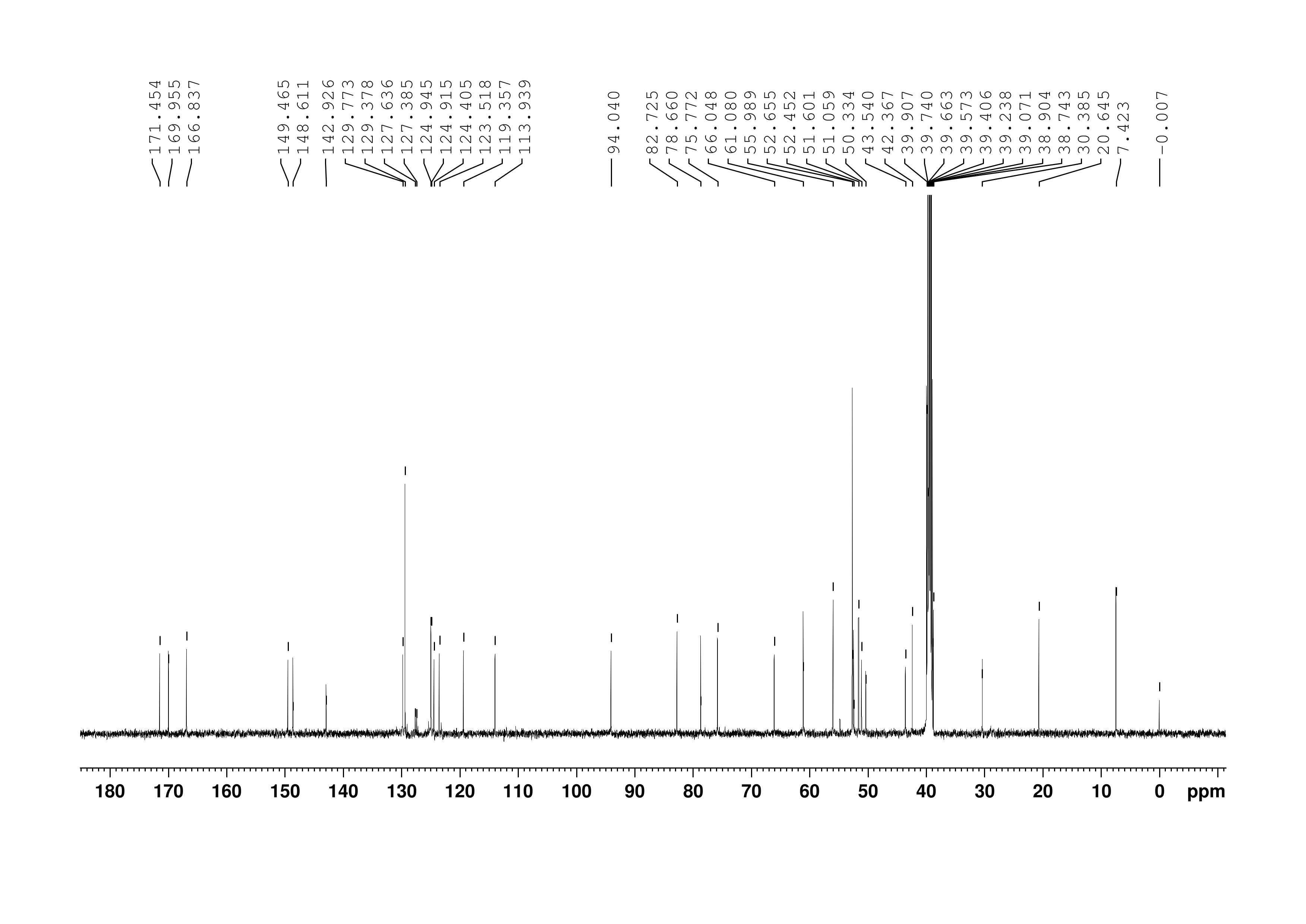
**

**Figure S19.** ^13^C NMR spectrum of compound **18.**

**
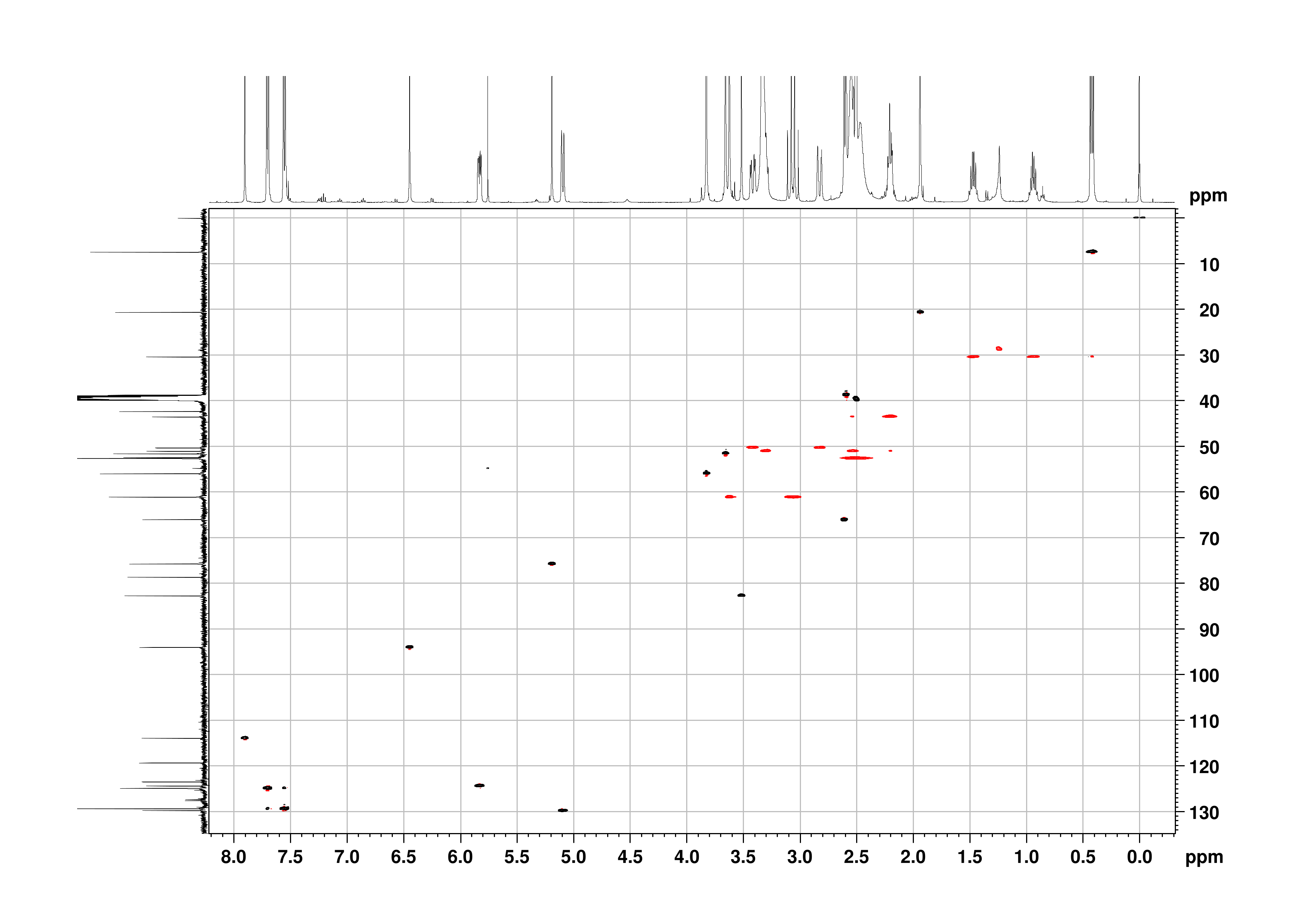
**

**Figure S20.** HSQC spectrum of compound **18.**

**
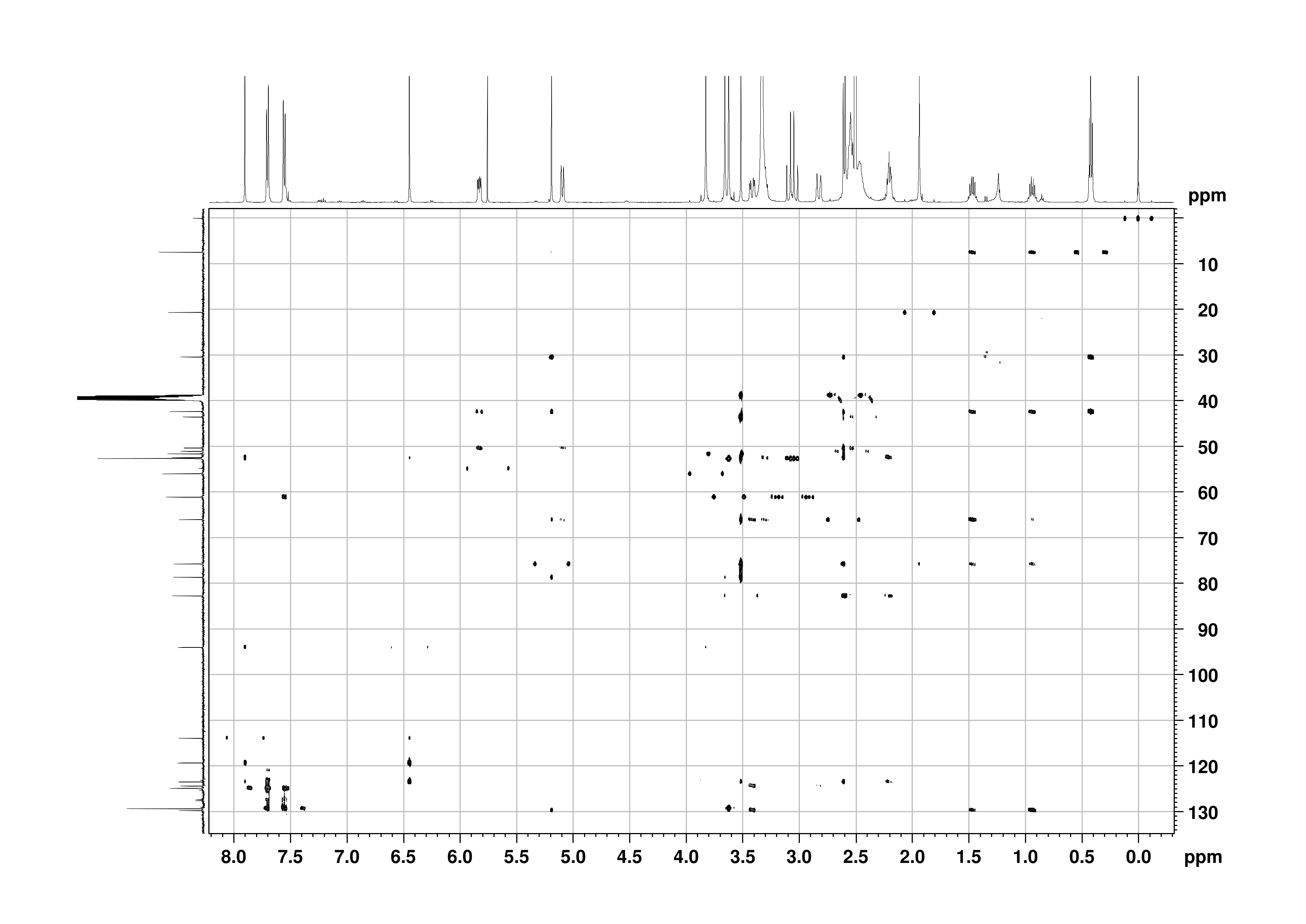
**

**Figure S21.** ^1^H-^13^C HMBC spectrum of compound **18.**

**
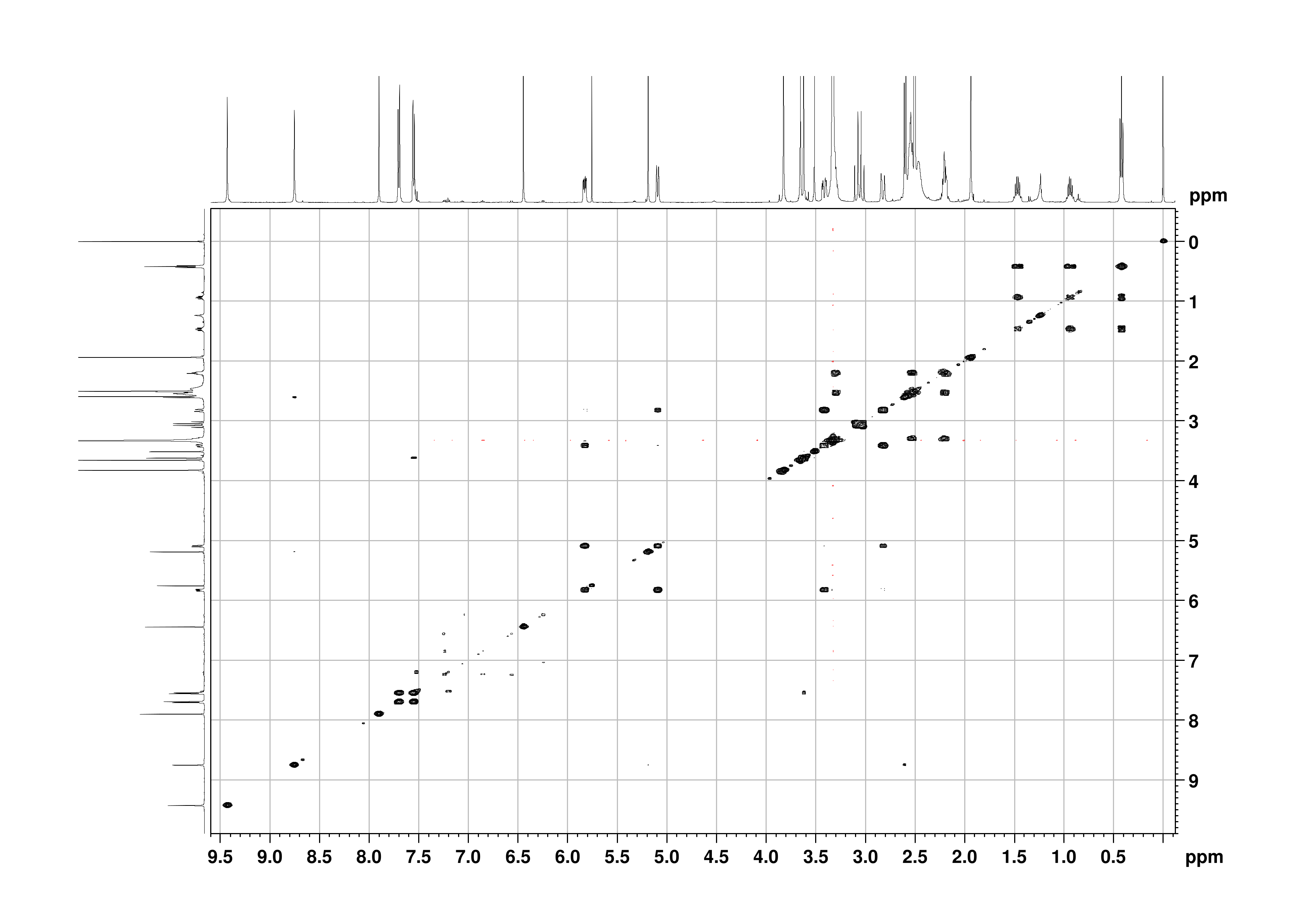
**

**Figure S22.** COSY spectrum of compound **18.**

**
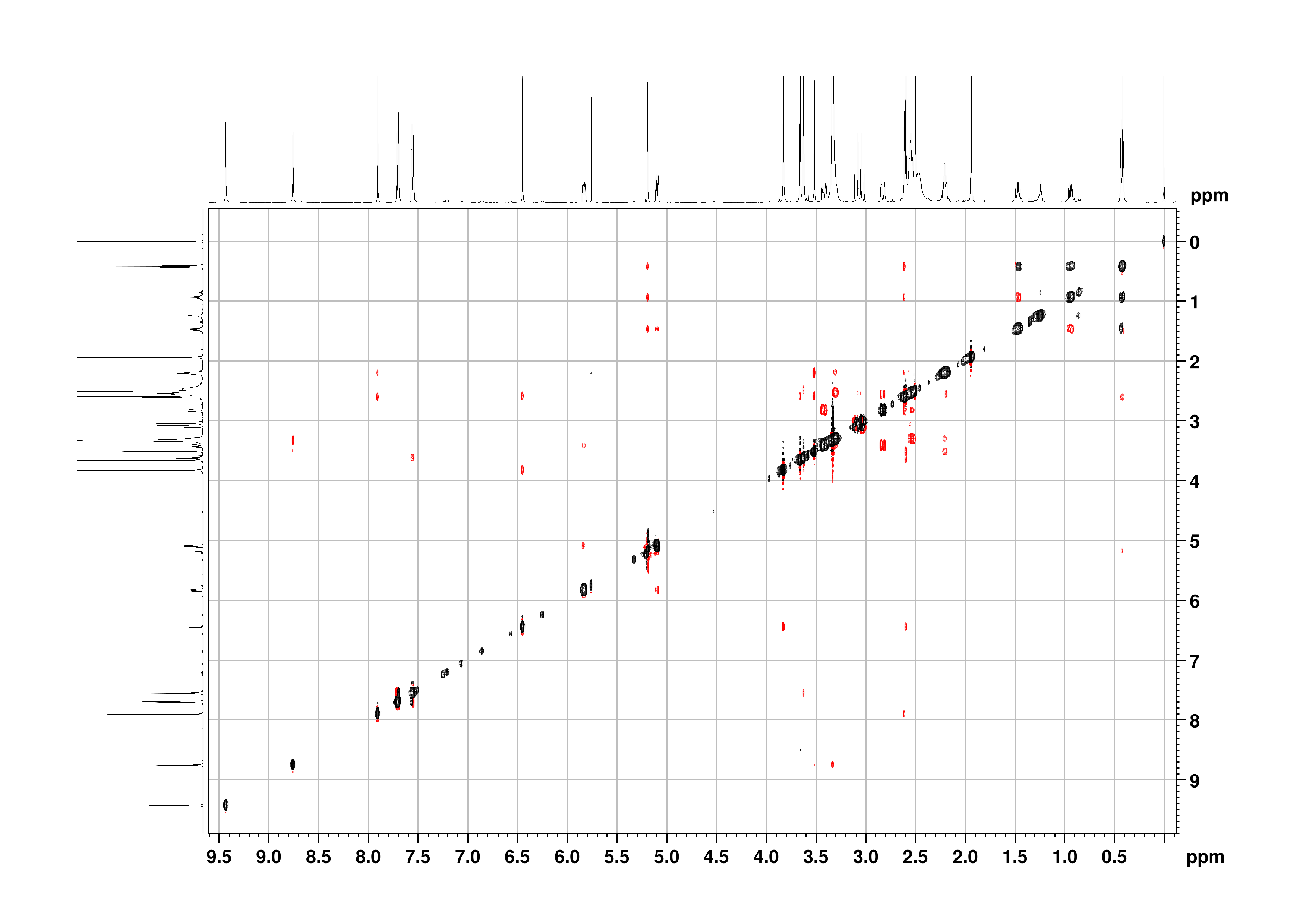
**

**Figure S23.** ROESY spectrum of compound **18.**

**Figure S24.** HRMS spectrum of compound **18.**

Product **19**

100 mg (78%). M.p.: 116-117 °C. TLC (DCM : MeOH = 15 : 1); *R_f_* = 0.36. IR (KBr) 2935, 2876, 2810, 1793, 1508, 1220 cm^-1^. ^1^H NMR (499.9 MHz; DMSO-*d*_6_) *δ* (ppm): 0.42 (3H; t; *J* = 7.3 Hz; H_3_-18); 0.95 (1H; dq; *J* = 14.2, 7.3 Hz; H_x_-19); 1.47 (1H; dq; *J* = 14.2, 7.4 Hz; H_y_-19); 1.94 (3H; s; C(17)-OCOCH_3_); 2.15-2.24 (2H; m; H_2_-6); 2.35-2.63 (13H; m; N(1)-CH_3_, H_x_-5, H-21, H_2_-2’, H_2_-3’, H_2_-5’, H_2_-6’); 2.82 (1H; br d, *J* = 16.4 Hz; H_x_-3); 3.02 (1H; d; *J* = 16.3 Hz; H_x_-2”); 3.08 (1H; d; *J* = 16.3 Hz; H_y_-2”); 3.26-3.34 (1H; m; H_y_-5); 3.41 (1H; br dd; *J* = 16.8, 4.5 Hz; H_y_-3); 3.50 (2H; s; H_2_-7’); 3.51 (1H; s; H-2); 3.66 (3H; s; C(16)-COOCH_3_); 3.83 (3H; s; C(11)-OCH_3_); 5.09 (1H; br d; *J* = 10.2 Hz; H-15); 5.19 (1H; s; H-17); 5.83 (1H; ddd; *J* = 10.2, 4.8, 1.4 Hz; H-14); 6.44 (1H; s; H-12); 7.11-7.18 (2H; m; H-10’, H-12’); 7.31-7.37 (2H; m; H-9’, H-13’); 7.90 (1H; s; Hz; H-9); 8.75 (1H; s; C(16)-OH); 9.43 (1H; s; C(10)-NH-C(1”)). ^13^C NMR (125.7 MHz; DMSO-*d*_6_) *δ* (ppm): 7.4 (C-18); 20.7 (C(17)-OCOCH_3_); 30.4 (C-19); 38.8 (N(1)-CH_3_); 42.4 (C-20); 43.6 (C-6); 50.3 (C-3); 51.1 (C-5); 51.6 (C(16)-COOCH_3_); 52.5 (C-7); 52.6 (C-2’, C-6’); 52.7 (C-3’, C-5’); 56.0 (C(11)-OCH_3_); 60.9 (C-7’); 61.1 (C-2”); 66.1 (C-21); 75.8 (C-17); 78.7 (C-16); 82.7 (C-2); 94.0 (C-12); 113.9 (C-9); 114.8 (d; *J* = 21.1 Hz; C-10’, C-12’); 119.4 (C-10); 123.5 (C-8); 124.4 (C-14); 129.8 (C-15); 130.6 (d; *J* = 7.9 Hz; C-9’, C-13’); 134.0 (d; *J* = 2.8 Hz; C-8’); 148.6 (C-13); 149.5 (C-11); 161.2 (d; *J* = 242.3 Hz; C-11’); 166.9 (C-1”); 170.0 (C(17)-OCOCH_3_); 171.5 (C(16)-COOCH_3_). HRMS: M+H= 706.36109; (delta = 0.1 ppm; C_38_H_49_O_7_N_5_F).

**Figure S25.** The skeleton numbering of compound **19** used for NMR assignment.


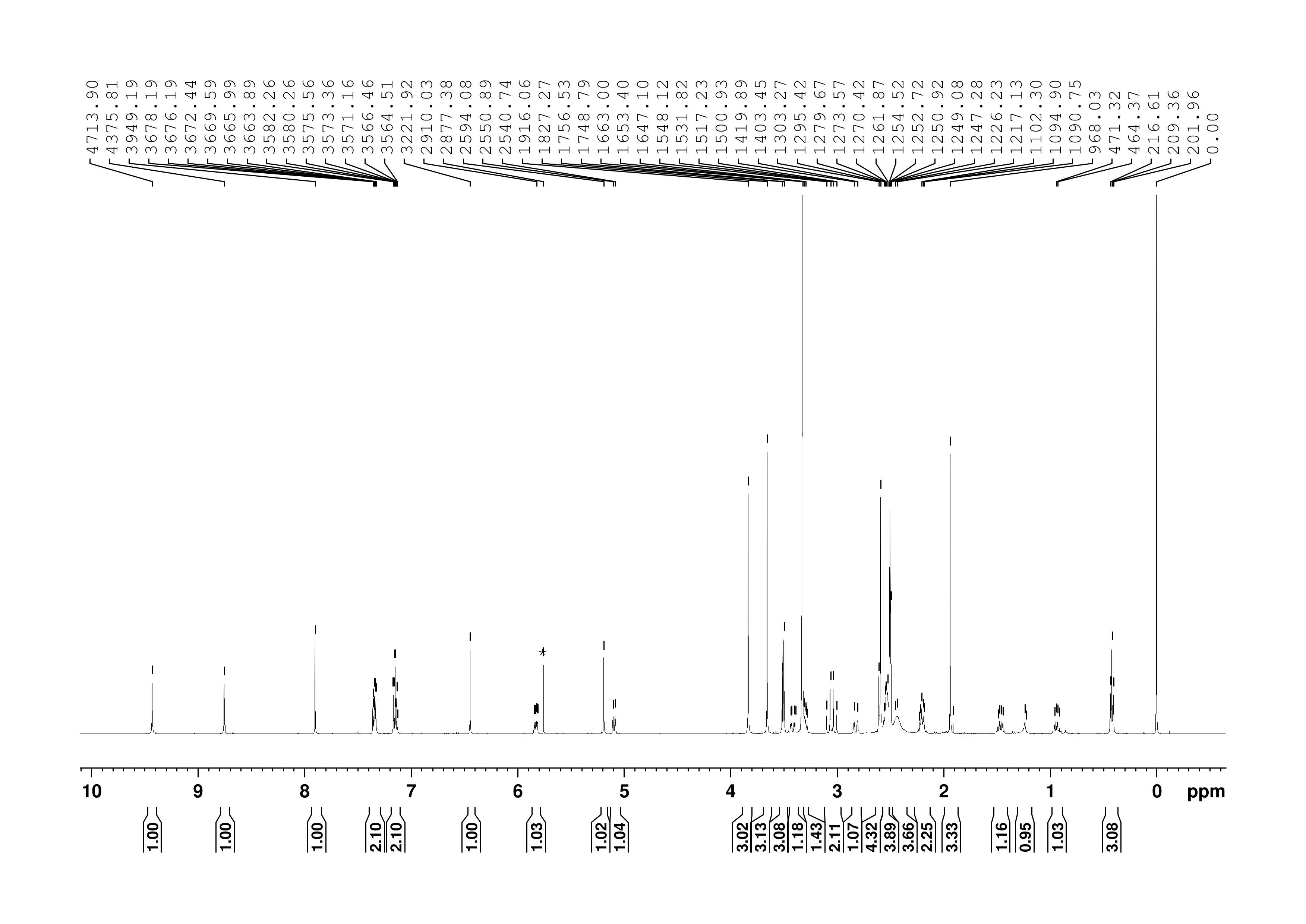


**Figure S26.** ^1^H NMR spectrum of compound **19.**


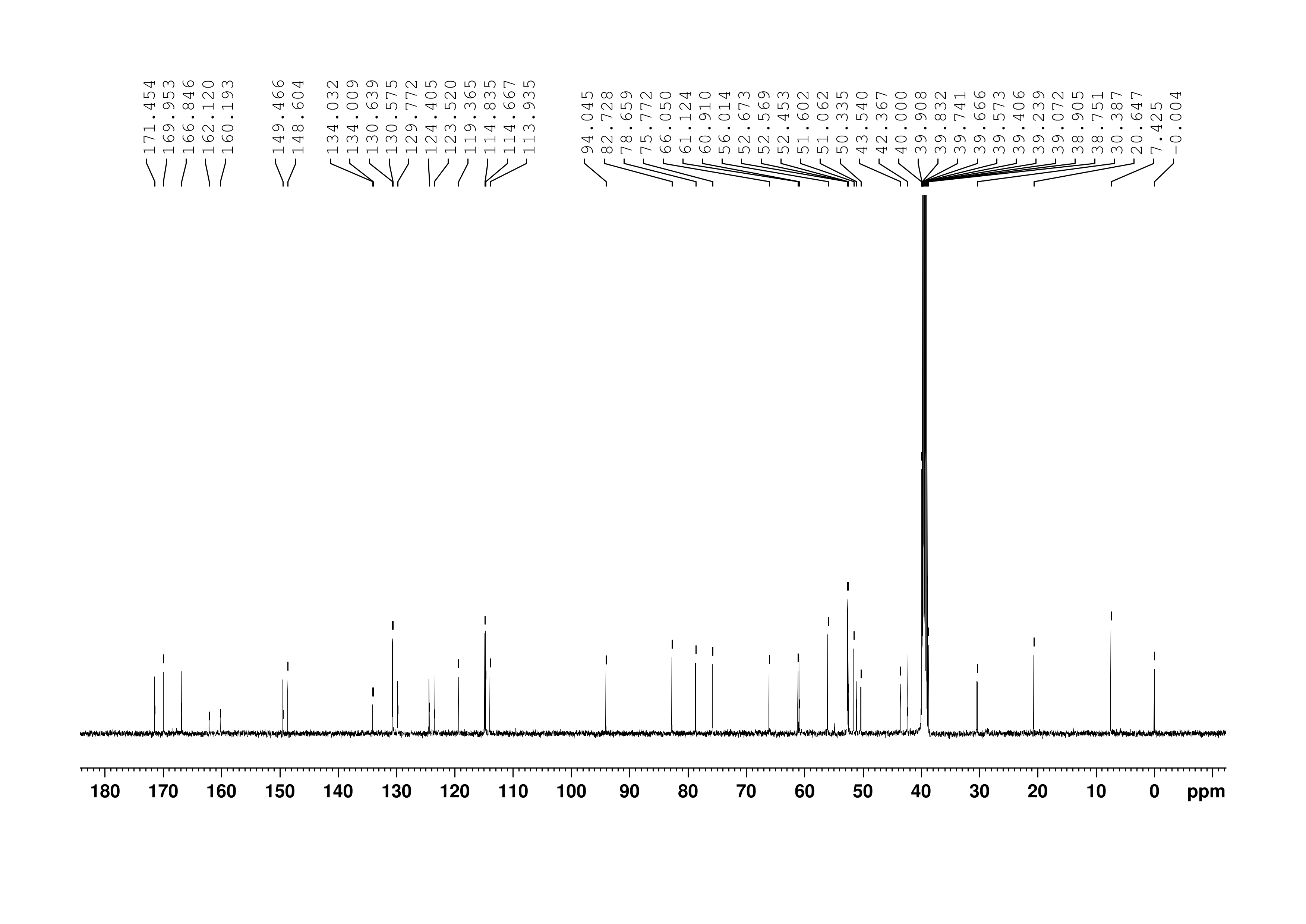


**Figure S27.** ^13^C NMR spectrum of compound **19.**


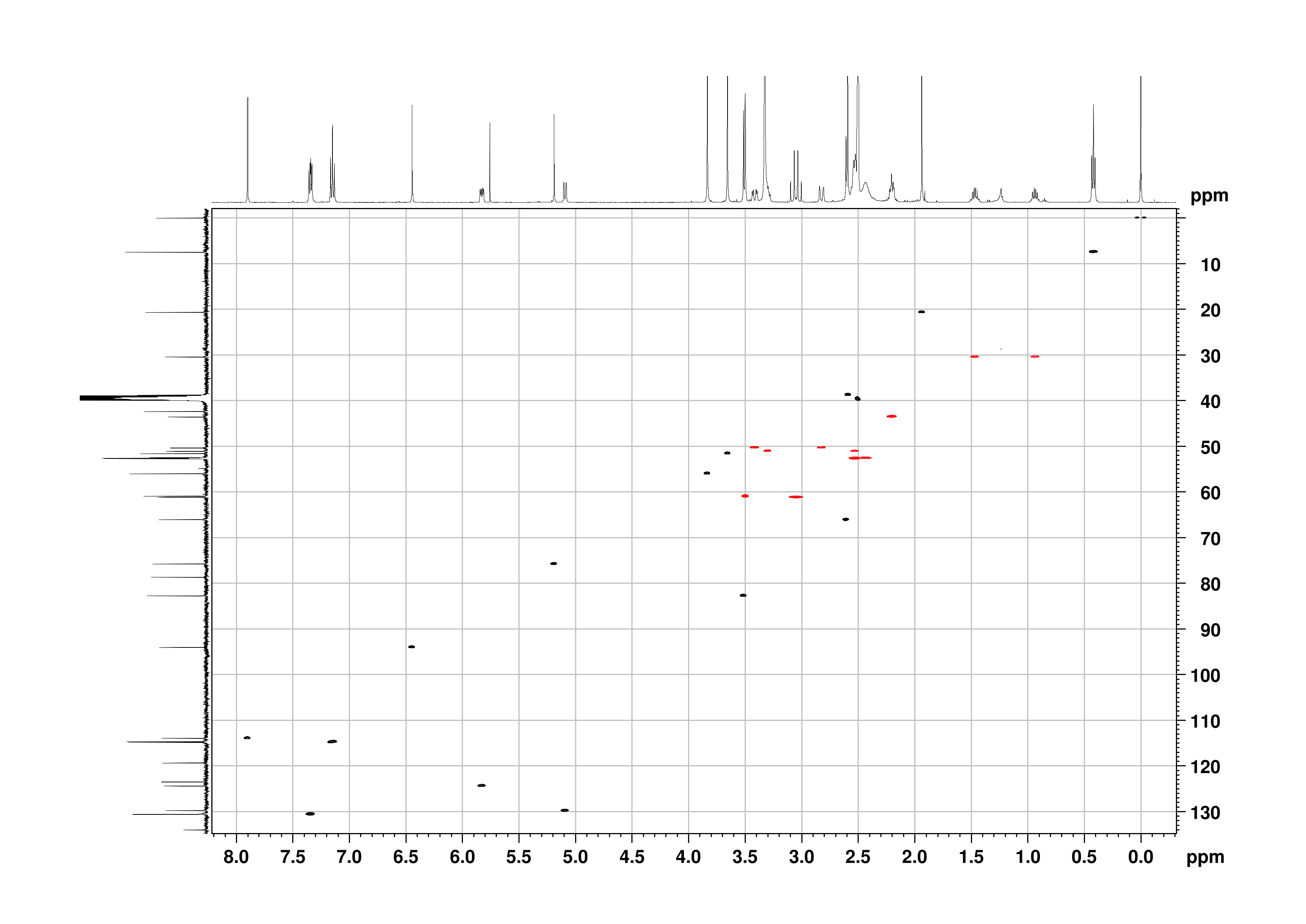


**Figure S28.** HSQC spectrum of compound **19.**


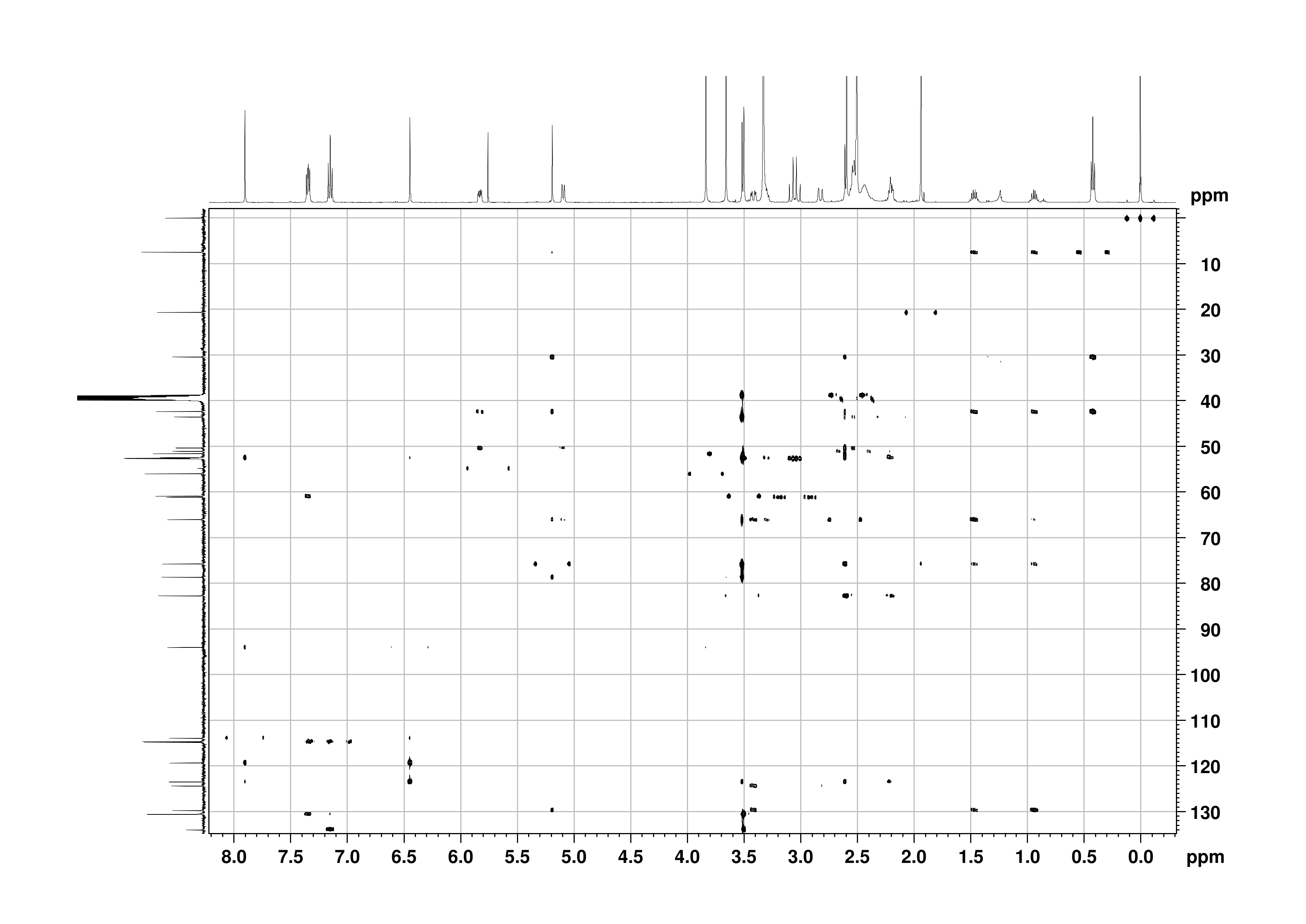


**Figure S29.** ^1^H-^13^C HMBC spectrum of compound **19.**


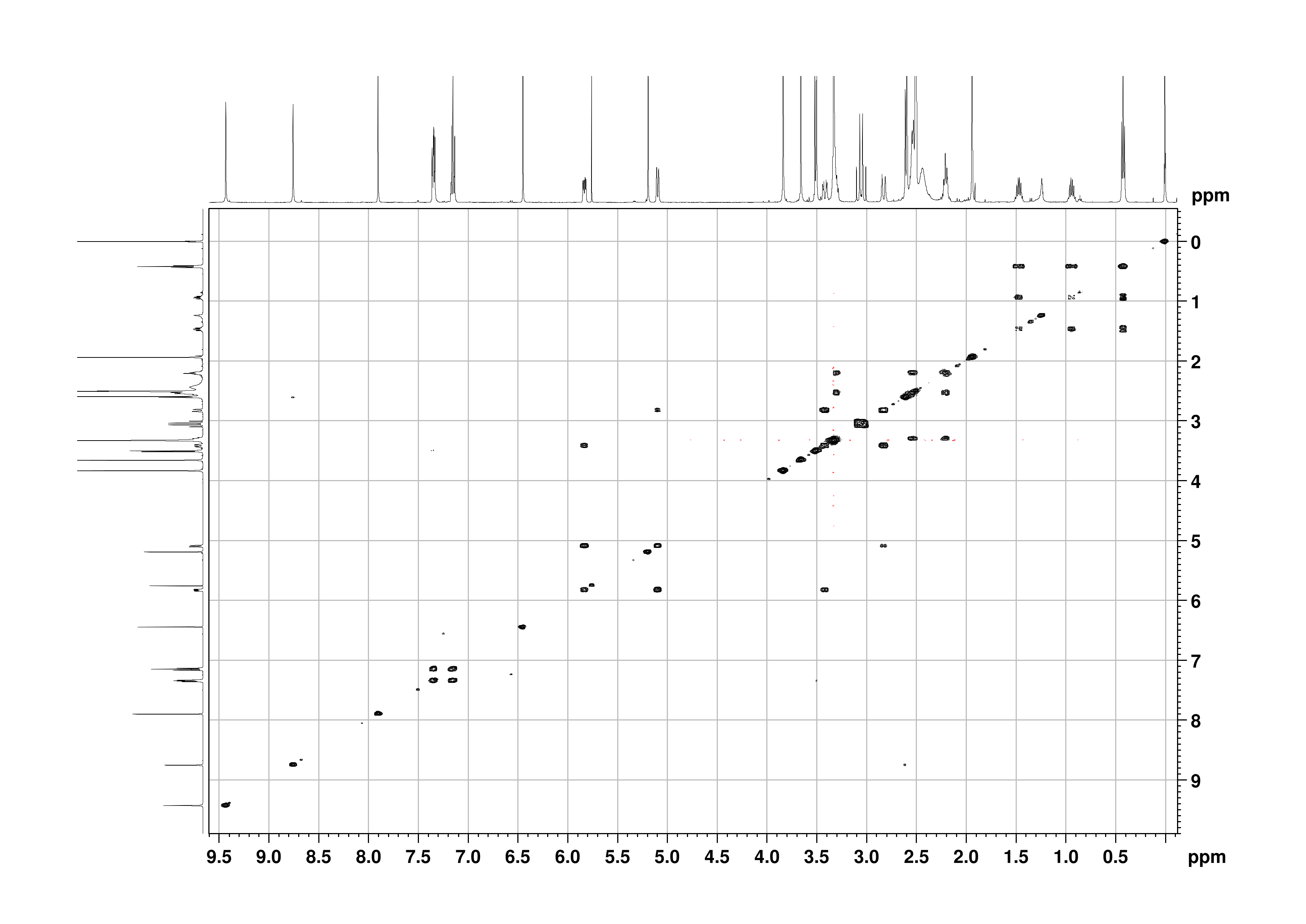


**Figure S30.** COSY spectrum of compound **19.**


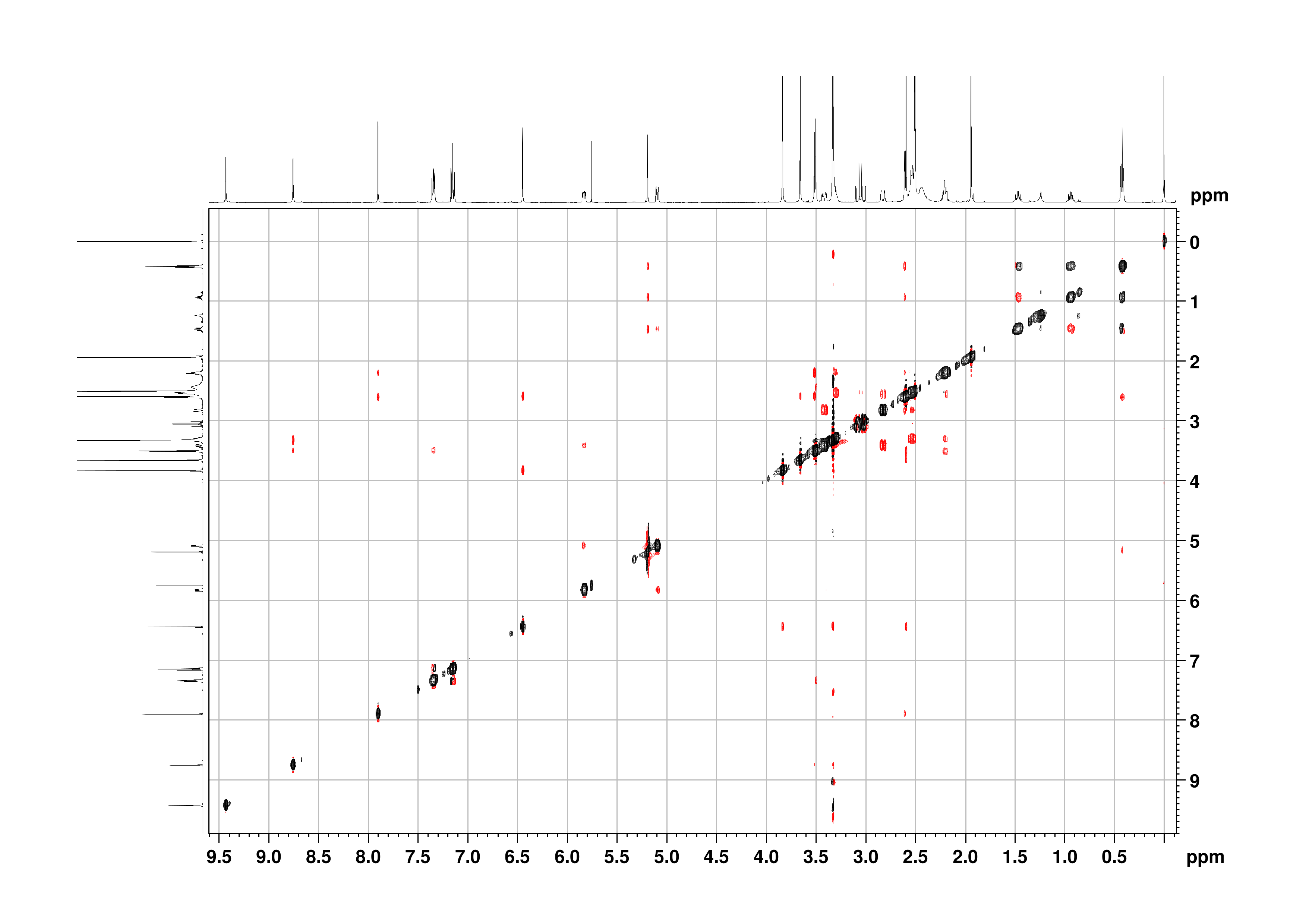


**Figure S31.** ROESY spectrum of compound **19.**

**Figure S32.** HRMS spectrum of compound **19.**

Product **20**

67 mg (46%). M.p.: 130-131 °C. TLC (DCM : MeOH = 15 : 1); *R_f_* = 0.47. IR (KBr) 3296, 2929, 2346, 1740, 1505, 1220, 1008, 827 cm^-1^. ^1^H NMR (499.9 MHz; DMSO-*d*_6_) *δ* (ppm): 0.41 (3H; t; *J* = 7.3 Hz; H_3_-18); 0.92 (1H; dq; *J* = 14.2, 7.3 Hz; H_x_-19); 1.46 (1H; dq; *J* = 14.2, 7.4 Hz; H_y_-19); 1.93 (3H; s; C(17)-OCOCH_3_); 2.11-2.46 (6H; m; H_2_-6, H_2_-3’, H_2_-5’); 2.49-2.63 (9H; m; N(1)-CH_3_, H_x_-5, H-21, H_2_-2’, H_2_-6’); 2.81 (1H; br d; *J* = 16.4 Hz; H_x_-3); 3.02 (1H; d; *J* = 16.3 Hz; H_x_-2”); 3.09 (1H; d; *J* = 16.3 Hz; H_y_-2”); 3.25-3.34 (1H; m; H_y_-5); 3.41 (1H; br dd; *J* = 16.6, 4.4 Hz; H_y_-3); 3.51 (1H; s; H-2); 3.65 (3H; s; C(16)-COOCH_3_); 3.80 (3H; s; C(11)-OCH_3_); 4.46 (1H; s; H-7’); 5.09 (1H; br d; *J* = 10.1 Hz; H-15); 5.18 (1H; s; H-17); 5.82 (1H; ddd; *J* = 10.2, 4.8, 1.4 Hz; H-14); 6.43 (1H; s; H-12); 7.14 (4H; ~t; *J* = 8.8 Hz; 4×C(7’)-PhF: H_meta_); 7.41-7.47 (4H; m; 4×C(7’)-PhF: H_orto_); 7.88 (1H; s; Hz; H-9); 8.75 (1H; s; C(16)-OH); 9.40 (1H; s; C(10)-NH-C(1”)). ^13^C NMR (125.7 MHz; DMSO-*d*_6_) *δ* (ppm): 7.4 (C-18); 20.6 (C(17)-OCOCH_3_); 30.4 (C-19); 38.7 (N(1)-CH_3_); 42.4 (C-20); 43.5 (C-6); 50.3 (C-3); 51.1 (C-5); 51.4 (C-3’, C-5’); 51.6 (C(16)-COOCH_3_); 52.5 (C-7); 52.8 (C-2’, C-6’); 56.0 (C(11)-OCH_3_); 61.1 (C-2”); 66.0 (C-21); 72.6 (C-7’); 75.8 (C-17); 78.7 (C-16); 82.7 (C-2); 94.0 (C-12); 114.0 (C-9); 115.2 (d; *J* = 21.1 Hz; 4×C(7’)-PhF: C_meta_); 119.3 (C-10); 123.5 (C-8); 124.4 (C-14); 129.4 (d; *J* = 8.0 Hz; 4×C(7’)-PhF: C_orto_); 129.8 (C-15); 138.3 (d; *J* = 2.6; 2×C(7’)-PhF: C_ipszo_); 148.6 (C-13); 149.5 (C-11); 161.0 (d; *J* = 243.1 Hz; 2×C(7’)-PhF: C_para_); 166.8 (C-1”); 170.0 (C(17)-OCOCH_3_); 171.5 (C(16)-COOCH_3_). HRMS: M+H=800.38305 (delta = 0.2 ppm; C_44_H_52_O_7_N_5_F_2_).

**Figure S33.** The skeleton numbering of compound **20** used for NMR assignment.


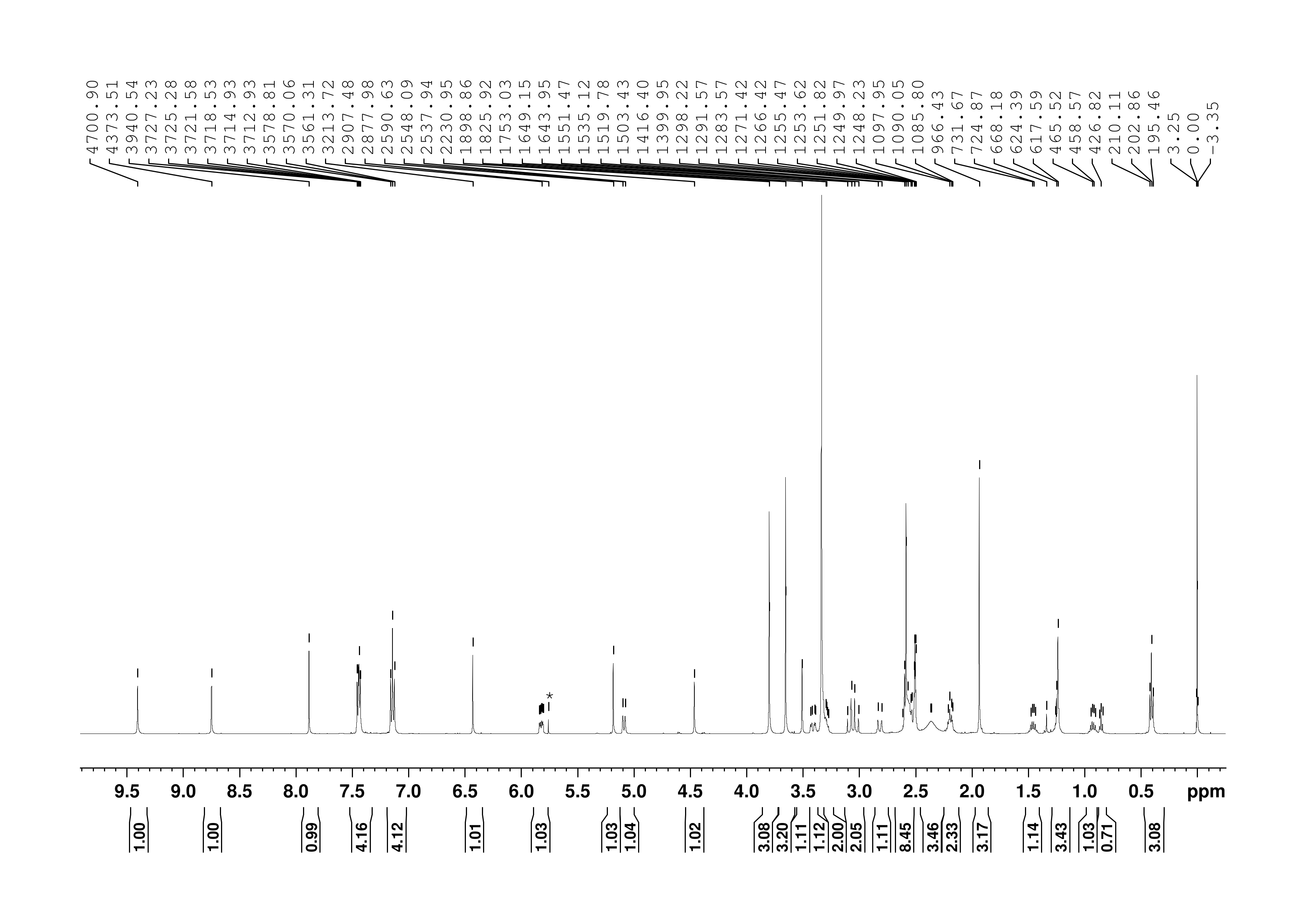


**Figure S34.** ^1^H NMR spectrum of compound **20.**


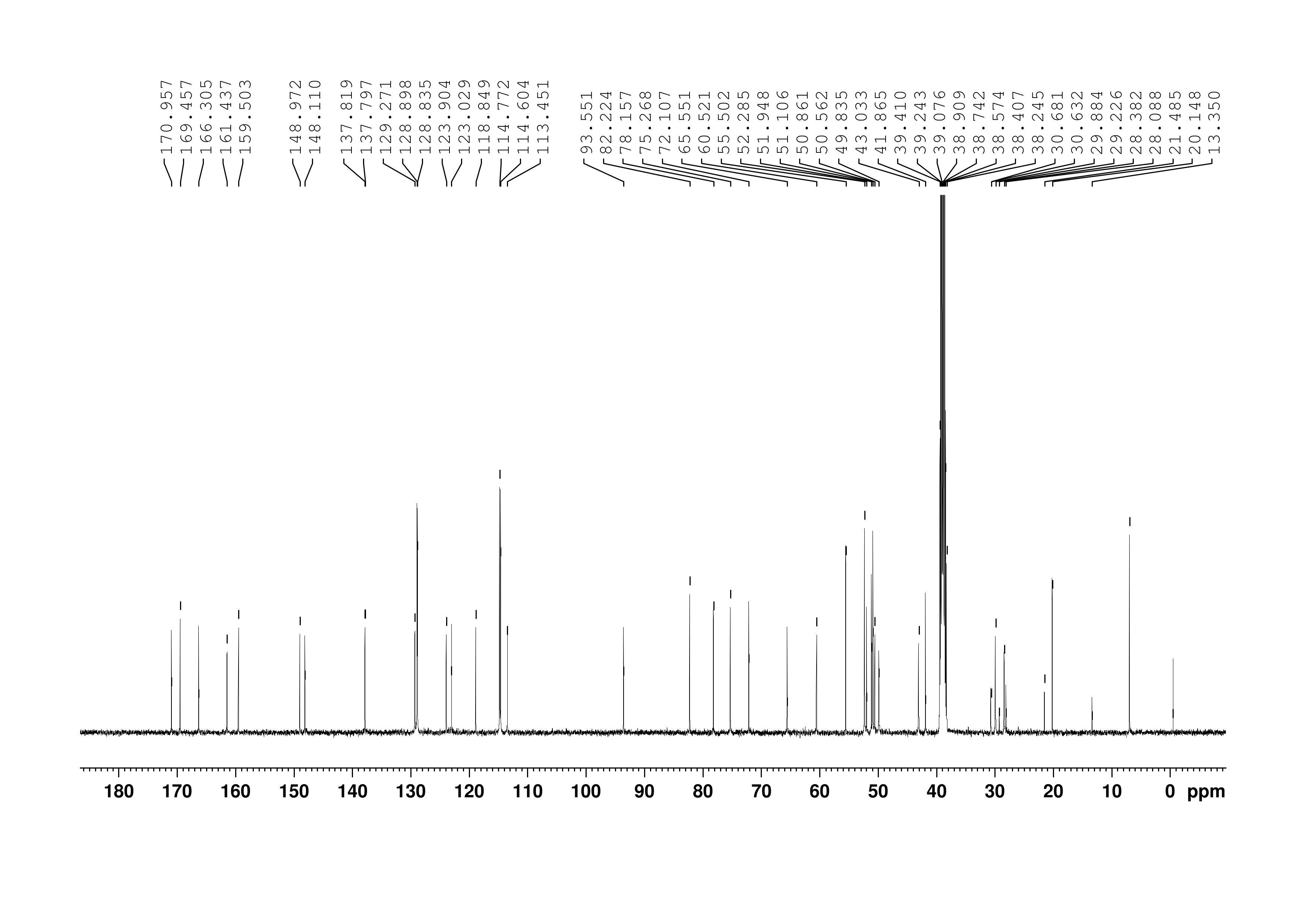


**Figure S35.** ^13^C NMR spectrum of compound **20.**


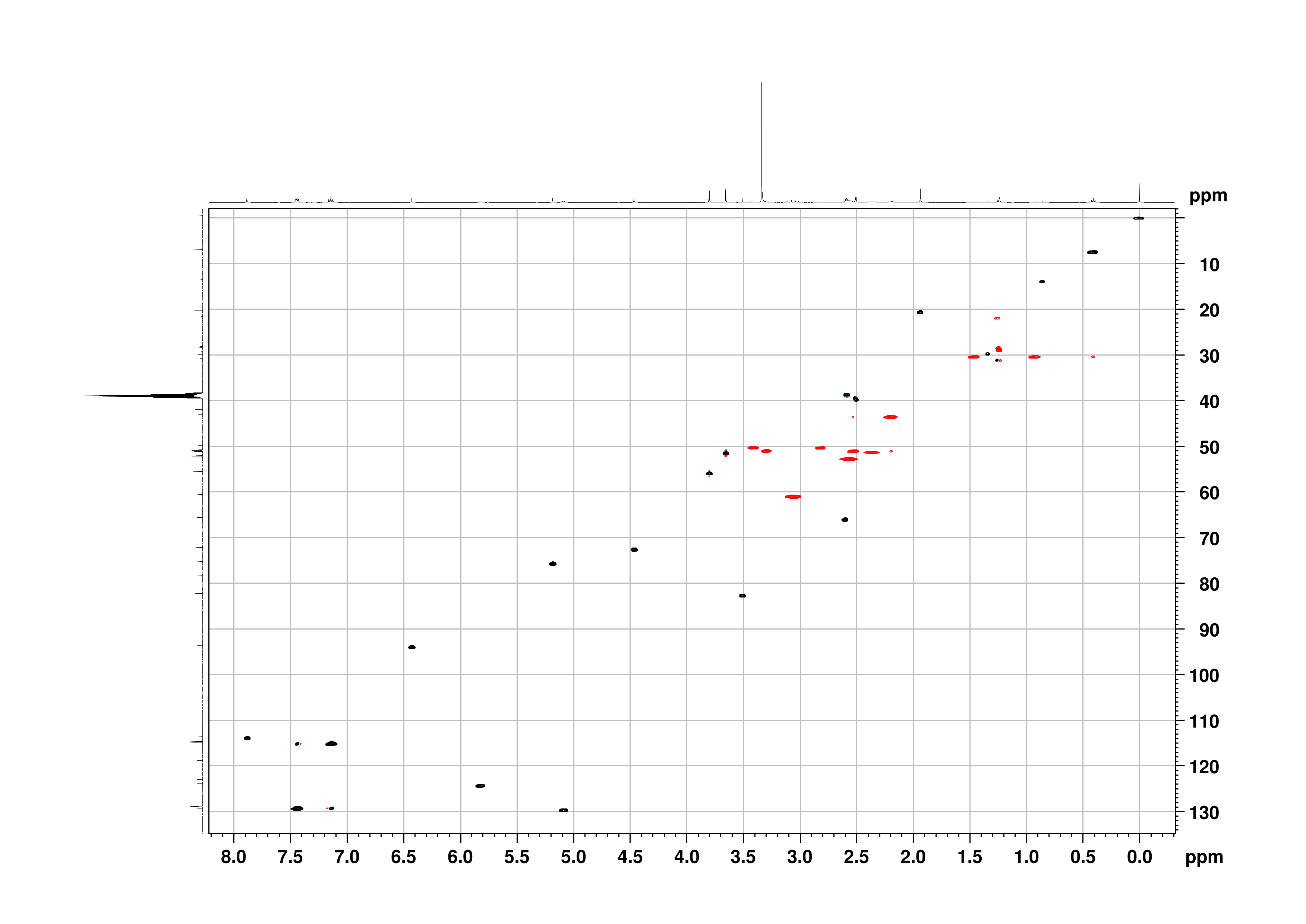


**Figure S36.** HSQC spectrum of compound **20.**


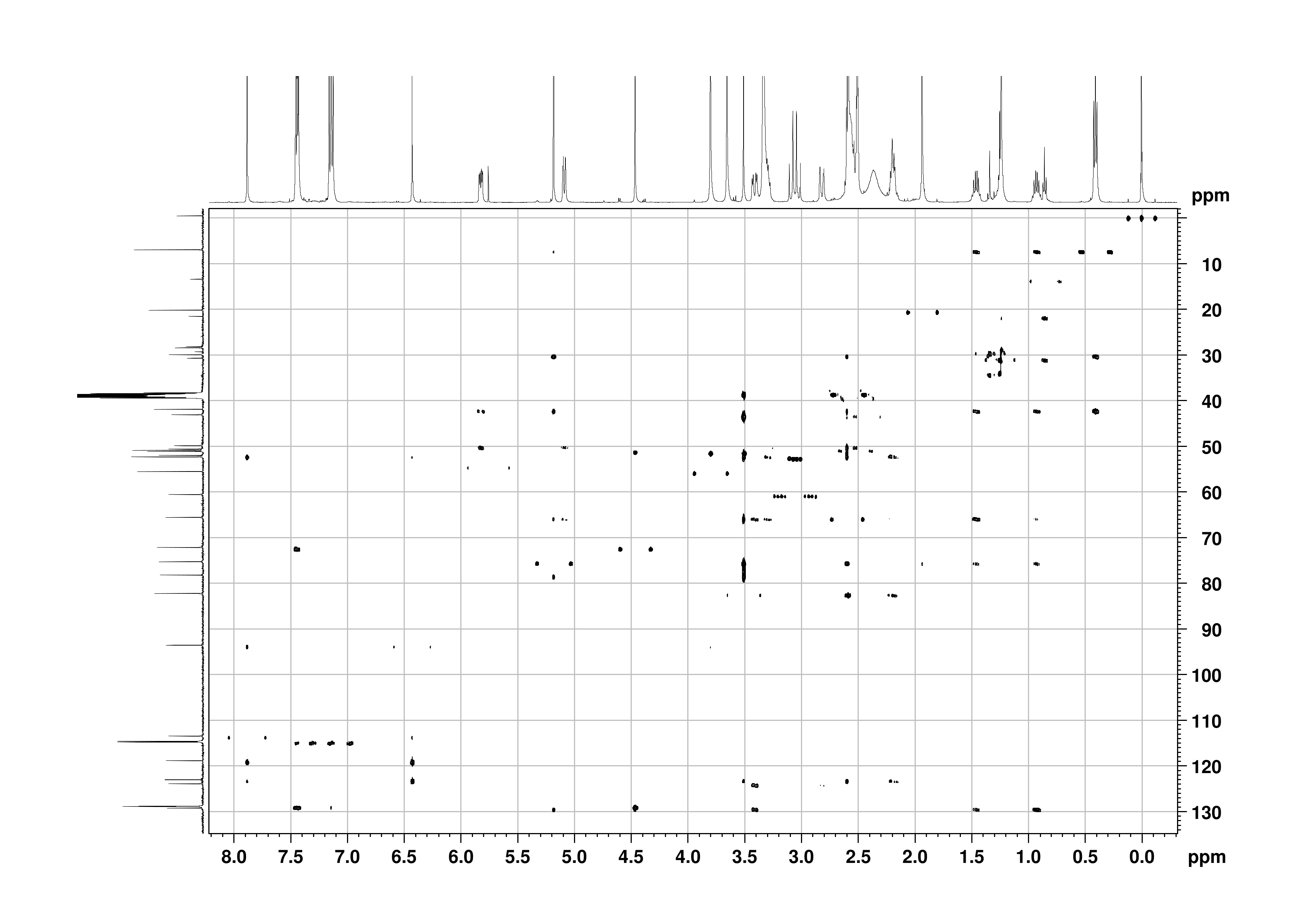


**Figure S37.** ^1^H-^13^C HMBC spectrum of compound **20.**


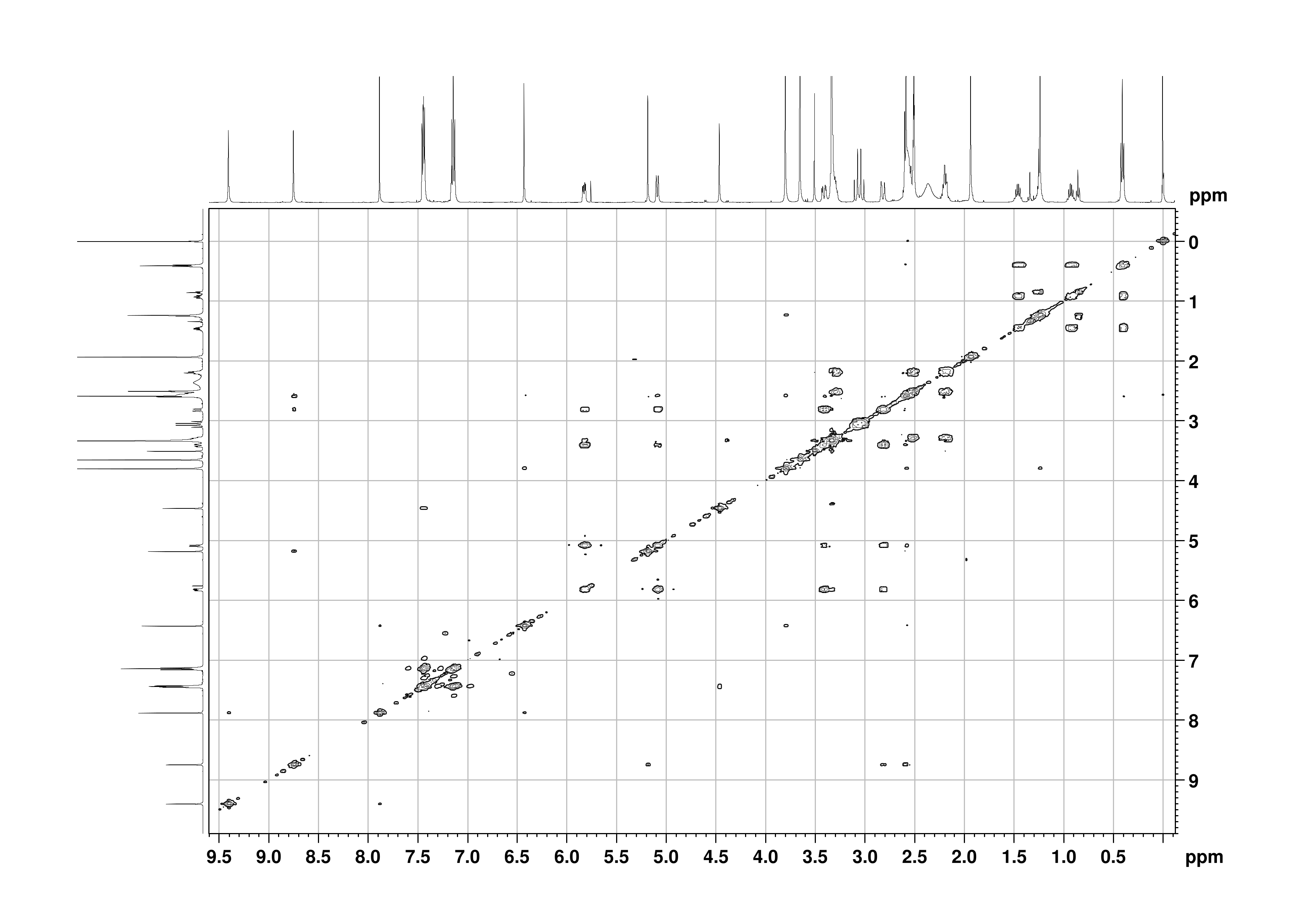


**Figure S38.** COSY spectrum of compound **20.**


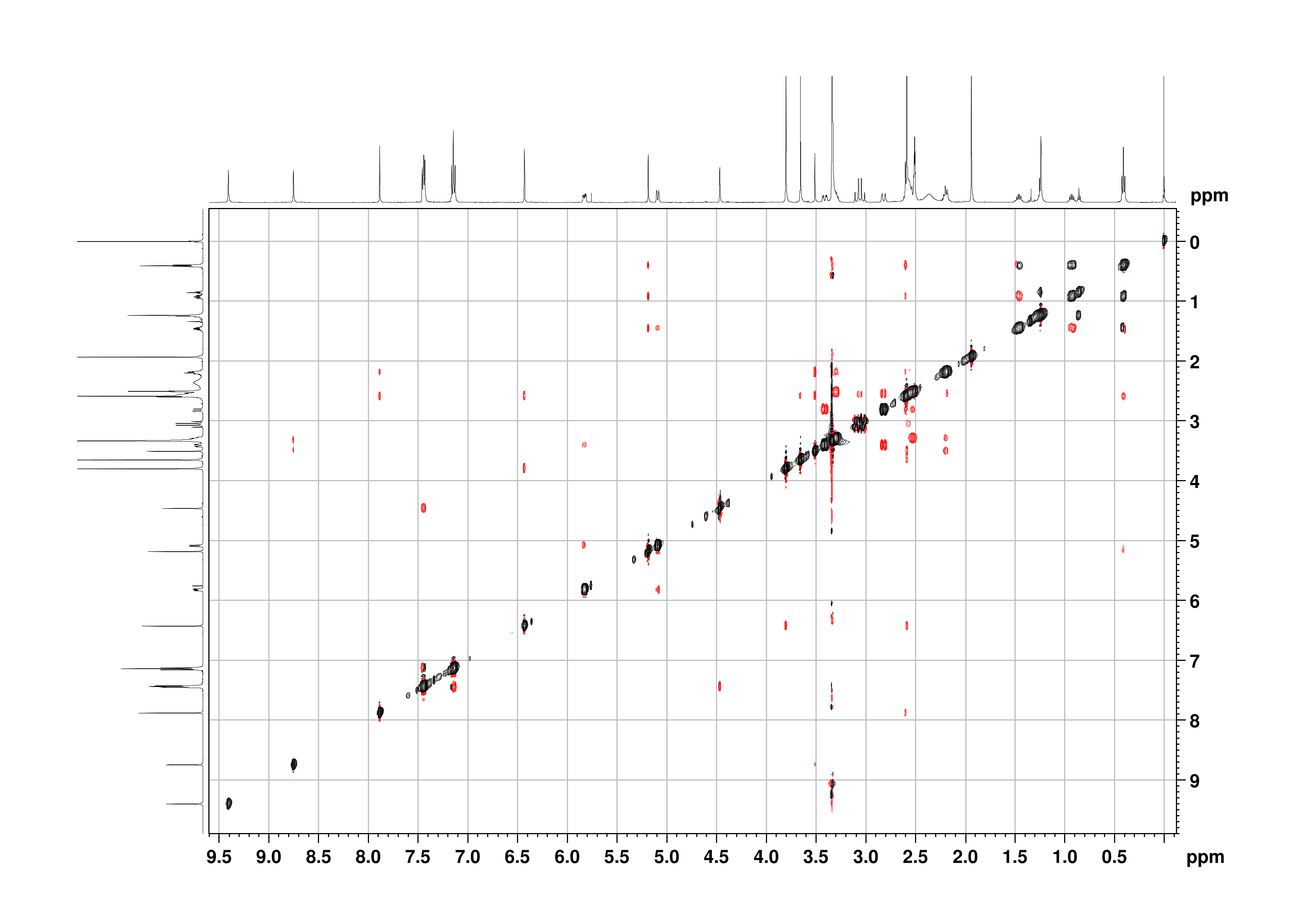


**Figure S39.** ROESY spectrum of compound **20.**

**Figure S40.** HRMS spectrum of compound **20.**

Product **21**

86 mg (68%). M.p.: 127-128 °C. TLC (DCM : MeOH = 15 : 1); *R_f_* = 0.35. IR (KBr) 3309, 3116, 2930, 2816, 1738, 1523, 1222 cm^-1^. ^1^H NMR (499.9 MHz; DMSO-*d*_6_) *δ* (ppm): 0.44 (3H; t; *J* = 7.3 Hz; H_3_-18); 0.95 (1H; dq; *J* = 14.2, 7.3 Hz; H_x_-19); 1.48 (1H; dq; *J* = 14.2, 7.3 Hz; H_y_-19); 1.94 (3H; s; C(17)-OCOCH_3_); 2.16-2.26 (2H; m; H_2_-6); 2.50-2.64 (9H; m; N(1)-CH_3_, H_x_-5, H-21, H_2_-2’, H_2_-6’); 2.83 (1H; br d; *J* = 16.4 Hz; H_x_-3); 3.10 (1H; d; *J* = 16.2 Hz; H_x_-2”); 3.17 (1H; d; *J* = 16.2 Hz; H_y_-2”); 3.26-3.34 (1H; m; H_y_-5); 3.42 (1H; br dd; *J* = 16.8, 4.6 Hz; H_y_-3); 3.52 (1H; s; H-2); 3.66 (3H; s; C(16)-COOCH_3_); 3.73 (4H; br s; H_2_-3’, H_2_-5’); 3.87 (3H; s; C(11)-OCH_3_); 5.10 (1H; br d; *J* = 10.2 Hz; H-15); 5.20 (1H; s; H-17); 5.83 (1H; ddd; *J* = 10.2, 4.8, 1.3 Hz; H-14); 6.45 (1H; s; H-12); 6.63 (1H; dd; *J* = 3.4, 1.8 Hz; H-11’); 7.01 (1H; dd; *J* = 3.4, 0.7 Hz; H-12’); 7.84 (1H; dd; *J* = 1.7, 0.7 Hz; H-10’); 7.87 (1H; s; Hz; H-9); 8.76 (1H; s; C(16)-OH); 9.38 (1H; s; C(10)-NH-C(1”)). ^13^C NMR (125.7 MHz; DMSO-*d*_6_) *δ* (ppm): 7.4 (C-18); 20.7 (C(17)-OCOCH_3_); 30.4 (C-19); 38.8 (N(1)-CH_3_); 42.4 (C-20); 43.6 (C-6); 50.3 (C-3); 51.1 (C-5); 51.6 (C(16)-COOCH_3_); 52.5 (C-7); 52.7 (C-2’, C-6’); 56.1 (C(11)-OCH_3_); 61.0 (C-2”); 66.0 (C-21); 75.8 (C-17); 78.7 (C-16); 82.7 (C-2); 94.0 (C-12); 111.2 (C-11’); 114.5 (C-9); 115.5 (C-12’); 119.2 (C-10); 123.5 (C-8); 124.4 (C-14); 129.8 (C-15); 144.7 (C-10’); 146.8 (C-8’); 148.8 (C-13); 149.8 (C-11); 158.2 (C-7’); 166.7 (C-1”); 170.0 (C(17)-OCOCH_3_); 171.5 (C(16)-COOCH_3_). HRMS: M+H=692.32903 (delta = 0.04 ppm; C_36_H_46_O_9_N_5_).

**Figure S41.** The skeleton numbering of compound **21** used for NMR assignment.


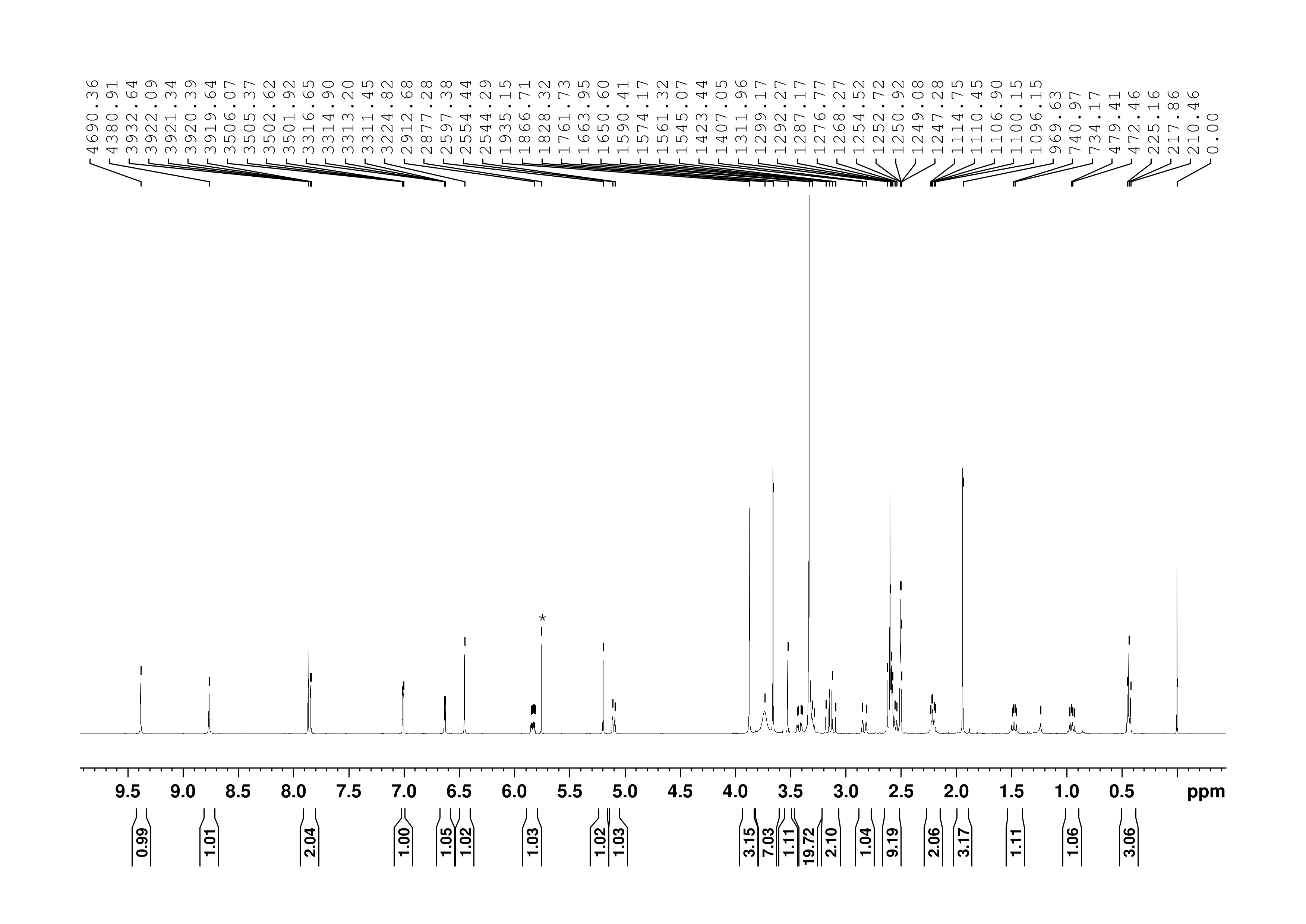


**Figure S42.** ^1^H NMR spectrum of compound **21.**


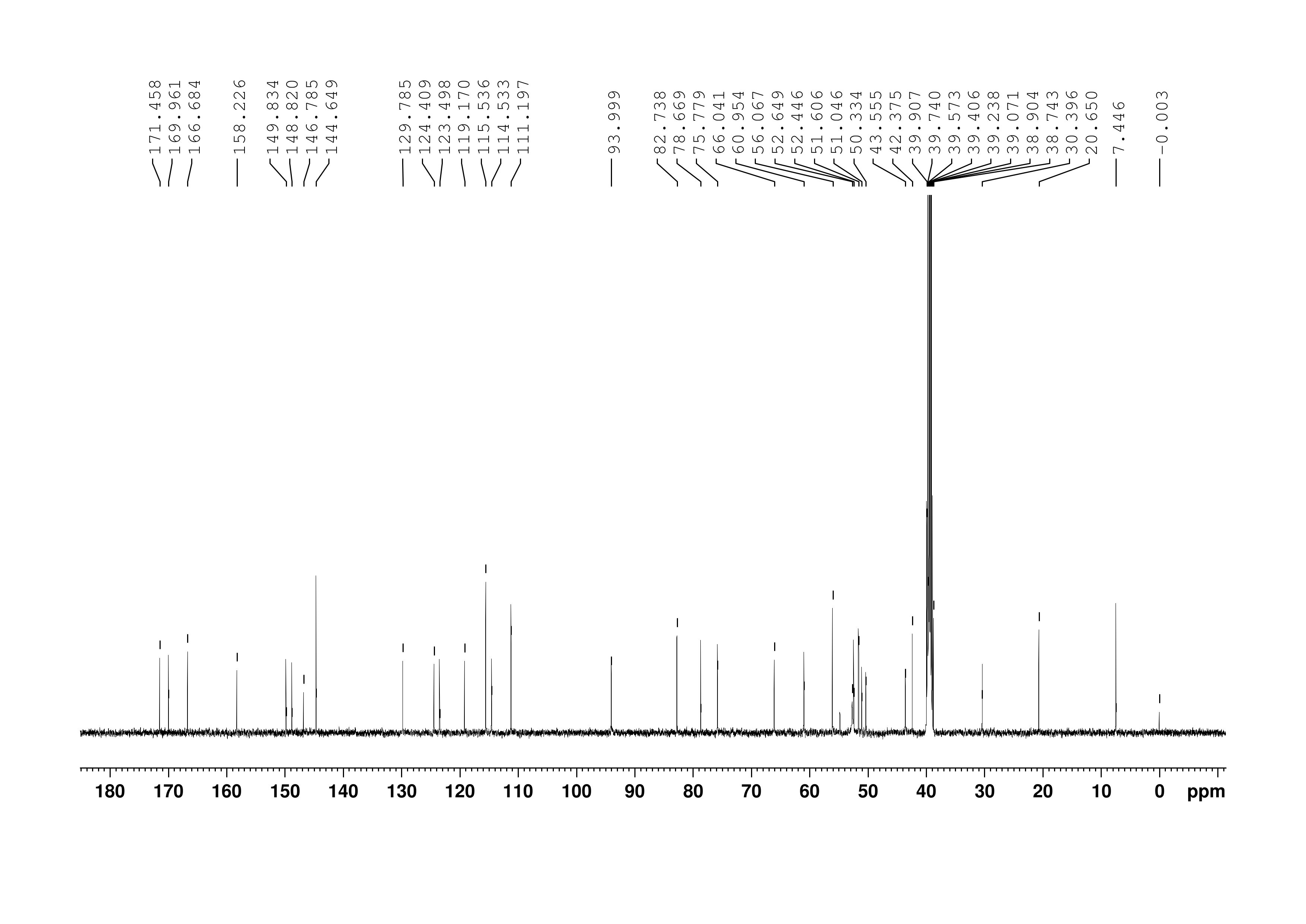


**Figure S43.** ^13^C NMR spectrum of compound **21.**


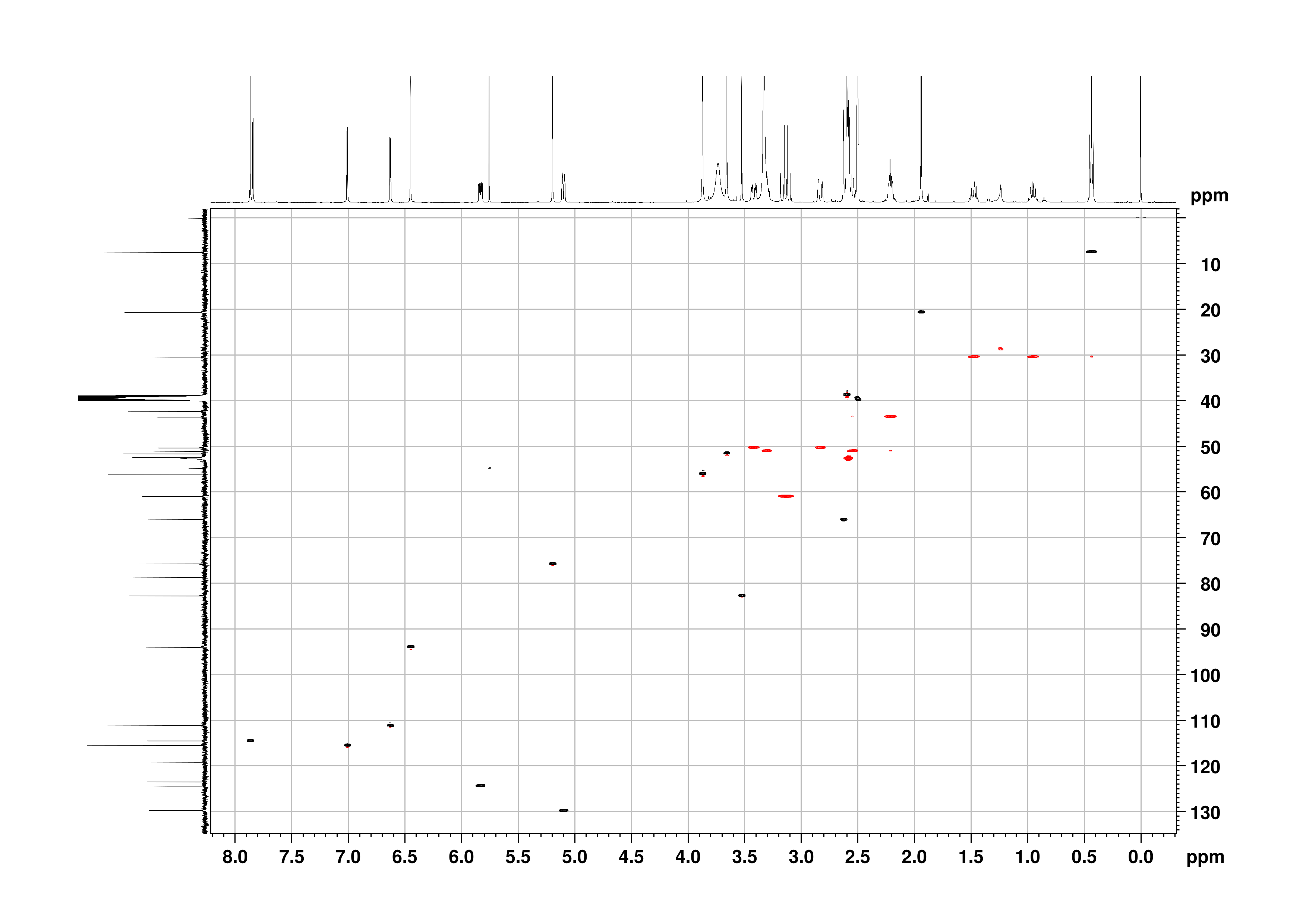


**Figure S44.** HSQC spectrum of compound **21.**


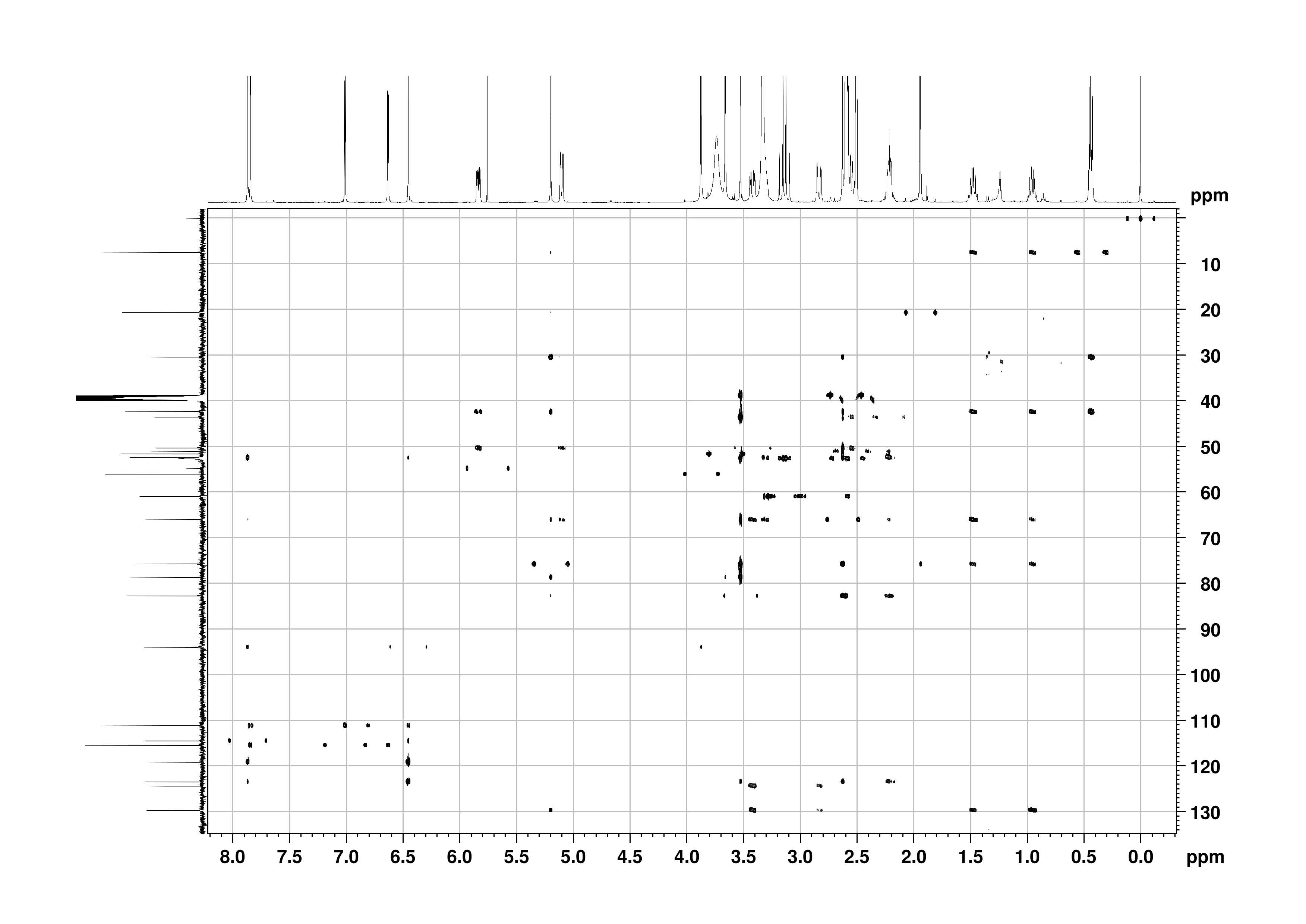


**Figure S45.** ^1^H-^13^C HMBC spectrum of compound **21.**


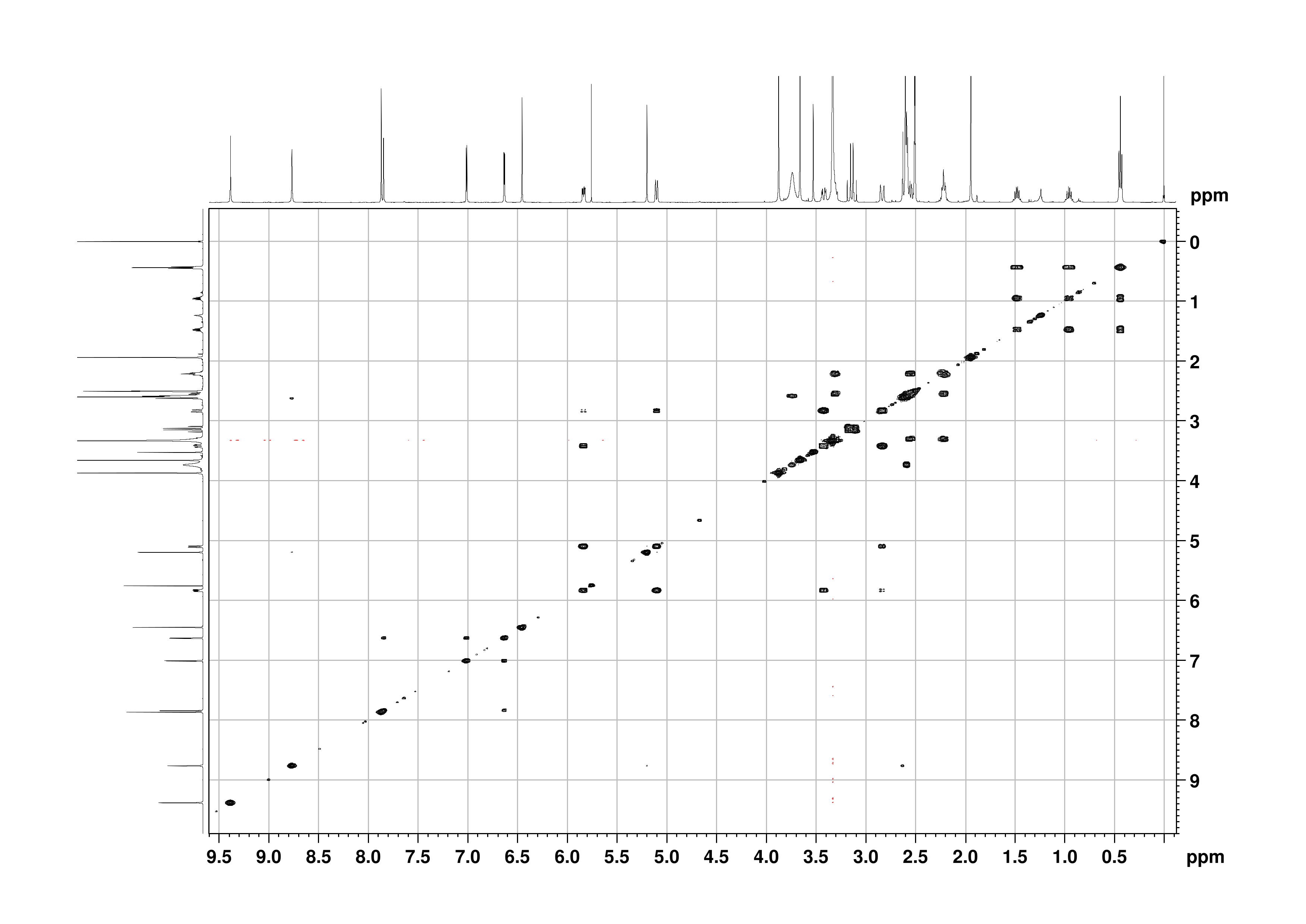


**Figure S46.** COSY spectrum of compound **21.**


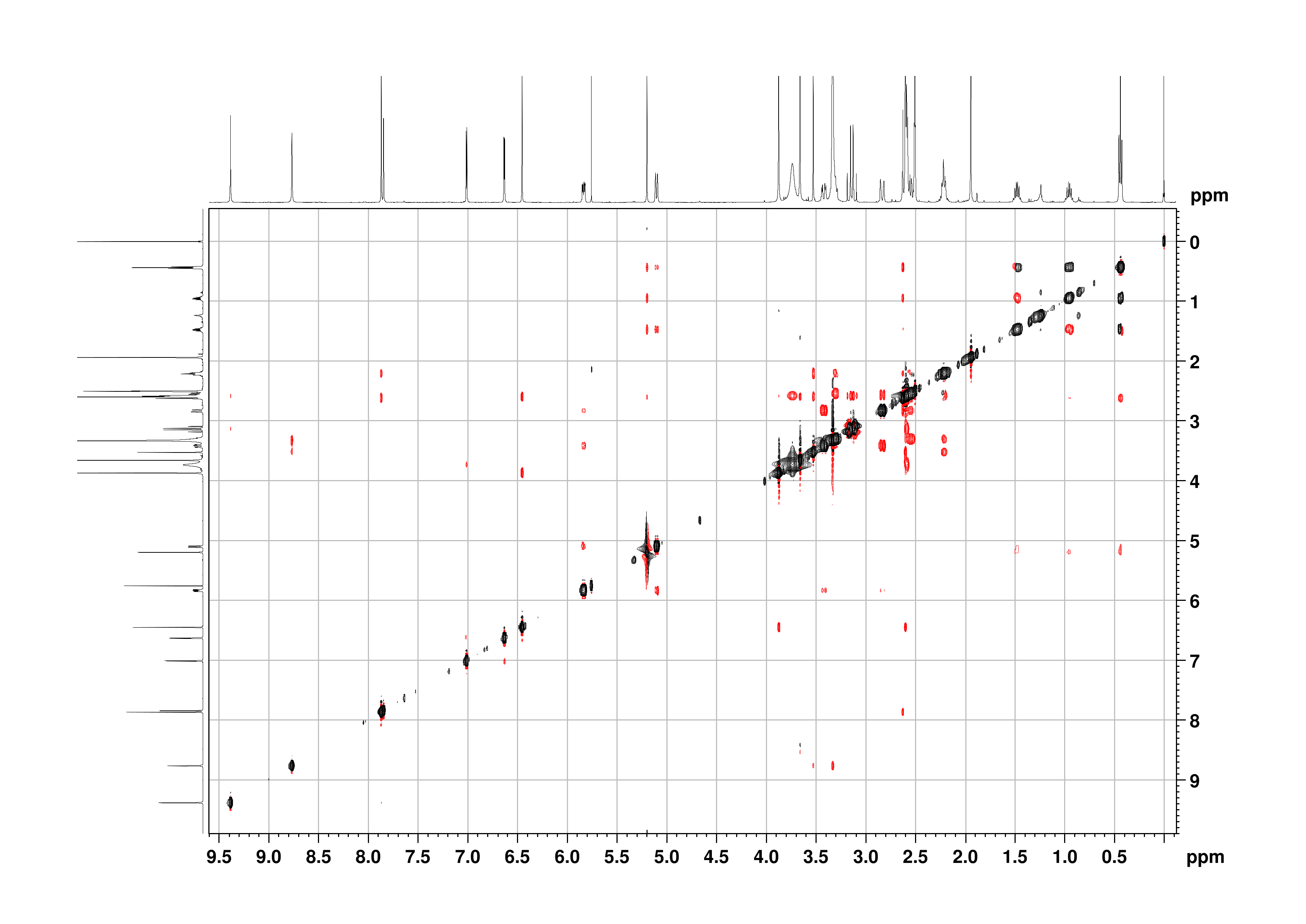


**Figure S47.** ROESY spectrum of compound **21.**

**Figure S48.** HRMS spectrum of compound **21.**

**S.1.2.2. General procedure for the synthesis of products (3 and 22-26)**

**Method A:** 17-(*O*-4-Bromobutanoyl)vindoline (**14**) (100 mg, 0.18 mmol), the appropriate piperazine (**5**, **7**, **9**) (0.36 mmol, 2.0 eq.), and triethylamine (25 µL, 0.18 mmol, 1.0 eq.) were dissolved in DCM (5 mL). The reaction mixture was refluxed at the time presented in Scheme 2. Then, the mixture was filtered, and the filtrate was evaporated under reduced pressure. The pale yellow crystalline products (**3**, **23**, and **25**) were isolated after preparative TLC (DCM : MeOH = 12 : 1).

**Method B:** 17-(*O*-4-Bromobutanoyl)vindoline (**14**) (100 mg, 0.18 mmol), the appropriate piperazine (**6**, **8**, **10**) (0.18 mmol, 1.0 eq.), and anhydrous potassium carbonate (25 mg, 0.18 mmol, 1.0 eq.) were dissolved in MeCN (5 mL). The reaction mixture was refluxed at the time presented in Scheme 2. Then, the mixture was filtered, and the filtrate was evaporated under reduced pressure. The pale yellow crystalline products (**22**, **24**, and **26**) were isolated after preparative TLC (DCM : MeOH = 12 : 1).

Product **3**

58 mg (56%). ^1^H NMR, m.p., and *R_f_* data were in good agreement with the literature [26].

Product **22**

82 mg (65%). M.p.: 75-76 °C. TLC (DCM : MeOH = 10 : 1); *R_f_* = 0.60. IR (KBr) 1736, 1613, 1500, 1328, 1235, 1160, 1104, 1069, 1023, 823 cm^-1^. ^1^H NMR (499.9 MHz; DMSO-*d*_6_) *δ* (ppm): 0.40 (3H; t; *J* = 7.4 Hz; H_3_-18); 0.96 (1H; dq; *J* = 14.1, 7.2 Hz; H_x_-19); 1.50 (1H; dq; *J* = 14.2, 7.4 Hz; H_y_-19); 1.60-1.75 (2H; m; H_2_-3’); 2.16-2.35 (6H; m; H_2_-6, H_2_-2’, H_2_-4’); 2.47 (4H; ~br t; *J* = 5.0 Hz; H_2_-6’, H_2_-10’); 2.52-2.61 (4H; m; N(1)-CH_3_, H_x_-5); 2.65 (1H; s; H-21); 2.79 (1H; br d; *J* = 16.4 Hz; H_x_-3); 3.21-3.32 (5H; m; H_y_-5, H_2_-7’, H_2_-9’); 3.41 (1H; br dd; *J* = 16.2, 4.9 Hz; H_y_-3); 3.54 (1H; s; H-2); 3.65 (3H; s; C(16)-COOCH_3_); 3.71 (3H; s; C(11)-OCH_3_); 5.11 (1H; br d; *J* = 10.1 Hz; H-15); 5.21 (1H; s; H-17); 5.82 (1H; ddd; *J* = 10.2, 4.8, 1.3 Hz; H-14); 6.20 (1H; d; *J* = 2.2 Hz; H-12); 6.28 (1H; dd; *J* = 8.2, 2.2 Hz; H-10); 7.02-7.07 (3H; m; H-9, H-13’, H-17’); 7.49 (2H; ~d; *J* = 8.8 Hz; H-14’, H-16’); 8.77 (1H; s; C(16)-OH). ^13^C NMR (125.7 MHz; DMSO-*d*_6_) *δ* (ppm): 7.5 (C-18); 21.4 (C-3’); 30.3 (C-19); 31.2 (C-2’); 38.0 (N(1)-CH_3_); 42.4 (C-20); 43.6 (C-6); 46.8 (C-7’, C-9’); 50.4 (C-3); 51.1 (C-5); 51.6 (C(16)-COOCH_3_); 52.0 (C-7); 52.3 (C-6’, C-10’); 55.0 (C(11)-OCH_3_); 56.6 (C-4’); 66.1 (C-21); 75.7 (C-17); 78.7 (C-16); 82.8 (C-2); 95.4 (C-12); 104.5 (C-10); 114.0 (C-13’, C-17’); 117.6 (q; *J* = 32.1 Hz; C-15’); 123.0 (C-9); 124.3 (C-14); 125.3 (C-8); 126.0 (q; *J* = 3.8 Hz; C-14’, C-16’); 129.9 (C-15); 153.2 (C-12’); 153.4 (C-13); 160.4 (C-11); 171.6 (C(16)-COOCH_3_); 172.4 (C-1’). HRMS: M+H=713.35162 (delta = -0.6 ppm; C_38_H_48_O_6_N_4_F_3_).

**Figure S49.** The skeleton numbering of compound **22** used for NMR assignment.


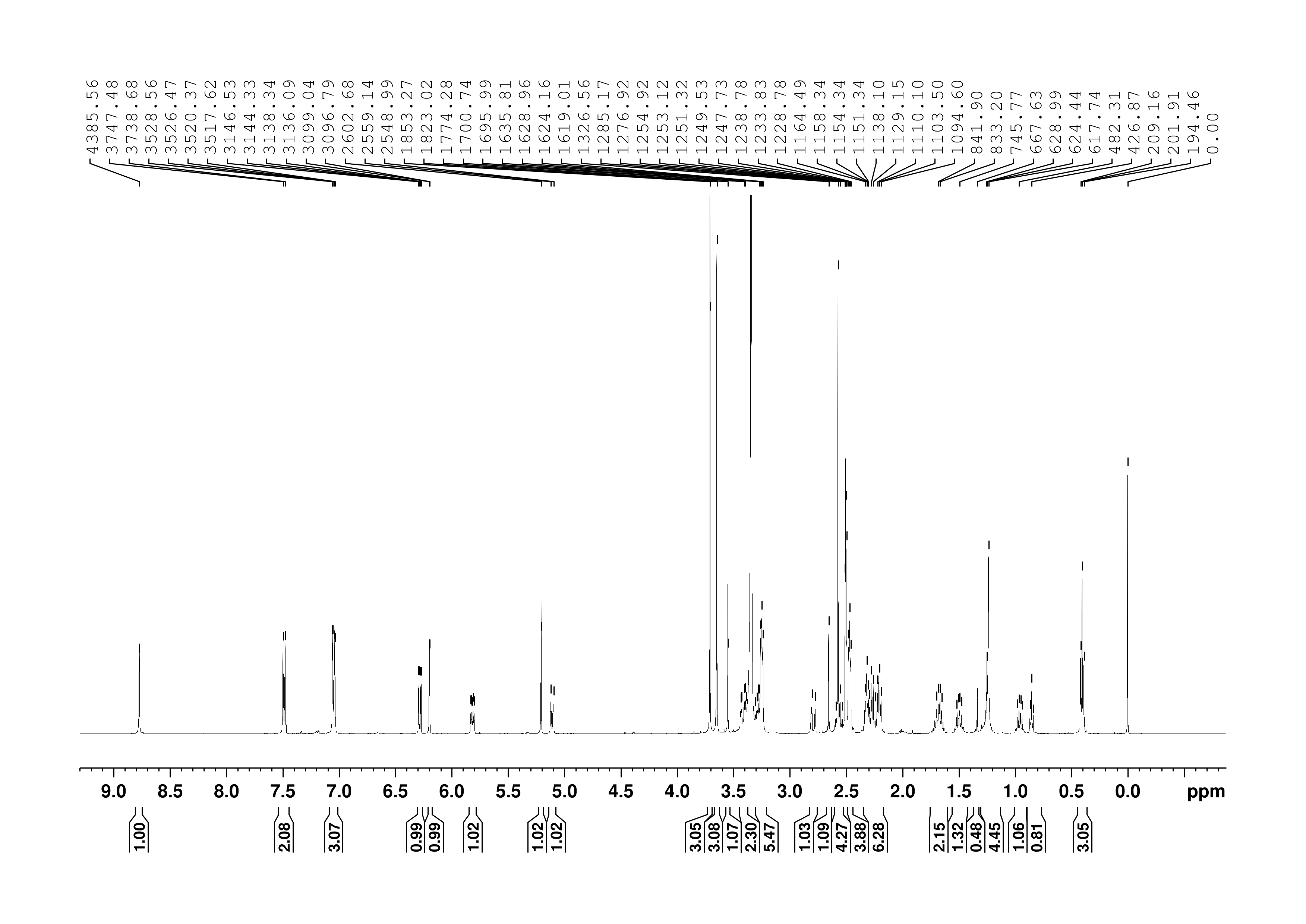


**Figure S50.** ^1^H NMR spectrum of compound **22.**


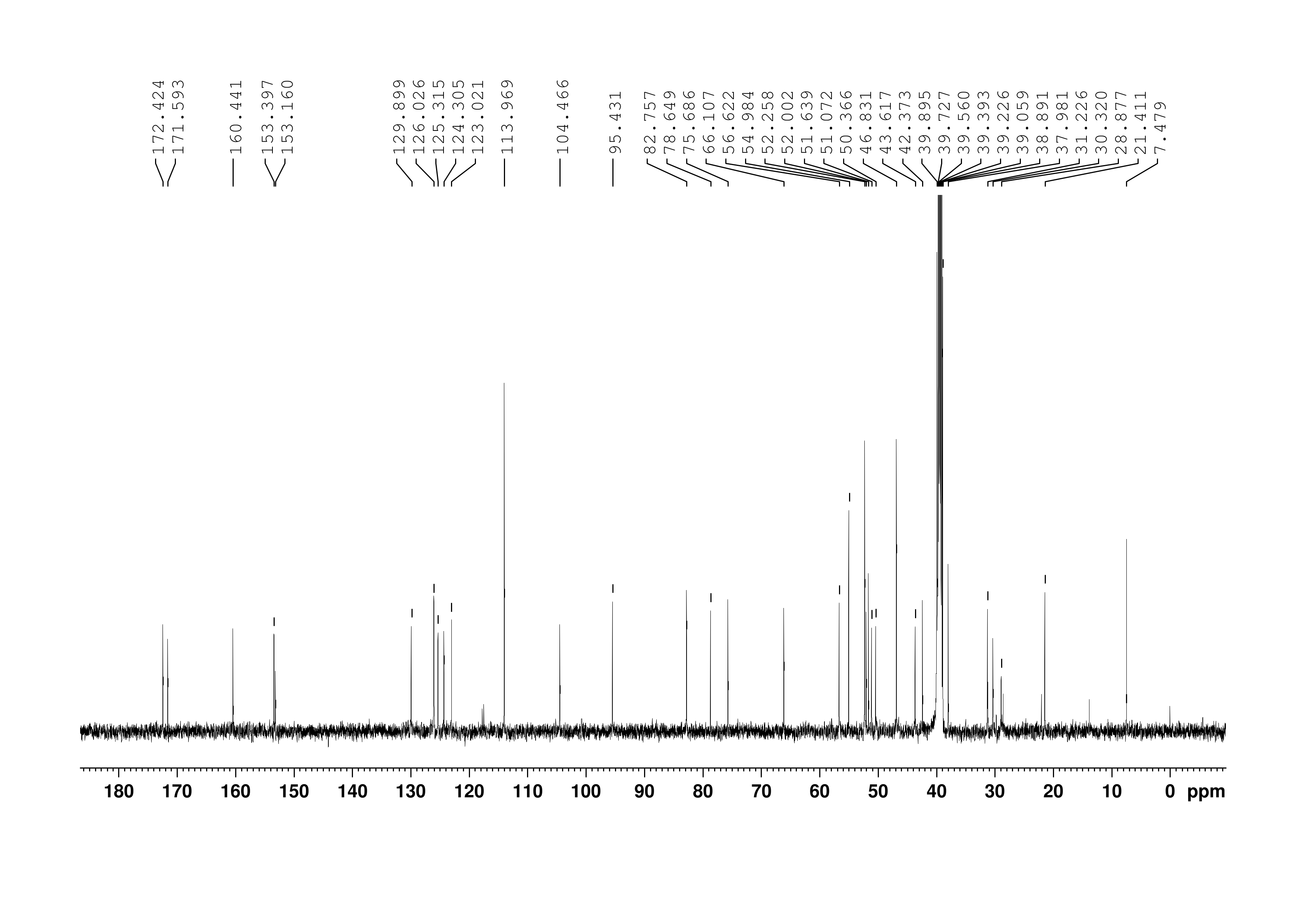


**Figure S51.** ^13^C NMR spectrum of compound **22.**


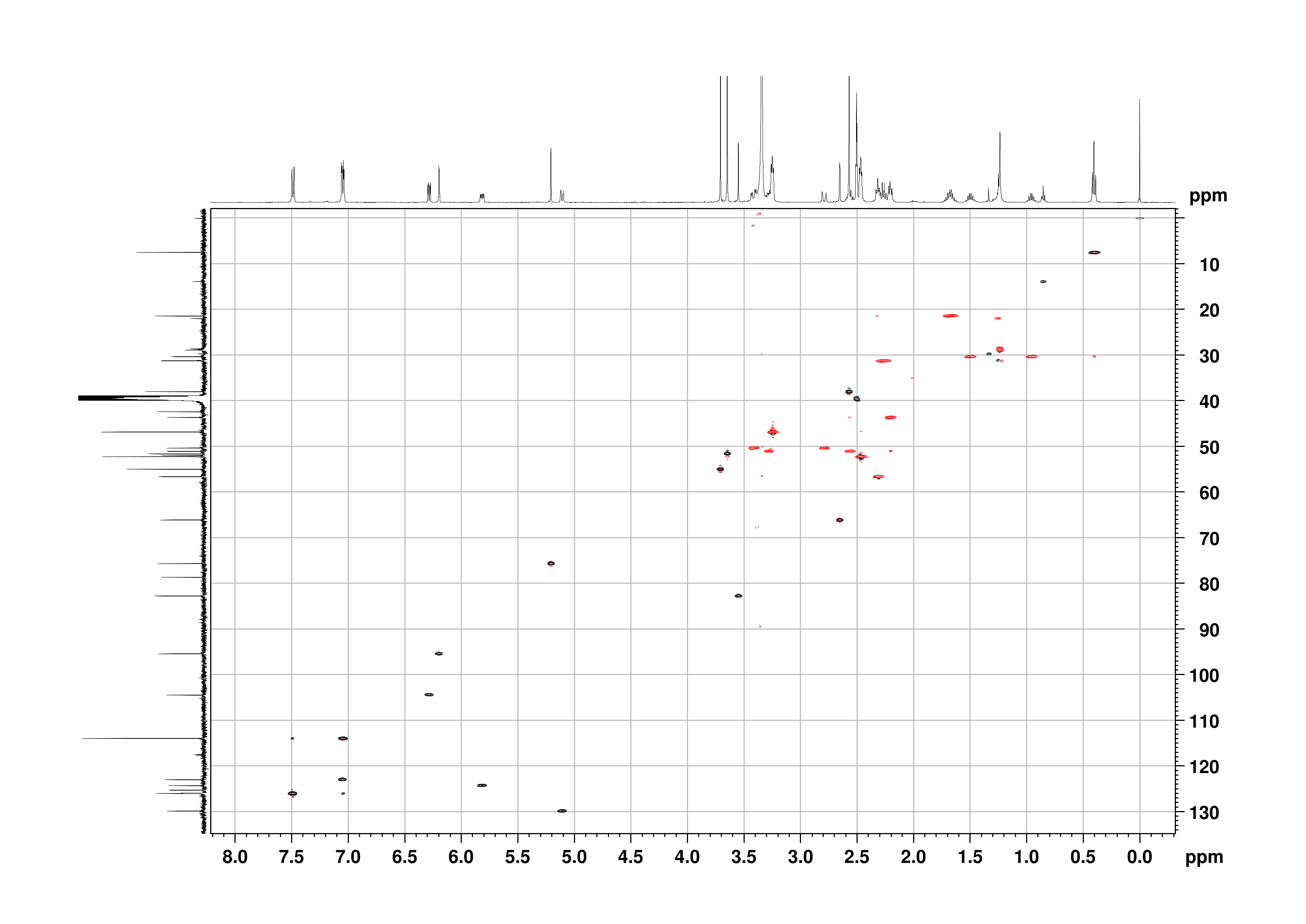


**Figure S52.** HSQC spectrum of compound **22.**


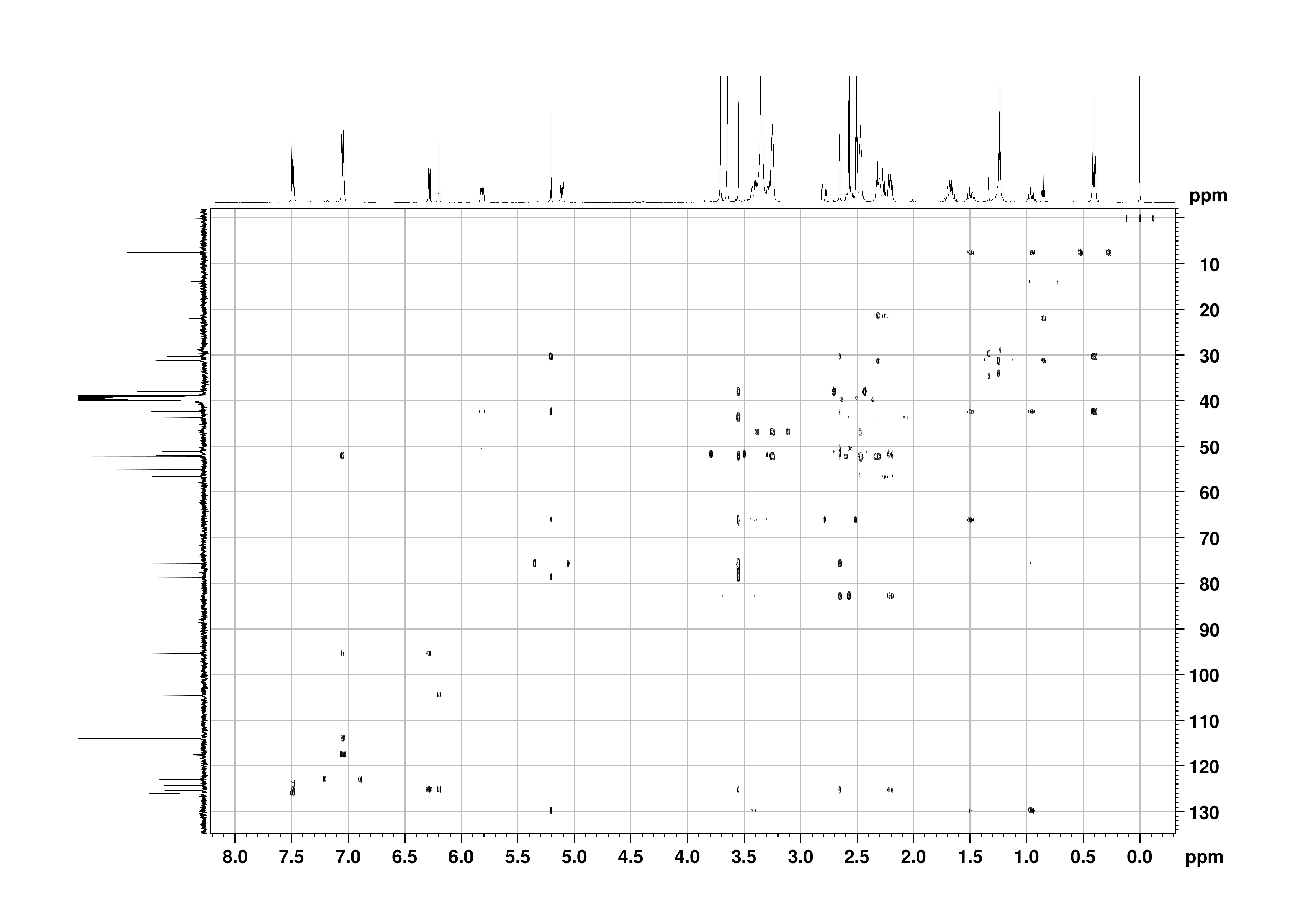


**Figure S53.** ^1^H-^13^C HMBC spectrum of compound **22.**


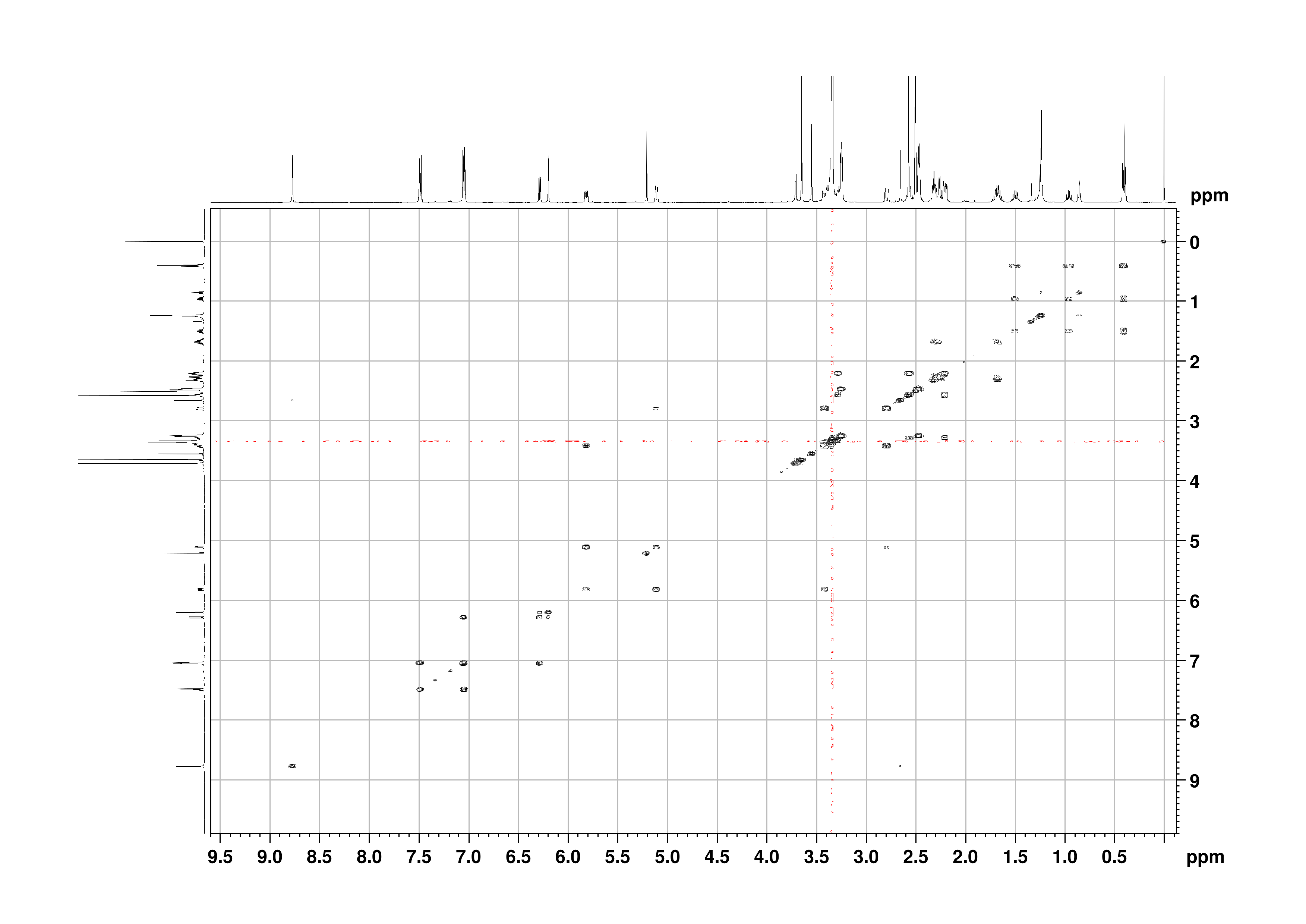


**Figure S54.** COSY spectrum of compound **22.**


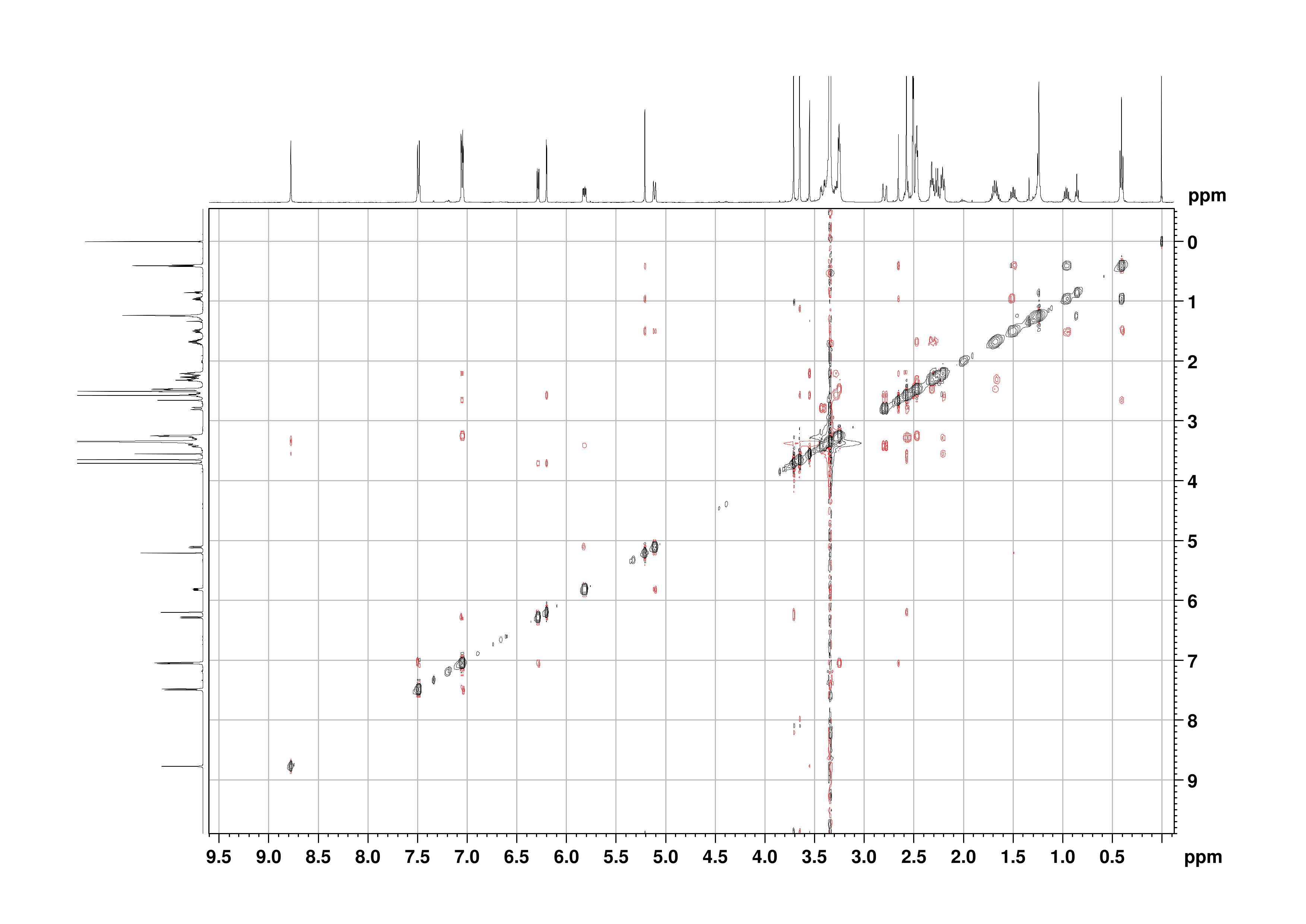


**Figure S55.** ROESY spectrum of compound **22.**

**Figure S56.** HRMS spectrum of compound **22.**

Product **23**

88 mg (68%). M.p.: 72-74 °C. TLC (DCM : MeOH = 15 : 1); *R_f_* = 0.21. IR (KBr) 2945, 2810, 1740, 1617, 1501, 1326, 1245, 1162, 1087, 1017 cm^-1^. ^1^H NMR (799.7 MHz; DMSO-*d*_6_) *δ* (ppm): 0.42 (3H; t; *J* = 7.4 Hz; H_3_-18); 0.95 (1H; dq; *J* = 14.2, 7.3 Hz; H_x_-19); 1.48 (1H; dq; *J* = 14.2, 7.4 Hz; H_y_-19); 1.56-1.67 (2H; m; H_2_-3’); 2.16-2.29 (6H; m; H_2_-6, H_2_-2’, H_2_-4’); 2.36 (8H; br s; H_2_-6’, H_2_-7’, H_2_-9’, H_2_-10’); 2.54-2.59 (4H; m; N(1)-CH_3_, H_x_-5); 2.65 (1H; s; H-21); 2.79 (1H; br d; *J* = 16.2 Hz; H_x_-3); 3.25-3.30 (1H; m; H_y_-5); 3.41 (1H; br dd; *J* = 16.4, 4.8 Hz; H_y_-3); 3.54 (3H; 2×s; H-2, H_2_-11’); 3.63 (3H; s; C(16)-COOCH_3_); 3.70 (3H; s; C(11)-OCH_3_); 5.09 (1H; br d; *J* = 10.1 Hz; H-15); 5.19 (1H; s; H-17); 5.80 (1H; ddd; *J* = 10.1, 5.1, 1.4 Hz; H-14); 6.19 (1H; d; *J* = 2.2 Hz; H-12); 6.28 (1H; dd; *J* = 8.2, 2.2 Hz; H-10); 7.05 (1H; d; *J* = 8.2 Hz; H-9); 7.52 (2H; d; *J* = 8.1 Hz; H-13’, H-17’); 7.68 (2H; d; *J* = 8.1 Hz; H-14’, H-16’); 8.76 (1H; s; C(16)-OH). ^13^C NMR (201.1 MHz; DMSO-*d*_6_) *δ* (ppm): 7.5 (C-18); 21.5 (C-3’) 30.3 (C-19); 31.1 (C-2’); 38.0 (N(1)-CH_3_); 42.4 (C-20); 43.6 (C-6); 50.4 (C-3); 51.1 (C-5); 51.6 (C(16)-COOCH_3_); 52.0 (C-7); 52.5 (C-6’, C-7’, C-9’, C-10’); 55.0 (C(11)-OCH_3_); 56.7 (C-4’); 61.2 (C-11’); 66.1 (C-21); 75.7 (C-17); 78.6 (C-16); 82.8 (C-2); 95.4 (C-12); 104.5 (C-10); 123.0 (C-9); 124.3 (C-14); 124.9 (q; *J* = 3.7 Hz; C-14’, C-16’); 125.3 (C-8); 129.3 (C-13’, C-17’); 129.9 (C-15); 153.4 (C-13); 160.5 (C-11); 171.6 (C(16)-COOCH_3_) 172.4 (C-1’). HRMS: M+H=727.36733 (delta = -0.5 ppm; C_39_H_50_O_6_N_4_F_3_).

**Figure S57.** The skeleton numbering of compound **23** used for NMR assignment.


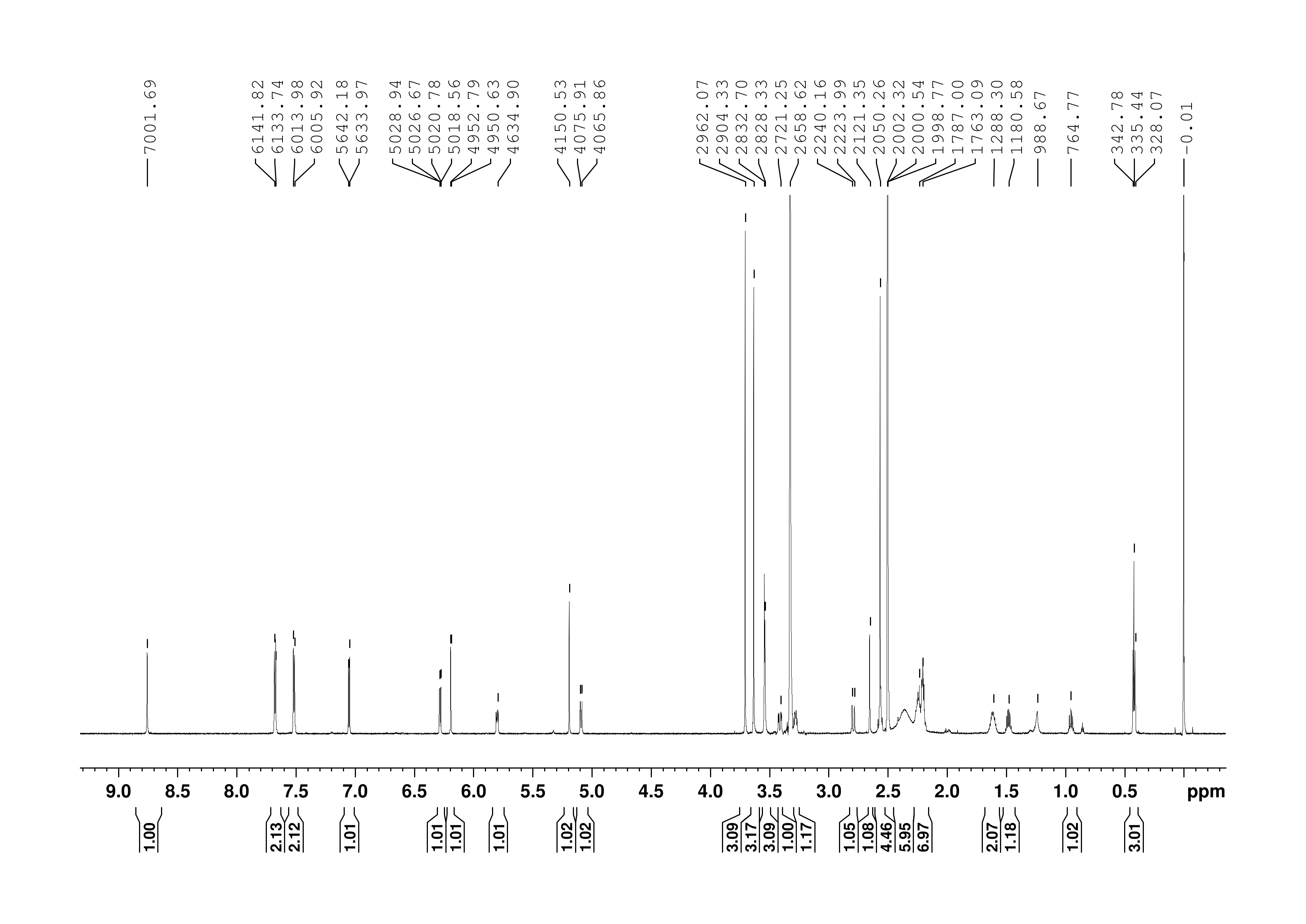


**Figure S58.** ^1^H NMR spectrum of compound **23.**


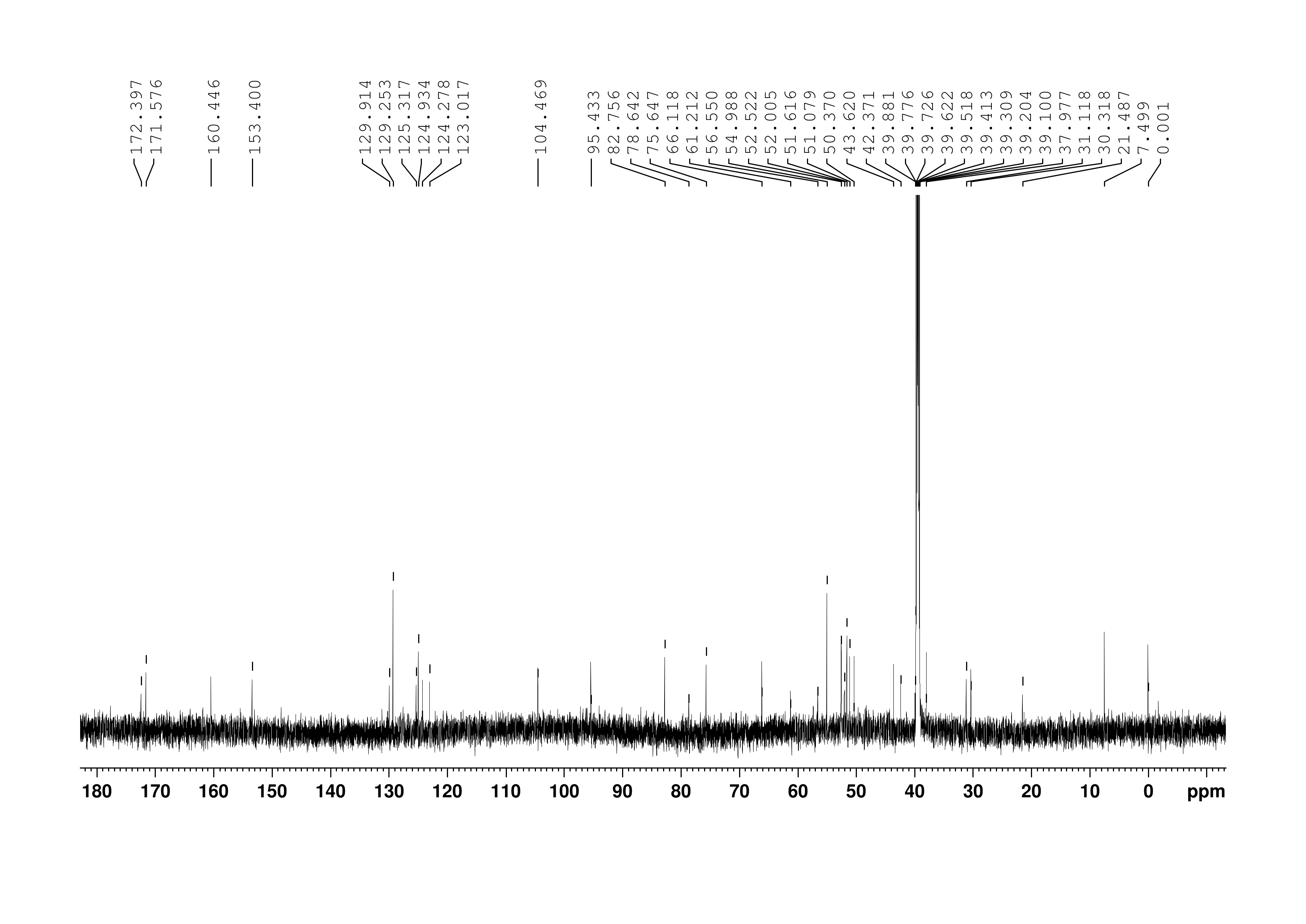


**Figure S59.** ^13^C NMR spectrum of compound **23.**


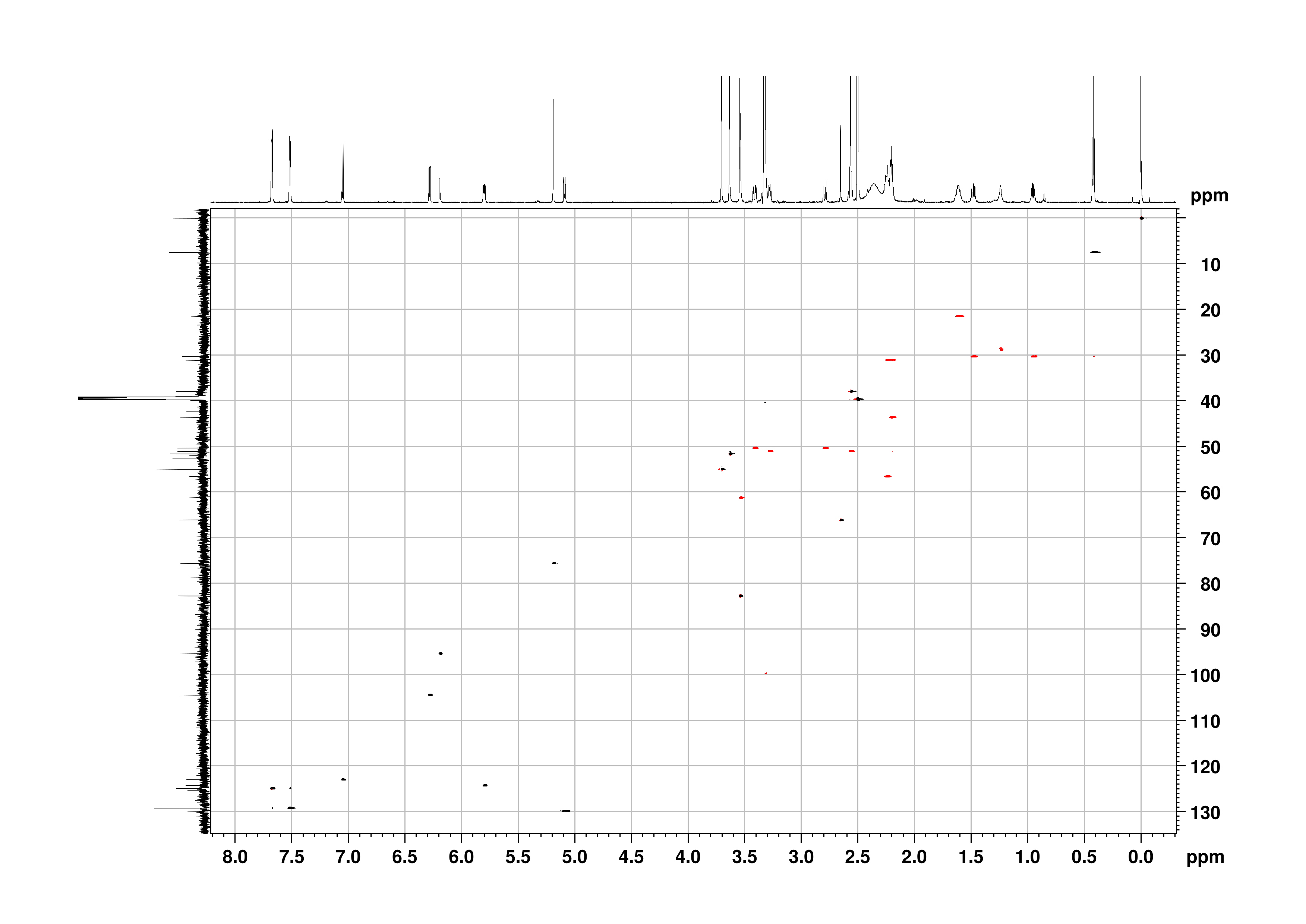


**Figure S60.** HSQC spectrum of compound **23.**


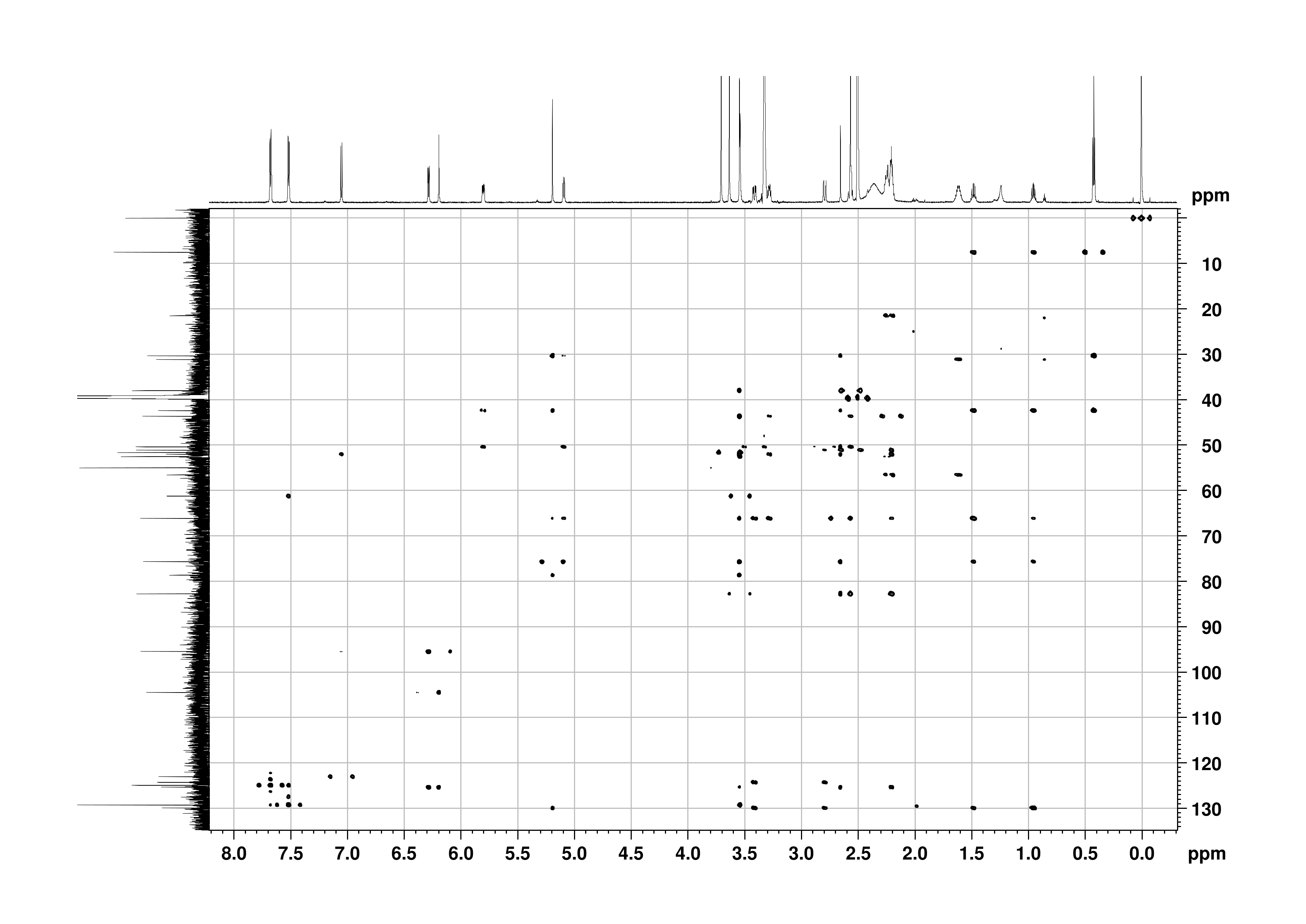


**Figure S61.** ^1^H-^13^C HMBC spectrum of compound **23.**


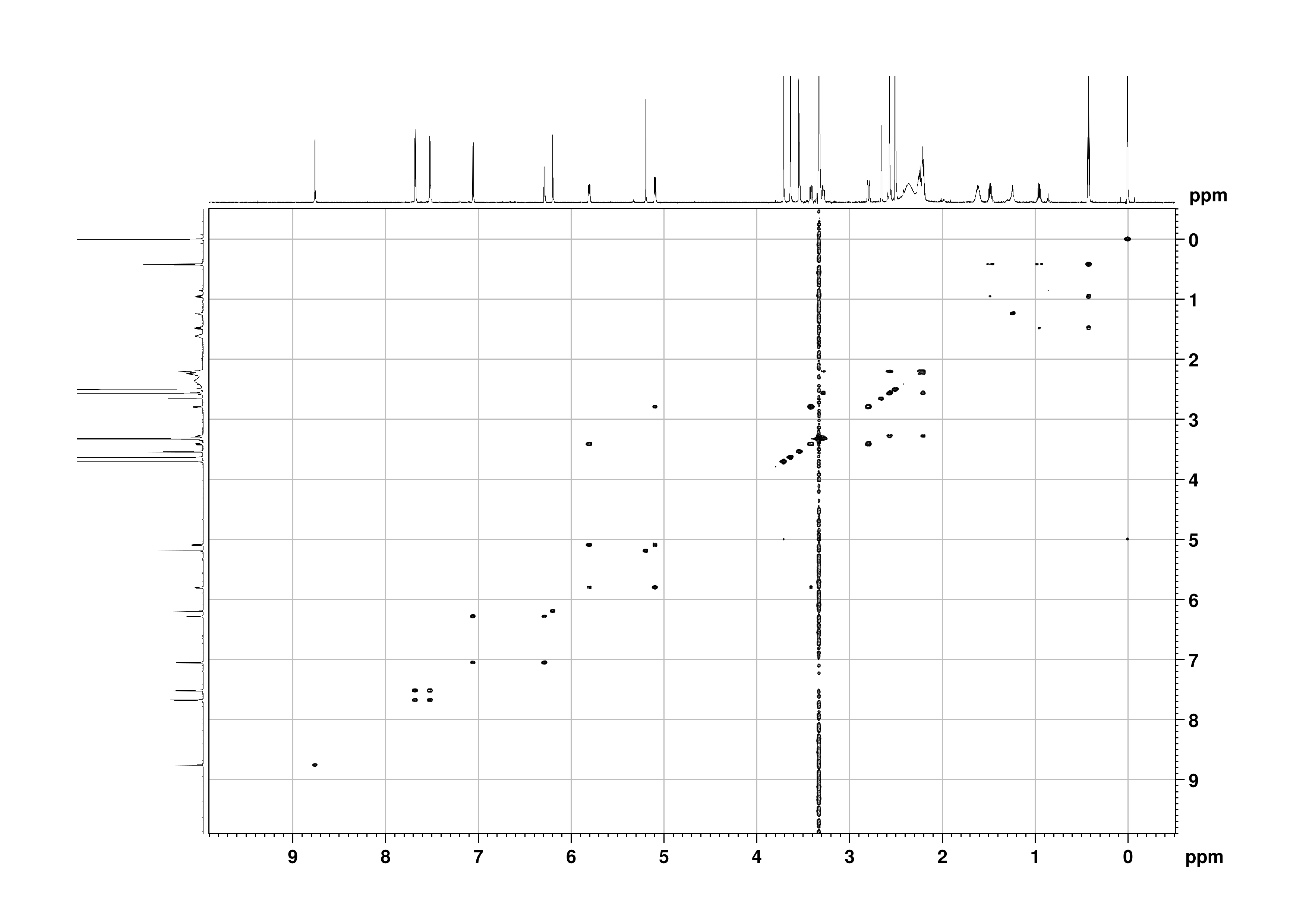


**Figure S62.** COSY spectrum of compound **23.**


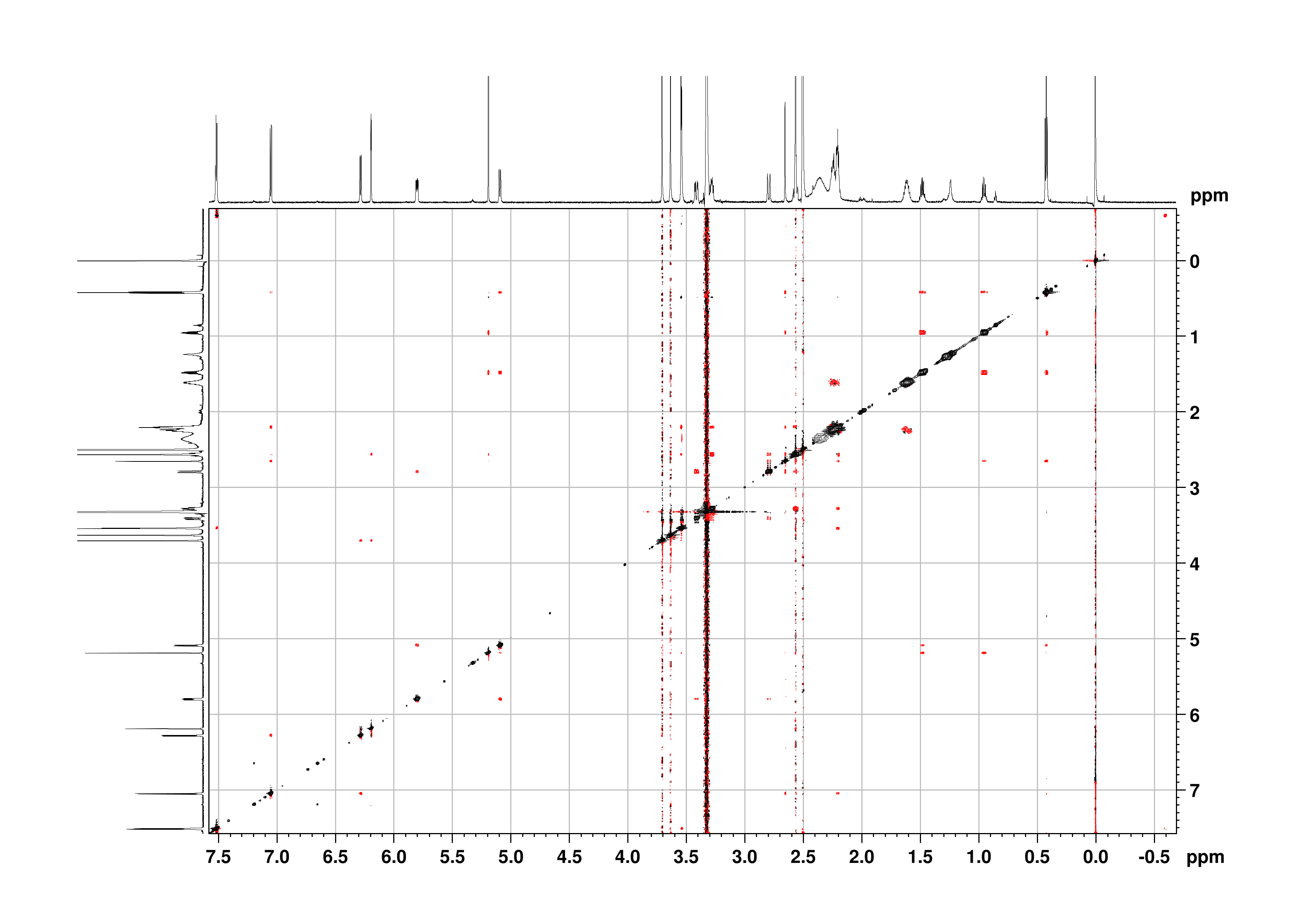


**Figure S63.** ROESY spectrum of compound **23.**

**Figure S64.** HRMS spectrum of compound **23**.

Product **24**

99 mg (82%). M.p.: 165-167 °C. TLC (DCM : MeOH = 15 : 1); *R_f_* = 0.26. IR (KBr) 2949, 2807, 1735, 1616, 1497, 1255, 1217, 1120 cm^-1^. ^1^H NMR (499.9 MHz; DMSO-*d*_6_) *δ* (ppm): 0.42 (3H; t; *J* = 7.4 Hz; H_3_-18); 0.95 (1H; dq; *J* = 14.2, 7.3 Hz; H_x_-19); 1.48 (1H; dq; *J* = 14.2, 7.4 Hz; H_y_-19); 1.60-1.70 (2H; m; H_2_-3’); 2.15-2.29 (6H; m; H_2_-6, H_2_-2’, H_2_-4’); 2.33 (8H; br s; H_2_-6’, H_2_-7’, H_2_-9’, H_2_-10’); 2.54-2.59 (4H; m; N(1)-CH_3_, H_x_-5); 2.65 (1H; s; H-21); 2.79 (1H; br d; *J* = 16.4 Hz; H_x_-3); 3.23-3.30 (1H; m; H_y_-5); 3.38-3.45 (3H; m; H_y_-3, H_2_-11'); 3.54 (1H; s; H-2); 3.63 (3H; s; C(16)-COOCH_3_); 3.70 (3H; s; C(11)-OCH_3_); 5.09 (1H; br d; *J* = 10.1 Hz; H-15); 5.19 (1H; s; H-17); 5.80 (1H; ddd; *J* = 10.2, 4.8, 1.3 Hz; H-14); 6.19 (1H; d; *J* = 2.2 Hz; H-12); 6.28 (1H; dd; *J* = 8.2, 2.2 Hz; H-10); 7.05 (1H; d; *J* = 8.2 Hz; H-9); 7.09-7.15 (2H; m; H-14’, H-16’); 7.27-7.33 (2H; m; H-13’, H-17’); 8.75 (1H; s; C(16)-OH). ^13^C NMR (125.7 MHz; DMSO-*d*_6_) *δ* (ppm): 7.5 (C-18); 21.5 (C-3’); 30.3 (C-19); 31.1 (C-2’); 38.0 (N(1)-CH_3_); 42.4 (C-20); 43.6 (C-6); 50.4 (C-3); 51.1 (C-5); 51.6 (C(16)-COOCH_3_); 52.0 (C-7); 52.4 (C-6’, C-10’); 52.6 (C-7’, C-9’); 55.0 (C(11)-OCH_3_); 56.6 (C-4’); 61.0 (C-11’); 66.1 (C-21); 75.6 (C-17); 78.6 (C-16); 82.8 (C-2); 95.4 (C-12); 104.5 (C-10); 114.7 (d; *J* = 30.0 Hz; C-14’, C-16’); 123.0 (C-9); 124.3 (C-14); 125.3 (C-8); 129.9 (C-15); 130.5 (d; *J* = 8.0 Hz; C-13’, C-17’); 134.3 (d; *J* = 2.8 Hz; C-12’); 153.4 (C-13); 160.4 (C-11); 161.1 (d; *J* = 242.2 Hz; C-15’); 171.6 (C(16)-COOCH_3_); 172.4 (C-1’). HRMS: M+H=677.37089 (delta = -0.4 ppm; C_38_H_50_O_6_N_4_F).

**Figure S65.** The skeleton numbering of compound **24** used for NMR assignment.


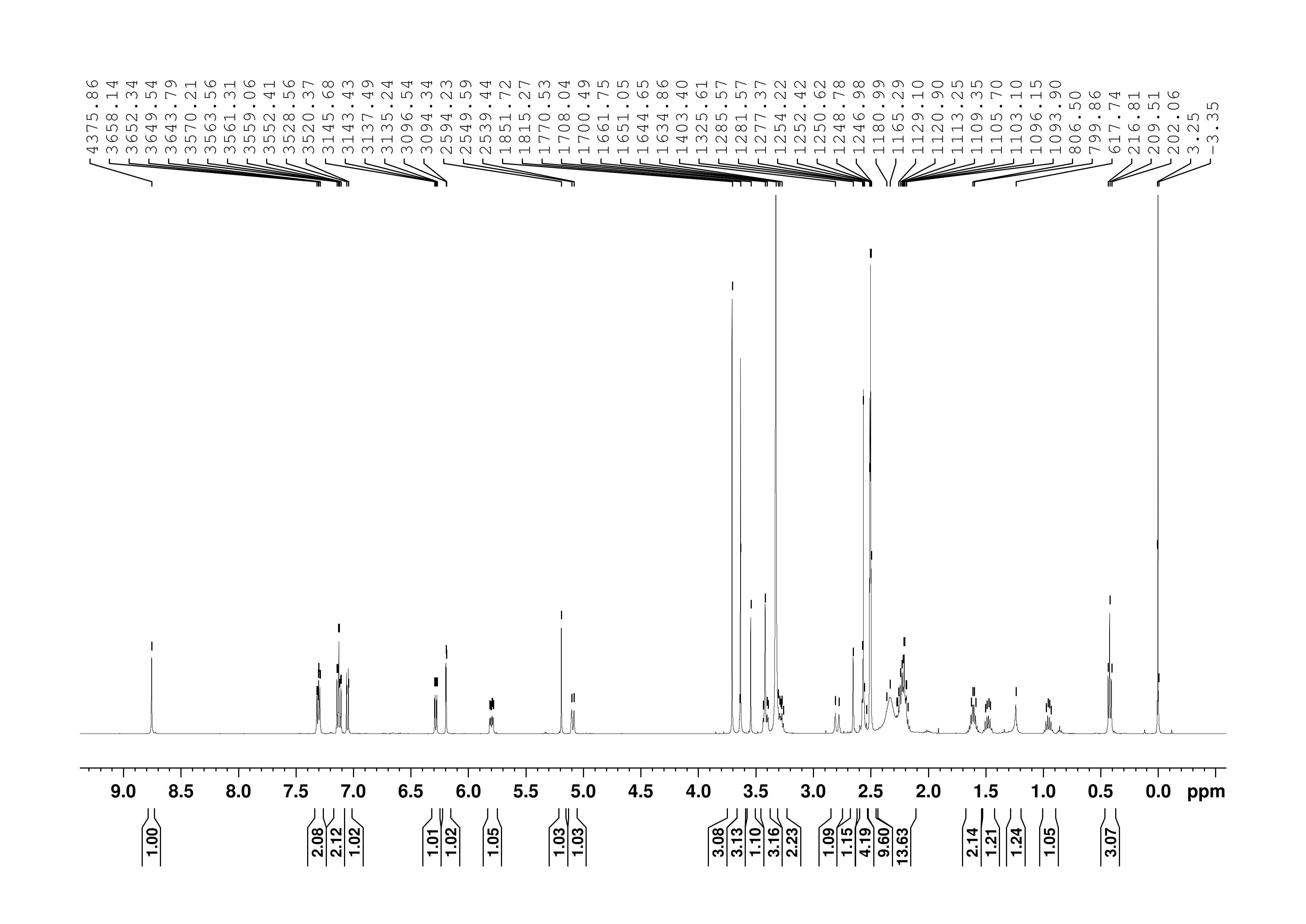


**Figure S66.** ^1^H NMR spectrum of compound **24.**


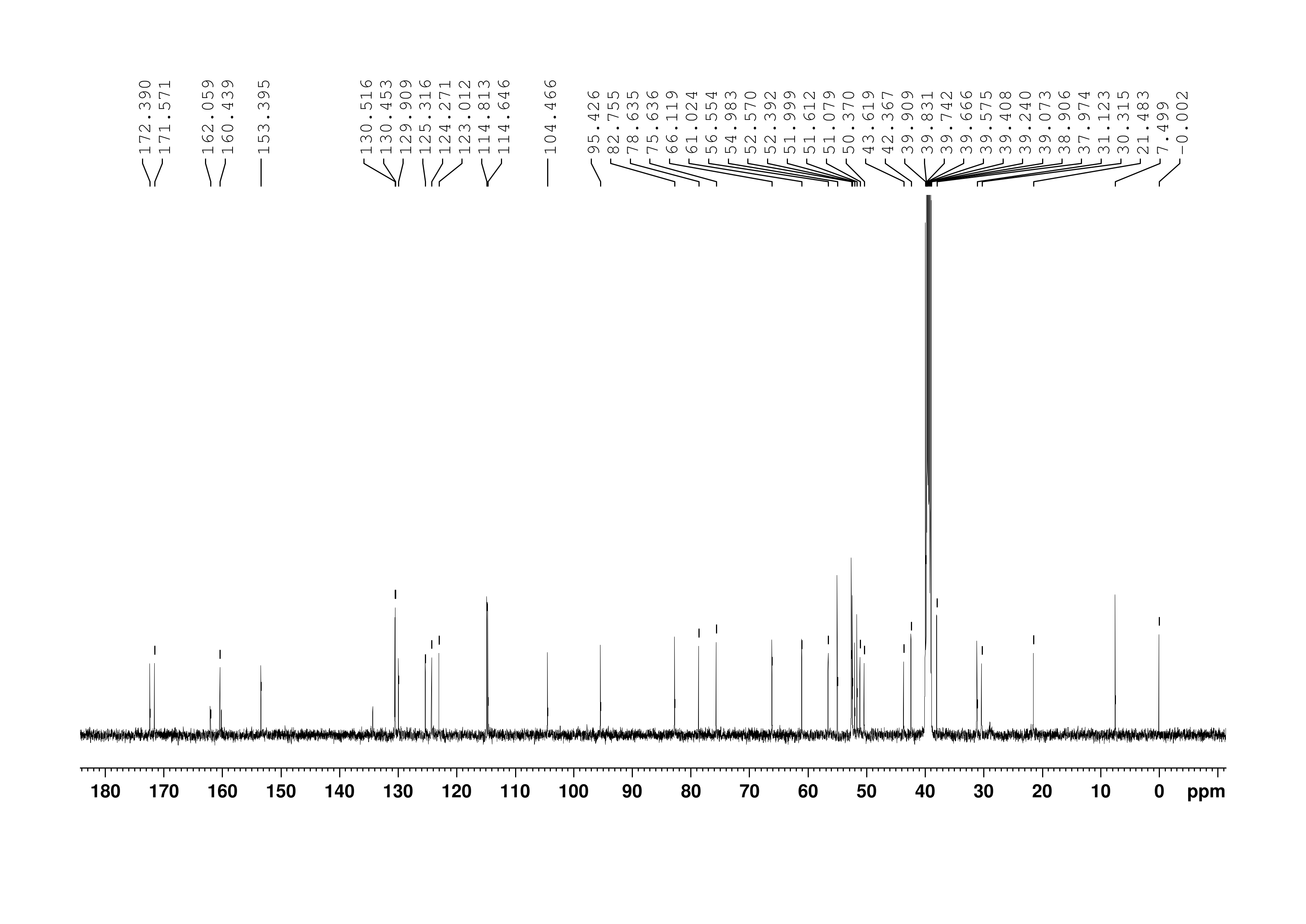


**Figure S67.** ^13^C NMR spectrum of compound **24.**


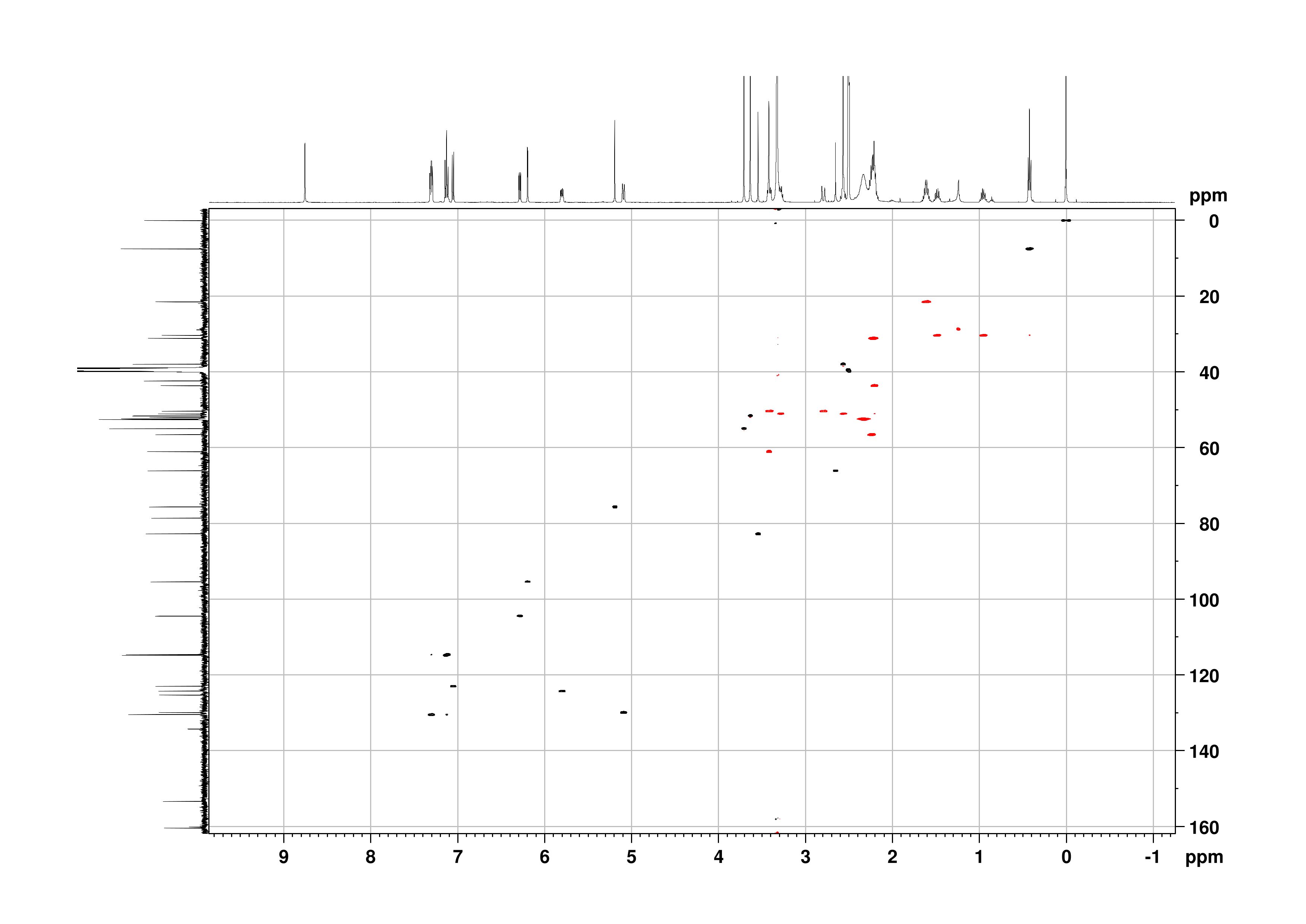


**Figure S68.** HSQC spectrum of compound **24.**


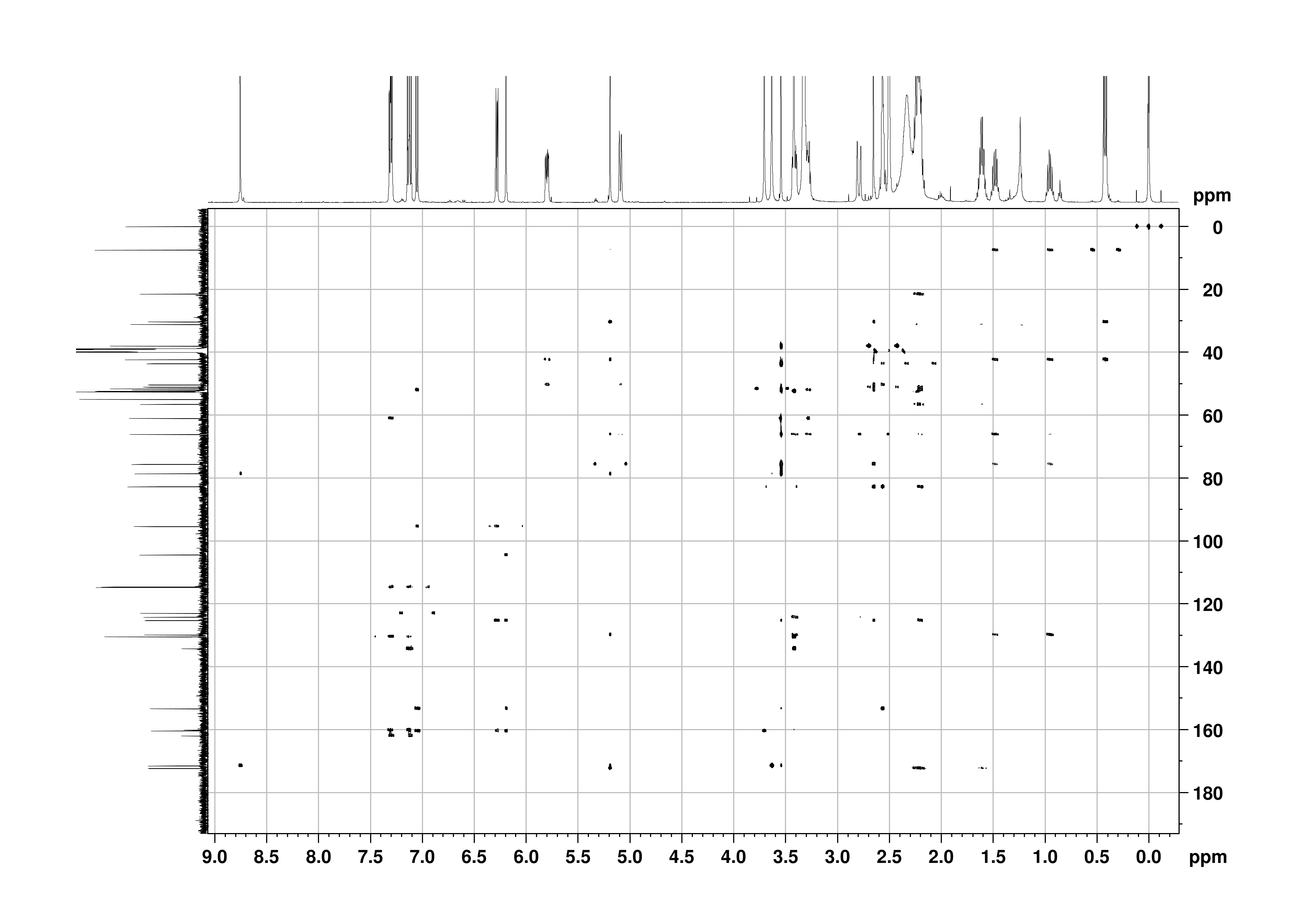


**Figure S69.** ^1^H-^13^C HMBC spectrum of compound **24.**


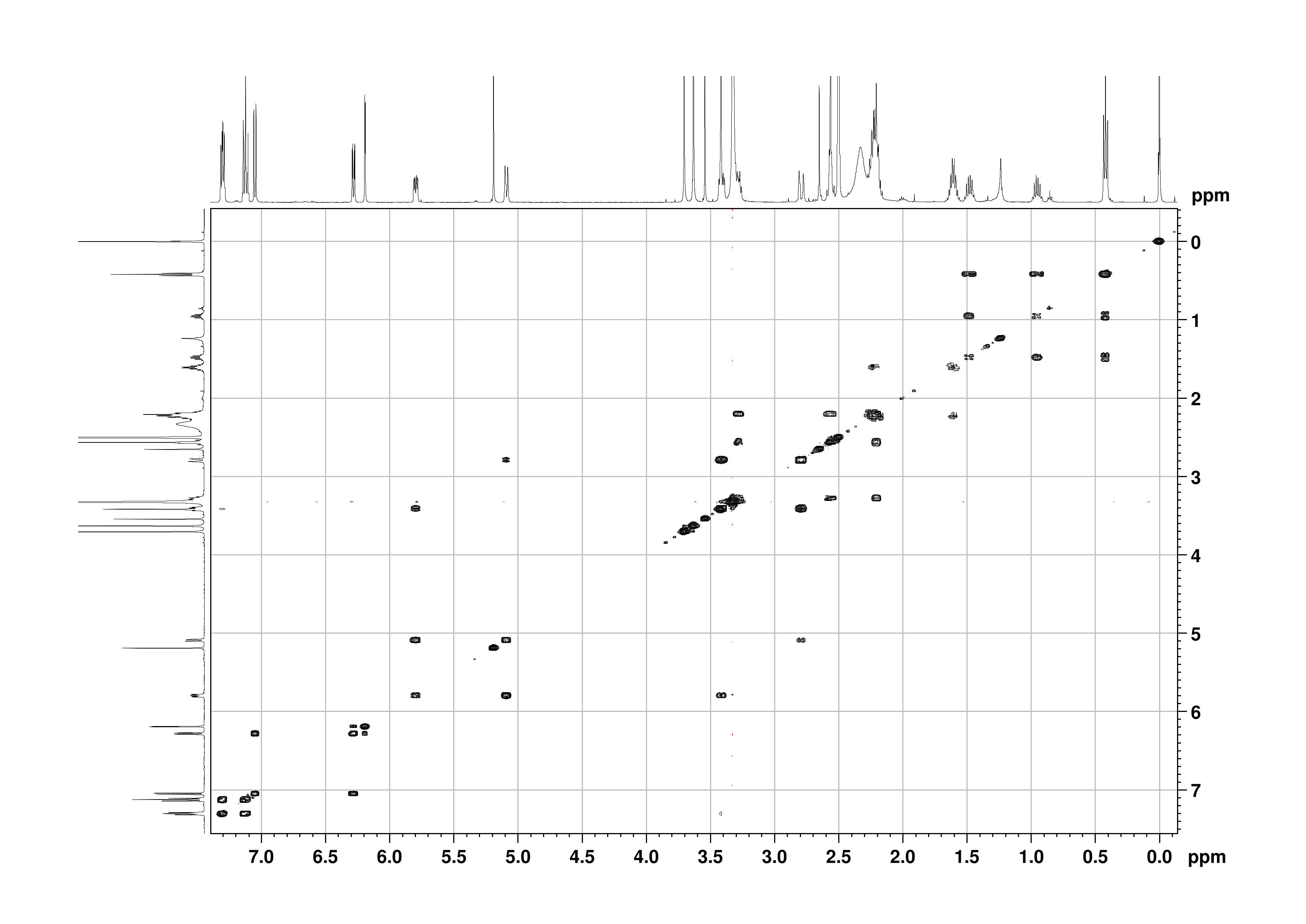


**Figure S70.** COSY spectrum of compound **24.**


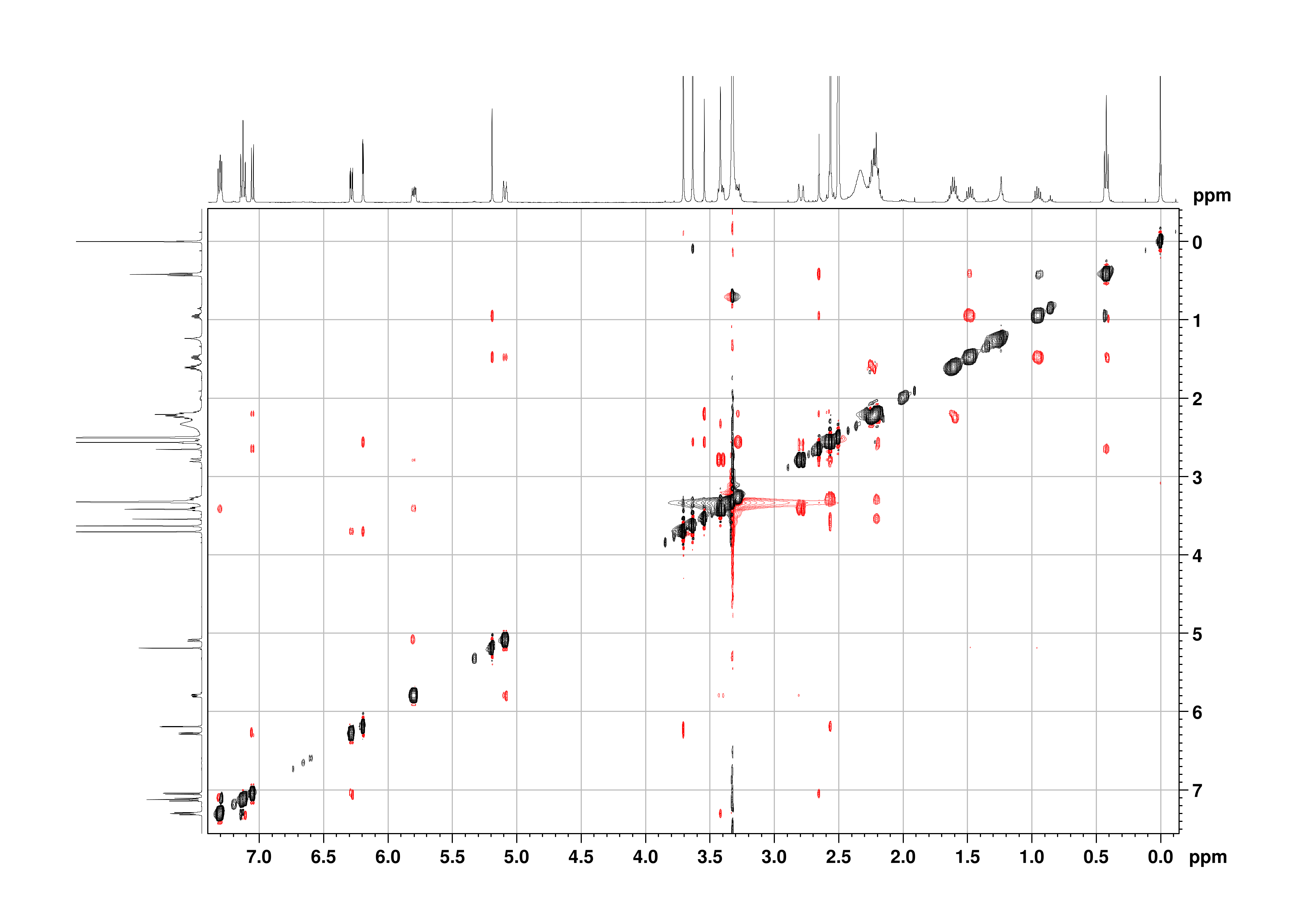


**Figure S71.** ROESY spectrum of compound **24.**

**Figure S72.** HRMS spectrum of compound **24.**

Product **25**

73 mg (53%). M.p.: 95-97 °C. TLC (DCM : MeOH = 15 : 1); *R_f_* = 0.41. IR (KBr) 3436, 2957, 2809, 1740, 1602, 1504, 1223, 1088, 826 cm^-1^. ^1^H NMR (799.7 MHz; DMSO-*d*_6_) *δ* (ppm): 0.41 (3H; t; *J* = 7.4 Hz; H_3_-18); 0.94 (1H; dq; *J* = 14.2, 7.3 Hz; H_x_-19); 1.47 (1H; dq; *J* = 14.2, 7.4 Hz; H_y_-19); 1.55-1.64 (2H; m; H_2_-3’); 2.15-2.28 (6H; m; H_2_-6, H_2_-2’, H_2_-4’); 2.33 (8H; br s; H_2_-6’, H_2_-7’, H_2_-9’, H_2_-10’); 2.54-2.58 (4H; m; N(1)-CH_3_, H_x_-5); 2.64 (1H; s; H-21); 2.78 (1H; br d; *J* = 16.4 Hz; H_x_-3); 3.25-3.39 (1H; m; H_y_-5); 3.37-3.42 (1H; m; H_y_-3); 3.53 (1H; s; H-2); 3.62 (3H; s; C(16)-COOCH_3_); 3.70 (3H; s; C(11)-OCH_3_); 4.32 (1H; s; H-11’); 5.07 (1H; dt; *J* = 10.1, 2.1 Hz; H-15); 5.18 (1H; s; H-17); 5.78 (1H; ddd; *J* = 10.2, 4.9, 1.5 Hz; H-14); 6.19 (1H; d; *J* = 2.2 Hz; H-12); 6.28 (1H; dd; *J* = 8.2, 2.3 Hz; H-10); 7.05 (1H; d; *J* = 8.2 Hz; H-9); 7.11 (4H; ~td; *J* = 8.8, 1.2 Hz; 4×C(11’)-PhF: H_meta_); 7.39-7.43 (4H; m; 4×C(11’)-PhF: H_orto_); 8.76 (1H; s; C(16)-OH). ^13^C NMR (201.1 MHz; DMSO-*d*_6_) *δ* (ppm): 7.5 (C-18); 21.5 (C-3’); 30.3 (C-19); 31.1 (C-2’); 38.0 (N(1)-CH_3_); 42.4 (C-20); 43.6 (C-6); 50.4 (C-3); 51.1 (C-5); 51.2 (C-7’, C-9’); 51.6 (C(16)-COOCH_3_); 52.0 (C-7); 52.7 (C-6’, C-10’); 55.0 (C(11)-OCH_3_); 56.5 (C-4’); 66.1 (C-21); 73.0 (C-11’); 75.6 (C-17); 78.6 (C-16); 82.8 (C-2); 95.4 (C-12); 104.5 (C-10); 115.2 (d; *J* = 21.0 Hz; 4×C(11’)-PhF: C_meta_); 123.0 (C-9); 124.3 (C-14); 125.3 (C-8); 129.2 (d; *J* = 7.7 Hz; 4×C(11’)-PhF: C_orto_); 129.9 (C-15); 138.7 (2×C(11’)-PhF: C_ipszo_); 153.4 (C-13); 160.4 (C-11); 160.9 (d; *J* = 243.0 Hz; 2×C(11’)-PhF: C_para_); 171.6 (C(16)-COOCH_3_); 172.4 (C-1’). HRMS: M+H=771.39277; (delta = -2.5 ppm; C_44_H_53_O_6_N_4_F_2_).

**Figure S73.** The skeleton numbering of compound **25** used for NMR assignment.


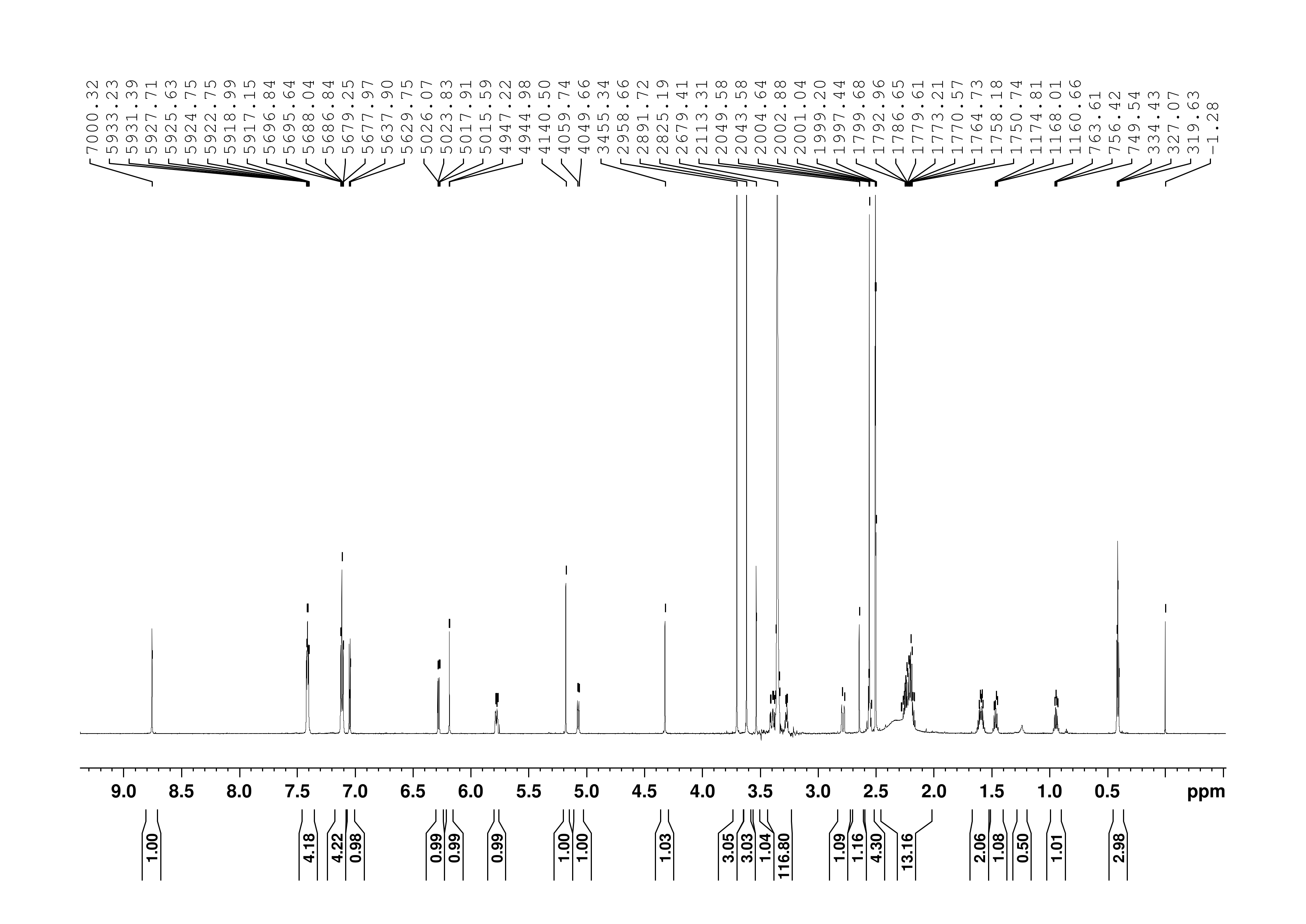


**Figure S74.** ^1^H NMR spectrum of compound **25.**


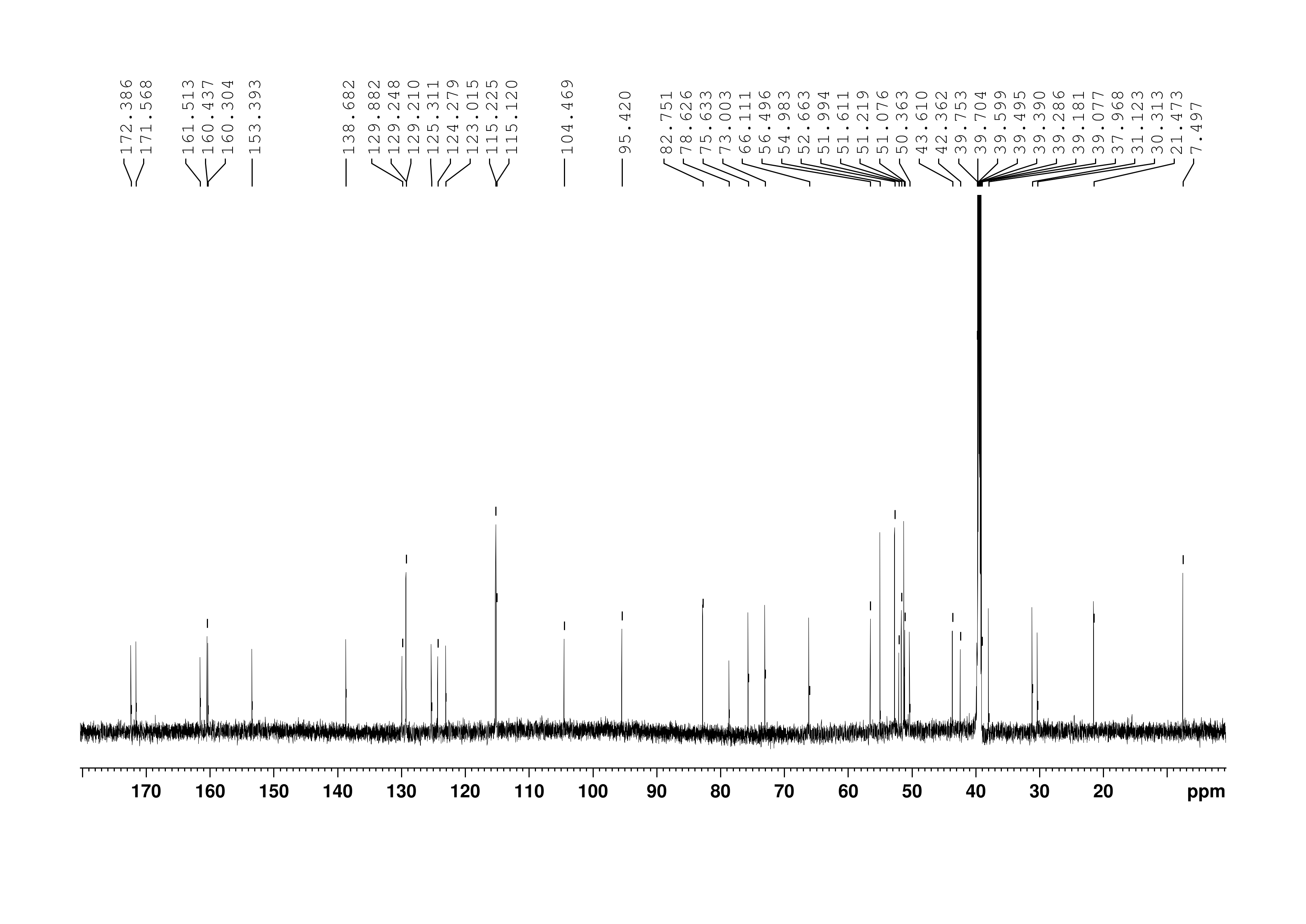


**Figure S75.** ^13^C NMR spectrum of compound **25.**


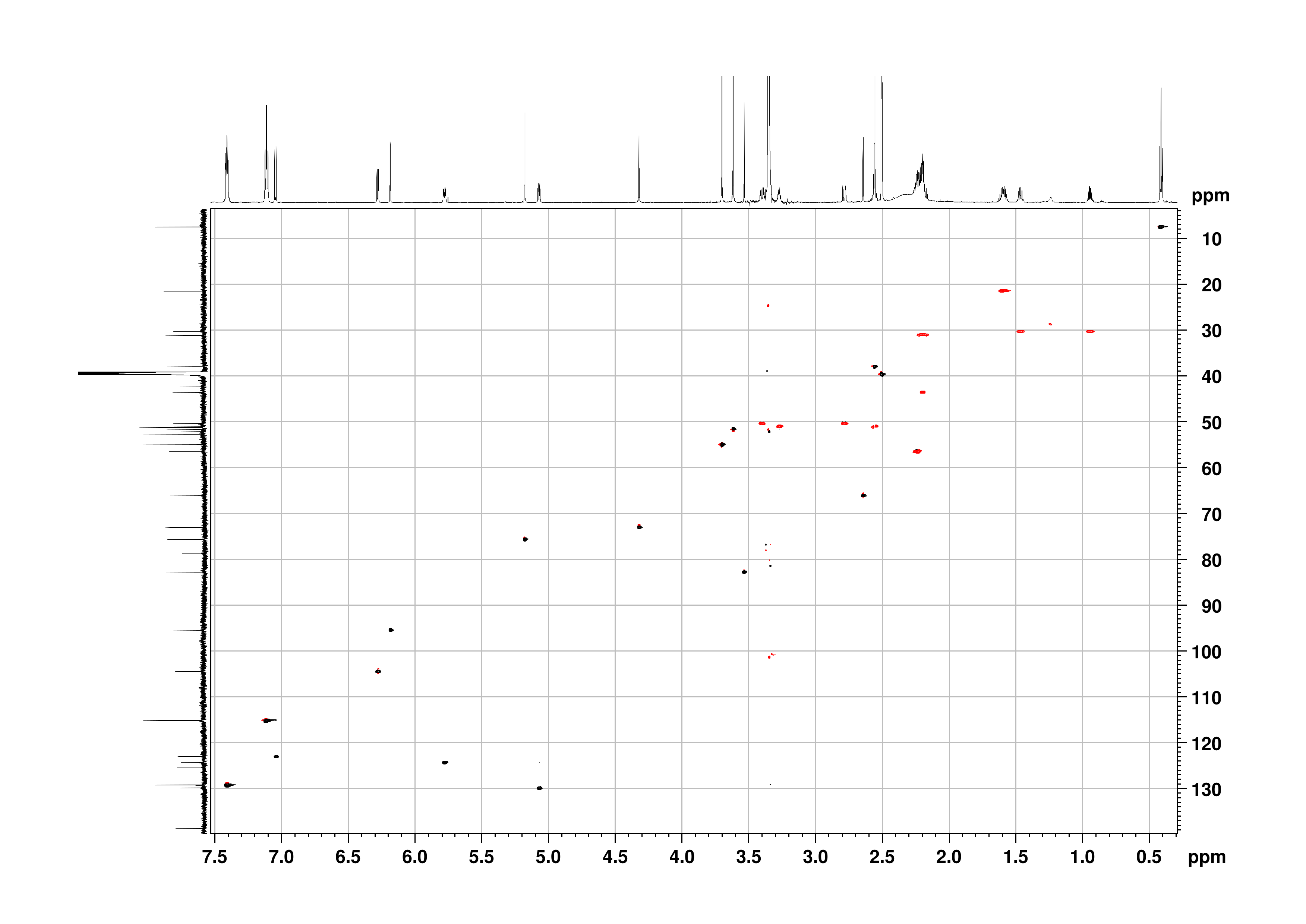


**Figure S76.** HSQC spectrum of compound **25.**


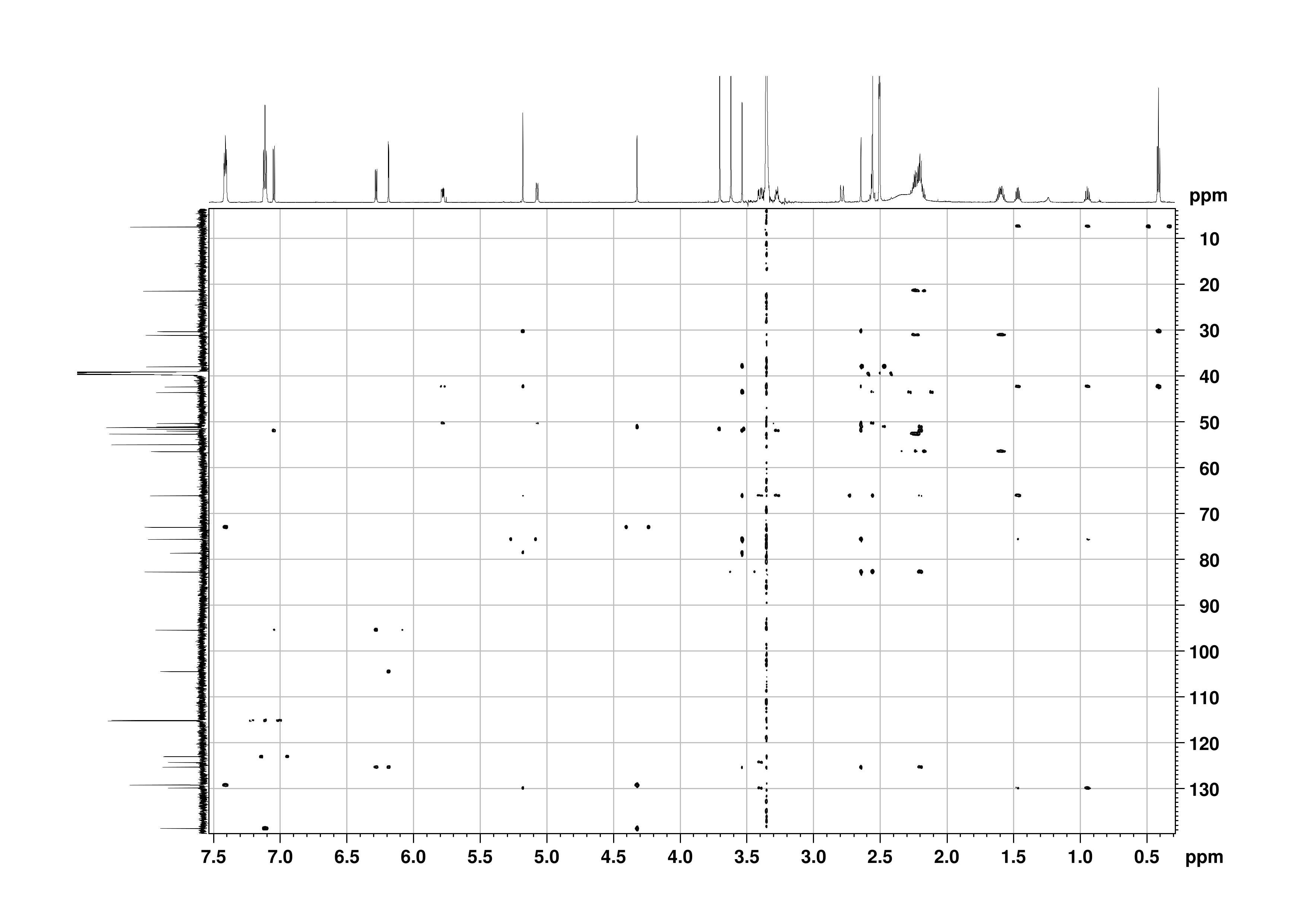


**Figure S77.** ^1^H-^13^C HMBC spectrum of compound **25.**


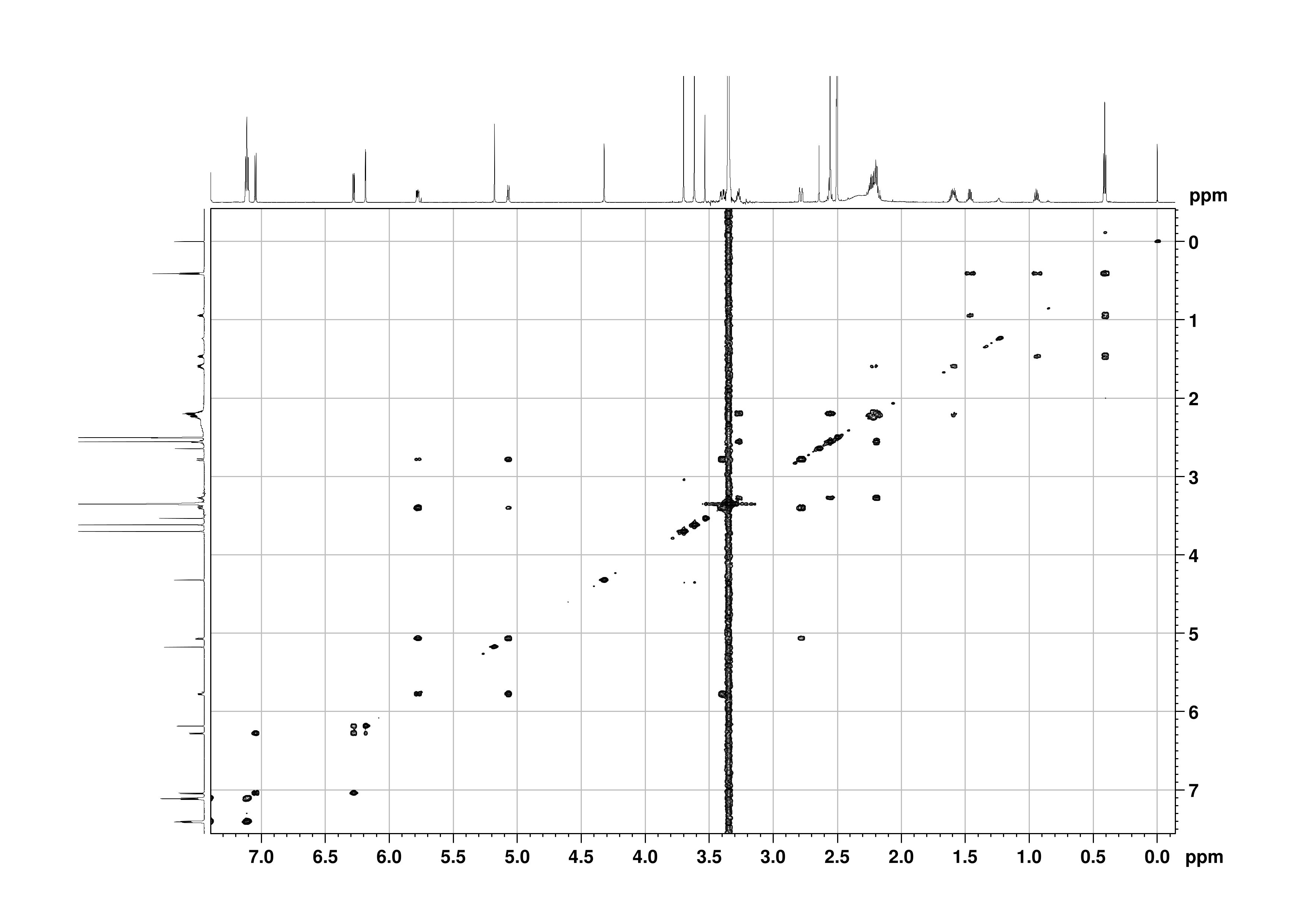


**Figure S78.** COSY spectrum of compound **25.**


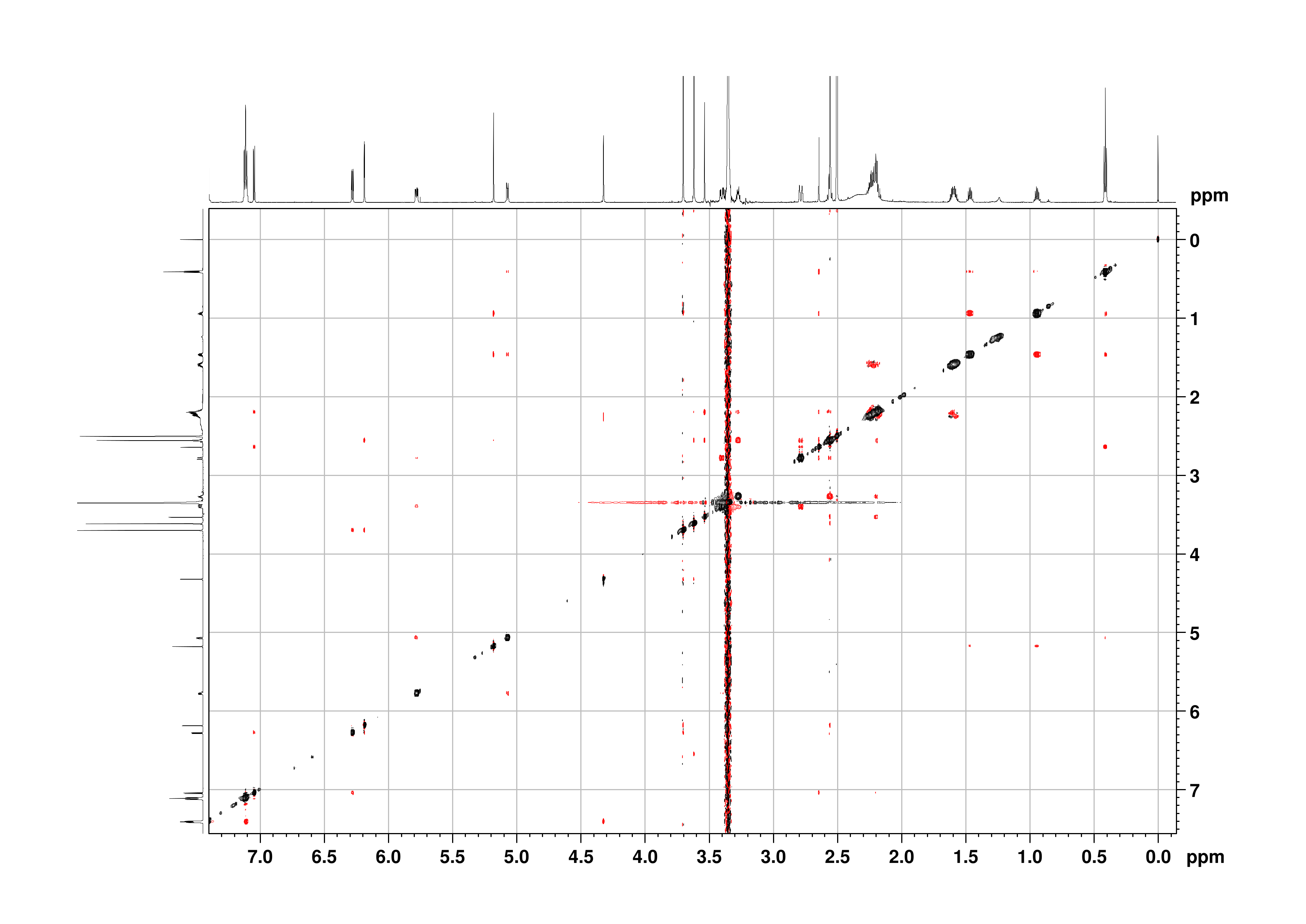


**Figure S79.** ROESY spectrum of compound **25.**

**Figure S80.** HRMS spectrum of compound **25.**

Product **26**

87 mg (74%). M.p.: 60-62 °C. TLC (DCM : MeOH = 15 : 1); *R_f_* = 0.48. IR (KBr) 3459, 2947, 2809, 1738, 1619, 1501, 1433, 1246, 1137, 1023 cm^-1^. ^1^H NMR (499.9 MHz; DMSO-*d*_6_) *δ* (ppm): 0.42 (3H; t; *J* = 7.4 Hz; H_3_-18); 0.96 (1H; dq; *J* = 14.2, 7.3 Hz; H_x_-19); 1.49 (1H; dq; *J* = 14.2, 7.4 Hz; H_y_-19); 1.60-1.70 (2H; m; H_2_-3’); 2.17-2.34 (6H; m; H_2_-6, H_2_-2’, H_2_-4’); 2.37 (4H; ~t; *J* = 5.0 Hz; H_2_-6’, H_2_-10’); 2.53-2.60 (4H; m; N(1)-CH_3_, H_x_-5); 2.65 (1H; s; H-21); 2.79 (1H; br d; *J* = 16.4 Hz; H_x_-3); 3.24-3.30 (1H; m; H_y_-5); 3.42 (1H; dd; *J* = 16.4, 4.9 Hz; H_y_-3); 3.55 (1H; s; H-2); 3.63 (4H; br s; H_2_-7’, H_2_-9’); 3.65 (3H; s; C(16)-COOCH_3_); 3.71 (3H; s; C(11)-OCH_3_); 5.09 (1H; br d; *J* = 10.1 Hz; H-15); 5.20 (1H; s; H-17); 5.83 (1H; ddd; *J* = 10.2, 4.8, 1.3 Hz; H-14); 6.20 (1H; d; *J* = 2.2 Hz; H-12); 6.28 (1H; dd; *J* = 8.2, 2.2 Hz; H-10); 6.61 (1H; dd; *J* = 3.5, 1.8 Hz; H-15’); 6.97 (1H; dd; *J* = 3.5, 0.7 Hz; H-16’); 7.05 (1H; d; *J* = 8.2 Hz; H-9); 7.82 (1H; dd; *J* = 1.7, 0.8 Hz; H-14’); 8.75 (1H; s; C(16)-OH). ^13^C NMR (125.7 MHz; DMSO-*d*_6_) *δ* (ppm): 7.5 (C-18); 21.4 (C-3’); 30.4 (C-19); 31.1 (C-2’); 38.0 (N(1)-CH_3_); 42.4 (C-20); 43.6 (C-6); 50.4 (C-3); 51.1 (C-5); 51.7 (C(16)-COOCH_3_); 52.0 (C-7); 52.6 (br, C-6’, C-7’, C-9’ C-10’); 55.0 (C(11)-OCH_3_); 56.4 (C-4’); 66.1 (C-21); 75.7 (C-17); 78.6 (C-16); 82.8 (C-2); 95.4 (C-12); 104.5 (C-10); 111.2 (C-15’); 115.3 (C-16’); 123.0 (C-9); 124.4 (C-14); 125.3 (C-8); 129.8 (C-15); 144.5 (C-14’); 146.9 (C-12’); 153.4 (C-13); 158.1 (C-11’); 160.4 (C-11); 171.6 (C(16)-COOCH_3_); 172.4 (C-1’). HRMS: M+H=663.33884 (delta = 0.5 ppm; C_36_H_47_O_8_N_4_).

**Figure S81.** The skeleton numbering of compound **26** used for NMR assignment.


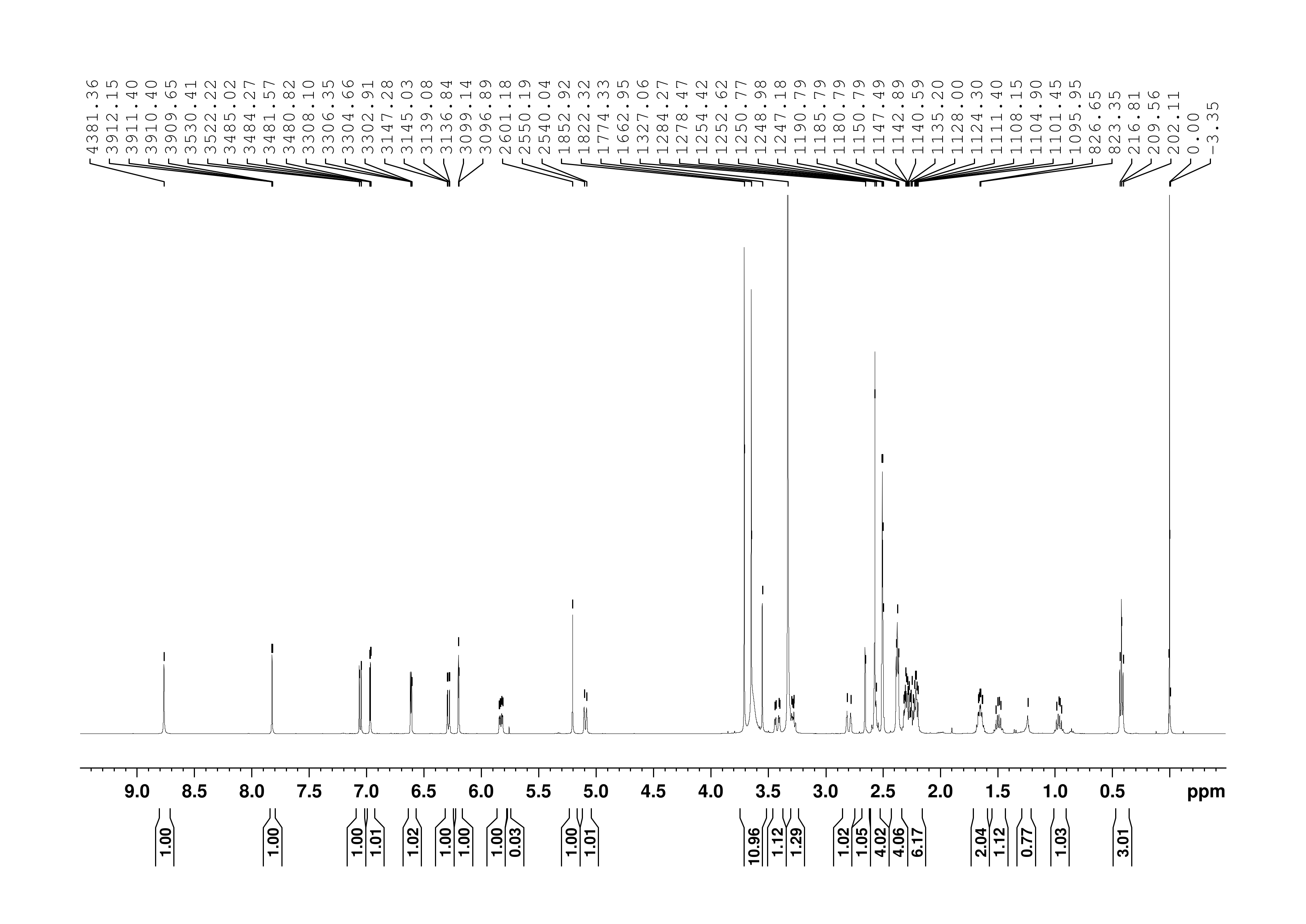


**Figure S82.** ^1^H NMR spectrum of compound **26.**


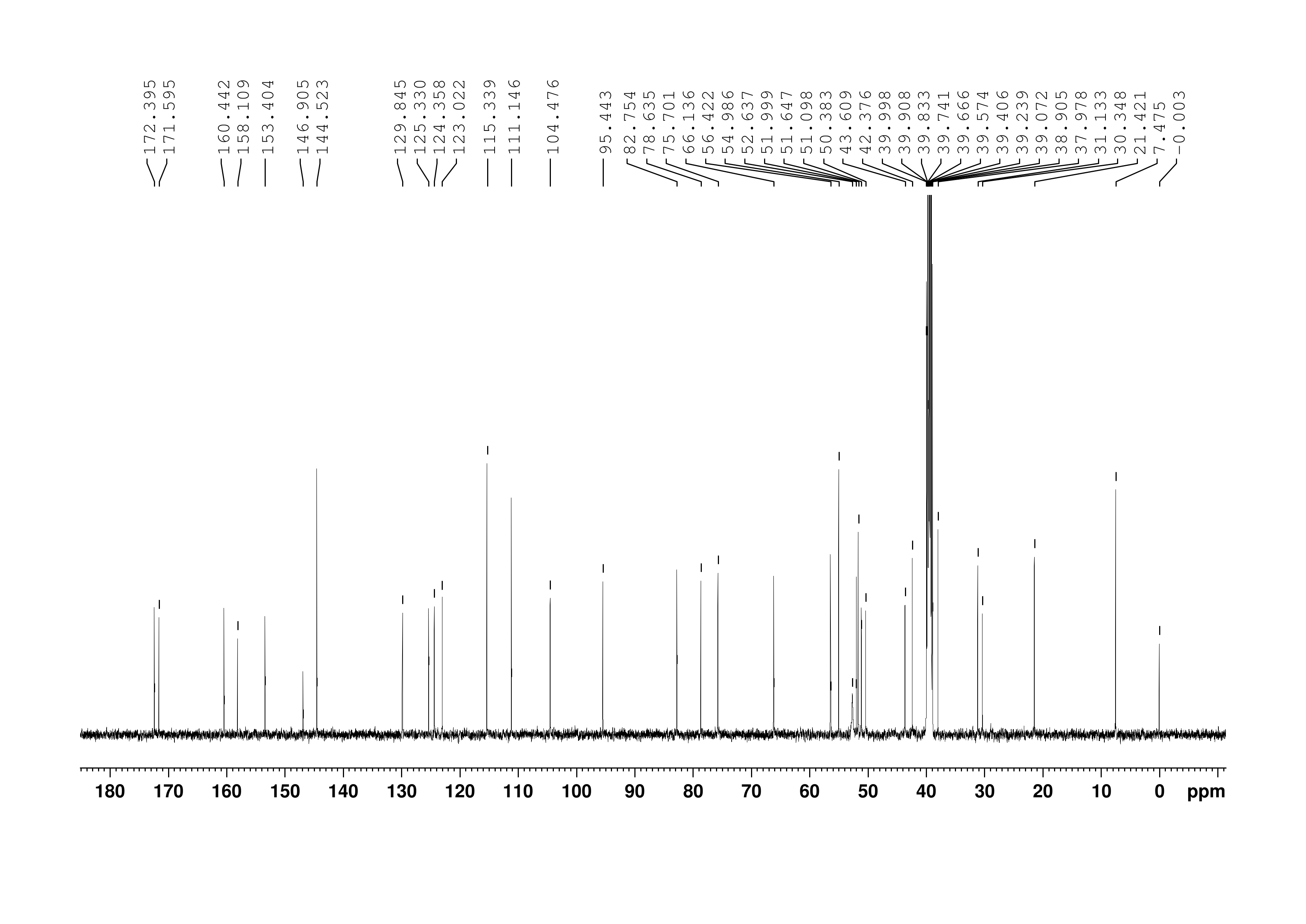


**Figure S83.** ^13^C NMR spectrum of compound **26.**


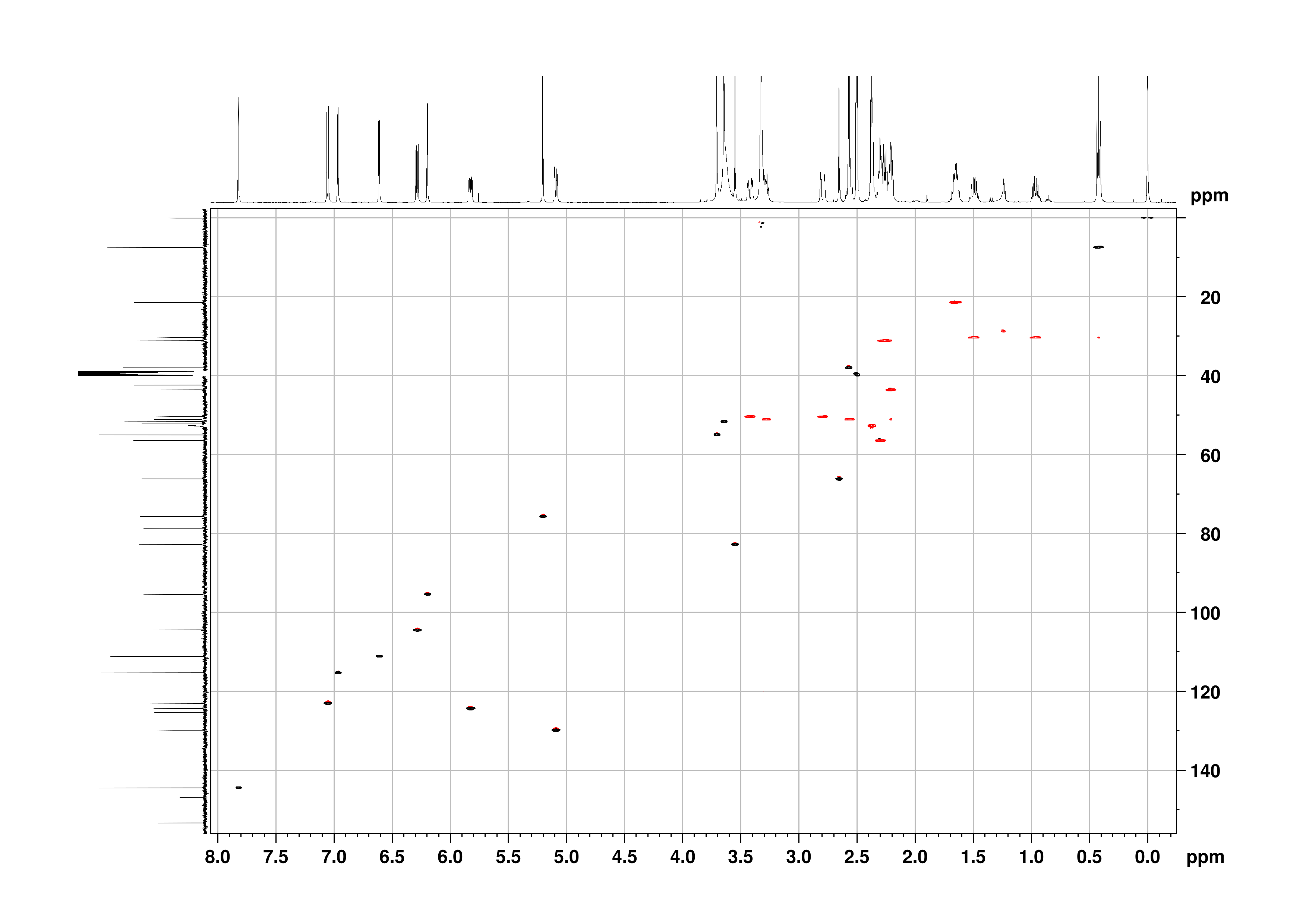


**Figure S84.** HSQC spectrum of compound **26.**


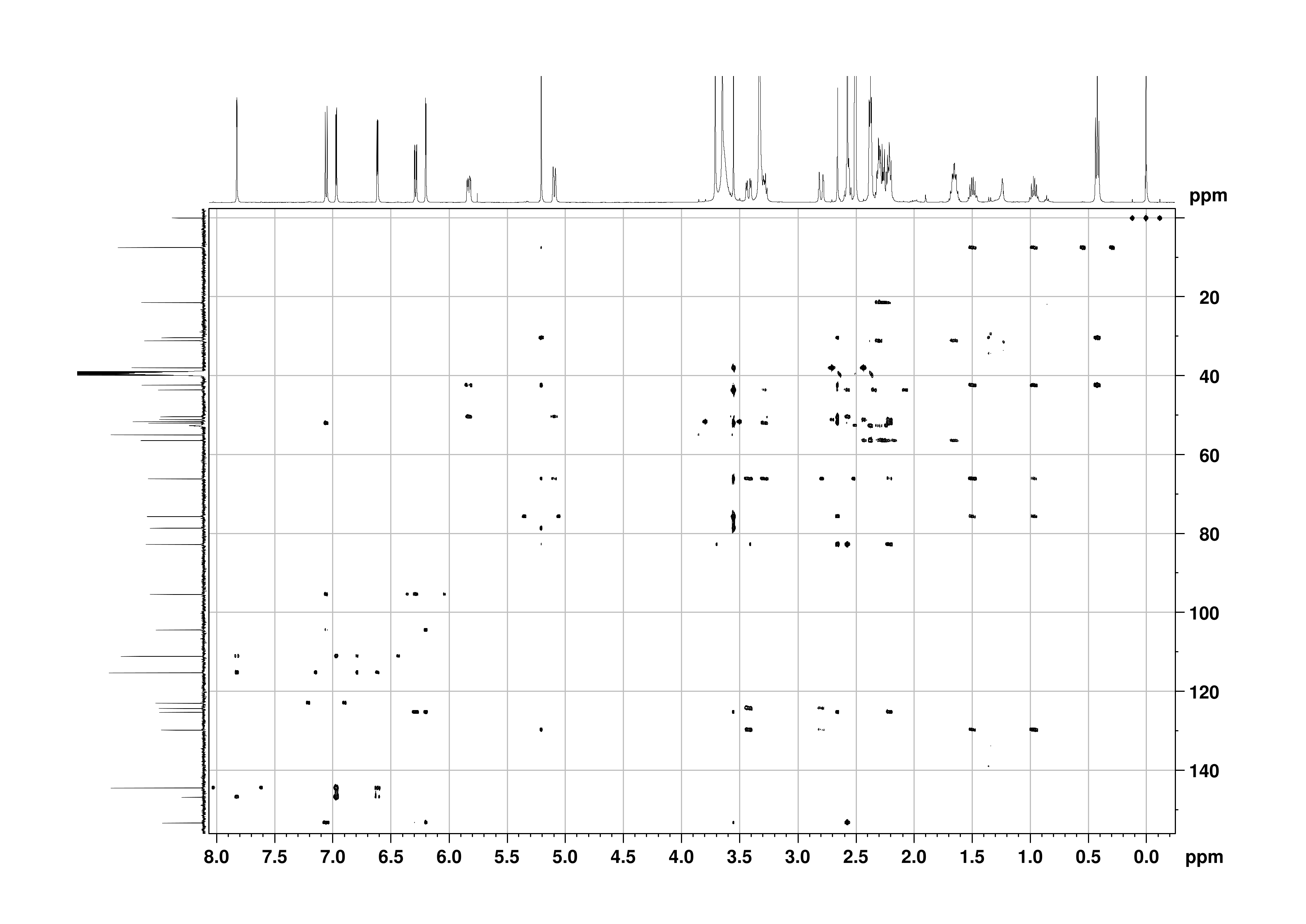


**Figure S85.** ^1^H-^13^C HMBC spectrum of compound **26.**


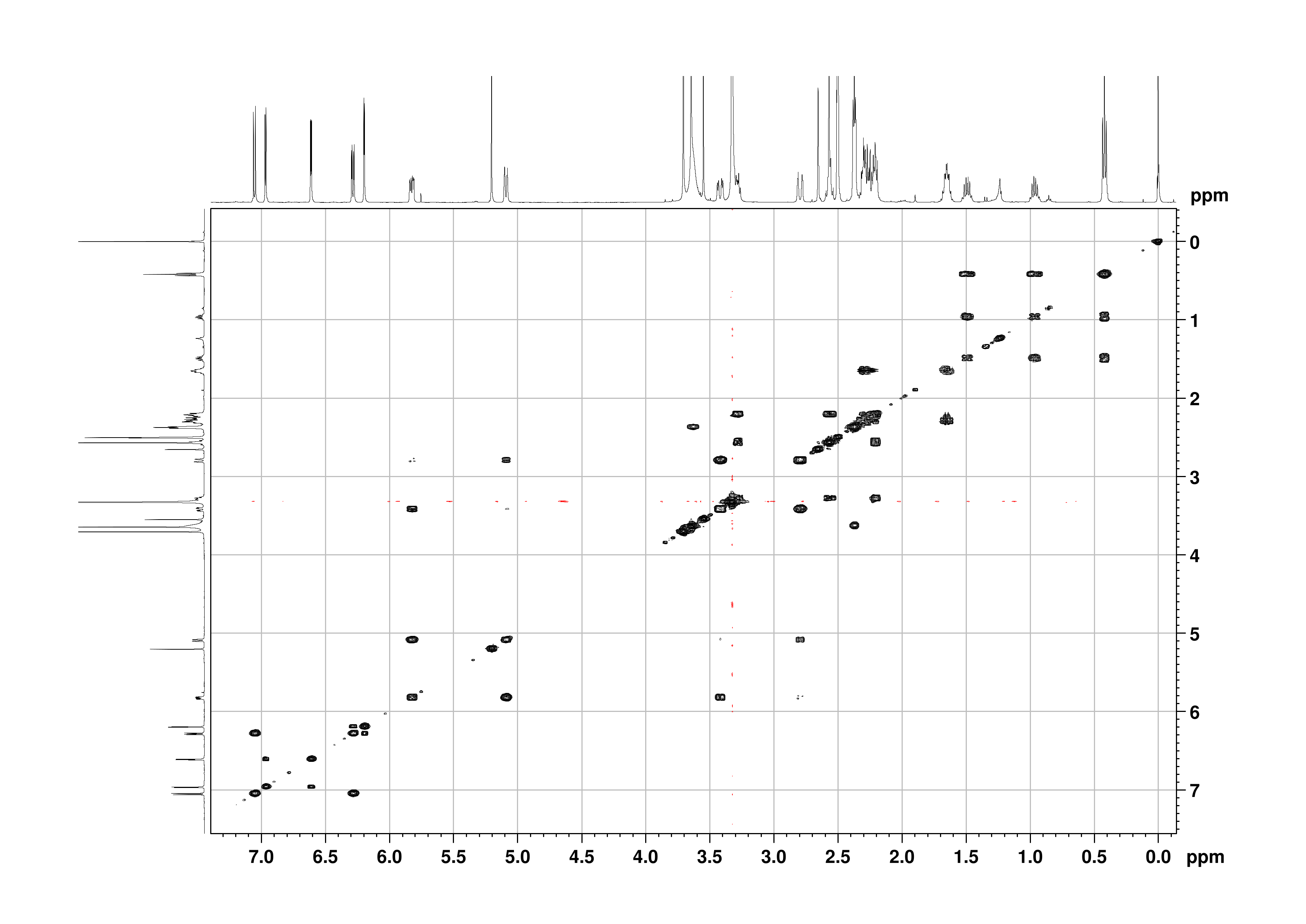


**Figure S86.** COSY spectrum of compound **26.**


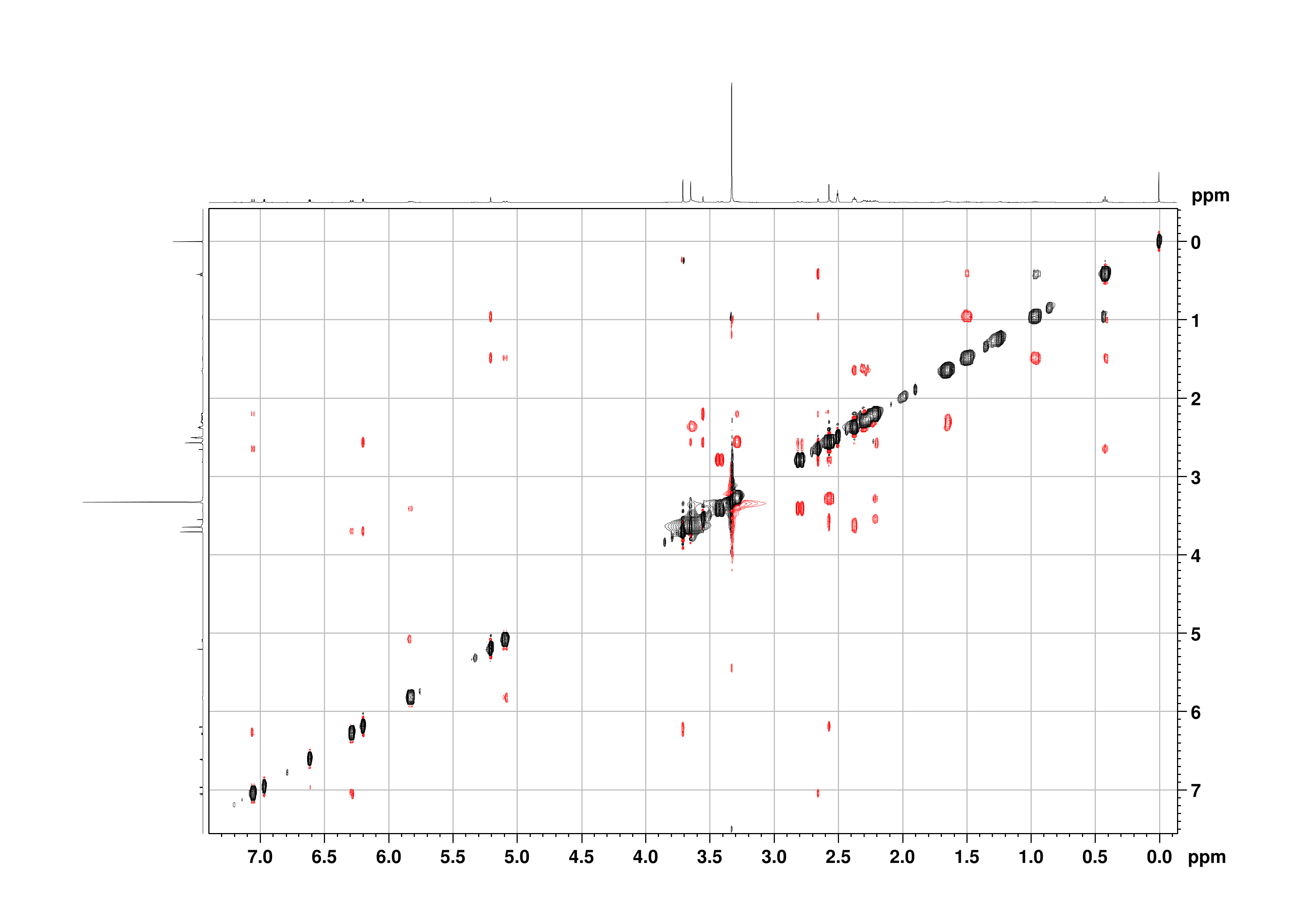


**Figure S87.** ROESY spectrum of compound **26.**

**Figure S88.** HRMS spectrum of compound **26.**

**S.1.2.3. General procedure for the synthesis of products (27-32)**

To a solution of 17-(*O*-3-carboxypropanoyl)vindoline (**15**) (100 mg, 0.19 mmol) in DCM (10mL) *N*,*N*′-dicyclohexylcarbodiimide (47 mg, 0.23 mmol, 1.2 eq.) was added at 0 ^o^C. After stirring the mixture for 5 min the appropriate piperazine (**5-10**) (0.29 mmol, 1.5 eq.) was added and the reaction mixture was refluxed at the time presented in Scheme 3. Then, the mixture was filtered, and the filtrate was evaporated under reduced pressure. The pale yellow crystalline products (**27**-**32**) were isolated after preparative TLC (DCM : MeOH = 10 : 1).

Product **27**

36 mg (31%). M.p.: 66-67 °C. TLC (DCM : MeOH = 10 : 1); *R_f_* = 0.24. IR (KBr) 2922, 1736, 1644, 1501, 1435, 1223, 1166, 1140, 1085, 1026 cm^-1^. ^1^H NMR (599.8 MHz; DMSO-*d*_6_) *δ* (ppm): 0.42 (3H; t; *J* = 7.2 Hz; H_3_-18); 0.91 (1H; dq; *J* = 14.0, 7.2 Hz; H_x_-19); 1.46 (1H; dq; *J* = 14.0, 7.3 Hz; H_y_-19); 2.12-2.39 (10H; m; H_2_-6, H_x_-2’, H_2_-7’, H_2_-9’, H_3_-11’ ); 2.41-2.61 (7H; m; N(1)-CH_3_, H_x_-5, H_y_-2’, H_2_-3’); 2.67 (1H; s; H-21); 2.79 (1H; br d; *J* = 16.4 Hz; H_x_-3); 3.23-3.49 (6H; m; H_y_-3, H_y_-5, H_2_-6’, H_2_-10’); 3.54 (1H; s; H-2); 3.64 (3H; s; C(16)-COOCH_3_); 3.70 (3H; s; C(11)-OCH_3_); 5.16-23 (2H; m; H-15, H-17); 5.80 (1H; br dd; *J* = 10.0, 4.3 Hz; H-14); 6.18 (1H; ~s; H-12); 6.27 (1H; br d; *J* = 8.2 Hz; H-10); 7.04 (1H; d; *J* = 8.1 Hz; H-9); 8.78 (1H; s; C(16)-OH). ^13^C NMR (150.8 MHz; DMSO-*d*_6_) *δ* (ppm): 7.6 (C-18); 27.2 (C-3’); 28.9 (C-2’); 30.2 (C-19); 38.0 (N(1)-CH_3_); 40.9 (C-6’ v, C-10’); 42.4 (C-20); 43.7 (C-6); 44.3 (C-6’ v, C-10’); 45.5 (C-11’); 50.3 (C-3); 51.0 (C-5); 51.6 (C(16)-COOCH_3_); 52.1 (C-7); 54.1 (C-7’ v, C-9’); 54.5 (C-7’ v, C-9’); 55.0 (C(11)-OCH_3_); 66.0 (C-21); 75.7 (C-17); 78.7 (C-16); 82.8 (C-2); 95.4 (C-12); 104.4 (C-10); 123.0 (C-9); 123.9 (C-14); 125.3 (C-8); 130.1 (C-15); 153.3 (C-13); 160.4 (C-11); 169.1 (C-4’); 171.5 (C(16)-COOCH_3_); 171.9 (C-1’). HRMS: M+H=597.32870 (delta = -0.2 ppm; C_32_H_45_O_7_N_4_).

**Figure S89.** The skeleton numbering of compound **27** used for NMR assignment.


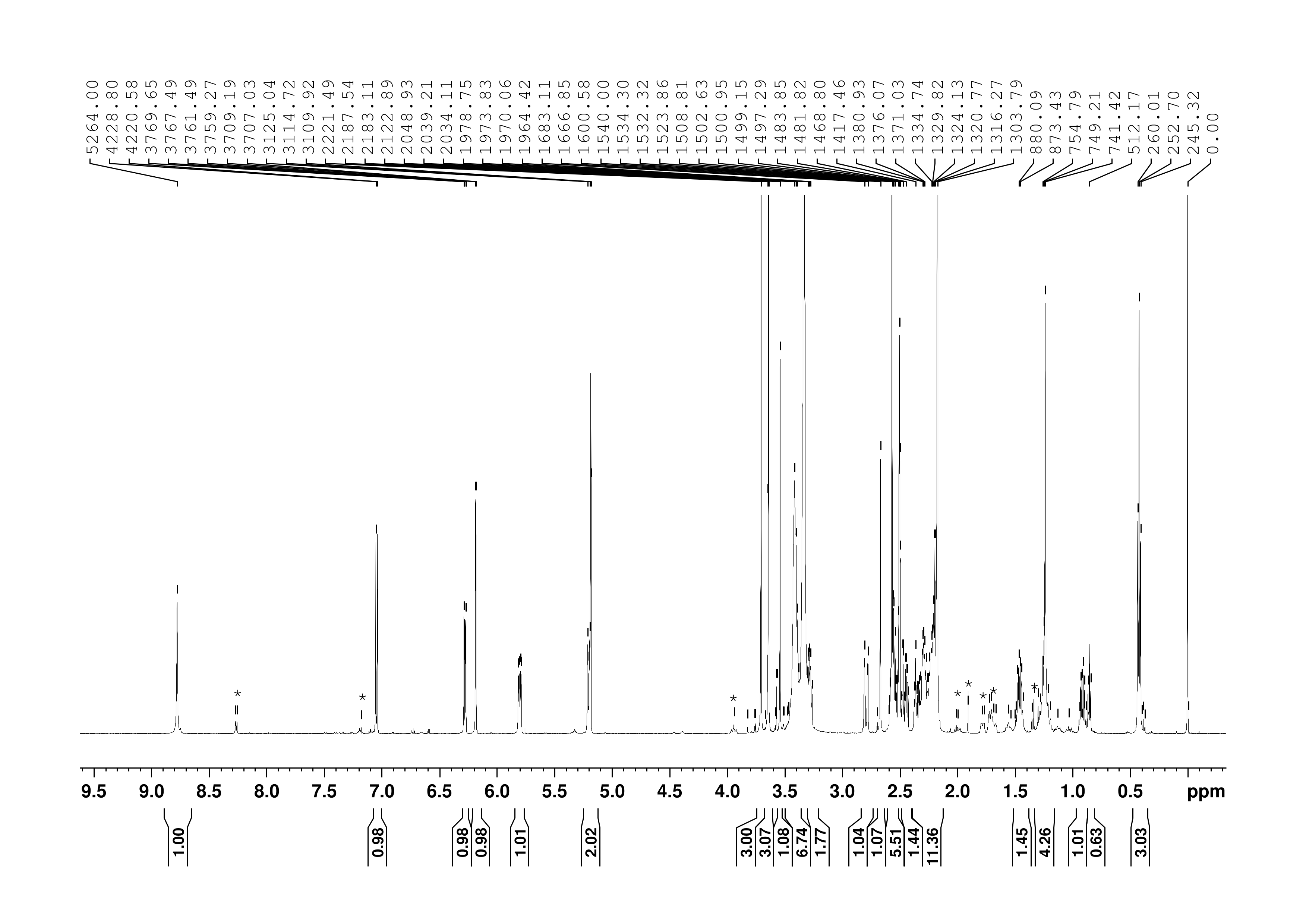


**Figure S90.** ^1^H NMR spectrum of compound **27.**


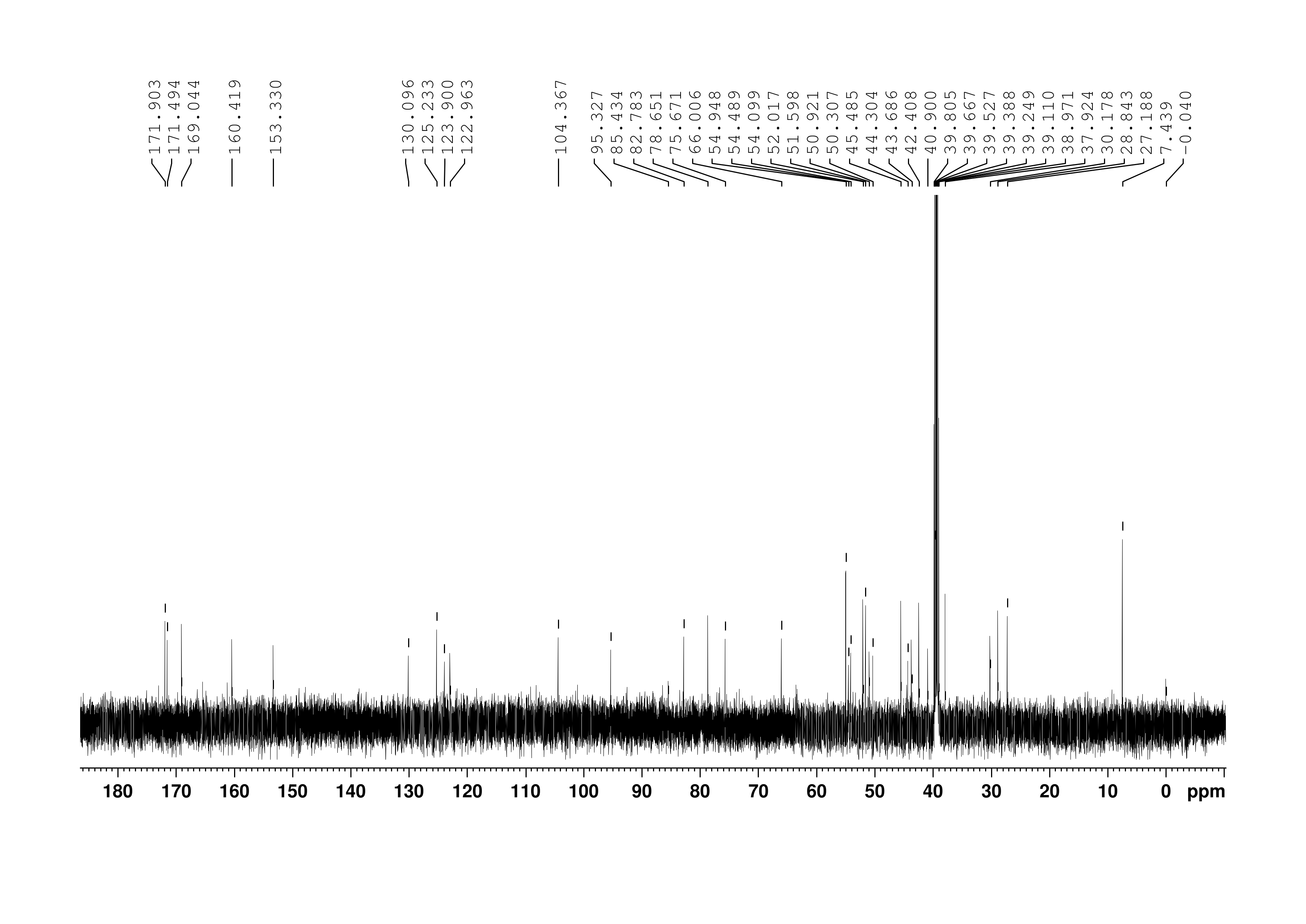


**Figure S91.** ^13^C NMR spectrum of compound **27.**


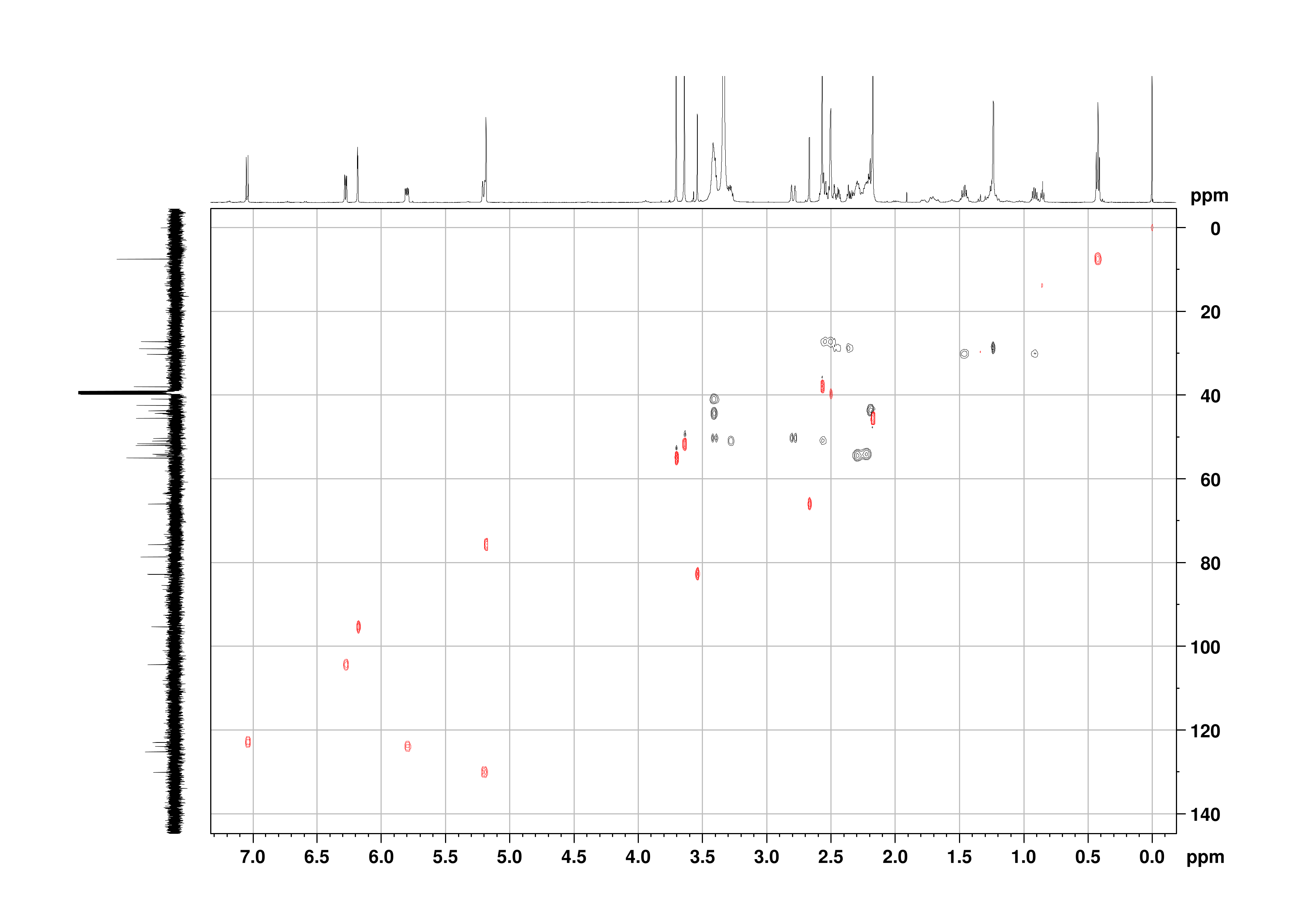


**Figure S92.** HSQC spectrum of compound **27.**


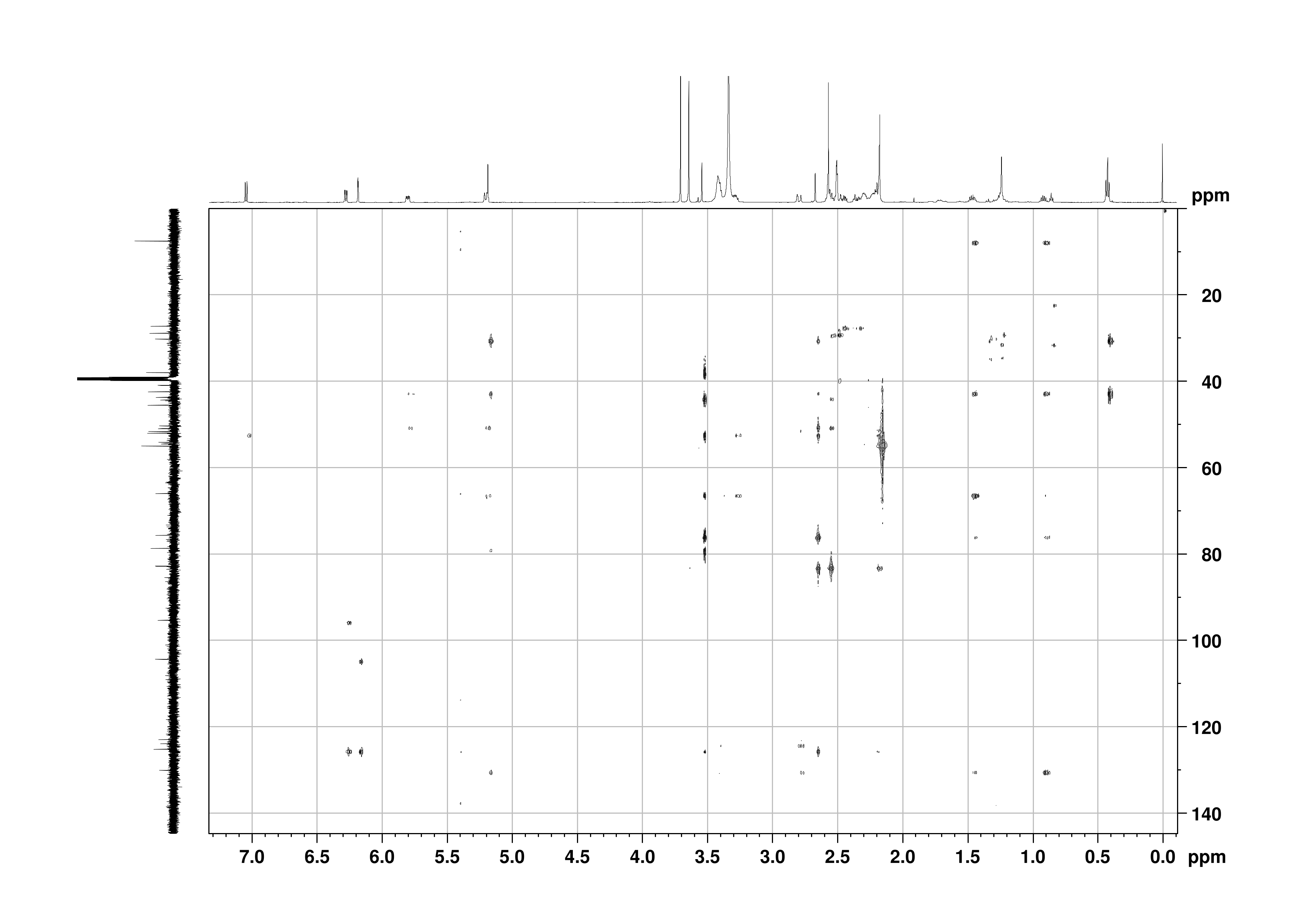


**Figure S93.** ^1^H-^13^C HMBC spectrum of compound **27.**


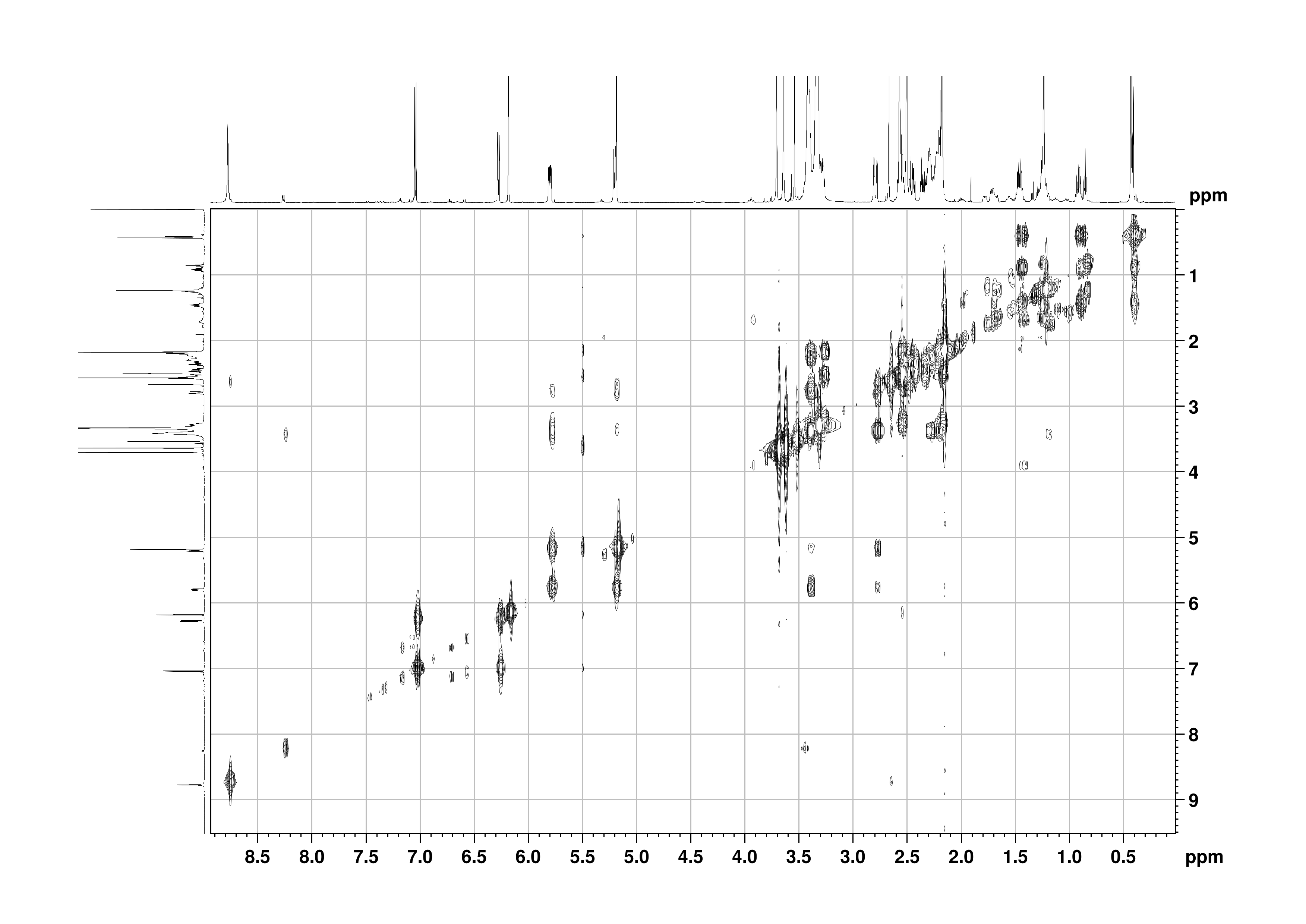


**Figure S94.** COSY spectrum of compound **27.**

**Figure S95.** HRMS spectrum of compound **27.**

Product **28**

69 mg (49%). M.p.: 98-99 °C. TLC (DCM : MeOH = 15 : 1); *R_f_* = 0.54. IR (KBr) 2922, 1736, 1648, 1614, 1500, 1436, 1328, 1224, 1158, 1105, 1069, 823 cm^-1^. ^1^H NMR (599.8 MHz; DMSO-*d*_6_) *δ* (ppm): 0.42 (3H; t; *J* = 7.4 Hz; H_3_-18); 0.92 (1H; dq; *J* = 14.2, 7.3 Hz; H_x_-19); 1.47 (1H; dq; *J* = 14.2, 7.5 Hz; H_y_-19); 2.14-2.23 (2H; m; H_2_-6); 2.40 (1H; ~dt; *J* = 16.6, 6.1 Hz; H_x_-2’); 2.46-2.70 (8H; m; N(1)-CH_3_, H_x_-5, H-21, H_y_-2’, H_2_-3’); 2.78 (1H; br d; *J* = 16.4 Hz; H_x_-3); 3.20-3.45 (6H; m; H_y_-3, H_y_-5, H_2_-9’, H_2_-7’); 3.54 (1H; s; H-2); 3.55-3.63 (4H; m; H_2_-6’, H_2_-10’); 3.65 (3H; s; C(16)-COOCH_3_); 3.70 (3H; s; C(11)-OCH_3_); 5.18-23 (2H; m; H-15, H-17); 5.80 (1H; ddd; *J* = 10.2, 4.9, 1.3 Hz; H-14); 6.18 (1H; d; *J* = 2.2 Hz; H-12); 6.27 (1H; dd; *J* = 8.2, 2.2 Hz; H-10); 7.04 (1H; d; *J* = 8.2 Hz; H-9); 7.06 (2H; ~d; *J* = 8.8 Hz; H-12’, H-16’); 7.52 (2H; ~d; *J* = 8.8 Hz; H-13’, H-15’); 8.78 (1H; s; C(16)-OH). ^13^C NMR (150.8 MHz; DMSO-*d*_6_) *δ* (ppm): 7.6 (C-18); 27.3 (C-3’); 29.0 (C-2’); 30.3 (C-19); 38.1 (N(1)-CH_3_); 40.7 (C-6’ v, C-10’); 42.5 (C-20); 43.8 (C-6); 44.1 (C-6’ v, C-10’); 46.6 (C-7’ v, C-9’); 46.9 (C-7’ v, C-9’); 50.4 (C-3); 51.1 (C-5); 51.7 (C(16)-COOCH_3_); 52.1 (C-7); 55.1 (C(11)-OCH_3_); 66.1 (C-21); 75.9 (C-17); 78.7 (C-16); 82.9 (C-2); 95.5 (C-12); 104.5 (C-10); 114.3 (C-12’, C-16’); 123.1 (C-9); 124.0 (C-14); 125.3 (C-8); 126.2 (C-13’, C-15’); 130.2 (C-15); 152.9 (C-11’); 153.4 (C-13); 160.5 (C-11); 169.4 (C-4’); 171.6 (C(16)-COOCH_3_); 172.0 (C-1’). HRMS: M+H=727.32923 (delta = -2.9 ppm; C_38_H_46_O_7_N_4_F_3_).

**Figure S96.** The skeleton numbering of compound **28** used for NMR assignment.


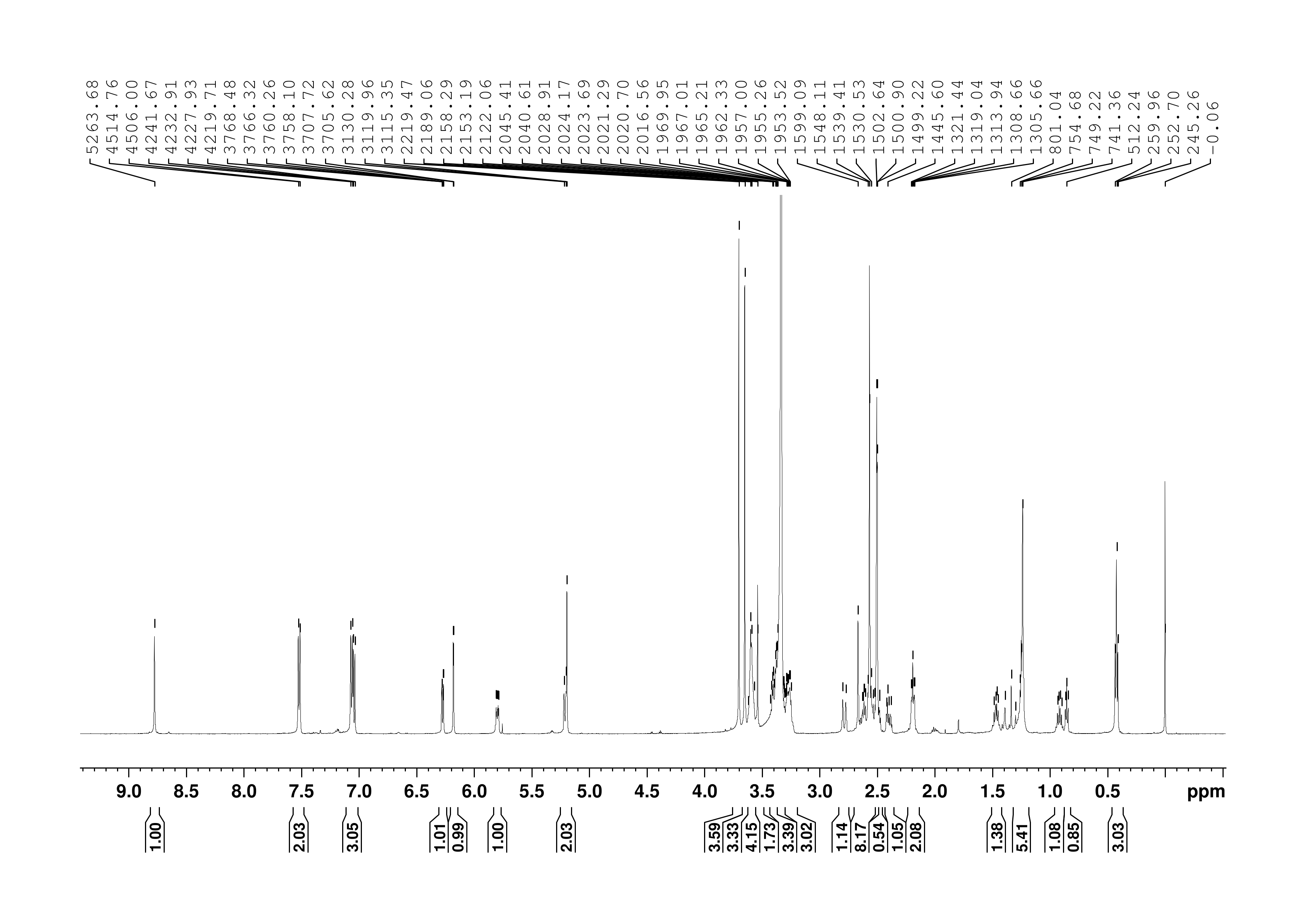


**Figure S97.** ^1^H NMR spectrum of compound **28.**


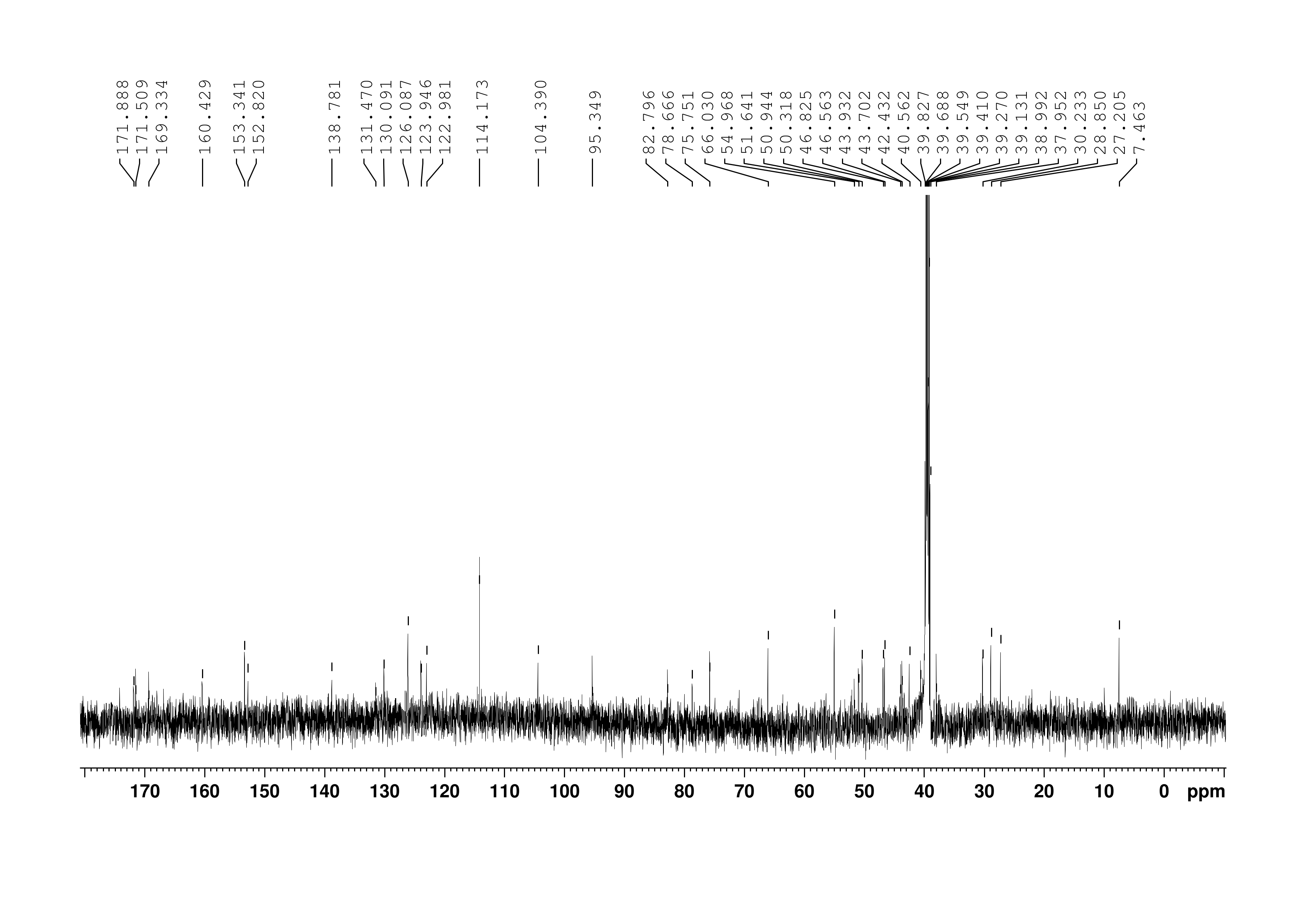


**Figure S98.** ^13^C NMR spectrum of compound **28.**


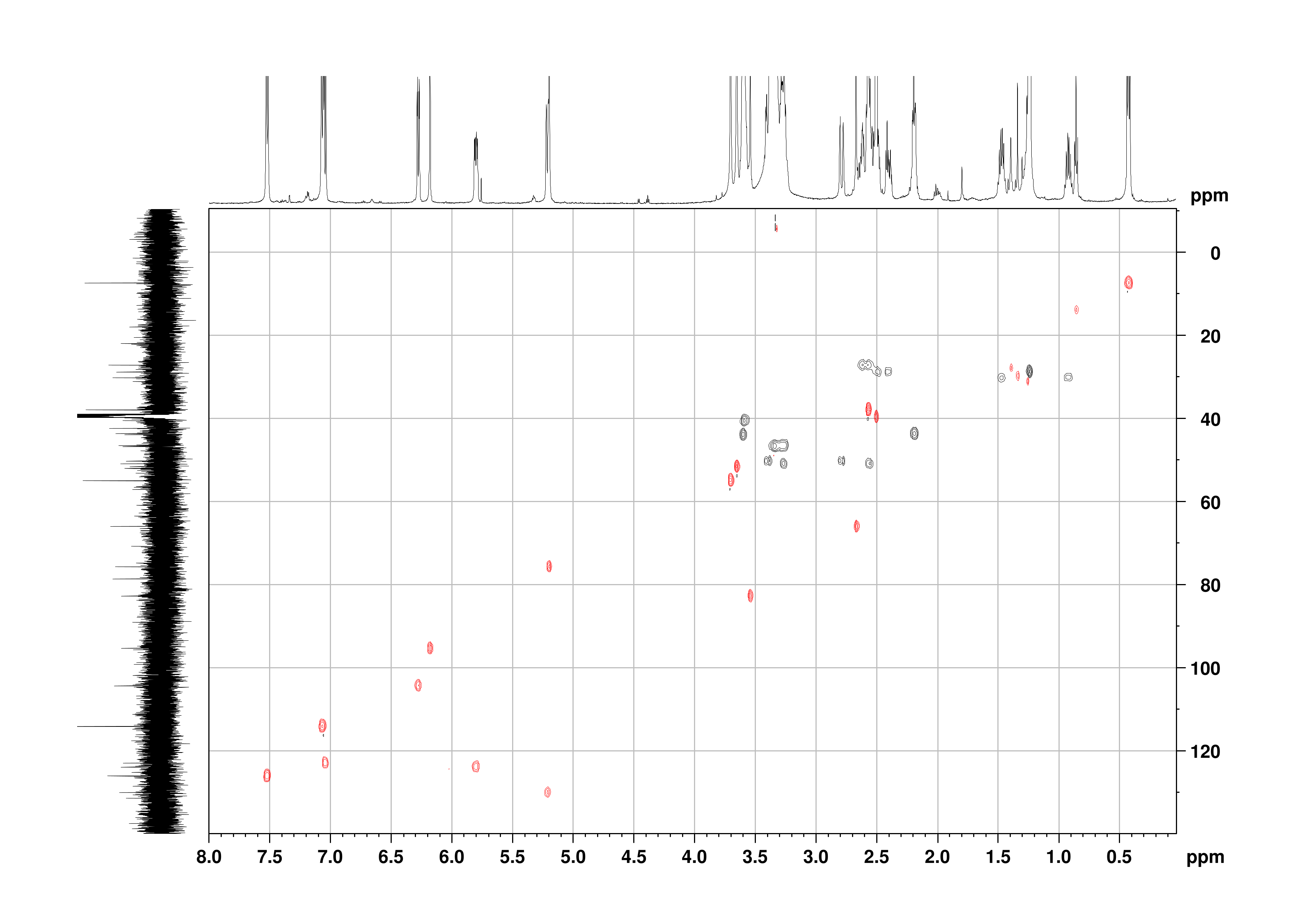


**Figure S99.** HSQC spectrum of compound **28.**


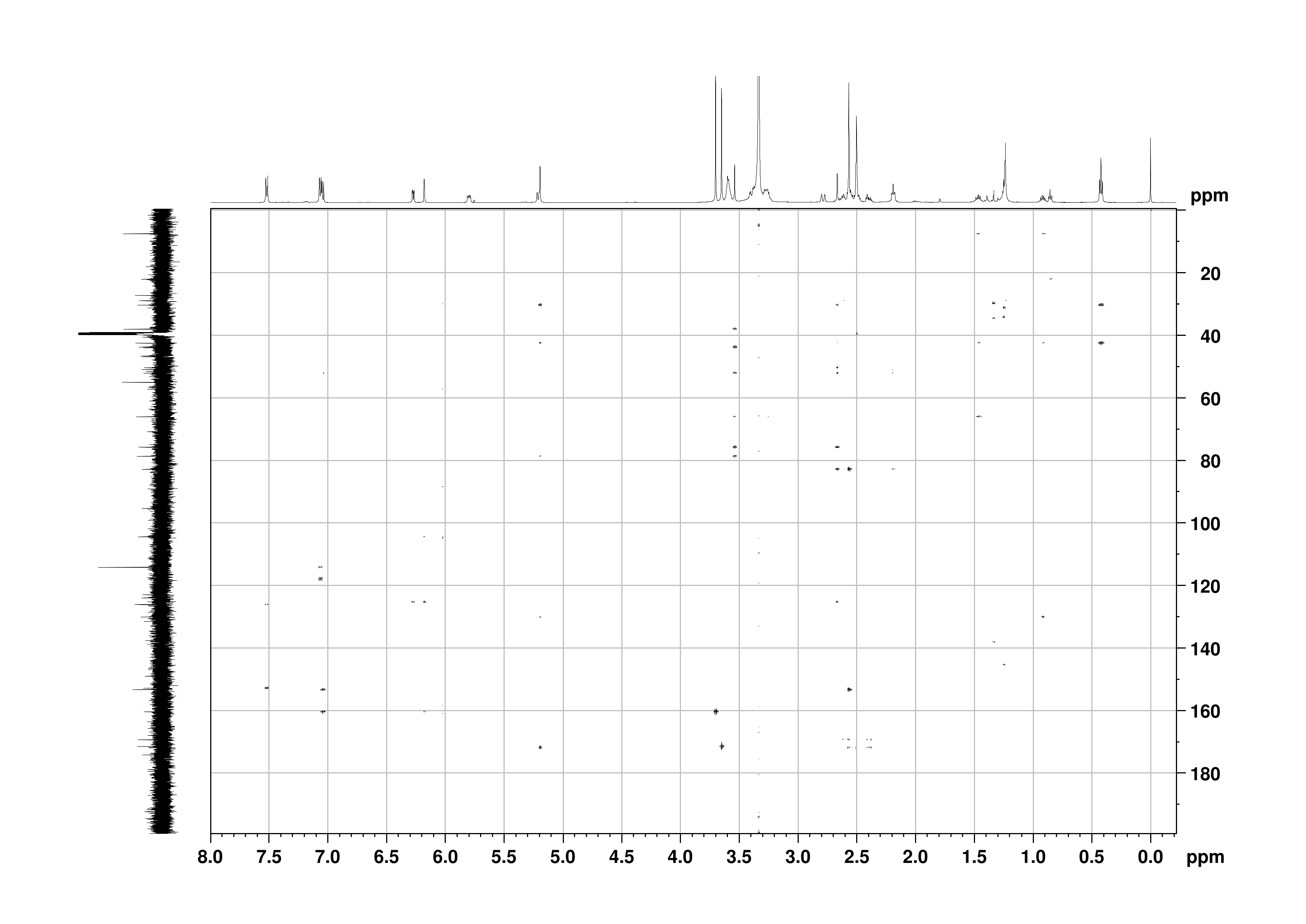


**Figure S100.** ^1^H-^13^C HMBC spectrum of compound **28.**


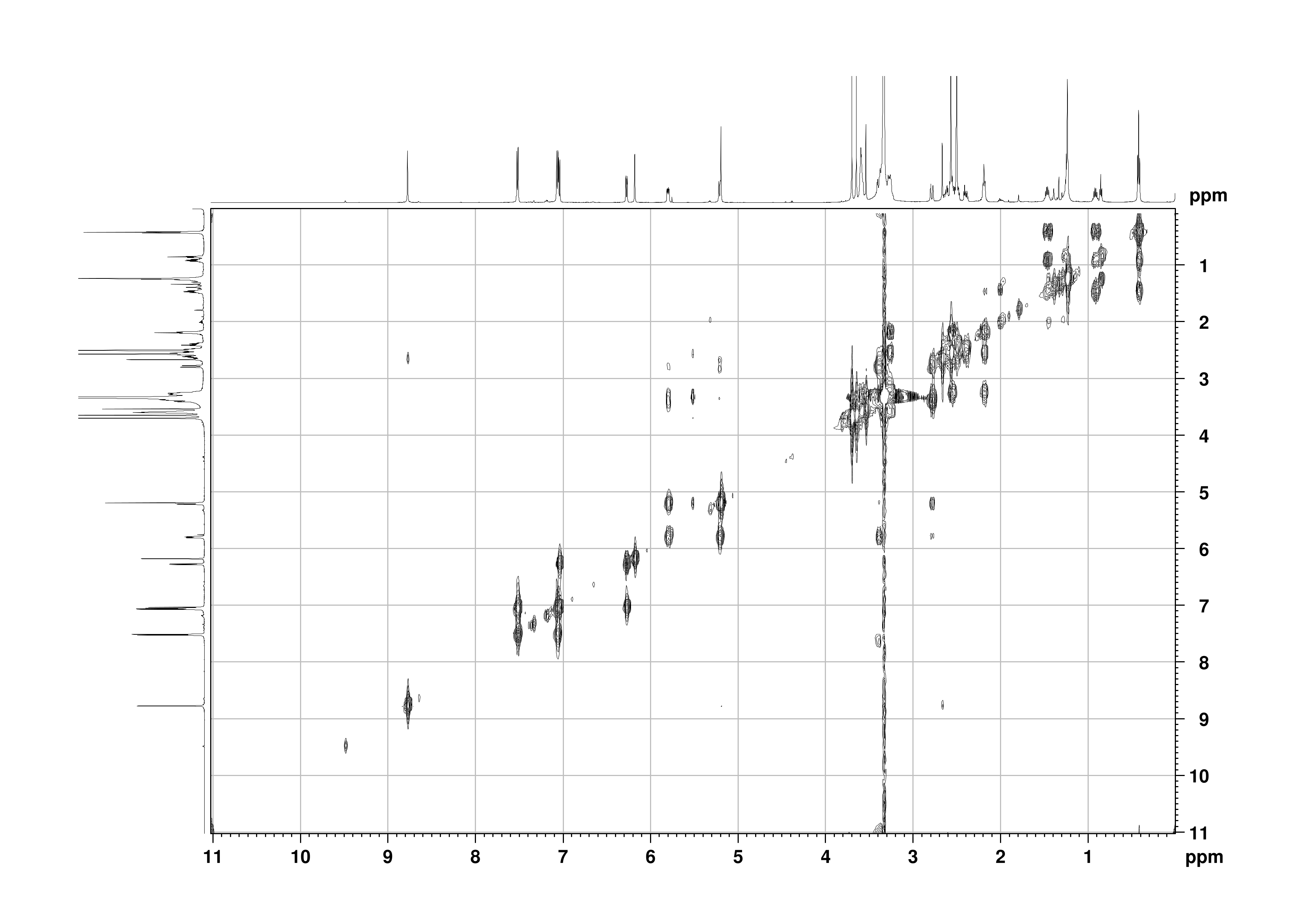


**Figure S101.** COSY spectrum of compound **28.**

**Figure S102.** HRMS spectrum of compound **28.**

Product **29**

43 mg (30%). M.p.: 88-90 °C. TLC (DCM : MeOH = 15 : 1); *R_f_* = 0.56. IR (KBr) 2944, 2808, 1741, 1650, 1326, 1246, 1164, 1121, 1046 cm^-1^. ^1^H NMR (499.9 MHz; DMSO-*d*_6_) *δ* (ppm): 0.42 (3H; t; *J* = 7.4 Hz; H_3_-18); 0.91 (1H; dq; *J* = 14.2, 7.3 Hz; H_x_-19); 1.46 (1H; dq; *J* = 14.2, 7.4 Hz; H_y_-19); 2.14-2.24 (2H; m; H_2_-6); 2.24-2.62 (12H; m; N(1)-CH_3_, H_x_-5, H_2_-2’, H_2_-3’, H_2_-7’, H_2_-9’); 2.67 (1H; s; H-21); 2.79 (1H; br d; *J* = 16.3 Hz; H_x_-3); 3.23-3.50 (6H; m; H_y_-3, H_y_-5.H_2_-6’, H_2_-10’); 3.54 (1H; s; H-2); 3.58 (2H; s; H_2_-11’); 3.64 (3H; s; C(16)-COOCH_3_); 3.71 (3H; s; C(11)-OCH_3_); 5.15-5.22 (2H;m; H-15, H-17); 5.79 (1H; ddd; *J* = 10.1, 4.8, 1.2 Hz; H-14); 6.18 (1H; d; *J* = 2.2 Hz; H-12); 6.28 (1H; dd; *J* = 8.2, 2.2 Hz; H-10); 7.04 (1H; d; *J* = 8.2 Hz; H-9); 7.55 (2H; d; *J* = 8.0 Hz; H-13’, H-17’); 7.69 (2H; d; *J* = 8.1 Hz; H-14’, H-16’); 8.77 (1H; s; C(16)-OH). ^13^C NMR (125.7 MHz; DMSO-*d*_6_) *δ* (ppm): 7.5 (C-18); 27.2 (C-2’); 28.9 (C-3’); 30.2 (C-19); 38.0 (N(1)-CH_3_); 41.1 (C-6’ v, C-10’) 42.4 (C-20); 43.7 (C-6); 44.5 (C-6’ v, C-10’); 50.3 (C-3); 51.0 (C-5); 51.6 (C(16)-COOCH_3_); 52.0 (C-7); 52.1; (C-7’ v, C-9’); 52.5 (C-7’ v, C-9’); 55.0 (C(11)-OCH_3_); 61.0 (C-11’); 66.0 (C-21); 75.7 (C-17); 78.7 (C-16); 82.8 (C-2); 95.4 (C-12); 104.4 (C-10); 123.0 (C-9); 123.9 (C-14); 125.0 (q; *J* = 3.6 Hz; C-14’, C-16’); 125.3 (C-8); 127.6 (q; *J* = 31.5 Hz; C-15’); 129.3 (C-13’, C-17’); 130.1 (C-15); 142.9 (C-12’); 153.4 (C-13); 160.4 (C-11); 169.0 (C-4’); 171.5 (C(16)-COOCH_3_) 171.9 (C-1’). HRMS: M+H=741.34573 (delta = -1.7 ppm; C_39_H_48_O_7_N_4_F_3_).

**Figure S103.** The skeleton numbering of compound **29** used for NMR assignment.


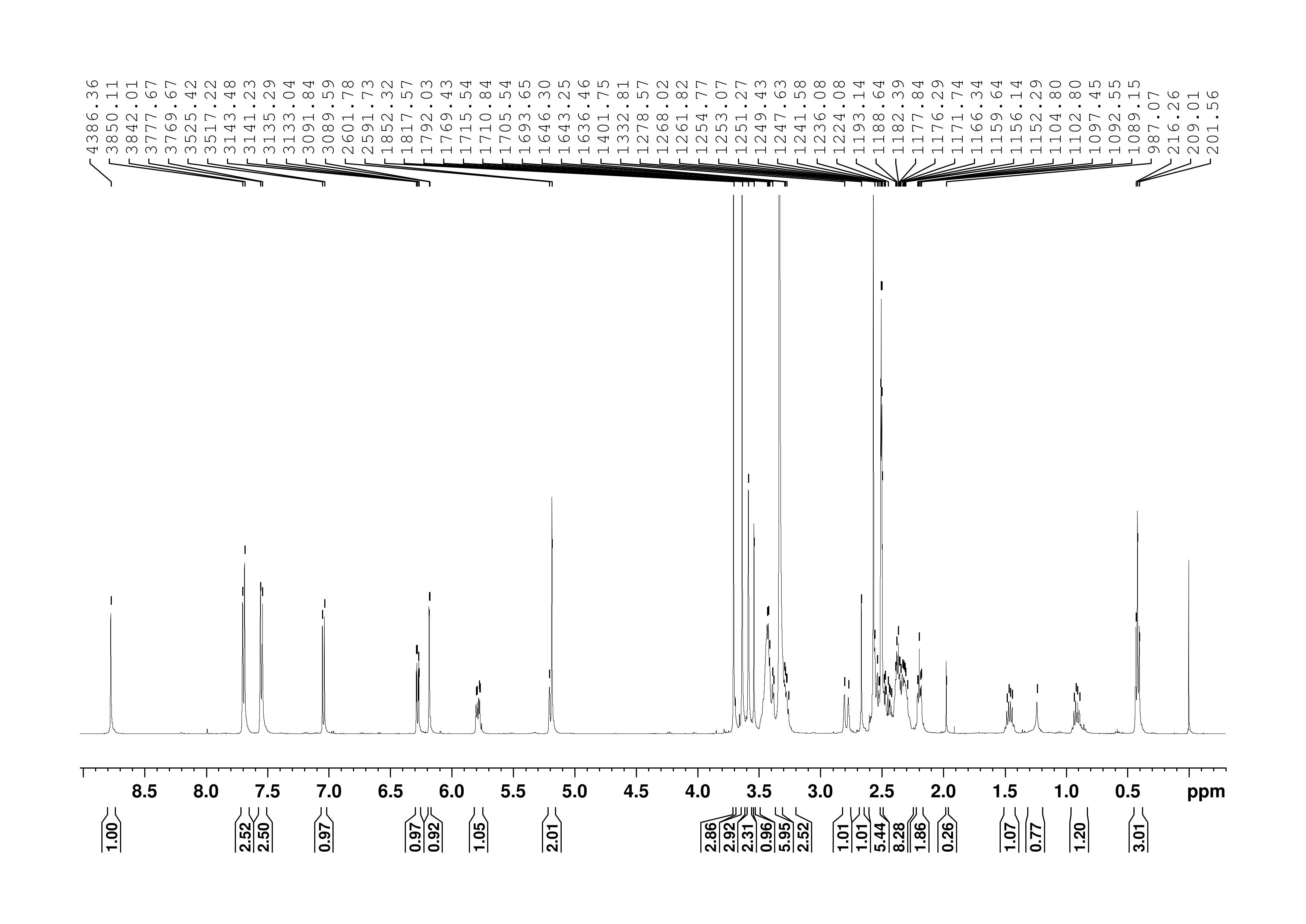


**Figure S104.** ^1^H NMR spectrum of compound **29.**


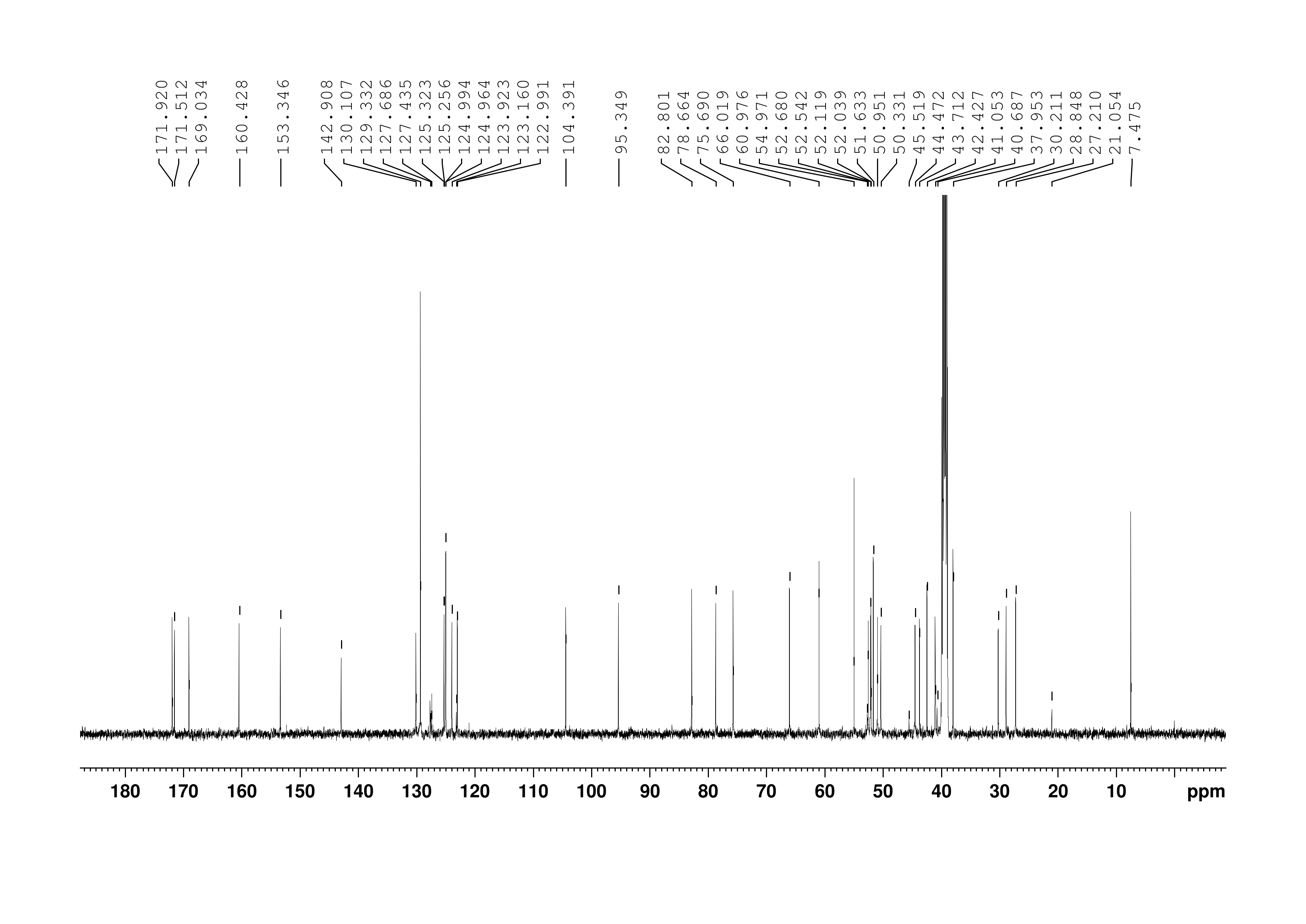


**Figure S105.** ^13^C NMR spectrum of compound **29.**


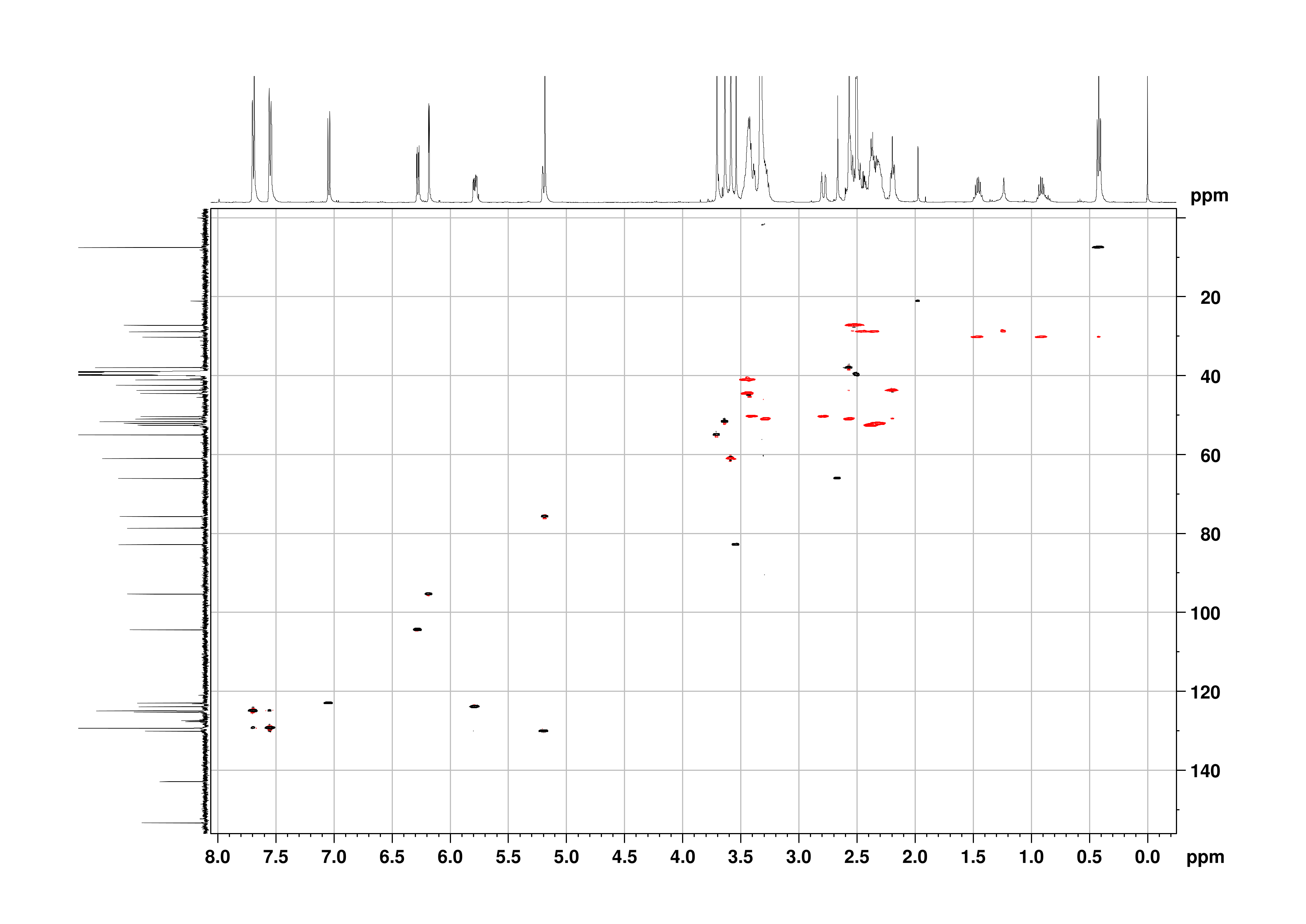


**Figure S106.** HSQC spectrum of compound **29.**

**
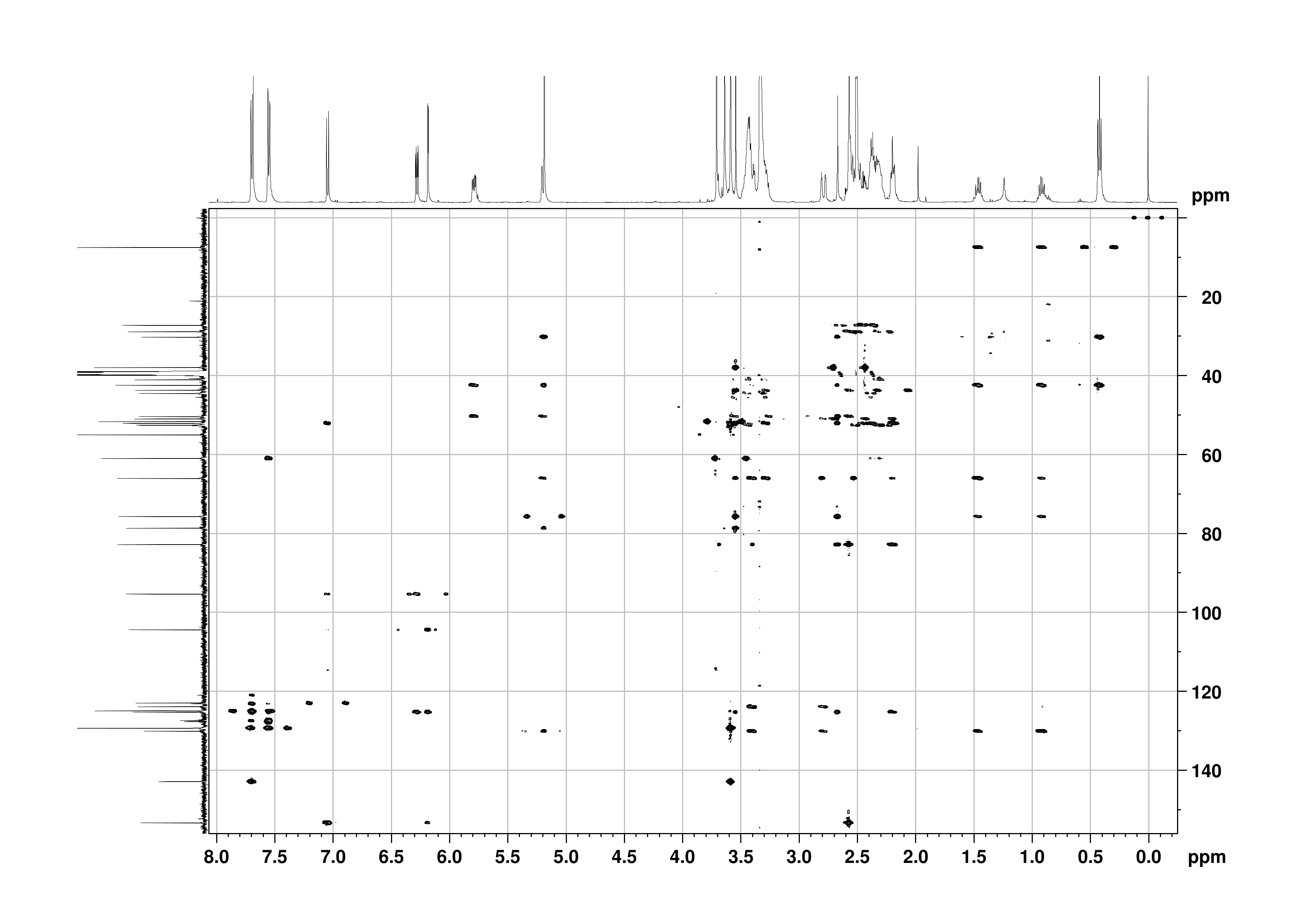
**

**Figure S107.** ^1^H-^13^C HMBC spectrum of compound **29.**


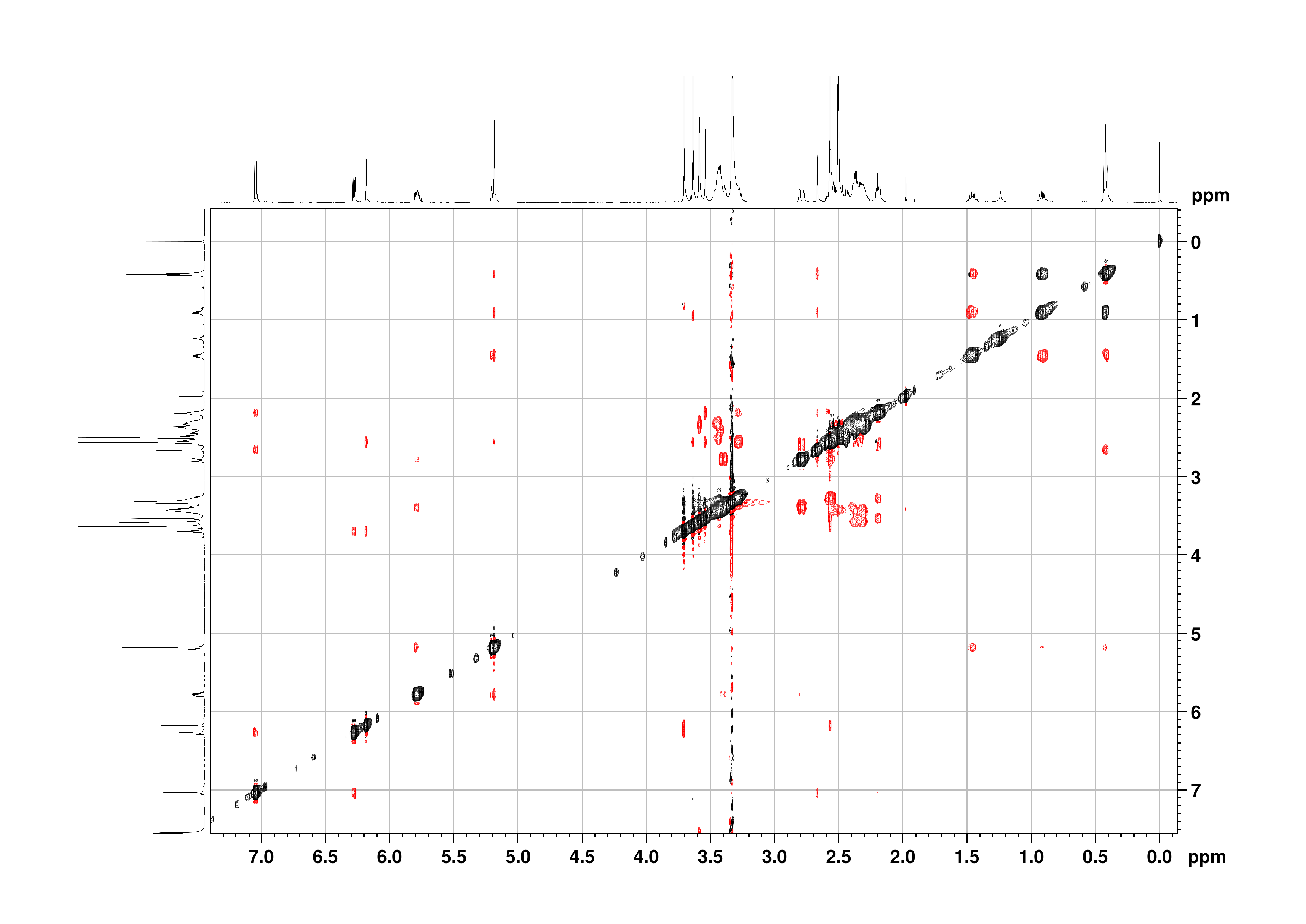


**Figure S108.** ROESY spectrum of compound **29.**

**Figure S109.** HRMS spectrum of compound **29**.

Product **30**

58 mg (43%). M.p.: 83-84 °C. TLC (DCM : MeOH = 10 : 1); *R_f_* = 0.57. IR (KBr) 1736, 1644, 1501, 1435, 1219, 1163, 1000, 827 cm^-1^. ^1^H NMR (499.9 MHz; DMSO-*d*_6_) *δ* (ppm): 0.42 (3H; t; *J* = 7.4 Hz; H_3_-18); 0.91 (1H; dq; *J* = 14.2, 7.3 Hz; H_x_-19); 1.46 (1H; dq; *J* = 14.1, 7.4 Hz; H_y_-19); 2.15-2.23 (2H; m; H_2_-6); 2.23-2.40 (5H; m; H_x_-3’, H_2_-7’, H_2_-9’); 2.41-2.61 (7H; m; N(1)-CH_3_, H_x_-5, H_2_-2’, H_y_-3’); 2.66 (1H; s; H-21); 2.79 (1H; br d; *J* = 16.4 Hz; H_x_-3); 3.21-3.51 (8H; m; H_y_-3, H_y_-5, H_2_-6’, H_2_-10’, H_2_-11’); 3.54 (1H; s; H-2); 3.63 (3H; s; C(16)-COOCH_3_); 3.70 (3H; s; C(11)-OCH_3_); 5.16-5.21 (1H; m; H-15, H-17); 5.78 (1H; ddd; *J* = 10.2, 4.8, 1.3 Hz; H-14); 6.18 (1H; d; *J* = 2.2 Hz; H-12); 6.28 (1H; dd; *J* = 8.2, 2.2 Hz; H-10); 7.04 (1H; d; *J* = 8.2 Hz; H-9); 7.11-7.18 (2H; m; H-14’, H-16’); 7.31-7.36 (2H; m; H-13’, H-17’); 8.77 (1H; s; C(16)-OH). ^13^C NMR (125.7 MHz; DMSO-*d*_6_) *δ* (ppm): 7.5 (C-18); 27.2 (C-3’); 28.9 (C-2’); 30.2 (C-19); 38.0 (N(1)-CH_3_); 41.1 (C-6’ v, C-10’); 42.4 (C-20); 43.7 (C-6); 44.5 (C-6’ v, C-10’); 50.3 (C-3); 51.0 (C-5); 51.6 (C(16)-COOCH_3_); 52.0 (C-7, C-7’ v, C-9’); 52.4 (C-7’ v, C-9’); 55.0 (C(11)-OCH_3_); 60.8 (C-11’); 66.0 (C-21); 75.7 (C-17); 78.7 (C-16); 82.8 (C-2); 95.4 (C-12); 104.4 (C-10); 114.8 (d; *J* = 21.1 Hz; C-14’, C-16’); 123.0 (C-9); 123.9 (C-14); 125.3 (C-8); 130.1 (C-15); 130.6 (d; *J* = 8.0 Hz; C-13’, C-17’); 133.9 (d; *J* = 3.0 Hz; C-12’); 153.4 (C-13); 160.4 (C-11); 161.2 (d; *J* = 242.4 Hz; C-15’); 169.0 (C-4’); 171.5 (C(16)-COOCH_3_); 171.9 (C-1’). HRMS: M+H=691.34962 (delta = -0.8 ppm; C_38_H_48_O_7_N_4_F_3_).

**Figure S110.** The skeleton numbering of compound **30** used for NMR assignment.


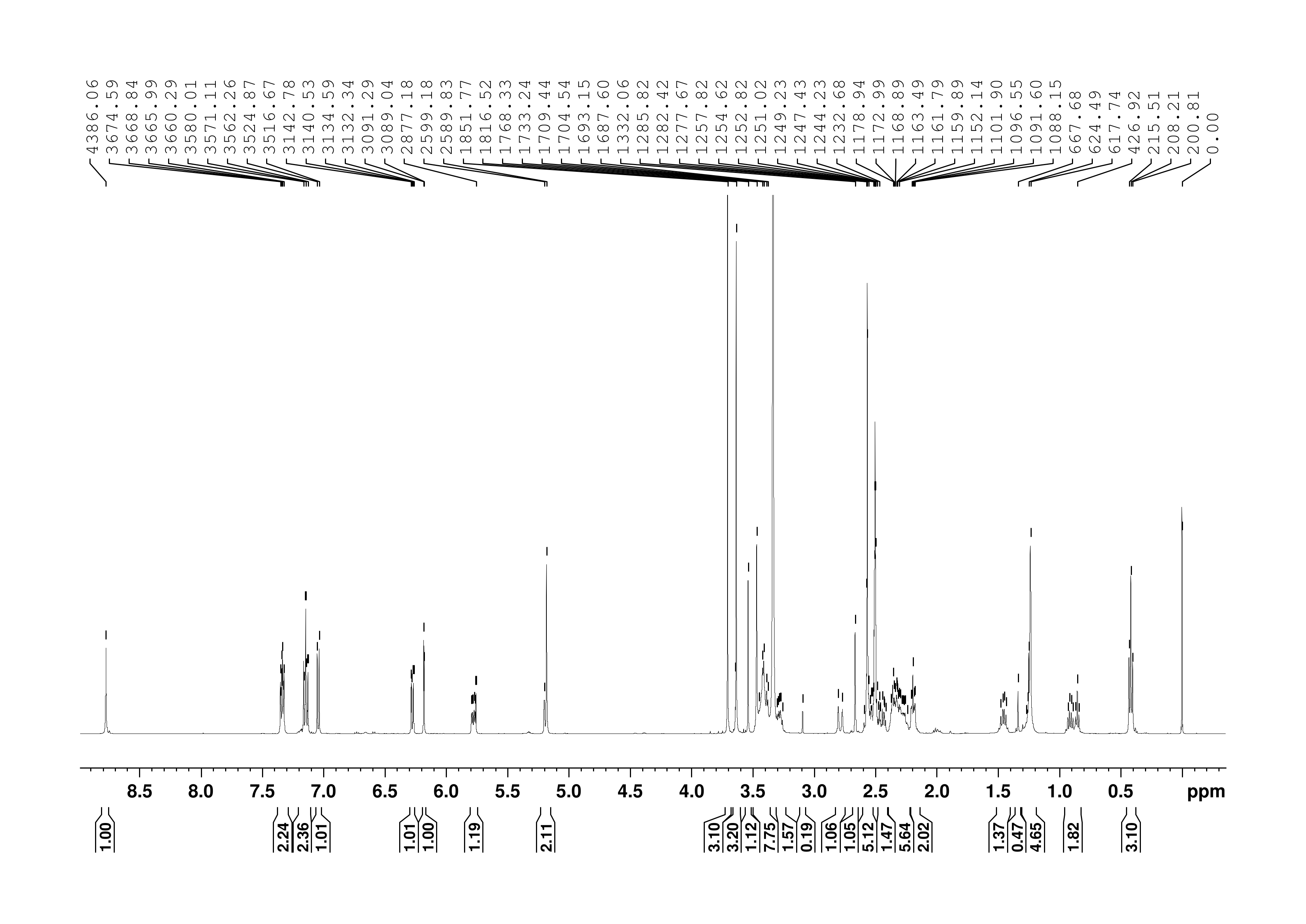


**Figure S111.** ^1^H NMR spectrum of compound **30.**


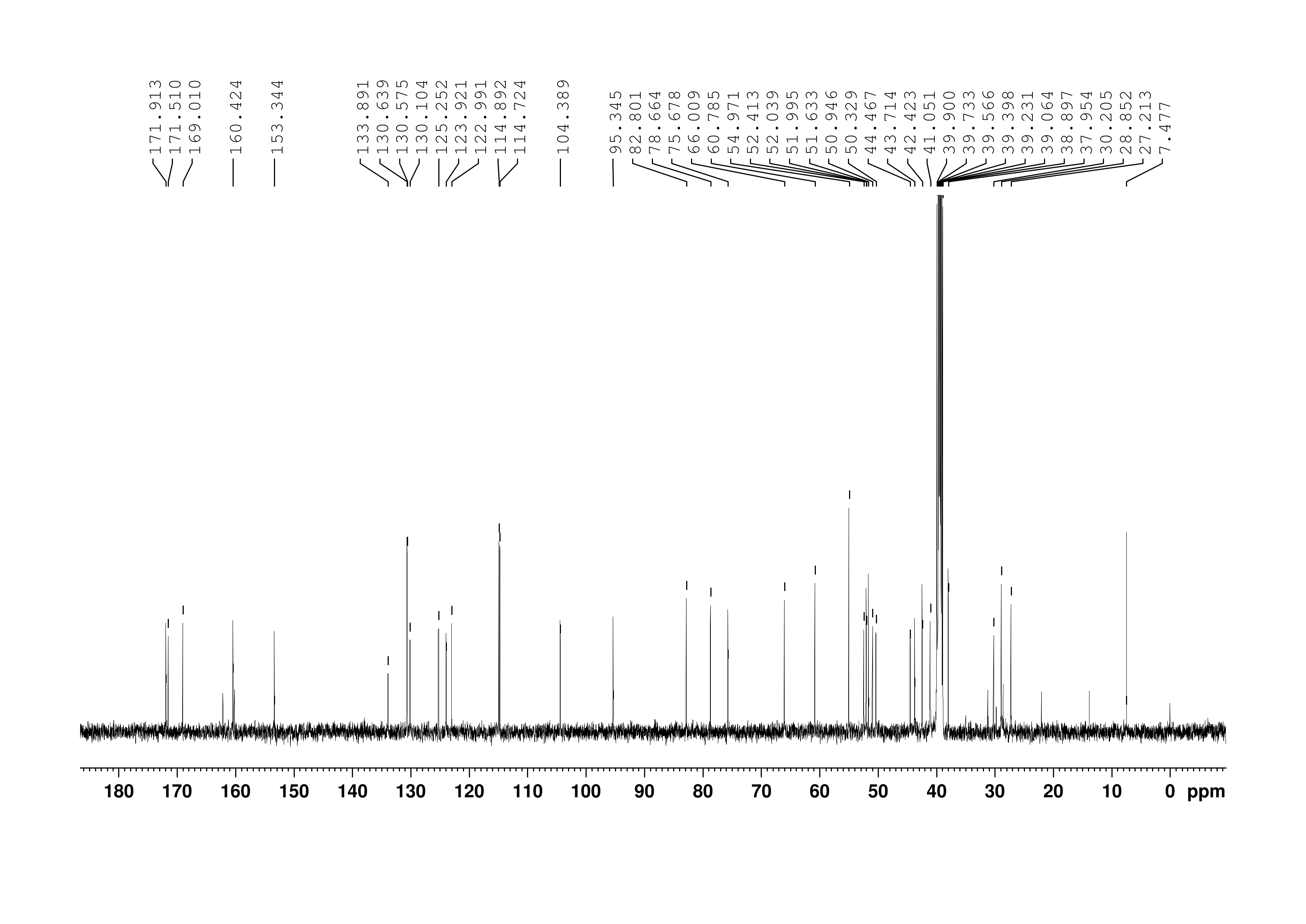


**Figure S112.** ^13^C NMR spectrum of compound **30.**


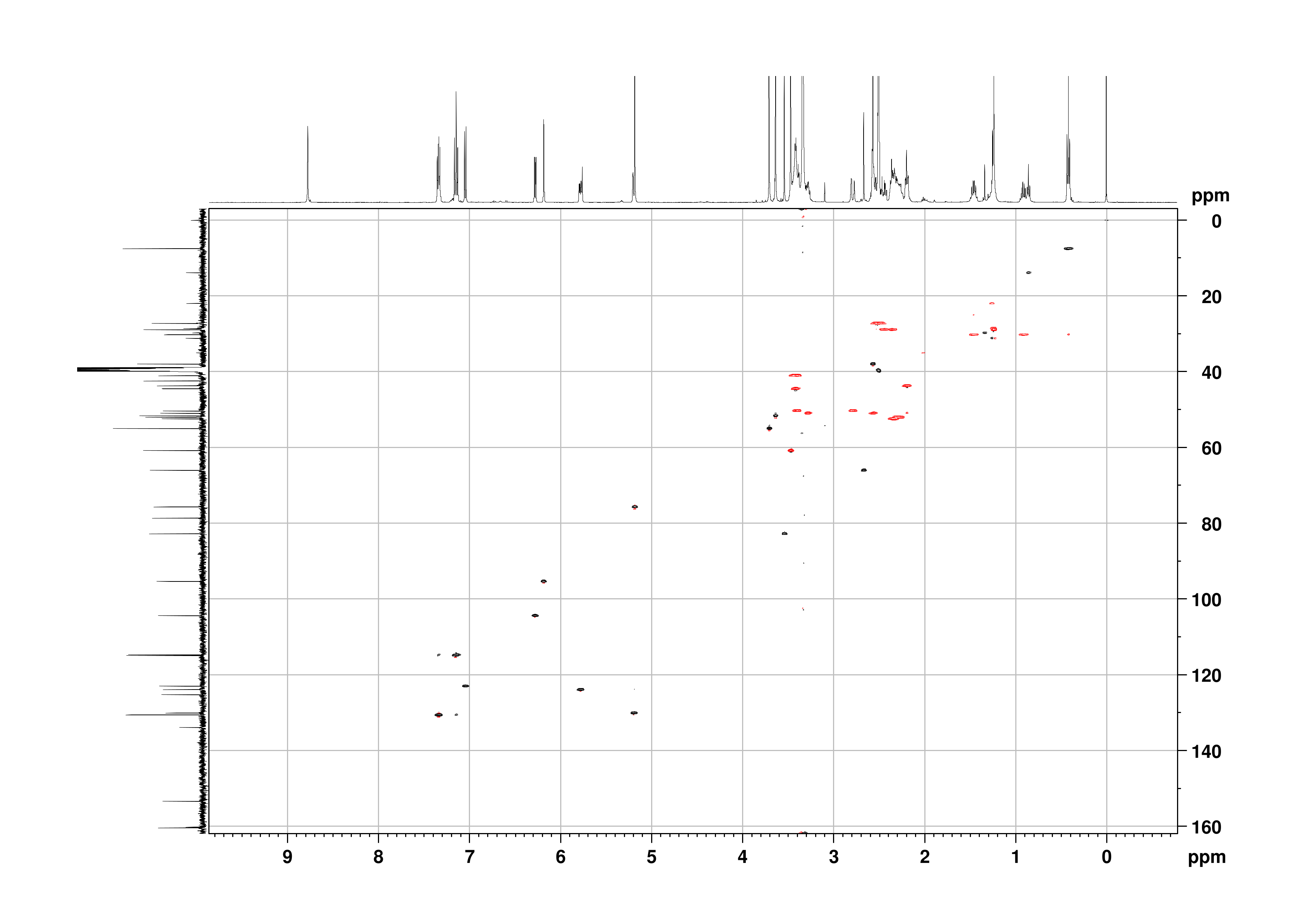


**Figure S113.** HSQC spectrum of compound **30.**


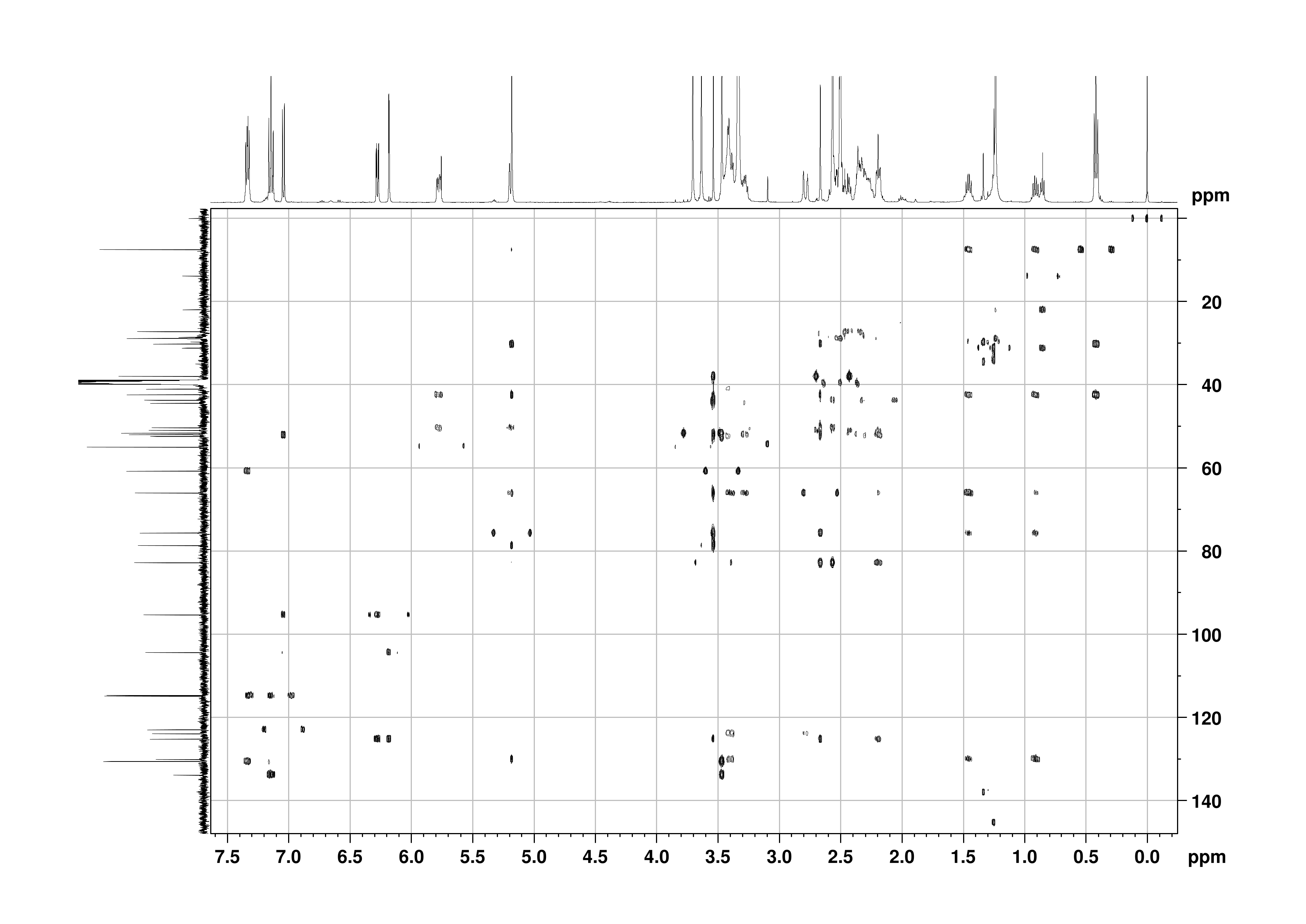


**Figure S114.** ^1^H-^13^C HMBC spectrum of compound **30.**


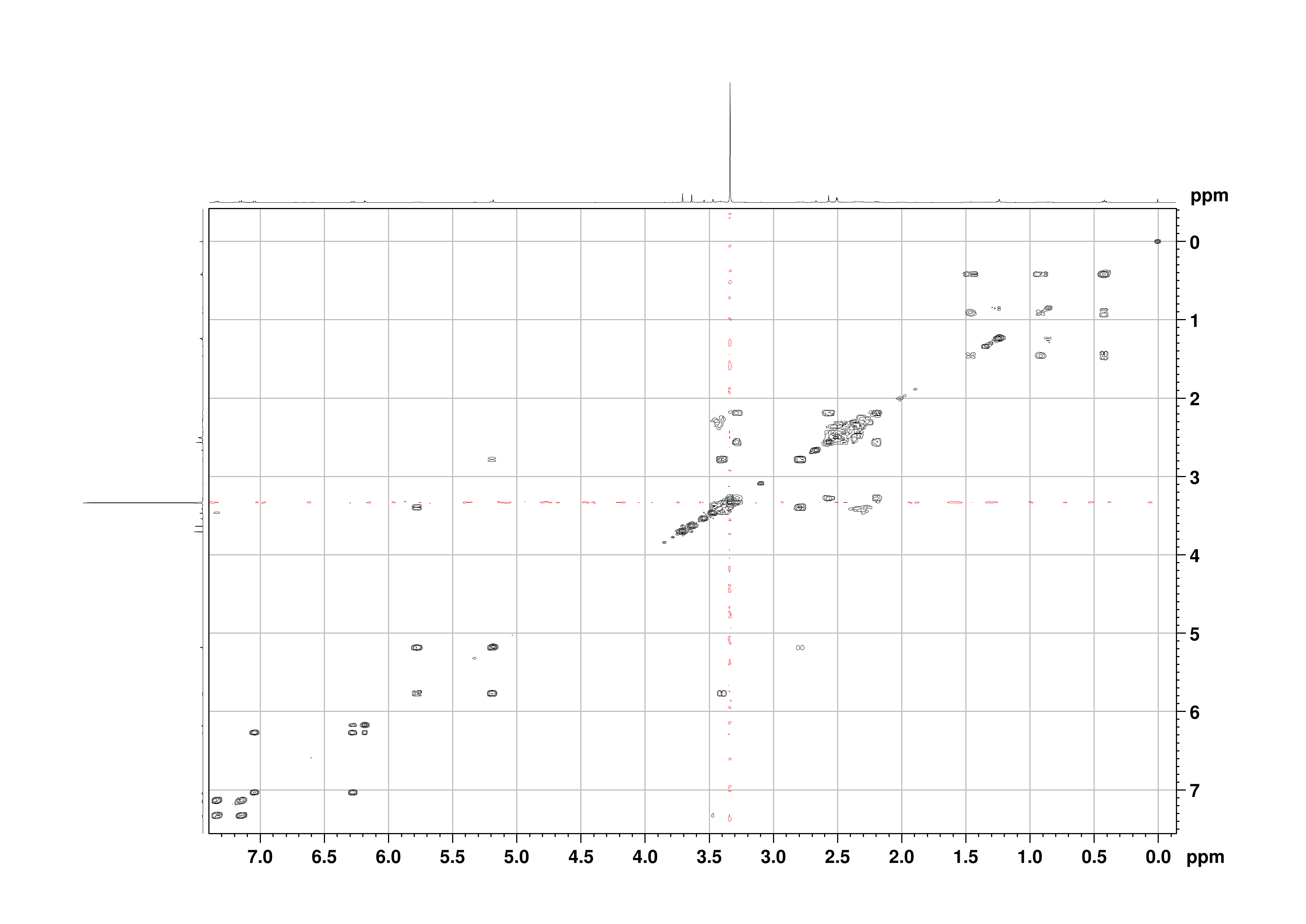


**Figure S115.** COSY spectrum of compound **30.**


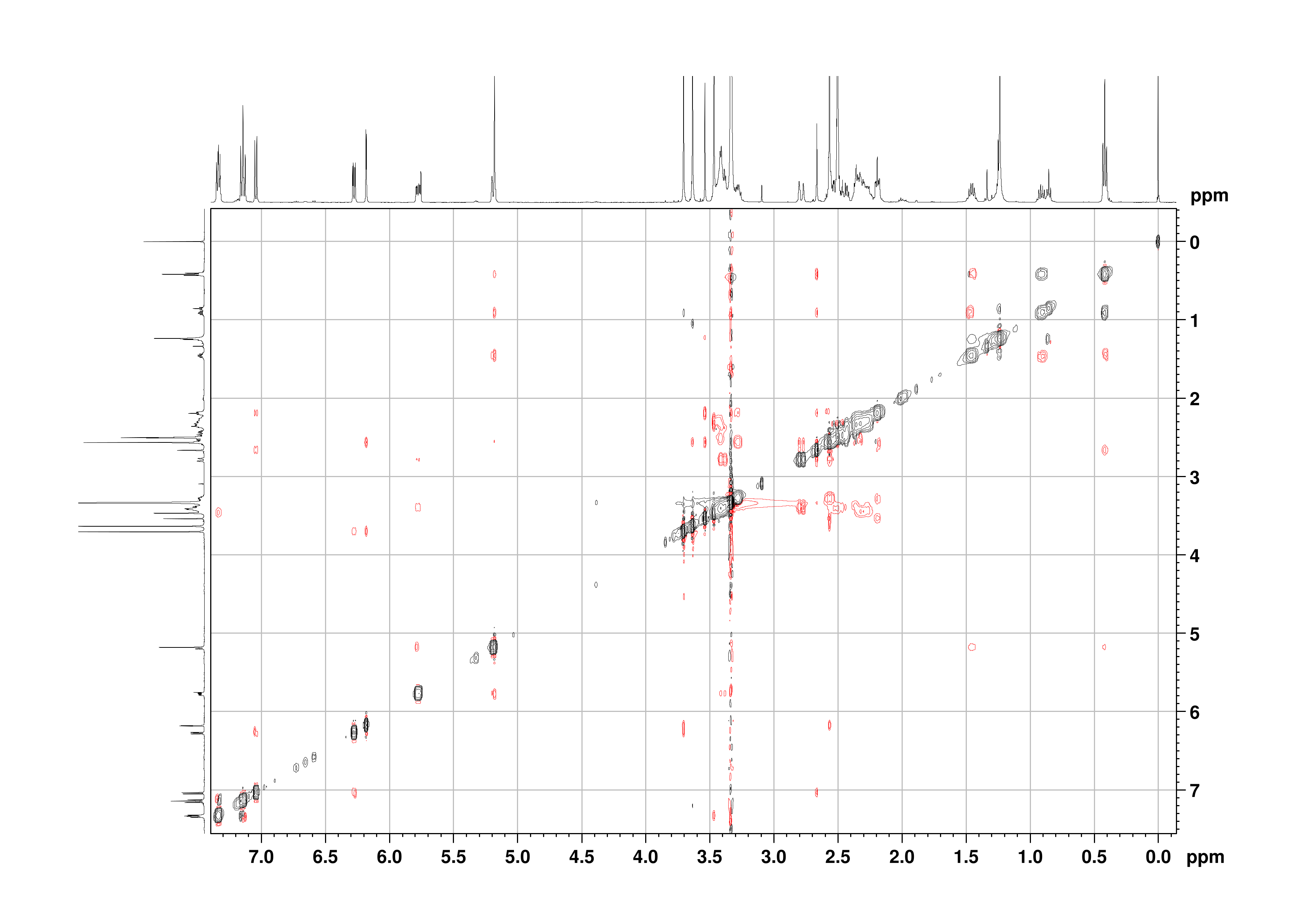


**Figure S116.** ROESY spectrum of compound **30.**

**Figure S117.** HRMS spectrum of compound **30.**

Product **31**

83 mg (54%). M.p.: 109-110 °C. TLC (DCM : MeOH = 10 : 1); *R_f_* = 0.84. IR (KBr) 2928, 1736, 1646, 1502, 1242, 1156, 1087, 823, 640, 548 cm^-1^. ^1^H NMR (599.8 MHz; CDCl_3_) *δ* (ppm): 0.48 (3H; t; *J* = 7.3 Hz; H_3_-18); 1.10 (1H; dq; *J* = 14.3, 7.1 Hz; H_x_-19); 1.60 (1H; dq; *J* = 14.3, 7.3 Hz; H_y_-19); 2.24-2.39 (6H; m; H_2_-6, H_2_-7’, H_2_-9’); 2.45-2.61 (4H; m; H_x_-5, H_x_-2’, H_x_-3’); 2.62-2.70 (5H; m; N(1)-CH_3_, H-21, H_y_-3’); 2.72-2.85 (2H; m; H_x_-3, H_y_-2’); 3.38-3.51 (4H; m; H_y_-3, H_y_-5, H_2_-6’ v, H_2_-10’); 3.58 (3H; br s; H_2_-6’ v, H_2_-10’); 3.74 (1H; s; H-2); 3.77 (3H; s; C(16)-COOCH_3_); 3.78 (3H; s; C(11)-OCH_3_); 4.21 (1H; s; H-11’); 5.32 (1H; br d; *J* = 10.1 Hz; H-15); 5.46 (1H; s; H-17); 5.84 (1H; ddd; *J* = 10.1, 4.6, 1.3 Hz; H-14); 6.06 (1H; d; *J* = 2.0 Hz; H-12); 6.29 (1H; dd; *J* = 8.1, 2.0 Hz; H-10); 6.88 (1H; d; *J* = 8.1 Hz; H-9); 6.97 (4H; t; *J* = 8.5 Hz; 4×C(11’)-PhF: H_meta_); 7.33 (4H; dd; *J* = 8.2, 5.9 Hz; 4×C(11’)-PhF: H_orto_); 9.53 (1H; br s; C(16)-OH). ^13^C NMR (150.8 MHz; CDCl_3_) *δ* (ppm): 7.7 (C-18); 27.8 (C-3’); 29.2 (C-2’); 30.7 (C-19); 38.2 (N(1)-CH_3_); 41.8 (C-6’ v, C-10’); 42.9 (C-20); 44.5 (C-6); 45.3 (C-6’ v, C-10’); 51.0 (C-3); 51.4 (C-7’ v, C-9’); 51.8 (C-5, C-7’ v, C-9’); 52.2 (C(16)-COOCH_3_); 52.8 (C-7); 55.3 (C(11)-OCH_3_); 66.9 (C-21); 74.2 (C-11’); 76.4 (C-17); 79.6 (C-16); 83.4 (C-2); 95.8 (C-12); 104.5 (C-10); 115.5 (d; *J* = 21.5 Hz; 4×C(11’)-PhF: C_meta_); 122.7 (C-9); 123.9 (C-14); 125.0 (C-8); 129.2 (d; *J* = 8.0 Hz; 4×C(11’)-PhF: C_orto_); 130.6 (C-15); 137.7 (2×C(11’)-PhF: C_ipszo_); 153.7 (C-13); 161.1 (C-11); 161.9 (d; *J* = 246.1 Hz; 2×C(11’)-PhF: C_para_); 169.5 (C-4’); 171.9 (C(16)-COOCH_3_); 172.9 (C-1’). HRMS: M+H=785.37184 (delta = -0.3 ppm; C_44_H_51_O_7_N_4_F_2_).

**Figure S118.** The skeleton numbering of compound **31** used for NMR assignment.


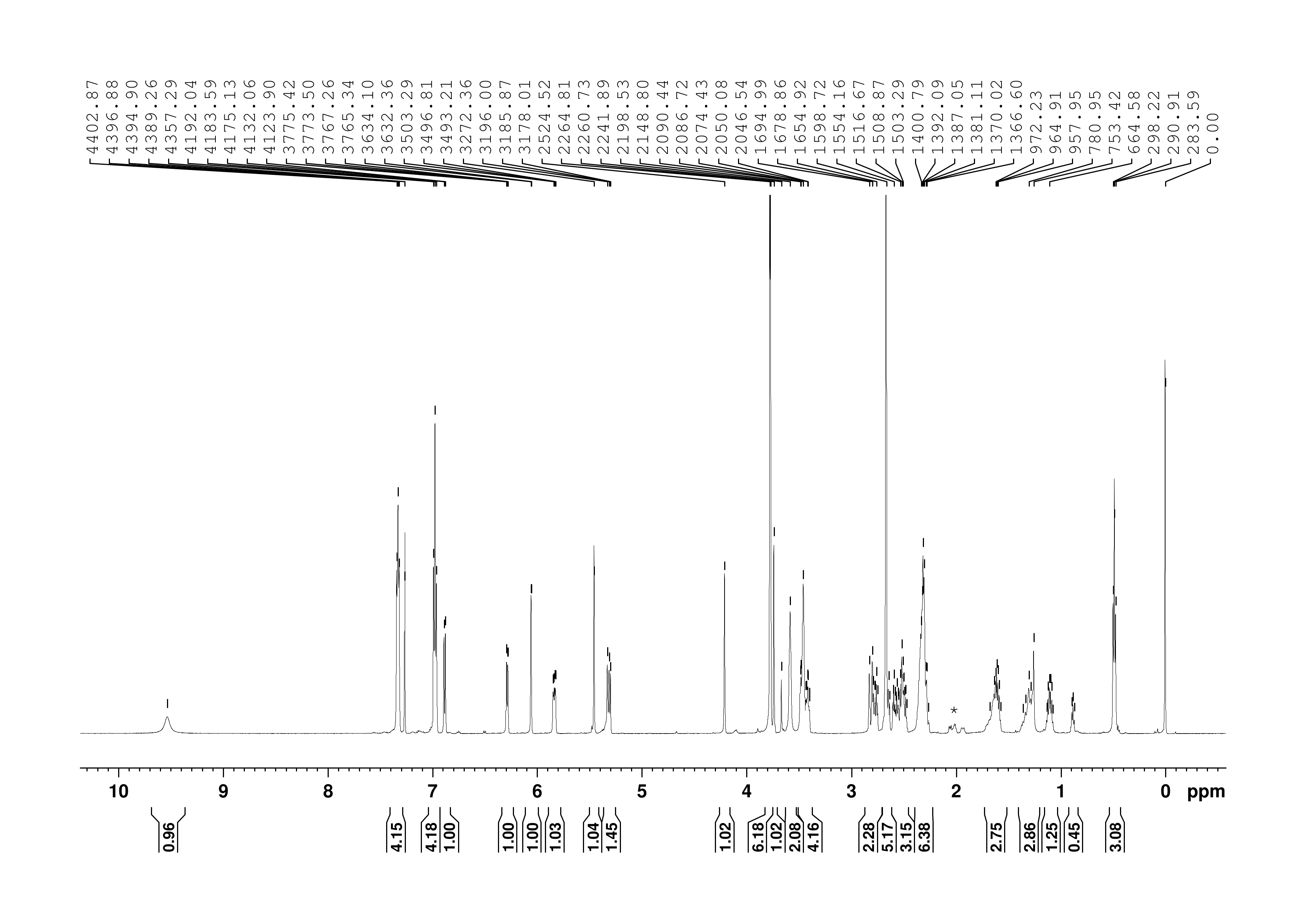


**Figure S119.** ^1^H NMR spectrum of compound **31.**


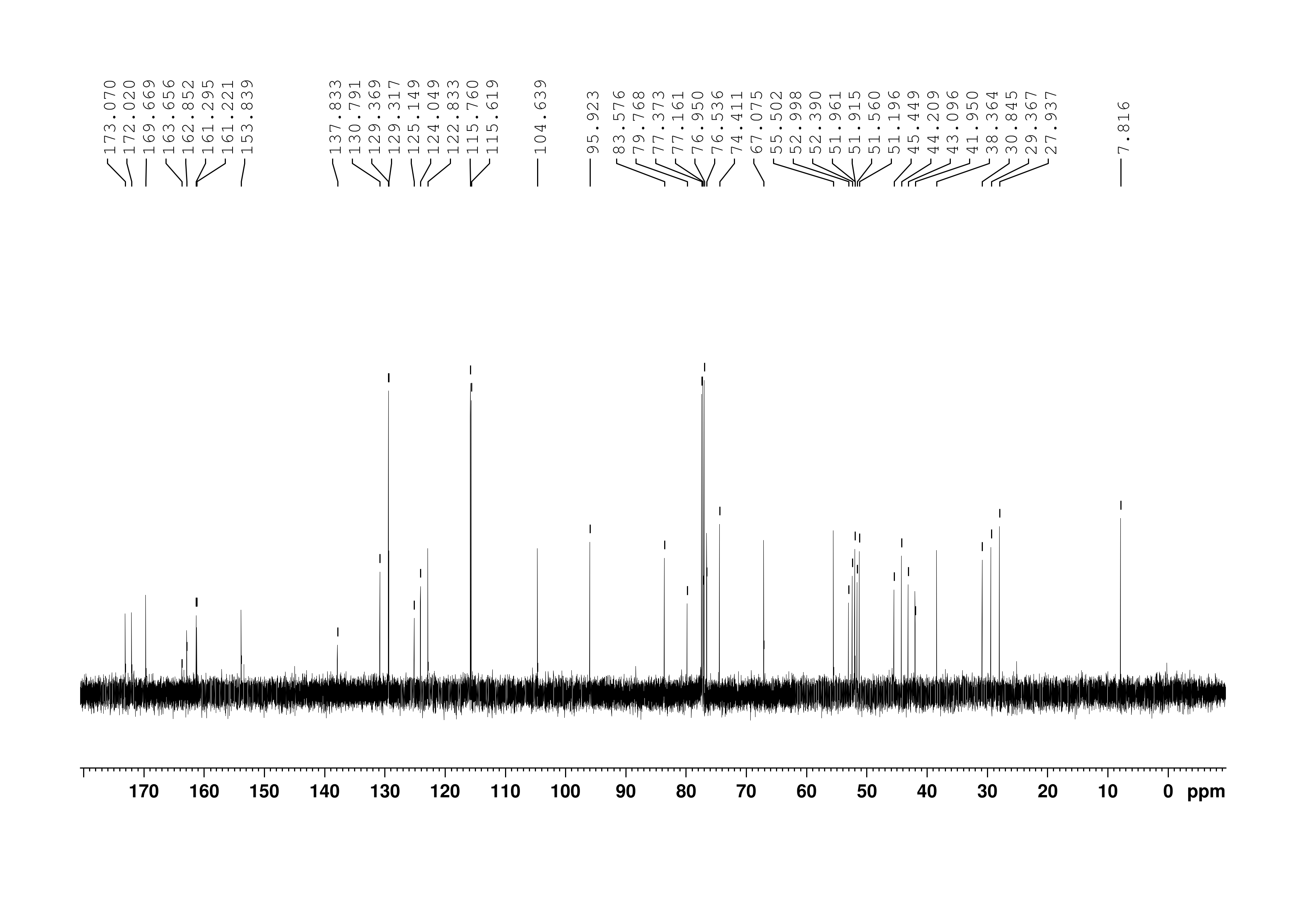


**Figure S120.** ^13^C NMR spectrum of compound **31.**


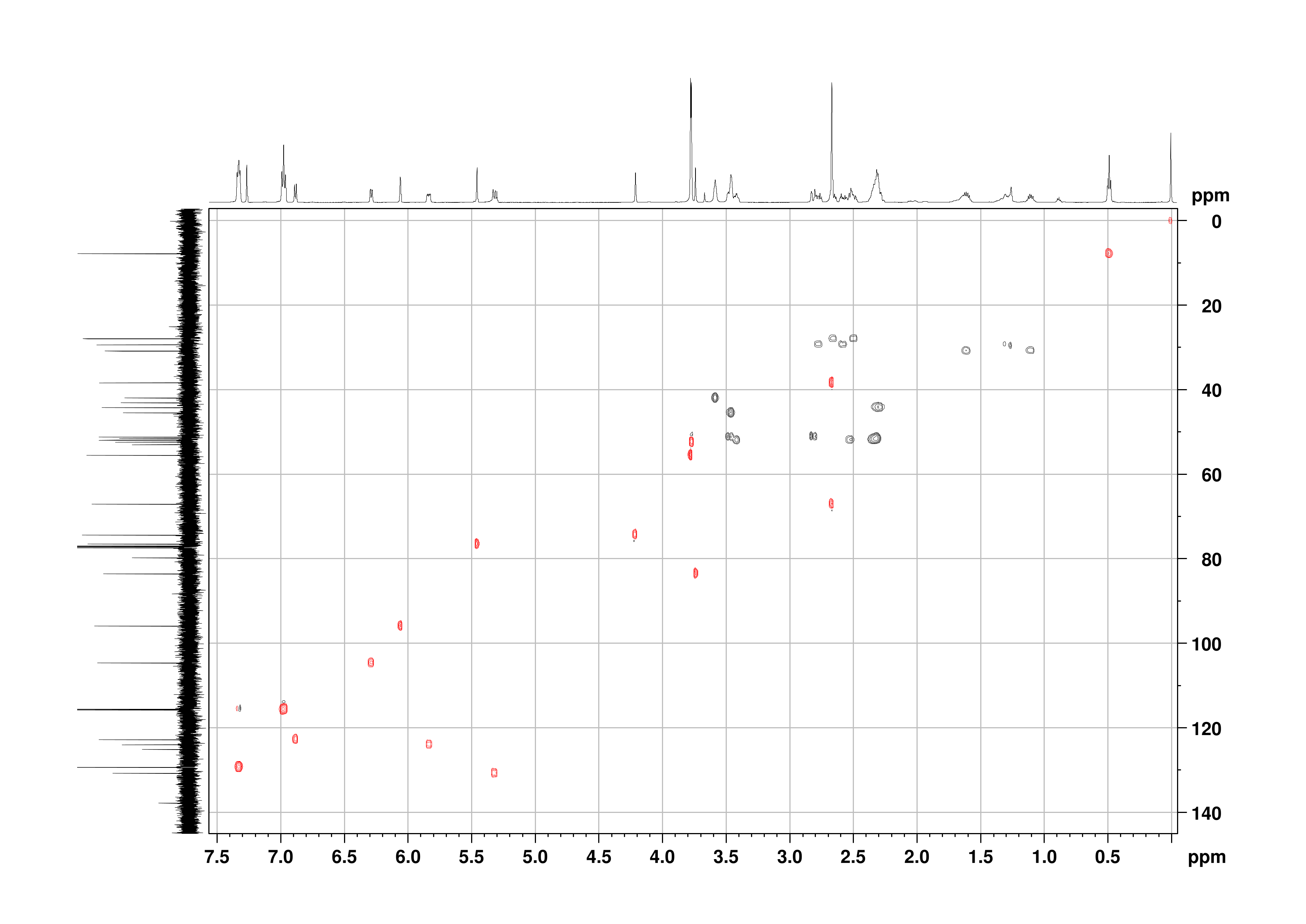


**Figure S121.** HSQC spectrum of compound **31.**


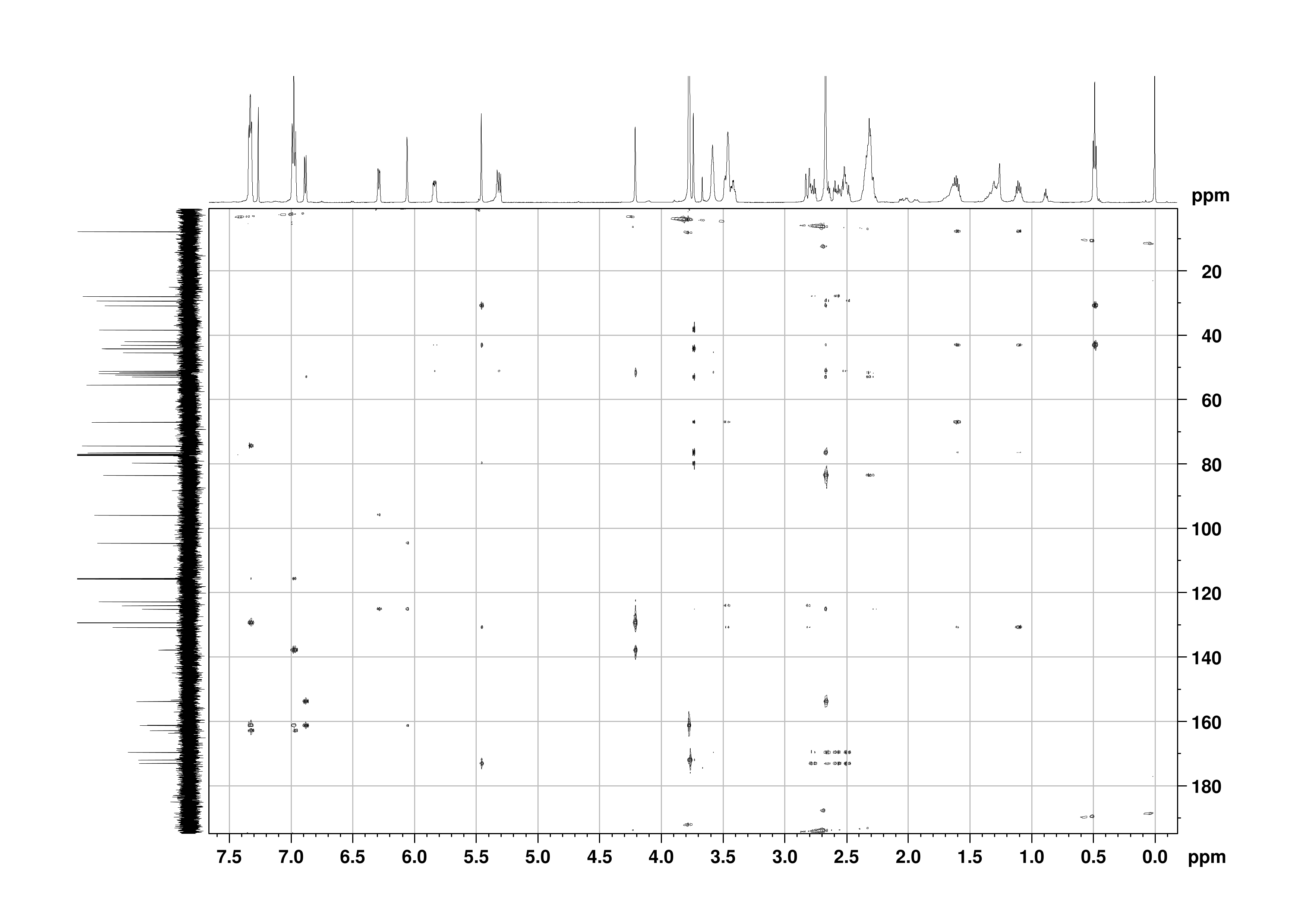


**Figure S122.** ^1^H-^13^C HMBC spectrum of compound **31.**


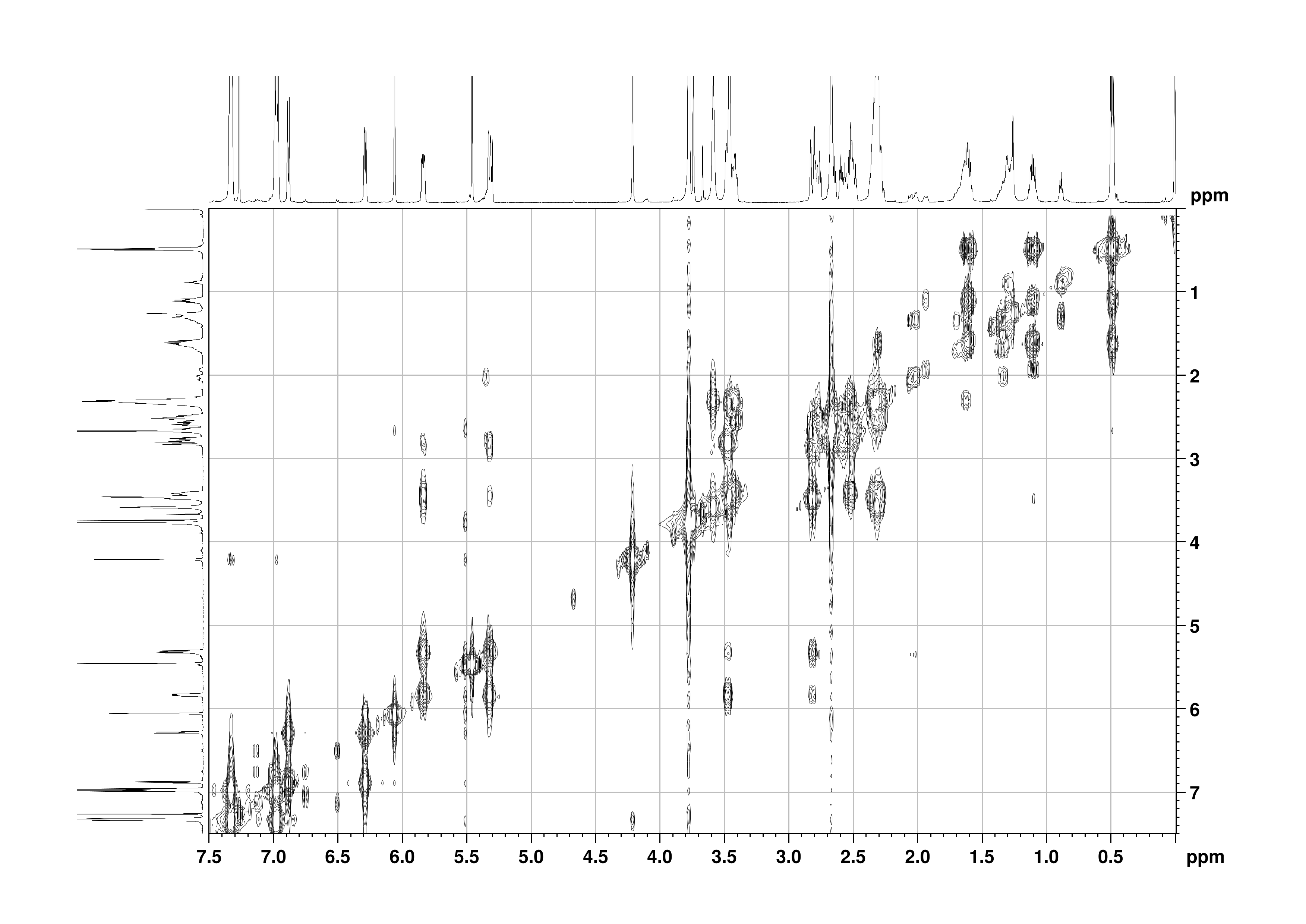


**Figure S123.** COSY spectrum of compound **31.**

**Figure S124.** HRMS spectrum of compound **31.**

Product **32**

55 mg (42%). M.p.: 85-86 °C. TLC (DCM : MeOH = 10 : 1); R*_f_* = 0.45. IR (KBr) 2921, 1735, 1617, 1426, 1243, 1222, 1164, 1007, 756 cm^-1^. ^1^H NMR (599.8 MHz; DMSO-*d*_6_) *δ* (ppm): 0.42 (3H; t; *J* = 7.4 Hz; H_3_-18); 0.96 (1H; dq; *J* = 14.1, 7.3 Hz; H_x_-19); 1.47 (1H; dq; *J* = 14.1, 7.4 Hz; H_y_-19); 2.15-2.24 (2H; m; H_2_-6.); 2.36-2.43 (1H; m; H_x_-2’); 2.46-2.64 (7H; m; N(1)-CH_3_, H_x_-5, H_y_-2’, H_2_-3’); 2.67 (1H; s; H-21); 2.79 (1H; br d; *J* = 16.6 Hz; H_x_-3); 3.24-3.36 (1H; m; H_y_-5); 3.41 (1H; ~dd; *J* = 16.4, 5.0 Hz; H_y_-3); 3.48-3.57 (9H; m; H-2, H-6’, H-7’, H-9’, H-10’); 3.65 (3H; s; C(16)-COOCH_3_); 3.70 (3H; s; C(11)-OCH_3_); 5.19-5.23 (2H; m; H-15, H-17); 5.81 (1H; ddd; *J* = 10.1, 4.8, 1.4 Hz; H-14); 6.18 (1H; d; *J* = 2.3 Hz; H-12); 6.28 (1H; dd; *J* = 8.2, 2.3 Hz; H-10); 6.64 (1H; dd; *J* = 3.5, 1.8 Hz; H-15’); 7.02 (1H; dd; *J* = 3.5, 0.7 Hz; H-16’); 7.04 (1H; d; *J* = 8.2 Hz; H-9); 7.85 (1H; dd; *J* = 1.8, 0.7 Hz; H-14’); 8.79 (1H; s; C(16)-OH). ^13^C NMR (150.8 MHz; DMSO-*d*_6_) *δ* (ppm): 7.5 (C-18); 27.2 (C-3’); 28.8 (C-2’); 30.3 (C-19); 38.0 (N(1)-CH_3_); 42.4 (C-20); 43.7 (C-6); 50.3 (C-3); 51.0 (C-5); 51.7 (C(16)-COOCH_3_); 52.0 (C-7); 55.0 (C(11)-OCH_3_); 66.0 (C-21); 75.8 (C-17); 78.7 (C-16); 82.8 (C-2); 95.4 (C-12); 104.4 (C-10); 111.2 (C-15’); 115.7 (C-16’); 123.0 (C-9); 124.0 (C-14); 125.3 (C-8); 130.1 (C-15); 144.8 (C-14’); 146.7 (C-12’); 153.4 (C-13); 158.4 (C-11’); 160.4 (C-11); 169.4 (C-4’); 171.5 (C(16)-COOCH_3_); 171.9 (C-1’). HRMS: M+H=677.31676 (delta = -1.9 ppm; C_36_H_45_O_9_N_4_).

**Figure S125.** The skeleton numbering of compound **32** used for NMR assignment.


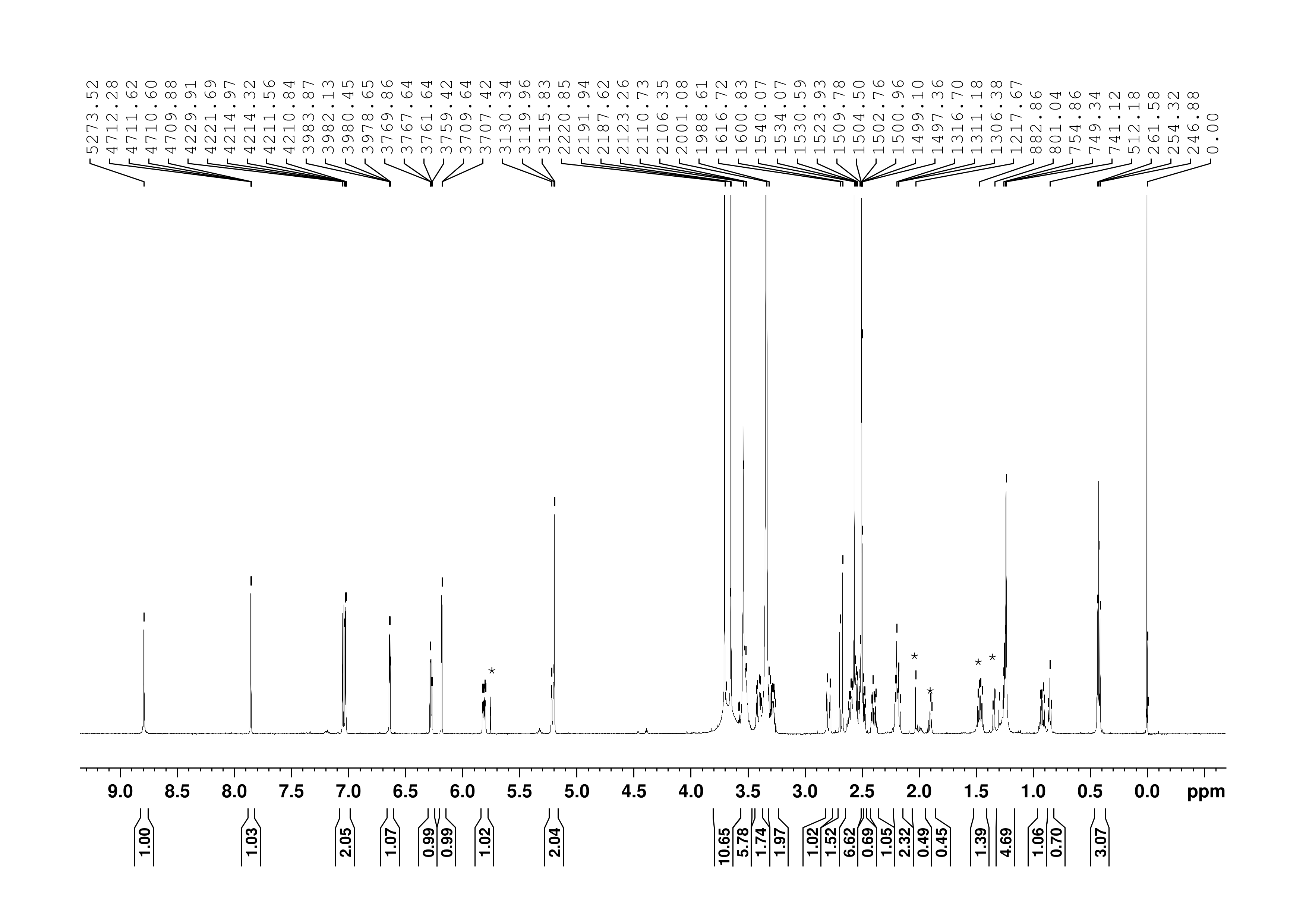


**Figure S126.** ^1^H NMR spectrum of compound **32.**


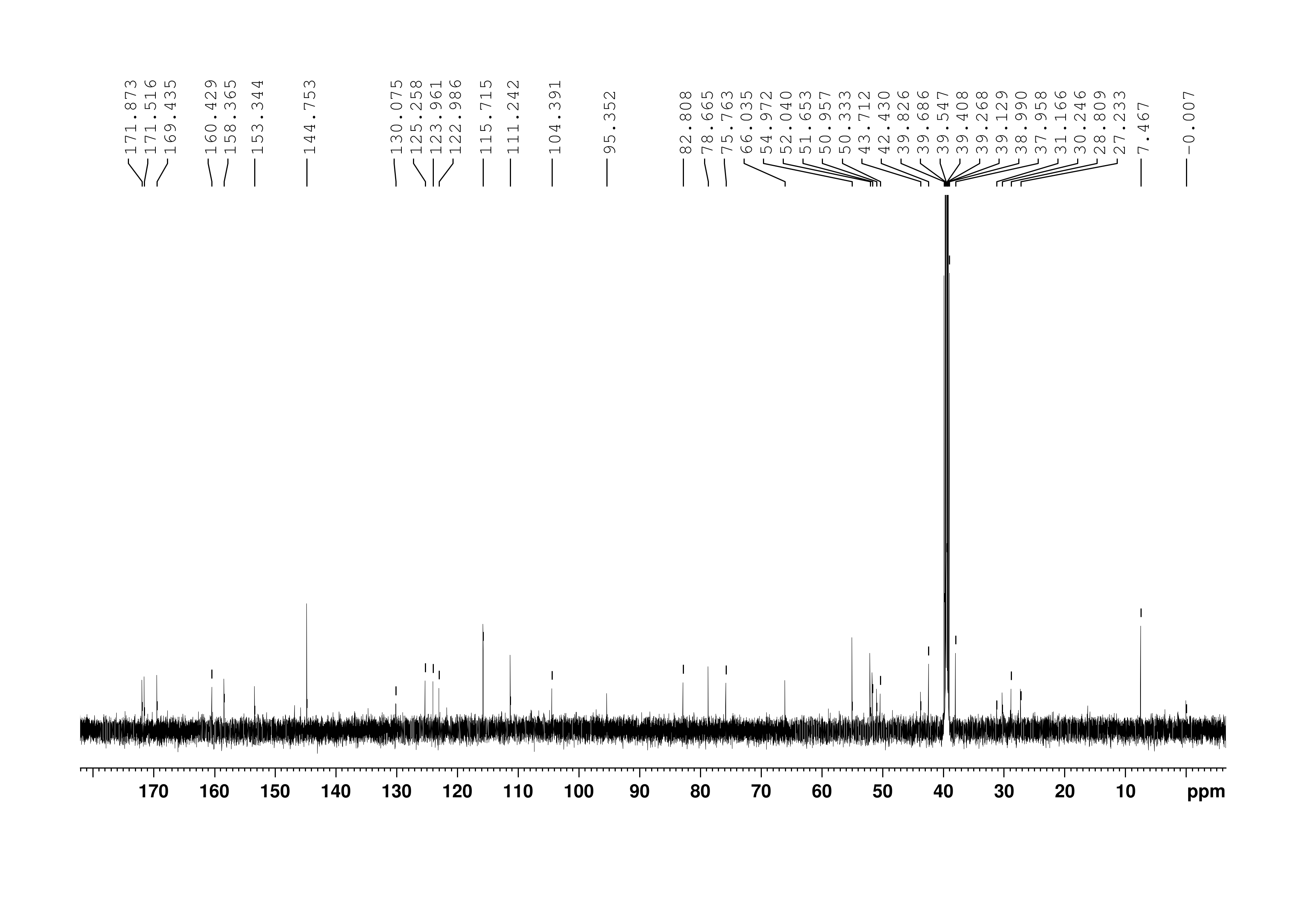


**Figure S127.** ^13^C NMR spectrum of compound **32.**


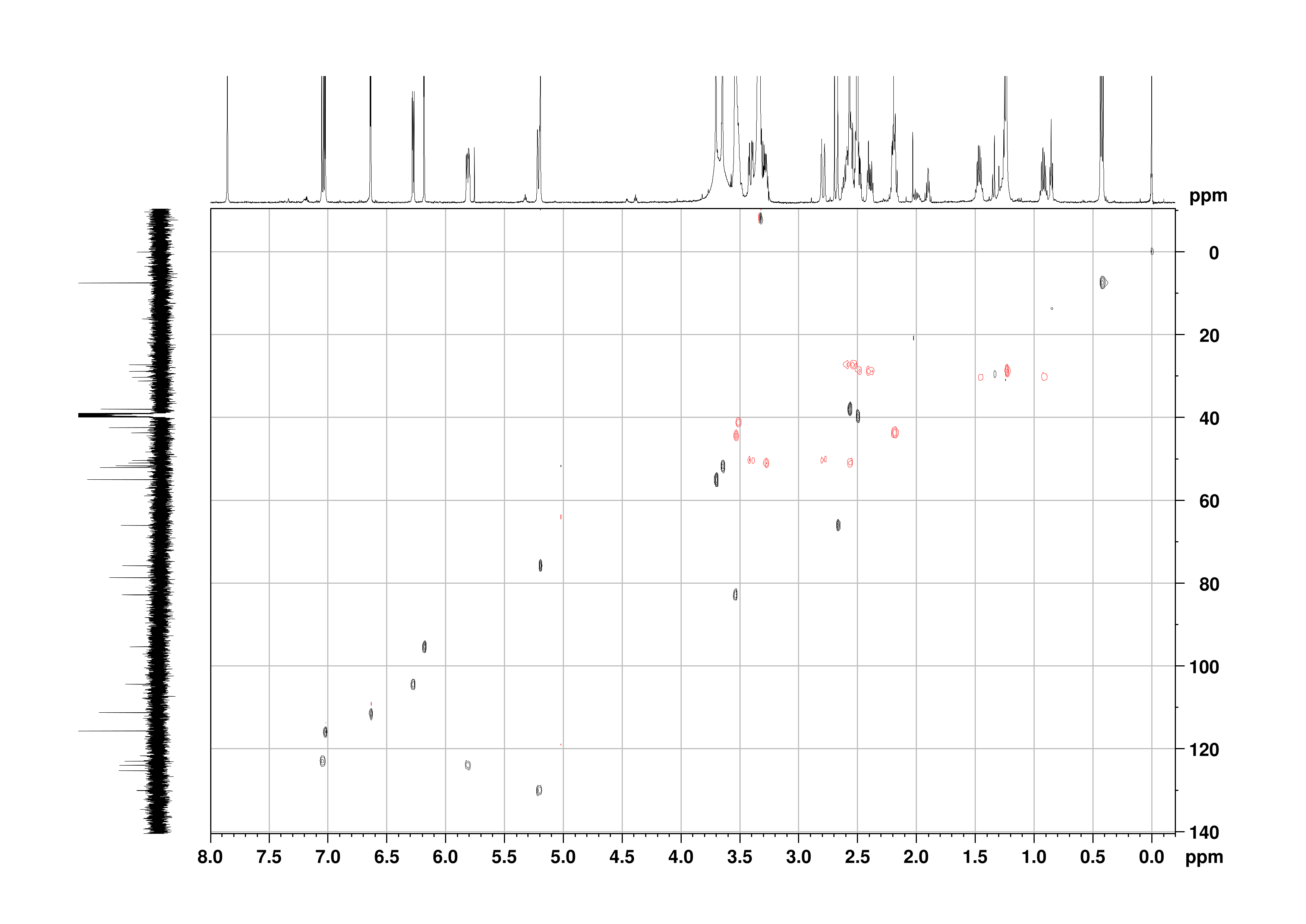


**Figure S128.** HSQC spectrum of compound **32.**


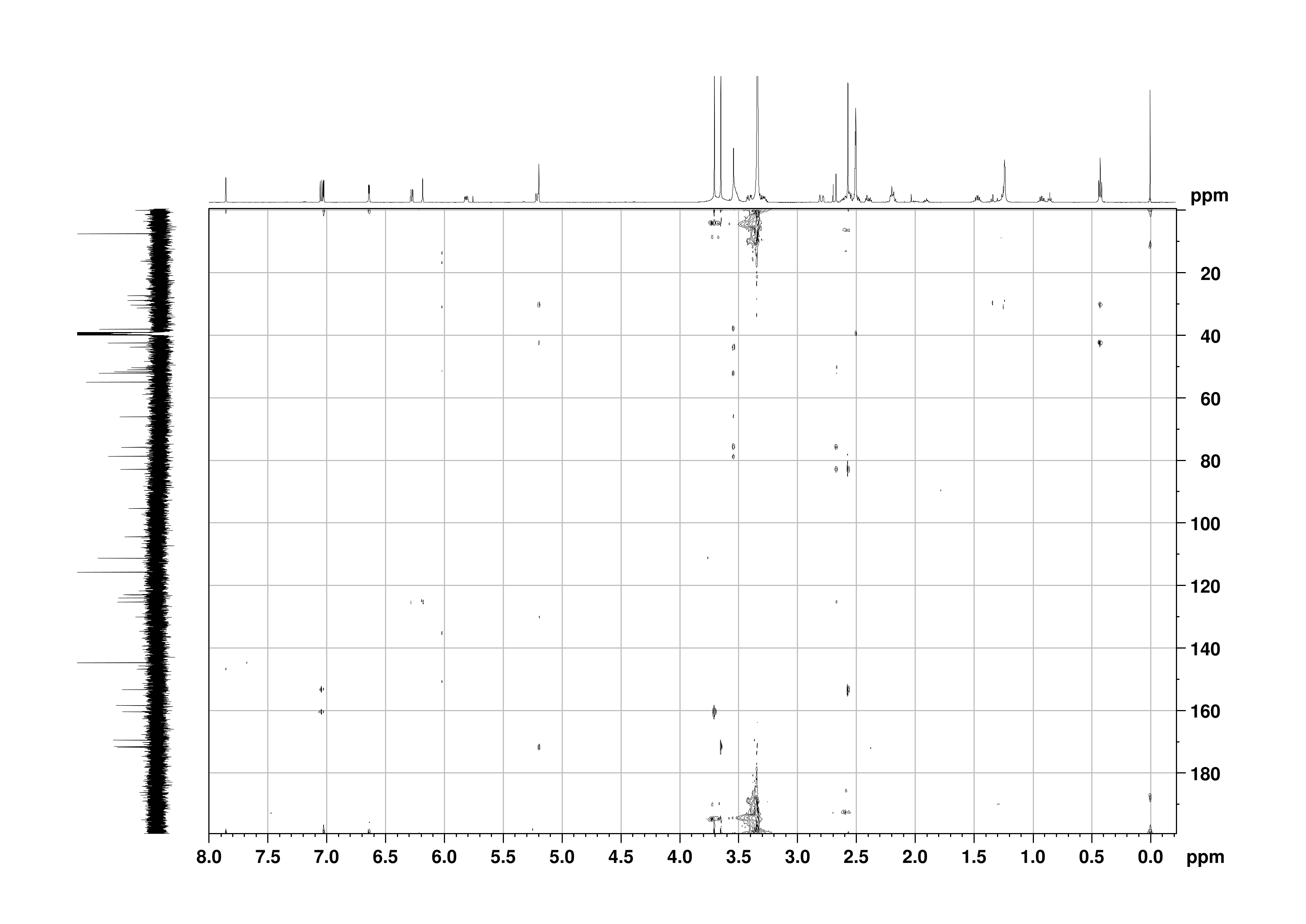


**Figure S129.** ^1^H-^13^C HMBC spectrum of compound **32.**


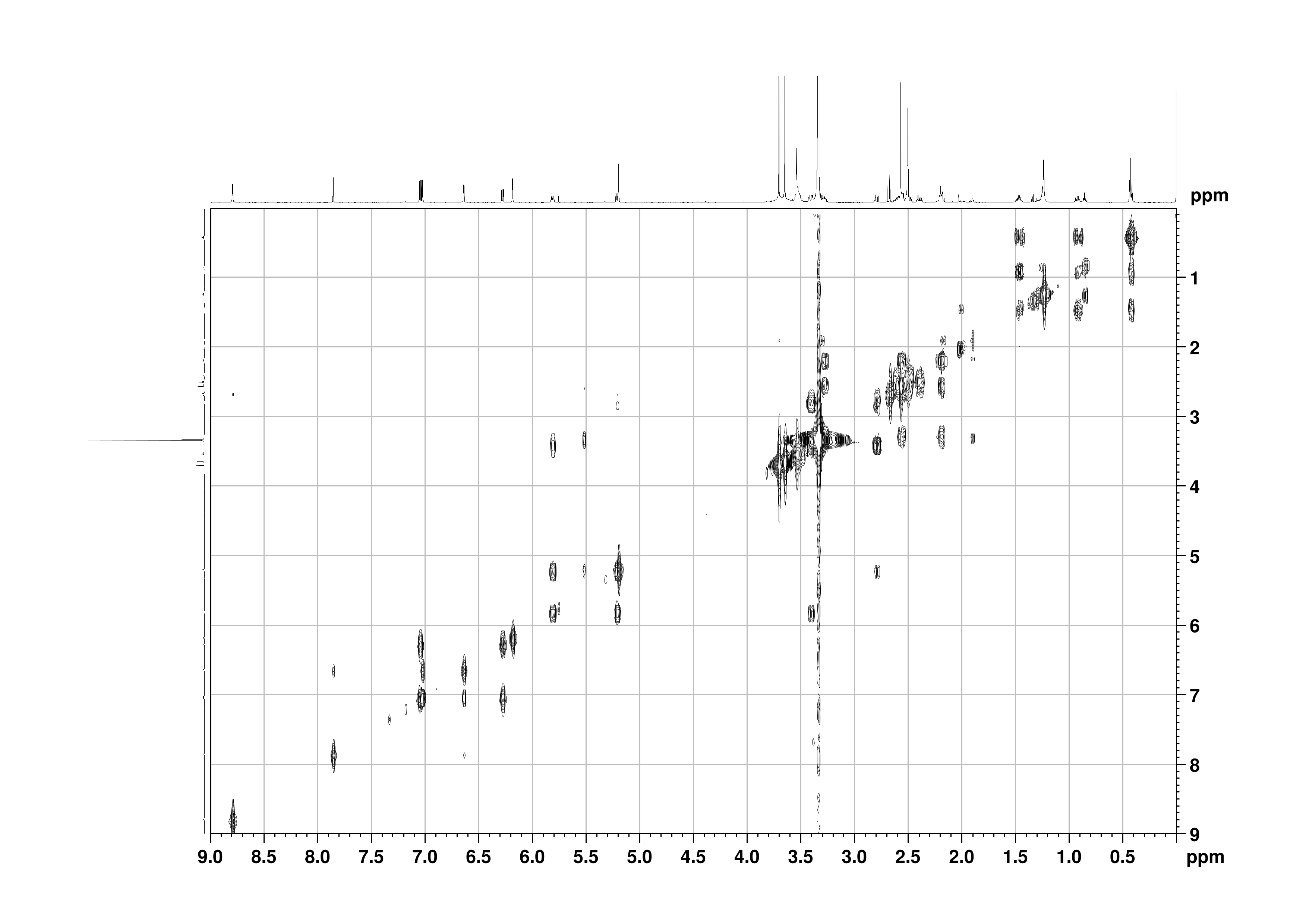


**Figure S130.** COSY spectrum of compound **32.**

**Figure S131.** HRMS spectrum of compound **32.**

**S.2. NCI60 Screening Results**

**Table S1.** Growth percent rates of vindoline (**1**), 10-chloroacetamidovindoline (**12**), and compounds **16**-**21** at the concentration of 10 μ*M* against 60 human cancer cell lines *in vitro*. All data with greater than 50% loss of cells are highlighted in bold.

|  | **1** | **12** | **16** | **17** | **18** | **19** | **20** | **21** |
| --- | --- | --- | --- | --- | --- | --- | --- | --- |
| Leukemia |  |  |  |  |  |  |  |  |
| CCRF-CEM | 102.07 | 12.62 | 112.44 | -7.07 | 39.39 | 104.93 | -18.88 | 102.88 |
| HL-60(TB) | 96.65 | 37.93 | 97.46 | 1.98 | 42.64 | 101.12 | -10.79 | 113.51 |
| K-562 | 89.30 | 36.35 | 85.80 | 11.39 | 43.77 | 74.02 | 17.02 | 107.53 |
| MOLT-4 | 92.69 | -26.85 | 107.53 | 8.66 | 16.74 | 77.99 | 1.17 | 101.34 |
| RPMI-8226 | 104.95 | -24.70 | 102.29 | -14.07 | 17.43 | 94.11 | -2.68 | 103.35 |
| SR | n.d. | n.d. | 93.79 | -11.40 | 48.17 | 75.35 | 0.34 | 97.24 |
| Non-small Cell Lung Cancer |  |  |  |  |  |  |  |  |
| A549/ATCC | 100.75 | 64.05 | 97.13 | 20.30 | 54.08 | 89.01 | 31.03 | 100.98 |
| EKVX | 91.96 | 46.60 | 94.75 | 21.33 | 61.61 | 88.85 | 16.20 | 99.17 |
| HOP-62 | 99.00 | **-57.61** | 104.50 | -38.83 | 86.12 | 106.10 | 60.29 | 100.45 |
| HOP-92 | 108.69 | 34.30 | n.d. | n.d. | n.d. | n.d. | n.d. | n.d. |
| NCI-H226 | 92.28 | 43.45 | 100.65 | **-61.85** | 67.67 | 97.77 | 24.09 | 110.86 |
| NCI-H23 | 95.33 | -4.42 | 103.09 | 15.19 | 73.10 | 102.94 | 37.99 | 105.95 |
| NCI-H322M | 109.09 | 58.90 | 100.73 | 39.09 | 64.63 | 98.55 | 42.81 | 110.29 |
| NCI-H460 | 120.46 | 51.01 | 142.35 | 9.27 | 62.08 | 129.18 | 17.33 | 108.36 |
| NCI-H522 | 99.65 | 8.28 | 90.20 | -24.41 | 55.18 | 93.46 | 10.98 | 98.19 |
| Colon Cancer |  |  |  |  |  |  |  |  |
| COLO 205 | 110.59 | 87.21 | 105.21 | **-74.55** | 56.46 | 100.78 | **-60.51** | 119.68 |
| HCC-2998 | 100.92 | 24.23 | 105.81 | **-69.94** | 79.11 | 102.49 | 3.93 | 126.11 |
| HCT-116 | 115.54 | 41.18 | 101.43 | 0.31 | 46.36 | 100.58 | 27.88 | 107.59 |
| HCT-15 | 102.64 | 50.09 | 101.47 | **-56.48** | 49.72 | 94.97 | 18.30 | 106.60 |
| HT29 | 108.21 | 54.15 | 104.13 | **-68.77** | 39.33 | 107.77 | -40.76 | 108.78 |
| KM12 | 108.48 | 41.52 | 111.73 | **-84.40** | 83.05 | 114.77 | 20.17 | 99.84 |
| SW-620 | 121.29 | 64.52 | 124.43 | 1.45 | 86.99 | 117.74 | 34.21 | 112.67 |
| CNS Cancer |  |  |  |  |  |  |  |  |
| SF-268 | 121.91 | -0.99 | 115.69 | -6.14 | 65.21 | 101.61 | 28.15 | 106.09 |
| SF-295 | 102.53 | 52.17 | 98.79 | **-70.16** | 75.44 | 99.85 | 13.78 | 109.91 |
| SF-539 | 115.91 | 4.74 | 100.87 | **-83.36** | 73.11 | 102.38 | **-61.07** | 114.18 |
| SNB-19 | 102.05 | 23.12 | 104.72 | 18.40 | 47.35 | 97.15 | 21.49 | 96.23 |
| SNB-75 | 119.24 | -8.81 | 95.62 | **-87.61** | 20.31 | 87.29 | **-60.47** | 90.97 |
| U251 | 95.93 | -47.91 | 97.39 | 7.24 | 74.85 | 103.25 | 31.73 | 118.37 |
| Melanoma |  |  |  |  |  |  |  |  |
| LOX IMVI | 95.28 | 11.46 | 95.19 | **-95.37** | 58.38 | 99.07 | **-61.03** | 98.54 |
| MALME-3M | 147.02 | -14.13 | 97.49 | **-89.50** | 73.31 | 101.68 | 0.00 | 103.83 |
| M14 | 104.65 | 37.02 | 100.90 | -31.06 | 67.17 | 101.66 | 49.66 | 109.76 |
| MDA-MB-435 | 104.36 | 39.87 | 114.34 | **-79.75** | 63.91 | 102.58 | 22.20 | 104.49 |
| SK-MEL-2 | 105.61 | **-69.58** | 107.65 | **-66.24** | 72.94 | 102.93 | -34.66 | 111.28 |
| SK-MEL-28 | 133.63 | -0.03 | 111.44 | **-67.56** | 90.77 | 109.35 | 51.16 | 126.48 |
| SK-MEL-5 | 89.22 | -17.58 | 102.09 | **-98.17** | 21.17 | 92.78 | **-76.17** | 105.19 |
| UACC-257 | 95.84 | -3.69 | -7.95 | **-65.31** | 80.62 | 98.38 | 37.85 | 105.52 |
| UACC-62 | n.d. | 22.13 | 98.94 | **-54.79** | 74.36 | 96.93 | 28.49 | 103.07 |
| Ovarian Cancer |  |  |  |  |  |  |  |  |
| IGROV1 | 145.14 | 27.54 | 111.60 | 12.70 | 71.29 | 113.25 | 44.86 | 126.84 |
| OVCAR-3 | 126.66 | -14.68 | 138.31 | -47.96 | 84.47 | 119.08 | 36.47 | 118.37 |
| OVCAR-4 | 135.95 | -17.12 | 108.51 | 20.38 | 63.12 | 101.96 | 31.45 | 107.77 |
| OVCAR-5 | 107.39 | n.d. | 101.74 | -41.58 | 108.02 | 110.75 | 66.93 | 120.94 |
| OVCAR-8 | 102.92 | 29.62 | 97.77 | 26.27 | 72.05 | 100.21 | 45.65 | 101.11 |
| NCI/ADR-RES | 106.61 | 79.36 | 103.89 | 0.40 | 61.90 | 97.26 | 20.95 | 112.03 |
| SK-OV-3 | 101.94 | 33.90 | 112.67 | 6.93 | 87.03 | 108.27 | 45.44 | 112.72 |
| Renal Cancer |  |  |  |  |  |  |  |  |
| 786-0 | 97.51 | 21.30 | 106.13 | 24.83 | 75.52 | 92.84 | 34.00 | 103.62 |
| A498 | 137.49 | 54.57 | 158.17 | -9.05 | 78.70 | 131.60 | 35.58 | 104.31 |
| ACHN | 105.27 | 37.26 | 104.49 | 3.15 | 67.31 | 101.91 | 11.42 | 109.22 |
| CAKI-1 | 105.39 | 43.81 | 89.48 | 10.76 | 33.03 | 69.27 | 27.67 | 97.46 |
| RXF 393 | 97.98 | -32.64 | 102.24 | -36.51 | 41.03 | 90.40 | -43.95 | 132.54 |
| SN12C | 100.31 | 11.03 | 108.76 | -16.99 | 59.34 | 103.20 | 26.95 | 106.83 |
| TK-10 | 97.53 | 24.81 | 102.85 | 33.44 | 81.48 | 106.82 | 37.65 | 109.43 |
| UO-31 | 84.63 | n.d. | 89.38 | 0.08 | 50.05 | 89.14 | 27.16 | 89.28 |
| Prostate Cancer |  |  |  |  |  |  |  |  |
| PC-3 | 98.60 | 24.51 | 93.07 | n.d. | n.d. | 73.62 | n.d. | n.d. |
| DU-145 | 118.76 | 43.85 | 114.53 | 24.10 | 71.60 | 110.84 | 37.11 | 110.07 |
| Breast Cancer |  |  |  |  |  |  |  |  |
| MCF7 | 89.87 | 2.74 | 84.28 | 0.95 | 32.61 | 79.98 | 5.47 | 98.28 |
| MDA-MB-231/ATCC | 113.10 | n.d. | 102.40 | **-86.16** | 43.71 | 102.05 | -4.47 | 98.77 |
| HS 578T | 97.99 | -16.78 | 111.15 | -3.36 | 62.53 | 109.79 | 15.54 | 121.20 |
| BT-549 | 105.88 | 14.73 | 112.69 | **-50.37** | 47.02 | 101.00 | -37.53 | 116.59 |
| T-47D | n.d. | n.d. | 104.12 | 9.79 | 51.44 | 93.04 | 10.18 | 111.14 |
| MDA-MB-468 | 133.75 | -40.78 | n.d. | **-62.45** | 39.76 | n.d. | 10.89 | 120.09 |
| Mean |  |  |  |  |  |  |  |  |
|  | 107.27 | 18.14 | 103.07 | -24.70 | 60.61 | 99.58 | 12.18 | 108.17 |

**Table S2.** Growth percent rates of 17-desacetylvindoline (**13**), 17-(*O*-4-bromobutanoyl)vindoline (**14**), and compounds **3** and **22**-**26** at the concentration of 10 μ*M* against 60 human cancer cell lines *in vitro*. All data with greater than 50% loss of cells are highlighted in bold.

|  | **13** | **14** | **3** | **22** | **23** | **24** | **25** | **26** |
| --- | --- | --- | --- | --- | --- | --- | --- | --- |
| Leukemia |  |  |  |  |  |  |  |  |
| CCRF-CEM | 103.20 | 99.81 | 107.83 | 3.97 | **-63.07** | 12.66 | -29.47 | 102.95 |
| HL-60(TB) | 118.91 | 90.48 | 91.01 | 7.90 | **-65.80** | 7.68 | **-56.19** | 93.75 |
| K-562 | 101.22 | 79.68 | 66.94 | 6.96 | **-73.29** | 0.40 | -1.70 | 76.73 |
| MOLT-4 | 100.46 | 76.11 | 102.25 | 9.30 | **-68.45** | 6.28 | -31.92 | 77.30 |
| RPMI-8226 | 99.38 | 91.39 | 112.08 | -9.29 | **-58.29** | 20.06 | **-50.14** | 69.23 |
| SR | n.d. | 98.39 | n.d. | 14.18 | **-55.21** | 4.82 | -43.61 | 75.40 |
| Non-small Cell Lung Cancer |  |  |  |  |  |  |  |  |
| A549/ATCC | 102.73 | 89.37 | 98.20 | 4.54 | **-67.76** | 51.25 | **-65.37** | 92.65 |
| EKVX | 98.90 | 91.25 | 96.52 | -49.86 | **-75.13** | 40.34 | **-89.80** | 87.82 |
| HOP-62 | 101.61 | 100.58 | 79.92 | 0.03 | **-86.69** | 70.10 | **-77.17** | 106.64 |
| HOP-92 | 88.62 | 77.57 | 80.93 | -30.89 | **-70.70** | n.d. | n.d. | n.d. |
| NCI-H226 | 88.50 | 95.99 | n.d. | **-50.74** | -32.31 | 50.17 | **-74.80** | 94.94 |
| NCI-H23 | 93.04 | 93.10 | 89.99 | **-61.07** | **-71.28** | 74.43 | **-92.67** | 108.81 |
| NCI-H322M | 92.80 | 100.58 | 96.92 | 50.53 | -48.16 | 71.68 | **-71.67** | 107.67 |
| NCI-H460 | 106.50 | 97.82 | 97.66 | -36.90 | **-84.73** | 30.57 | **-70.36** | 112.01 |
| NCI-H522 | 93.97 | 79.00 | 45.92 | **-85.99** | **-59.27** | 27.26 | **-88.15** | 91.63 |
| Colon Cancer |  |  |  |  |  |  |  |  |
| COLO 205 | 117.58 | 101.01 | 89.95 | **-93.46** | **-82.88** | **-59.64** | **-78.53** | 99.72 |
| HCC-2998 | 96.79 | 93.84 | 92.37 | **-82.28** | **-83.62** | **-62.37** | **-97.17** | 106.20 |
| HCT-116 | 99.24 | 87.02 | 112.06 | -25.62 | **-89.34** | 24.10 | **-98.59** | 104.09 |
| HCT-15 | 99.22 | 96.80 | 91.28 | **-82.88** | **-68.09** | 15.65 | **-96.57** | 98.78 |
| HT29 | 101.93 | 94.12 | 98.81 | **-70.28** | **-54.40** | 1.44 | **-83.94** | 102.59 |
| KM12 | 103.04 | 100.24 | 85.32 | **-70.02** | **-70.88** | 45.72 | **-96.90** | 107.71 |
| SW-620 | 99.40 | 100.22 | 86.99 | 7.47 | **-87.06** | 64.34 | **-78.48** | 111.78 |
| CNS Cancer |  |  |  |  |  |  |  |  |
| SF-268 | 106.97 | 83.21 | 90.83 | 0.04 | **-55.08** | 42.49 | **-81.88** | 97.29 |
| SF-295 | 101.29 | 86.91 | 96.17 | **-84.82** | **-86.72** | 70.25 | **-90.97** | 89.47 |
| SF-539 | 96.57 | 91.86 | 88.12 | **-96.98** | **-98.47** | 14.67 | **-98.50** | 101.25 |
| SNB-19 | 97.41 | 89.30 | 90.58 | -3.08 | **-89.11** | 49.08 | **-93.57** | 94.37 |
| SNB-75 | 84.92 | 92.60 | 85.28 | 2.76 | **-68.81** | -0.13 | **-86.41** | 68.29 |
| U251 | 99.27 | 87.16 | 89.89 | **-88.44** | **-78.09** | 40.30 | **-79.05** | 93.43 |
| Melanoma |  |  |  |  |  |  |  |  |
| LOX IMVI | 97.48 | 88.62 | 84.05 | **-97.23** | **-54.44** | 5.04 | **-98.79** | 96.62 |
| MALME-3M | 90.82 | 100.62 | 77.15 | **-90.33** | **-95.20** | **-97.20** | **-90.98** | 102.14 |
| M14 | 94.69 | 95.68 | 103.11 | **-75.52** | **-88.87** | **-77.89** | **-94.82** | 100.61 |
| MDA-MB-435 | 89.01 | 104.27 | 28.86 | **-78.93** | **-95.34** | 38.73 | **-78.74** | 104.30 |
| SK-MEL-2 | 116.34 | 103.22 | 84.66 | **-93.88** | **-72.57** | -26.07 | **-89.47** | 108.89 |
| SK-MEL-28 | 92.11 | 105.16 | 84.95 | **-92.07** | **-94.83** | -45.32 | **-96.55** | 104.80 |
| SK-MEL-5 | 90.52 | 84.26 | 74.46 | **-98.54** | **-98.45** | **-98.27** | **-99.56** | 75.56 |
| UACC-257 | 96.66 | 88.38 | 90.12 | **-79.28** | **-82.25** | **-72.67** | **-78.15** | 100.97 |
| UACC-62 | 90.70 | 83.77 | 83.19 | **-90.93** | **-91.48** | 34.74 | **-92.55** | 86.42 |
| Ovarian Cancer |  |  |  |  |  |  |  |  |
| IGROV1 | 95.78 | 100.01 | 101.75 | -28.87 | **-71.05** | 25.10 | **-77.05** | 109.27 |
| OVCAR-3 | 111.51 | 99.72 | 78.09 | **-71.51** | **-68.43** | 50.60 | **-82.99** | 120.93 |
| OVCAR-4 | 95.81 | 83.92 | 101.13 | -18.95 | -32.07 | 33.00 | -41.47 | 93.95 |
| OVCAR-5 | 91.18 | 100.64 | 96.44 | -31.15 | **-86.01** | 81.16 | **-91.97** | 111.57 |
| OVCAR-8 | 102.67 | 93.40 | 92.88 | -47.44 | -41.98 | 66.00 | **-69.31** | 103.70 |
| NCI/ADR-RES | 99.43 | 89.24 | 94.68 | -10.89 | n.d. | 42.02 | **-80.80** | 91.85 |
| SK-OV-3 | n.d. | 101.35 | 93.27 | 23.57 | -43.99 | 70.01 | **-72.54** | 108.34 |
| Renal Cancer |  |  |  |  |  |  |  |  |
| 786-0 | 97.71 | 94.26 | 105.98 | **-72.67** | **-95.68** | 31.44 | **-96.37** | 94.21 |
| A498 | 103.88 | 90.09 | 83.23 | -15.07 | **-93.94** | 58.33 | **-97.15** | 106.75 |
| ACHN | 95.98 | 99.75 | 103.15 | -20.27 | **-78.08** | 33.71 | **-69.38** | 103.04 |
| CAKI-1 | 98.15 | 80.94 | 54.48 | **-64.98** | **-78.05** | 5.13 | **-86.20** | 79.78 |
| RXF 393 | 104.37 | 99.45 | 72.96 | **-91.93** | **-85.28** | -8.13 | **-94.49** | 101.93 |
| SN12C | 98.98 | 97.37 | 89.14 | **-66.44** | -4.35 | 43.91 | **-95.04** | 103.12 |
| TK-10 | 93.30 | 100.47 | 99.04 | -48.54 | **-81.66** | 42.58 | **-90.39** | 104.22 |
| UO-31 | 87.63 | 82.52 | 75.84 | 2.06 | **-69.49** | 20.69 | **-97.81** | 80.63 |
| Prostate Cancer |  |  |  |  |  |  |  |  |
| PC-3 | 96.52 | 79.02 | 92.88 | -25.38 | **-72.04** | 12.90 | **-72.28** | 80.54 |
| DU-145 | 110.30 | 99.61 | 99.62 | -38.77 | **-64.90** | 39.49 | **-94.44** | 104.78 |
| Breast Cancer |  |  |  |  |  |  |  |  |
| MCF7 | 95.71 | 80.27 | 78.04 | **-51.53** | **-74.26** | 6.31 | **-88.17** | 88.74 |
| MDA-MB-231/ATCC | 100.52 | 90.63 | 80.42 | **-67.42** | **-94.37** | 51.47 | **-93.97** | 101.86 |
| HS 578T | 100.38 | 94.50 | 78.59 | -46.24 | **-61.09** | 56.40 | **-60.05** | 96.14 |
| BT-549 | 100.92 | 99.10 | n.d. | **-69.09** | **-92.41** | 16.31 | **-92.43** | 96.76 |
| T-47D | 108.46 | 77.23 | 90.91 | -11.39 | -19.96 | 28.78 | **-64.63** | 82.24 |
| MDA-MB-468 | 92.15 | 95.74 | 70.45 | **-83.37** | **-58.18** | n.d. | n.d. | n.d. |
| Mean |  |  |  |  |  |  |  |  |
|  | 98.85 | 92.48 | 87.85 | -44.47 | **-71.75** | 20.38 | **-79.86** | 96.85 |

**Table S3.** Growth percent rates of 17-(*O*-3-carboxypropanoyl)vindoline (**15**) and compounds **27**-**32** at the concentration of 10 μ*M* against 60 human cancer cell lines *in vitro*. All data with greater than 50% loss of cells are highlighted in bold.

|  | **15** | **27** | **28** | **29** | **30** | **31** | **32** |
| --- | --- | --- | --- | --- | --- | --- | --- |
| Leukemia |  |  |  |  |  |  |  |
| CCRF-CEM | 104.88 | 103.55 | 4.40 | 3.80 | 61.40 | **-81.88** | 86.75 |
| HL-60(TB) | 114.45 | 111.62 | 4.32 | **-58.16** | 82.15 | **-98.40** | 90.87 |
| K-562 | 90.54 | 124.01 | 16.91 | -12.50 | 65.16 | 7.65 | 101.84 |
| MOLT-4 | 105.57 | 100.26 | 18.74 | -4.63 | 59.43 | **-98.81** | 85.81 |
| RPMI-8226 | 84.25 | 94.49 | 8.80 | -14.18 | 46.29 | **-98.14** | 99.38 |
| SR | n.d. | n.d. | 18.44 | -48.31 | 56.41 | n.d. | 117.39 |
| Non-small Cell Lung Cancer |  |  |  |  |  |  |  |
| A549/ATCC | 94.26 | 94.19 | 13.55 | 25.32 | 55.37 | 1.83 | 95.19 |
| EKVX | 87.44 | 90.69 | 20.85 | 25.36 | 60.18 | -17.77 | 99.42 |
| HOP-62 | 102.49 | 118.56 | 30.39 | 22.02 | 88.39 | **-57.01** | 96.20 |
| HOP-92 | 98.83 | 105.12 | -3.33 | -22.63 | 66.38 | -44.91 | 92.40 |
| NCI-H226 | 66.56 | 80.42 | 14.89 | 16.70 | 69.88 | -32.35 | 115.10 |
| NCI-H23 | 92.47 | 98.93 | 8.32 | 29.43 | 83.42 | -23.37 | 100.56 |
| NCI-H322M | 73.92 | 109.81 | 43.73 | 35.40 | 97.16 | -0.86 | 114.10 |
| NCI-H460 | 97.43 | 101.77 | 1.83 | -11.38 | 83.42 | **-60.93** | 107.08 |
| NCI-H522 | 84.17 | 84.45 | -9.16 | 28.24 | 59.68 | -22.21 | 81.41 |
| Colon Cancer |  |  |  |  |  |  |  |
| COLO 205 | 93.56 | 117.79 | **-90.33** | **-91.01** | 66.74 | -44.97 | 45.78 |
| HCC-2998 | 71.05 | 86.51 | **-75.20** | **-52.65** | 85.26 | **-66.87** | 112.65 |
| HCT-116 | 98.61 | 102.99 | 29.42 | 7.59 | 67.76 | -32.37 | 119.10 |
| HCT-15 | 98.39 | 103.18 | 6.71 | 15.04 | 79.50 | 3.11 | 100.06 |
| HT29 | 89.90 | 96.68 | **-50.41** | -10.02 | 60.48 | **-83.35** | 102.49 |
| KM12 | 82.78 | 100.29 | 3.83 | 3.78 | 83.57 | **-50.82** | 100.12 |
| SW-620 | 101.60 | 104.68 | 20.74 | 9.15 | 102.23 | -36.98 | 117.35 |
| CNS Cancer |  |  |  |  |  |  |  |
| SF-268 | 87.17 | 99.45 | 13.11 | 14.59 | 50.78 | 7.32 | 92.24 |
| SF-295 | 113.13 | 97.94 | 1.73 | 7.76 | 67.90 | **-61.14** | 95.25 |
| SF-539 | 92.91 | 100.39 | 8.28 | 11.65 | 78.68 | -38.41 | 98.83 |
| SNB-19 | 94.90 | 108.08 | 16.17 | 20.07 | 69.49 | 8.85 | 91.19 |
| SNB-75 | 53.42 | 64.34 | 26.24 | 7.51 | 64.55 | -43.51 | 108.78 |
| U251 | 104.99 | 109.62 | 12.39 | 19.09 | 71.61 | -15.09 | 97.44 |
| Melanoma |  |  |  |  |  |  |  |
| LOX IMVI | 106.58 | 93.00 | **-75.78** | -7.07 | 71.76 | **-56.04** | 97.77 |
| MALME-3M | 90.34 | 112.25 | 2.45 | -26.51 | 93.31 | -16.58 | 81.97 |
| M14 | 96.50 | 114.34 | **-66.75** | -20.29 | 60.96 | 0.99 | 88.35 |
| MDA-MB-435 | 109.94 | 120.53 | 5.70 | 3.59 | 85.67 | -23.31 | 98.20 |
| SK-MEL-2 | 100.14 | 88.20 | **-84.73** | **-63.35** | 90.19 | **-85.42** | 96.75 |
| SK-MEL-28 | 86.69 | 94.81 | 14.99 | 7.72 | 104.30 | -6.67 | 67.58 |
| SK-MEL-5 | 96.76 | 91.49 | **-92.46** | **-90.94** | 62.60 | **-87.99** | 83.85 |
| UACC-257 | 91.53 | 99.17 | 19.49 | 22.56 | 85.95 | -30.78 | 61.71 |
| UACC-62 | 92.39 | 112.09 | **-75.77** | -19.73 | 67.09 | **-84.26** | 59.50 |
| Ovarian Cancer |  |  |  |  |  |  |  |
| IGROV1 | 89.95 | 96.75 | -42.31 | -31.20 | 68.39 | **-92.21** | 126.15 |
| OVCAR-3 | 74.90 | 89.18 | -2.22 | 0.33 | 63.74 | **-71.03** | 99.79 |
| OVCAR-4 | 80.15 | 104.84 | 6.50 | 10.46 | 56.41 | -34.64 | 98.12 |
| OVCAR-5 | n.d. | n.d. | 2.55 | 15.22 | 96.28 | n.d. | 104.64 |
| OVCAR-8 | 91.77 | 99.56 | 10.72 | 23.39 | 73.69 | -13.16 | 100.12 |
| NCI/ADR-RES | 94.58 | 107.30 | 5.44 | n.d. | 65.21 | **-62.13** | 106.06 |
| SK-OV-3 | 92.30 | 106.47 | 37.48 | 12.44 | 103.23 | -15.61 | 111.06 |
| Renal Cancer |  |  |  |  |  |  |  |
| 786-0 | 98.06 | 99.66 | 20.71 | 11.86 | 84.20 | -40.27 | 104.22 |
| A498 | 90.59 | 105.29 | 21.62 | 20.09 | 109.73 | **-61.65** | 116.53 |
| ACHN | 102.24 | 99.15 | -3.87 | 3.70 | 71.29 | -35.75 | 103.22 |
| CAKI-1 | 95.47 | 96.62 | -10.21 | 6.02 | 28.12 | 1.73 | 78.40 |
| RXF 393 | 96.64 | 82.35 | -12.87 | 10.17 | 60.14 | **-56.33** | 102.12 |
| SN12C | 92.92 | 86.69 | 9.74 | 14.82 | 71.21 | **-89.11** | 94.40 |
| TK-10 | 90.85 | 91.93 | -5.03 | 45.68 | 60.61 | -32.11 | 103.17 |
| UO-31 | n.d. | n.d. | 10.27 | 5.16 | 62.29 | n.d. | 98.80 |
| Prostate Cancer |  |  |  |  |  |  |  |
| PC-3 | 87.70 | 100.70 | 3.75 | 2.13 | 44.53 | -28.47 | 90.59 |
| DU-145 | 98.07 | 103.00 | 15.45 | 20.31 | 82.64 | -3.52 | 115.61 |
| Breast Cancer |  |  |  |  |  |  |  |
| MCF7 | 68.25 | 81.00 | 3.97 | 10.93 | 62.25 | -28.67 | 91.50 |
| MDA-MB-231/ATCC | n.d. | n.d. | -46.97 | 3.02 | 65.30 | n.d. | 91.10 |
| HS 578T | 69.34 | 70.82 | 20.15 | 0.42 | 78.54 | -32.58 | 102.61 |
| BT-549 | 80.50 | 88.60 | 12.90 | **-54.49** | 98.10 | **-60.64** | 122.02 |
| T-47D | n.d. | n.d. | 38.35 | 24.04 | 73.85 | n.d. | 92.52 |
| MDA-MB-468 | 98.72 | 124.29 | -42.84 | -20.49 | 1.38 | **-69.09** | 78.05 |
| Mean |  |  |  |  |  |  |  |
|  | 91.70 | 99.45 | -3.07 | -1.41 | 71.44 | -41.76 | 97.19 |

**S.3. NCI60 Screening**

A detailed description of the NCI screening procedures [30-34] can also be found on the website of NCI [35], and in our previous work [26].

**S.3.1. One-Dose Screen**

All candidates were examined at first at a single high dose (10 µ*M*) in the full NCI60 cell panel [30-34]. The value reported for the one-dose test is growth relative to the no-drug control and relative to the time zero number of cells. This made possible the disclosure of both growth inhibition (numbers between 0 and 100) and lethality (numbers less than 0). For example, a value of 100 means no growth inhibition. A value of 20 would mean 80% growth inhibition. A value of 0 means no net growth over the course of the analysis. A value of −20 would mean 20% lethality. A value of −100 means all cells are dead.

**S.3.2. Five-Dose Screen**

Candidates that showed remarkable growth inhibition in the one-dose assay were subjected to the 60-cell panel at five concentration levels. The human tumor cell lines of the cancer screening panel were grown in RPMI 1640 medium containing 5% fetal bovine serum and 2 m*M* l-glutamine. Characteristically, cells were injected in 96-well microtiter plates in 0.1 mL at plating densities ranging from 5000 to 40,000 cells/well, depending on the doubling time of individual cell lines. After cell inoculation, the microtiter plates were incubated at 37 °C, 5% carbon dioxide, 95% air, and 100% relative humidity for 24 hours before the addition of tested compounds. After 24 hours, 2 plates of each cell line were fixed *in situ* with Cl_3_COOH (TCA), to represent a measurement of the cell population for each cell line at the time of drug addition (*t*_z_). Tested compounds were solubilized in DMSO at 400-fold the desired final maximum test concentration and stored frozen before application. In the course of the drug addition, an aliquot of frozen concentrate was thawed and diluted to twice the desired final maximum test concentration with a complete medium containing 50 μg mL^−1^ gentamicin. Additional four, 10-fold or ½ log serial dilutions were produced to furnish a total of five drug concentrations plus control. Aliquots of 0.1 mL of these different drug dilutions were added to the appropriate microtiter wells already containing 0.1 mL of medium, resulting in the required final drug concentrations.

Following drug addition, the plates were incubated at 37 °C, 5% carbon dioxide, 95% air, and 100% relative humidity for an additional 48 hours. For adherent cells, the test was finished by the addition of cold Cl_3_COOH. Cells were fixed *in situ* by the addition of 50 μL of cold 50% (w/v) Cl_3_COOH, and incubated at 4 °C for 1 hour. The supernatant was discarded, and the plates were washed with H_2_O (5×) and dried in air. Sulforhodamine B (SRB) solution (0.1 mL) at 0.4% (w/v) in 1% CH_3_COOH was added to each well, and plates were incubated at RT for 10 min. After staining, the unbound dye was removed by washing five times with 1% CH_3_COOH, and the plates were dried in the air. The bound stain is afterward solubilized with a 10 mM trizma base, and the absorbance is read on an automated plate reader at λ = 515 nm. Utilizing the seven absorbance measurements [time zero (*t*_z_), control growth (*c*), and test growth in the presence of the drug at the five concentration levels (*t*_i_)], the percentage growth was determined at each of the drug concentration levels. Growth inhibition (%) was calculated as:

[(*t*_i_ − *t*_z_)/(*c* − *t*_z_)] × 100, for concentrations where *t*_i_ ≥ *t*_z_ (1)

[(*t*_i_ − *t*_z_)/(*t*_z_)] × 100, for concentrations where *t*_i_ < *t*_z_. (2)

Three dose‒response parameters were calculated as follows. *GI*_50_ (growth inhibition of 50%) was determined from Equation (3), which is the drug concentration resulting in a 50% reduction in the net protein increase (as measured by SRB staining) in control cells in the course of the drug incubation.

[(*t*_i_ − *t*_z_)/(*c* − *t*_z_)] × 100 = 50 (3)
